# Free‐Standing Supramolecular Pyridine *N*‐Oxide‐Silver(I) Metallogels

**DOI:** 10.1002/adma.202502818

**Published:** 2025-07-01

**Authors:** Rakesh Puttreddy, Boonya Thongrom, J. Mikko Rautiainen, Manu Lahtinen, Esa Kukkonen, Rainer Haag, Jani O. Moilanen, Jan Lundell, Kari Rissanen

**Affiliations:** ^1^ University of Jyvaskyla Department of chemistry University of Jyvaskyla P.O. BOX 35 Jyvaskyla FI‐40014 Finland; ^2^ Institute for Chemistry and Biochemistry Freie Universität Berlin Takustrasse 3 14195 Berlin Germany

**Keywords:** metallogels, N‐oxide, shaping, silver(I), structuring

## Abstract

Twenty‐seven pyridine *N*‐oxides (PyNOs) are investigated to evaluate the gelation of their silver(I) trifluoroacetate (AgTFA) complexes across eight solvents. Gelation occurs selectively with PyNOs featuring electron‐donating groups, while those with electron‐withdrawing or mixed groups do not form gels. A combination of two different PyNOs, one with an electron‐donating group and the second with an electron‐withdrawing group, form a gel, suggesting that gel‐forming PyNO‐AgTFA can override the non‐gelling tendency of PyNOs comprising electron‐withdrawing groups. Pyridine‐AgTFA complexes lacking the N–O group fail to gel, underscoring the crucial role of the N–O functionality and its coordination with silver(I) in facilitating gelation. The resulting PyNO‐AgTFA gels demonstrate remarkable mechanical strengths, enabling the fabrication of free‐standing and load‐bearing gel shapes, such as rods and horseshoes, with sizes up to several centimeters. The analysis of 65 X‐ray crystal structures reveals that PyNO‐AgTFA complexes manifest four distinct structural motifs, even when crystallized under different solvents and ligand‐to‐metal ratios, demonstrating a strong preference for a specific set of silver(I) complexes. X‐ray crystallography and powder X‐ray diffraction studies predict gel structures without single crystals. Density functional theory calculations of recurring non‐covalent interactions in the crystal structures show interaction energies ranging from −1 to −92 kJ mol^−1^.

## Graphical abstract

A comprehensive analysis of pyridine *N*‐oxide‐silver(I) trifluoroacetate complexes reveals remarkable mechanical strengths that enable the creation of free‐standing objects with shape control, reaching dimensions of up to centimeters and shedding new insights into the gelation mechanisms.

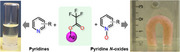

## 1 Introduction

Supramolecular chemistry, the study of non‐covalent interactions (NCIs), plays an important role in various aspects of daily life, particularly in the formulation of gels that promote health and wellness.^[^ ^1^ ^]^ Gels are essential in everyday products such as toothpaste, hair gels, face cleansers, hand lotions, and detergents, all of which rely on supramolecular principles. The International Union of Pure and Applied Chemistry (IUPAC)^[^ ^2^ ^]^ defines a gel as a “nonfluid colloidal or polymer network that is expanded throughout its whole volume by a fluid”. This definition essentially refers gels consisting of a small amount of cross‐linked solid material (gelator) that immobilizes a large volume of liquid through surface tension^[^ ^3^ ^]^ and capillary forces.^[^ ^4^ ^]^ Gels can be synthesized using various methods, with the bottom‐up supramolecular approach being particularly effective.^[^ ^5^ ^]^ This approach relies on the spontaneous self‐assembly of small molecules, or Low Molecular Weight Gelators (LMWGs), into three‐dimensional networks through NCIs such as hydrogen bonding (HB)^[^ ^6^ ^]^ and π–π interactions.^[^ ^7^ ^]^ Successful gelation hinges on the strength and balance of NCIs between gelators, as well as the finely tuned interplay of specific (e.g., HB) and non‐specific (e.g., dipole‐induced) NCIs with the solvent.^[^ ^8^ ^]^ Thus, mastering control over the self‐assembly of LMWGs and their NCIs is crucial for effective gel formation but remains an extremely challenging task. One powerful strategy to streamline the molecular complexity of LMWG networks and circumvent “chaotic” self‐assembly is to “stitch” gelators together using metals.^[^ ^9^ ^]^ This approach incorporates metal‐ligand coordination bonds (CBs) to orchestrate the ordered self‐assembly of LMWGs driven by metal‐specific geometries. A wide variety of metallogels formed via metal coordination have been reported, including discrete structures^[^ ^10^ ^]^ and coordination polymers.^[^ ^11^ ^]^ The mechanism of metallogelation generally falls into two categories:^[^ ^12^ ^]^ i) gelation triggered by CBs formed upon the addition of metal ions to LMWGs, and ii) direct gelation by metal complexes that immobilize solvents through NCIs facilitated by metal‐metal and metal‐LMWGs interactions. If LMWGs fail to gel with metals, they can often be chemically modified to promote gelation. Strategies include changing the metal cations^[^ ^13^ ^]^ and counter anions,^[^ ^14^ ^]^ or introducing long alkyl and polyether chains to the gelator structure.^[^ ^13^ ^]^ Among the known systems, metallogels incorporating pyridines,^[^ ^15^ ^]^ phenolates,^[^ ^16^ ^]^ and carboxylates^[^ ^15^ ^]^ are prevalent, however, there remains a significant gap in the use of pyridine *N*‐oxides (PyNOs) in metallogels. To date, only a few examples of PyNO‐organogels have been reported,^[^ ^17^ ^]^ highlighting the need for further exploration in this area.

Molecules featuring the *N*‐oxide group play critical roles in biological processes.^[^ ^18^ ^]^ For instance, trimethylamine *N*‐oxide, a natural protein stabilizer, is found at high concentrations within deep‐sea fish.^[^ ^19^ ^]^ In healthcare, synthetic *N*‐oxides serve important functions as prodrugs^[^ ^20^ ^]^ and MRI agents.^[^ ^21^ ^]^ Additionally, hydrophilic oligomeric *N*‐oxides demonstrate blood compatibility, making them promising stealth agents for pharmaceutical surface conjugation.^[^ ^22^ ^]^ These diverse applications rely on the unique chemical properties of the N–O group. On the other hand, silver(I) complexes are well‐established for their antibiotics and antiseptic properties and are widely used in medical settings.^[^ ^23^ ^]^ Applications include cardiac devices,^[^ ^24^ ^]^ tissue implants,^[^ ^25^ ^]^ catheters^[^ ^26^ ^]^ and water purification systems.^[^ ^27^ ^]^ Given the biological significance of PyNOs and silver(I), there is a strong incentive to explore PyNO‐silver(I) gels for potential healthcare applications. However, a fundamental challenge remains: the relationship between the supramolecular structure of PyNO‐silver(I) complexes and their gelation behavior is not yet well understood. Addressing this fundamental knowledge gap is essential for the rational design of smarter and more effective gel materials for technological and biomedical applications.

**Figure 1:**
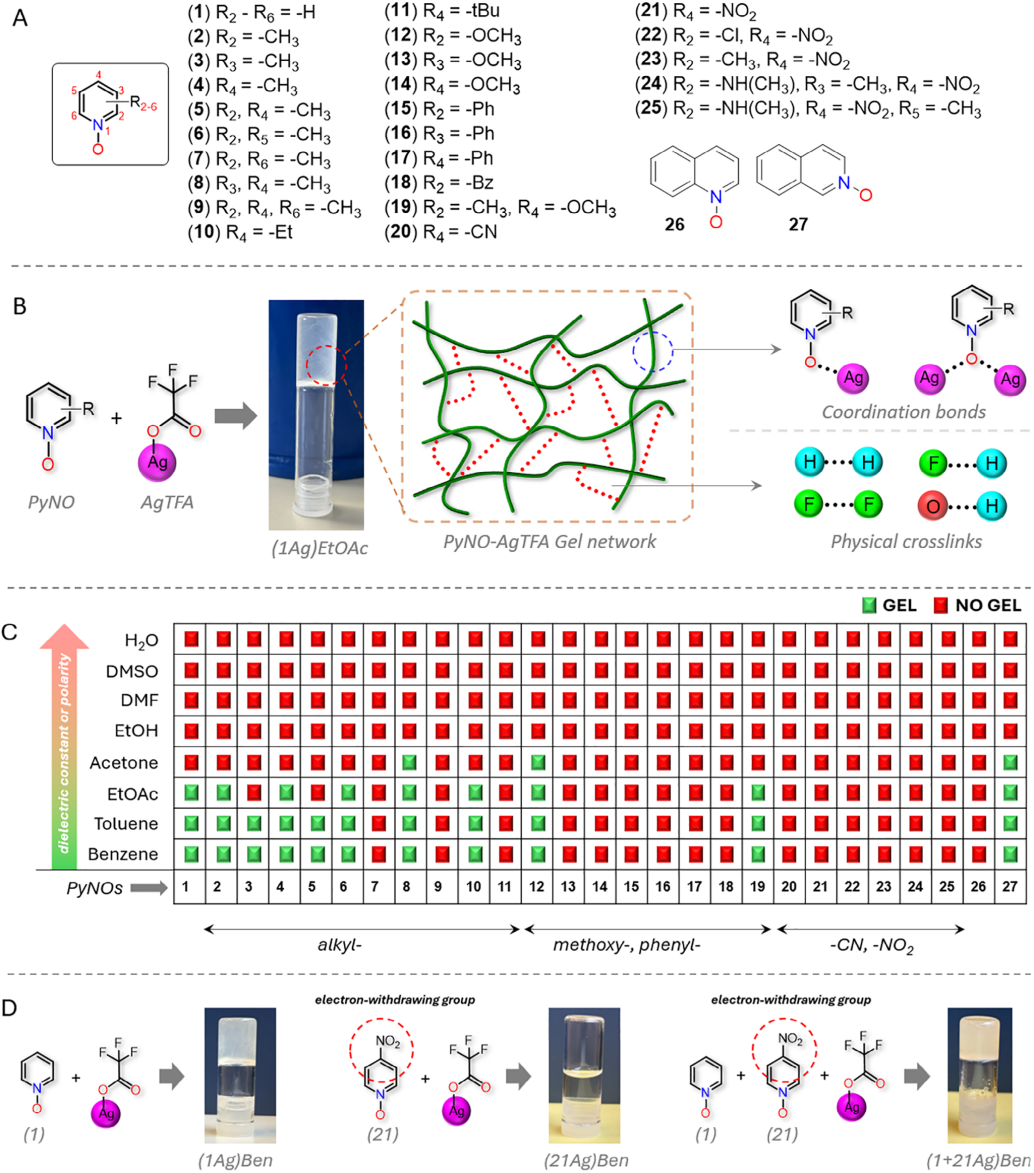
The components, gel network and the summary of the gelation experiments. A) A list of the pyridine *N*‐oxides (**1** – **27**) utilized in gelation studies. B) PyNO‐AgTFA gel network depicting coordination and physical crosslinks. C) A summary of gelation experiments. D) An example showing gelation and non‐gelation capabilities of single and mixed PyNO‐AgTFA systems.

To initiate this exploration, we examine the gelation properties of PyNO‐silver(I) complexes through a comprehensive study involving 27 PyNOs (**Figure** **1**A). Understanding the gelation mechanism from the perspective of PyNOs is challenging due to the diverse coordination geometries silver(I) ions can adopt.^[^ ^28^ ^]^ This variability stems from their *d* ^10^ electronic configuration and sensitivity to packing forces. To reduce structural complexity and ensure consistency across the study, we use AgTFA as the silver(I) source. The trifluoroacetate group in AgTFA reliably forms a well‐defined paddlewheel structure with silver(I) ions,^[^ ^29^ ^]^ offering a robust and predictable building block for the systems under investigation.

## 2 Results and Discussion

### 2.1 Gelation Studies

The gelation capability of 27 PyNOs with AgTFA is rigorously assessed using a 1:1 PyNO:AgTFA equivalent ratio (0.25/0.5 w/v% PyNO and 0.30–0.58/0.6–1.2 w/v% AgTFA in 1.0 mL) across eight solvents: benzene (Ben, dielectric constant (ɛ at 25 °C) = 2.3),^[^ ^30^ ^]^ toluene (Tol, ɛ = 2.4), ethyl acetate (EtOAc, ɛ = 6), acetone (Acet, ɛ = 20.7), ethanol (EtOH, ɛ = 24.3), dimethylformamide (DMF, ɛ = 36.7), dimethyl sulfoxide (DMSO, ɛ = 46.7), and water (H_2_O, ɛ = 78.5). A higher dielectric constant (ɛ) corresponds to greater solvent polarity. In the standard procedure, PyNO is dissolved in 0.5 mL of the solvent and AgTFA in a separate 0.5 mL of the same solvent, and the two solutions are subsequently combined (For more details, see Supporting Information). Gelation is evaluated using the “inversion tube test” (e.g., Figure 1B). These experiments reveal that 11 PyNOs can form gels in one or more of four solvents, benzene, toluene, EtOAc, and acetone (Tables S1–S4, Supporting Information). The gelation experiments in these solvents were repeated three times, while experiments involving gel‐forming PyNO‐AgTFA experiments were repeated five times to ensure reproducibility. Overall, benzene, toluene, ethyl acetate, and acetone prove to be good solvents for gel formation, while ethanol, DMF, DMSO, and water are unsuitable (Figure 1C). These differences can be attributed to variations in solvation power, polarity and HB abilities among solvents,^[^ ^31^ ^]^ all of which impact the gelator‐gelator and solvent‐gelator NCIs and physical crosslinks critical for gel assembly (Figure 1B). From a PyNO structural perspective, PyNOs bearing alkyl, methoxy, ethyl, *tert*‐butyl, and isoquinoline groups exhibit effective gelation, whereas other PyNO variants did not. The critical gel concentrations (CGC) of 11 gel‐forming PyNO‐AgTFA complexes in toluene were determined by varying PyNO concentrations from 0.025 w/v% and upward. Their CGCs range between 0.09 and 0.30 w/v% PyNO concentrations, with **(12Ag)Tol**, gelling at 0.09 w/v% and **(19Ag)Tol** and **(27Ag)Tol** gelling at 0.1 w/v%. These three silver(I) complexes can be categorized as supergelators (Table S11, Supporting Information). Pyridines‐based silver(I) gels are well‐known.^[^ ^32^ ^]^ To probe the role of the N–O group, the unoxidized parent pyridines of PyNOs featuring electron‐donating groups are tested under 0.25 w/v% pyridine concentrations across eight solvents (Tables S5 and S6, Supporting Information). The pyridine‐AgTFA complexes, even at tenfold higher concentrations of pyridines, fail to form gels across eight solvents. These results underscore the essential role of the N–O group and its ability to engage in silver(I) coordination critical for facilitating gelation.

The gelation ability of 4‐methylpyridine *N*‐oxide (**4**) with other silver(I) salts, AgBF_4_, AgPF_6_, AgSbF_6_, AgClO_4_, and AgSO_3_CF_3_ is evaluated in eight different solvents using a 1:1 PyNO:Ag(I) salt equivalent ratio. Among these, only the combination of **4** and AgClO_4_ in acetone resulted in gel formation, all other systems failed to gel under the tested conditions (Table S7, Supporting Information). Although these non‐AgTFA complexes could form gels upon variation of parameters such as increased concentrations of PyNO and silver(I) salts, optimizing these conditions falls outside the scope of the present study. Our subsequent investigations focus exclusively on PyNO‐AgTFA complexes. For clarity in the following discussions: i) The label **(1Ag)EtOAc** refers to the complex formed from pyridine *N*‐oxide (**1**) and AgTFA in EtOAc, ii) the **1Ag** label broadly denotes the silver(I) complex of **1** with AgTFA, and iii) only PyNOs concentrations are reported as the w/v%.

The impact of the PyNO:AgTFA ratio on gelation in various solvents, EtOAc, acetone, EtOH, DMF, DMSO, and H_2_O is examined using 4‐methylpyridine *N*‐oxide (**4**). These six solvents are selected because **4Ag** at a 0.25 w/v% PyNO concentration forms gel in benzene, toluene and EtOAc, but not in the others. Given that acetone and EtOAc possess carbonyl functionalities, EtOAc serves as a reference solvent. Three sets of ratio tests were performed: i) increasing the concentrations of **4** and AgTFA while maintaining a 1:1 **4**:AgTFA equivalent ratio (**Table** **1**, Entries 1–4); ii) increasing the AgTFA concentration while maintaining **4** at 0.25 w/v% PyNO (Entries 5–7); and iii) increasing the PyNO concentration while fixing AgTFA constant at 0.5 w/v% (Entries 8–10). **4** and AgTFA consistently gels in EtOAc across all tested concentrations, whereas in acetone, gelation does not occur. This highlights a strong solvent‐dependent effect, with acetone's polarity likely influencing the gelator‐gelator and solvent‐gelator NCIs and physical crosslinks despite structural similarities to EtOAc. **4** and AgTFA do not form gels in EtOH, DMF, DMSO, and H_2_O at any tested concentrations, which can be attributed to the large polarities and HB capabilities of these solvents.^[^ ^8^ ^]^

**Table 1:** Ligand‐to‐silver(I) ratio study using 4‐methylpyridine *N*‐oxide‐AgTFA in EtOAc and acetone.

Entry	4:AgTFA equivalent ratio	4:AgTFA w/v% ratio	EtOAc	XRD	Acetone	XRD
1	1:1	0.25:0.5	Gels	No	No gel	Yes
2	1:1	0.5:1.0	Gels	No	No gel	No
3	1:1	1.0:2.0	Gels	No	No gel	No
4	1:1	1.5:3.0	Gels	No	No gel	No
5	1:2	0.25:1.0	Gels	No	No gel	Yes
6	1:3	0.25:1.5	Gels	No	No gel	Yes
7	1:4	0.25:2.0	Gels	No	No gel	Yes
8	2:1	0.5:0.5	Gels	No	No gel	No
9	3:1	0.75:0.5	Gels	No	No gel	No
10	4:1	1.0:0.5	Gels	No	No gel	No

Sonication (45 kHz, 25 °C) and 80 °C heating ↔ room temperature cooling cycles fail to trigger gelation of PyNO‐AgTFA complexes that inherently lack gelation ability. PyNOs featuring electron‐withdrawing groups consistently fail to form gels, even in hydrophobic, non‐polar solvents such as benzene and toluene, regardless of PyNO and AgTFA concentrations. For instance, neither 4‐cyano‐ (**20**) nor 4‐nitropyridine *N*‐oxide (**21**) form gels at 1:4 and 4:1 PyNO:AgTFA equivalent ratios in benzene, toluene, EtOAc, and acetone (Table S8, Supporting Information). Similarly, PyNOs with both electron‐donating and electron‐withdrawing groups (e.g., **22**–**25**) also fail to gel, suggesting that NCIs imparted by electron‐withdrawing groups are either too weak or strong to immobilize the solvent, or that gelation is solvent dependent. To differentiate electronic effects from solvent effects, pyridine *N*‐oxide (**1**), 2‐methyl (**2**), and 4‐methylpyridine *N*‐oxide (**4**) are mixed with 4‐nitropyridine *N*‐oxide (**21**) and 2‐methyl‐4‐nitropyridine *N*‐oxide (**23**) and AgTFA in benzene, toluene, EtOAc, acetone, and EtOH (Table S9, Supporting Information). Note that **1Ag**, **2Ag**, and **4Ag** at 0.25 w/v% gels in benzene, toluene, and EtOAc but not in acetone and EtOH, whereas **21Ag** and **23Ag** fail to gel in all solvents. If the ─NO_2_ group inhibits gelation, mixtures such as (**1**+**21)Ag**, (**1**+**23**)**Ag**, (**2**+**21**)**Ag**, (**2**+**23**)**Ag**, (**4**+**21)Ag**, and (**4**+**23**)**Ag** should not gel. However, all these systems successfully gel in benzene, toluene, and EtOAc but not in acetone and EtOH, suggesting that the PyNOs comprising the NO_2_ group do not fundamentally disrupt gelation. Instead, solvent properties, such as polarity, and their differences in HB capabilities govern gelation. Further probing solvent polarity effects, **1**, **2**, and **4** are tested at 0.25 and 0.5 w/v% in aprotic solvents with ɛ < 20.7, including 2‐hexanone (ɛ = 14.5), 2‐pentanone (ɛ = 15.4), and 3‐pentanone (ɛ = 17.3), as well as in protic solvents with ɛ < 24.5, including 6‐methyl‐2‐heptanol (ɛ = 5.25), 1‐hexanol (ɛ = 13.3), 1‐pentanol (ɛ = 13.9), 1‐butanol (ɛ = 17.1), and 1‐propanol (ɛ = 20.1) (Table S10, Supporting Information). **1Ag**, **2Ag**, and **4Ag**, gel in all aprotic solvents but fail to gel in any protic solvents, regardless of solvent dielectric constant or alkyl chain length, suggesting that the ─OH group and associated HB capability suppress the gelation.

**Figure 2:**
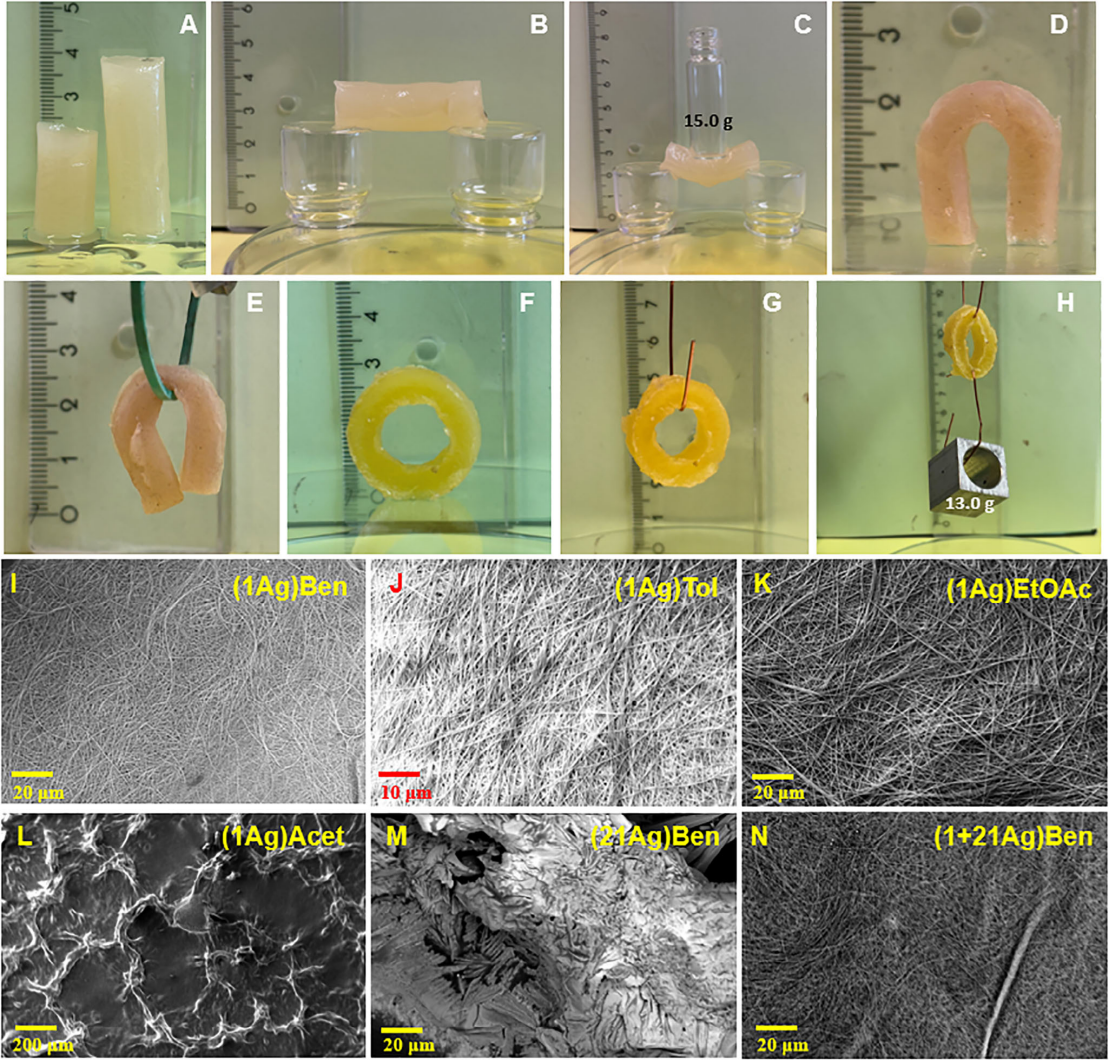
Photographs free‐standing gels and SEM photographs. A) **(19Ag)Tol**’s free‐standing rod shape gel (short‐rod, L = 2 cm, d = 1.2 cm; long‐rod, L = 4 cm, d = 1.2 cm), B) **(19Ag)Tol**’s long‐rod (L = 4 cm, d = 1.2 cm) balanced on two glass vials, C) **(19Ag)Tol**’s long‐rod (L = 4 cm, d = 1.2 cm) tolerating 15.0g glass vial. D) **(8Ag)Tol**’s horseshoe‐type free‐standing (L×W×H = 2 × 1.5 × 0.3 cm), and E) **(8Ag)Tol**’s horseshoe type hanging gel (L×W×H = 2 × 1.5 × 0.3 cm). F) **(2Ag)Tol**’s O‐shape free‐standing gel (L×W×H = 2 × 2 × 0.3 cm), G) **(2Ag)Tol**’s O‐shape hanging gel (L×W×H = 2 × 2 × 0.3 cm), and H) O‐shape hanging gel bearing 13.0g weight. The SEM photographs of gel and non‐gel samples prepared at 0.25 w/v % PyNO concentration of I) **(1Ag)Ben**, J) **(1Ag)Tol**, K) **(1Ag)EtOAc**, L) **(1Ag)Acet**, M) **(21Ag)Ben**, and N) **(1+21Ag)Ben**.

The PyNO‐AgTFA gels can be readily synthesized on a large scale (Figure S2, Supporting Information), since their individual components are either commercially available at low cost or can be prepared in one step via oxidation of pyridines.^[^ ^33^ ^]^ Newly discovered supramolecular gels have demonstrated the ability to withstand gravitational collapses, as shown by inversion vial tests. These gelators effectively immobilize solvents within the prepared vials, although their shapeable characteristics may vary.^[^ ^34^ ^]^ While supramolecular gels offer superior synthetic tunability and reversibility, their gel networks often lack the robustness of polymer gels, making it challenging to prepare or achieve free‐standing structures.^[^ ^35^ ^]^ To date, only a limited number of free standing shapes made from metallogels,^[^ ^36^ ^]^ hydrogels,^[^ ^37^ ^]^ and others^[^ ^38^ ^]^ have been reported. The shapeability of PyNO‐AgTFA gels is evaluated to assess their potential for free‐standing applications. To prepare gel shapes, 50 °C hot gelator solutions are poured into 3D‐printed molds to test their ability to form free‐standing structures, emulating the process of traditional kitchen jelly preparation. This method is chosen because the rapid gelation upon mixing PyNO and AgTFA makes transferring the gel material into the molds challenging. The PyNO‐AgTFA gels exhibit free‐standing properties. Various shapes, including rods, horseshoe‐like, and O‐ring forms, were successfully prepared using PyNO‐AgTFA, demonstrating the robustness of their silver(I) network (**Figure** **2**A–H). A rod measuring 4 cm in length and 1.2 cm in diameter, resting across two glass vials, maintains a stiff structure (Figure 2B) and remarkably supports up to 15.0 g without releasing toluene. However, applying an 18.0 g weight causes the rod to release toluene (Figure 2C). The O‐shape withstands a weight of 13.0 g without deforming its geometry, demonstrating the mechanical integrity of these metallogels (Figure 2H).

### 2.2 Scanning Electron Microscopy (SEM)

The SEM analysis is performed on PyNO‐AgTFA complexes at 0.25 w/v% PyNO concentration in benzene, toluene, EtOAc, and acetone, and on **1Ag**, **2Ag**, **4Ag**, and **8Ag** in 2‐pentanone, 3‐pentanone, and 2‐hexanone and on different ligand‐to‐metal ratios presented in Table 1. The goal is to identify characteristic gel morphologies. A total of 160 SEM photographs reveal fibrous morphologies for gel samples (Figure 2 and Figures S3–S44, Supporting Information), consistent with previously reported silver(I) metallogels.^[^ ^39^ ^]^ Fibers show considerable variation in size, with a thickness of 10–100 nm, widths of 100 nm–2 µm, and lengths of 100 nm–100 µm, forming densely entangled networks, often stacking heavily upon one another. Many fibers also split longitudinally or branch into two or more separate fibers, increasing the cross‐linking density and gel robustness. In contrast, non‐gelator complexes lack fibrous morphologies. For instance, **1Ag** forms fibers in benzene, toluene, and EtOAc, but not in acetone (Figure 2I–L and Figures S3–S23, Supporting Information). **21Ag**, derived from 4‐nitropyridine *N*‐oxide (**21**), shows crystalline morphologies across four solvents. However, the mixed PyNO **(1+21)Ag** system, which forms gels in benzene, toluene, and EtOAc, displays fibrous morphologies (Figures 2M vs 2N), whereas its non‐gelator counterpart does not (for more examples, see Figures S36–S39, Supporting Information). These morphological differences are largely governed by solvent–gelator interaction strengths.^[^ ^31^ ^]^ Strong gelator–gelator interactions in hydrophobic solvents favor nanofiber formation, whereas strong solvent–gelator interactions likely promote isotropic molecular aggregation, leading to a loss of fibrous structure, as observed in **(1Ag)Acet** and **(21Ag)Ben** (Figure 2L,M)

### 2.3 Rheology

**Figure 3:**
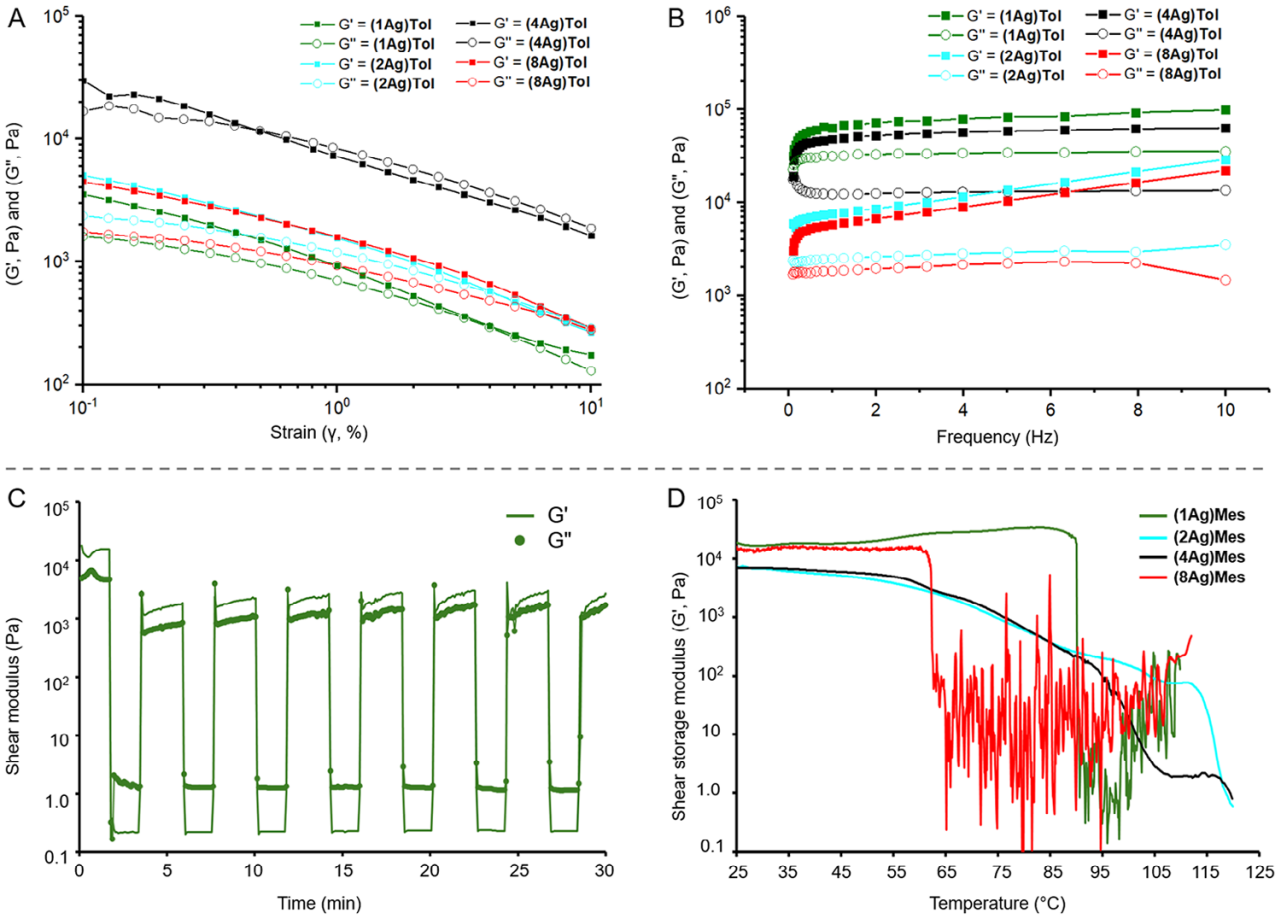
Rheology studies of PyNO‐AgTFA gels. A) Strain‐sweep analysis, and B) Frequency‐sweep analysis of **(1Ag)Tol** – green trace, **(2Ag)Tol –** magenta trace, **(4Ag)Tol** – blue trace, **(8Ag)Tol –** red trace. C) Self‐healing test of **(1Ag)Tol** by step‐strain analysis at 600% and 1% strain in 7 cycles. D) Determination of transition temperature via temperature sweep test of **(1Ag)Mes** – green trace, **(2Ag)Mes –** magenta trace, **(4Ag)Mes** – blue trace, **(8Ag)Mes –** red trace.

The gel strength and mechanical stability of **(1Ag)Tol**, **(2Ag)Tol**, **(4Ag)Tol**, and **(8Ag)Tol** are assessed using rheological amplitude‐sweep measurements by gradually increasing the deformation amplitude (γ) from 0.1 to 10% at a constant frequency of 1Hz. The storage modulus (G′), reflecting the gel's elastic properties, and the loss modulus (G″), reflecting the gel's viscous properties, are assessed. A scenario when G′ exceeds G″ suggests a viscoelastic gel‐like structure, the opposite indicates a viscous liquid‐like behavior.^[^ ^40^ ^]^ As shown in **Figure** **3**A and Figures S45–S48 (Supporting Information), G′ consistently dominates over G″ for all four gels, underscoring their elastic‐like behavior. Increasing shear strain leads to cross‐over points where G′ and G″ intersect, indicating the threshold at which the gel's internal network begins to collapse. Beyond these points, the material transitions from a solid‐like to a flow‐like state. The crossover strain (γ_cp_) is 3% for **(1Ag)Tol** (no distinct crossover but G′ and G″ approach each other), 4% for **(2Ag)Tol**, 0.5% for **(4Ag)Tol**, and 10% for **(8Ag)Tol**. Among these, **(8Ag)Tol** exhibits the greatest resistance and flexibility under increasing shear strain, maintaining its structure before transitioning to a fluid‐like state. The viscoelastic properties of the gels are evaluated across various frequencies under a low, constant strain to preserve the gel network. G′ and G″ values are measured similarly to an amplitude‐sweep test. Gels with highly interconnected networks typically show frequency‐independent G′ values, appearing as horizontal lines (parallel to the *x*‐axis). In contrast, greater frequency dependence indicates weaker network connectivity. All four gels demonstrate G′ dominance at a constant 0.1% shear strain, confirming robust gel networks (Figure 3B and Figures S49–S52, Supporting Information). Although a plateau G′, a measure of gel stiffness, cannot be defined due to frequency sensitivity from physical crosslinking, the storage modulus at 1 Hz serves as a representative measure of stiffness. The average G′ values at 1Hz follow the order: **(1Ag)Tol** (64908 Pa) > **(4Ag)Tol** (47142 Pa) > **(2Ag)Tol** (9943 Pa) > **(8Ag)Tol** (7485 Pa). Among them, **(1Ag)Tol** and **(4Ag)Tol** show G′ values more parallel to the *X*‐axis compared to **(2Ag)Tol** and **(8Ag)Tol**, indicating denser and more stable gel networks. The larger G′ values for unsubstituted and methyl‐substituted PyNO gels can be attributed to stronger NCIs and lower steric hindrance. For example, in **(1Ag)Tol**, the unsubstituted pyridine *N*‐oxide (**1**) can engage freely in NCIs, promoting a strongly interconnected gel network and contributing to higher stiffness (i.e., higher G′). In contrast, methyl groups in 3,4‐dimethylpyridine *N*‐oxide (**8**) of **(8Ag)Tol** introduce steric hindrance, disrupting gelator–gelator close packing that interferes with the alignment necessary for the fiber formation, leading to softer gels with lower G′. Nevertheless, the denser networks of **(1Ag)Tol** and **(4Ag)Tol** provide greater deformation resistance and mechanical stability. The G′ values of **(1Ag)Tol** and **(4Ag)Tol** exceed 10^3^ Pa, further suggesting the presence of stronger NCIs within these networks.

Time sweep step‐strain tests are performed on **(1Ag)Tol**, **(2Ag)Tol**, **(4Ag)Tol**, and **(8Ag)Tol** to evaluate reversibility and self‐healing at 25 °C. Tests are conducted at 1Hz frequency, alternating between high (600%) and low strain (1%) over seven cycles. As shown in Figure 3C and Figures S53–S55 (Supporting Information), all four gels demonstrate self‐healing behavior. Under high strain, gels collapse, and the G′, which reflects the gel stiffness through energy storage in the crosslinked network, drops to ≈0.1 Pa, and G″ becomes dominant as the network dissipates the applied energy. Upon returning to low strain, G′ rapidly recovers to ≈1000 Pa in all four samples, while G″ decreases, confirming the reformation of the crosslinked gel network. The consistent recovery of mechanical properties over seven cycles highlights self‐healing and reversible gelation. Thermal responsiveness is evaluated via transition temperature tests on **(1Ag)Mes**, (**2Ag)Mes**, **(4Ag)Mes**, and **(8Ag)Mes** (Mes = mesitylene), using a heating rate of 3 °C/min at 1% shear strain and 1Hz frequency. Mesitylene is chosen over toluene due to its higher boiling point (165 °C) and lower volatility (Figure 3D). All four mesitylene gels exhibit upper critical solution temperature^[^ ^41^ ^]^ (UCST)‐like behavior, with G′ decreasing as the temperature rises, indicating the breakdown of fiber networks into monomeric or oligomeric species. **(1Ag)Mes** and **(8Ag)Mes** show a sharp G′ decrease at ≈90 °C and ≈62 °C, respectively, indicating weaker coordination and network stability. In contrast, **(2Ag)Mes** and **(4Ag)Mes** exhibit gradual G′ decrease and retain structural integrity beyond 100 °C. This behavior suggests that the i) crosslinked networks of **(2Ag)Mes** and **(4Ag)Mes** are composed of stronger and intricate coordination polymeric fibers, and ii) PyNOs in **(2Ag)** and **(4Ag)** systems form more stable and thermally resistant networks with AgTFA, compared to those in **(1Ag)** and **(8Ag)**.

### 2.4 X‐Ray Crystallography

Our study, which employs a set of structurally similar PyNOs with systematically varied functional groups, provides an opportunity to quantitatively analyze X‐ray crystal structures and determine the key NCIs involved in gelation. In principle, correlating gelation mechanisms across the 27 PyNO‐AgTFA complexes in eight solvents requires 216 crystal structures. However, only 66 samples yield crystals suitable for X‐ray diffraction, with just 2 from gel‐forming samples (Figures S58–S123, Supporting Information). Attempts to obtain more crystal structures of gel‐forming silver(I) complexes are unsuccessful, largely due to i) inherent disorder in gels, which lack the long‐range periodicity necessary for single‐crystal formation, ii) weak intermolecular forces, which do not support the rigid, repetitive atomic arrangement necessary for crystallinity, and iii) multiple nucleation sites in the gel medium, leading to polycrystalline samples unsuitable for X‐ray studies.

**Figure 4:**
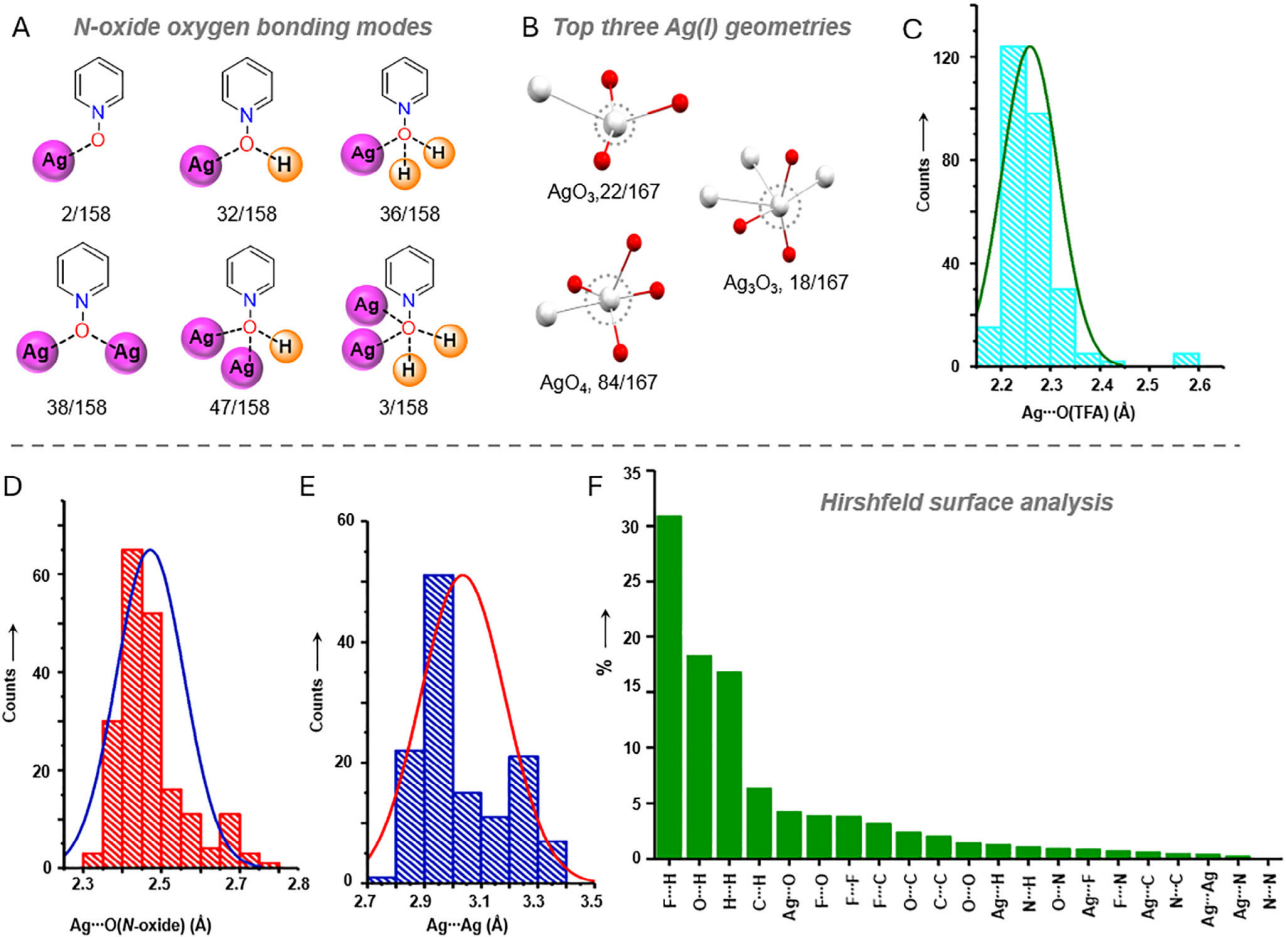
A summary of the bonding modes of *N*‐oxide oxygens, silver(I) geometries and bond distances. A) The *N*‐oxide oxygen bonding modes. B) The top three coordination geometries of silver(I). C–E) Histogram charts showing the frequency of occurrences of Ag···O(TFA), Ag···O(*N*‐oxide), and Ag···Ag distances in 65 silver(I) complexes. F) Hirshfeld surface analysis of PyNO‐AgTFA complexes. Note:, E.g., 2/158 indicates that the monodentate mode is observed 2 times out of the 158 interaction modes. The data in Figure 4 corresponds to 65 silver(I) complexes (sample size, n). The mean value of Ag···O(*N*‐oxide) bond distances is 2.4711 ± 0.0867 Å (number of Ag···O(*N*‐oxide) distances, n = 196, variance = 0.00752), AgO(TFA) bond distances is 2.2585 ± 0.0574 Å (number of Ag···O(TFA) distances, n = 279, variance = 0.00329), and Ag···Ag distances is 3.0353 ± 0.1491 Å (number of Ag−Ag distances, n = 128, variance = 0.02223).

We conducted a statistical analysis of key structural features, including *N*‐oxide oxygen bonding modes, and silver(I) coordination geometries, across a large dataset of crystal structures. Among the 66 crystal structures, there are 28 discrete complexes, 36 polymeric, and one crystallizes only as a ligand. Solvent molecules are present in 29 of the 66 structures; when the solvent is water, in some structures, it is coordinated to silver(I), but in others, it is not. Although groups like ─NO_2_,^[^ ^42^ ^]^ ─CN,^[^ ^43^ ^]^ and aryl^[^ ^44^ ^]^ are known to coordinate with silver(I), PyNO functional groups show no such coordination except for the ─CN group in 4‐cyanopyridine *N*‐oxide (see Figure S124, Supporting Information). The *N*‐oxide oxygen atom, exhibiting *sp* ^2^ and *sp* ^3^ characteristics due to the zwitterionic nature of the N–O bond, acts as a one‐, two‐ and three‐electron donor, enabling polydentate interactions with metals.^[^ ^45^ ^]^ The N–O group bonding modes in PyNO‐AgTFA complexes are summarized in **Figure** **4**A (and Tables S12, Supporting Information). The packing analysis reveals 158 bonding modes. Among these, *N*‐oxide oxygen manifests pure CB monodentate interaction (one Ag···**⁻**O–N^+^ CB) in two of the 158, and μ_2_‐*O*,*O* in 38/158 (two Ag···**⁻**O–N^+^ CBs). The others exhibit hybrid interaction patterns such as μ_2_‐*O*,*O* in 32/158 (one Ag···**⁻**O–N^+^ CB, one H···**⁻**O–N^+^ HB), and μ_3_‐*O*,*O*,*O* in 36/158 (one Ag···**⁻**O–N^+^ CB and two H···**⁻**O–N^+^ HBs). Although *N*‐oxide oxygen can, in principle, coordinate to three silver(I) ions,^[^ ^46^ ^]^ the maximum “pure” CB denticity in PyNO‐AgTFA complexes is limited to two (Figure 4A). This limitation arises from the strong coordination nature of the TFA anion to silver(I) and its innate capability to form sterically hindered paddlewheel structures, which are unsuitable for tridentate silver(I) coordination. The O···H interactions play a critical role in establishing the three‐dimensional gel and crystal networks. However, these *N*‐oxide based CBs and HBs alone do not trigger gelation. Instead, gelation arises from a range of physical crosslinks such as hydrophobic effects and π–π stacking, guided by a donor‐acceptor molecular recognition strategy. These interactions collectively enable PyNO and AgTFA components to self‐assemble into networks capable of entrapping solvent molecules, giving rise to macroscopic gel properties such as viscoelasticity and self‐healing. To investigate these interactions, we applied Hirshfeld Surface (HS) analysis^[^ ^47^ ^]^ to quantify closed intermolecular contacts (Tables S14–S25). Each PyNO‐AgTFA crystal structure exhibits 21 interatomic contacts (Figure 4F). Although it is impossible to determine the specific non‐covalent interactions (NCIs) that singularly drive gelation, HS analysis across multiple structures reveals dominant contributors: F···H interactions account for 30% of the surface. Other interactions involving fluorine atoms include F···O (3.9%), F···F (3.8%), and F···C (3.2%). There are large contributions from O···H (18.3%), O···H (16.8%) and C···H (6.3%) interactions, reflecting the high proportion of hydrogen atoms in the PyNO structure. π‐System related interactions, including F···C (3.2%), C···C (2.4%), and O···C (2.0%), also contribute to the packing. Although Ag‐based interactions account for less than 2% of the surface, they, along with minor contributors (<1%), help minimize global electrostatic repulsions within the gel and crystal system.

The packing analysis reveals that AgTFA consistently forms a paddlewheel structure across all silver(I) complexes, except for one complex, **(11Ag)H_2_O**, which displays both paddlewheel and non‐paddlewheel structures (See Figure S125, Supporting Information). The silver(I) complexes showcase a total of eleven distinct silver(I) geometries. The three most common are: 84 five‐coordinate silver(I) in an AgO_4_ environment, 22 four‐coordinate silver(I) in an AgO_3_ environment, and 18 six‐coordinate silver(I) in an Ag_3_O_3_ environment (See Figure 4B and Table S13, Supporting Information). Given the frequency of these geometrical preferences in the crystalline state, it is likely that silver(I) geometries in non‐crystalline gels adopt similar geometries, with a strong tendency towards one of the top three. All observed Ag···O distances fall within the expected range for silver(I) complexes.^[^ ^28^ ^]^ The Ag···O(TFA) distances are significantly shorter than the Ag···O(*N*‐oxide), indicating that TFA anion oxygens exhibit a stronger coordination ability compared to *N*‐oxide oxygen.

**Figure 5:**
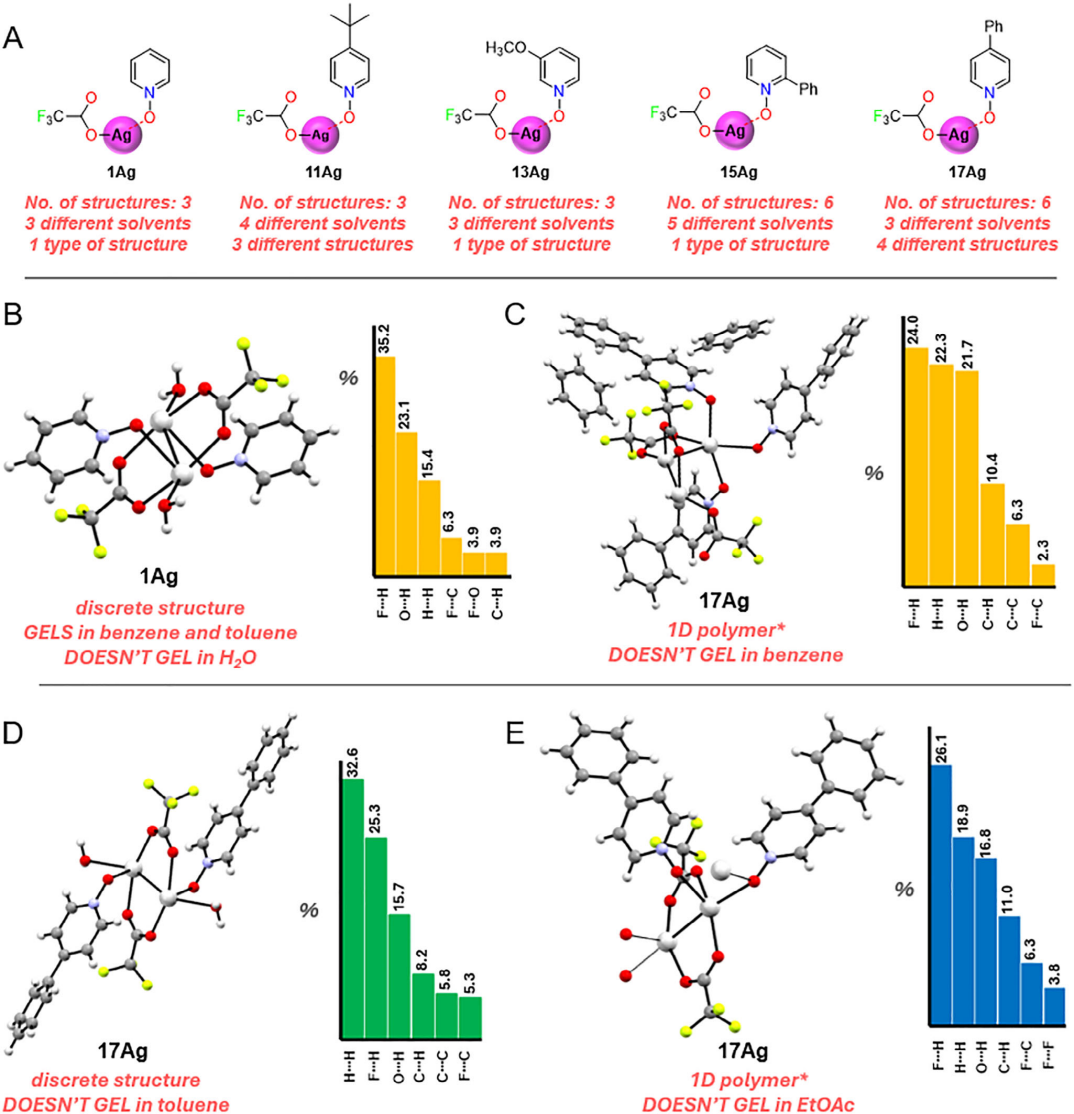
A summary of selected X‐ray crystallography data and their Hirshfeld surface analysis. A) A selected list of PyNO‐AgTFA complexes showing the number of structures obtained from different solvents. The chemical structures of silver(I) complexes are shown in a simple form for viewing clarity. The Hirsfeld surface analysis histogram charts showing the top NCIs for B) the pyridine *N*‐oxide‐AgTFA (**1Ag**) crystal structures in benzene, toluene, and water; and C–E) the 4‐phenylpyridine *N*‐oxide‐AgTFA (**17Ag**) crystal structures in benzene, toluene, and EtOAc. *Note: The crystal structures of **17Ag** in benzene and EtOAc are polymeric, and only partial structures were shown for viewing clarity.

Analysis of 66 crystal structures reveals a key trend: PyNOs complexed with AgTFA consistently form no more than four different silver(I) structures, regardless of the ligand:silver(I) ratio, or the solvent employed (**Figure** **5**A, and Figures S56 and S57, Supporting Information). This highlights the limited structural variability of PyNO‐AgTFA mixtures to form a “narrow range” of silver(I) complexes. For instance, the pyridine *N*‐oxide‐AgTFA (**1Ag**) crystallizes in a single structural form across benzene, toluene, and H_2_O (Figure 5B). In contrast, the 4‐phenylpyridine *N*‐oxide‐AgTFA (**17Ag**) demonstrates a greater structural diversity. In benzene, **17Ag** forms a 1D polymer incorporating benzene molecules, in toluene, it yields a discrete complex with H_2_O coordinated to silver(I), and in EtOAc, it forms a polymer (Figure 5C–E). The Hirshfeld surface analysis^[^ ^47^ ^]^ further supports this difference: **(1Ag)Ben/Tol/H_2_O** show identical NCI percentages, while **(17Ag)Ben/Tol/EtOAc** exhibit varied NCI percentages. Notably, **1Ag** forms gels in benzene and toluene but not in H_2_O, despite maintaining the same discrete structure. This suggests that gelation in benzene and toluene arises from intermolecular hydrogen‐bonded networks between discrete silver(I) complexes, effectively immobilizing the solvent. In water, such a network structure is likely disrupted. The coordinated water molecule in **1Ag** complexes may result from residual moisture in benzene or toluene or infiltration during the crystallization process. It is also plausible that the gel‐forming network forms a water‐free silver(I) complex distinct from the crystallized structural form. The crystal structure of **(1Ag)Tol** closely resembles **(17Ag)Tol**, differing mainly from the *para*‐phenyl group in **17Ag**, which correlates with loss of gelation. This underscores the crucial role of substitution effects in modulating gelation behaviour.

**Figure 6:**
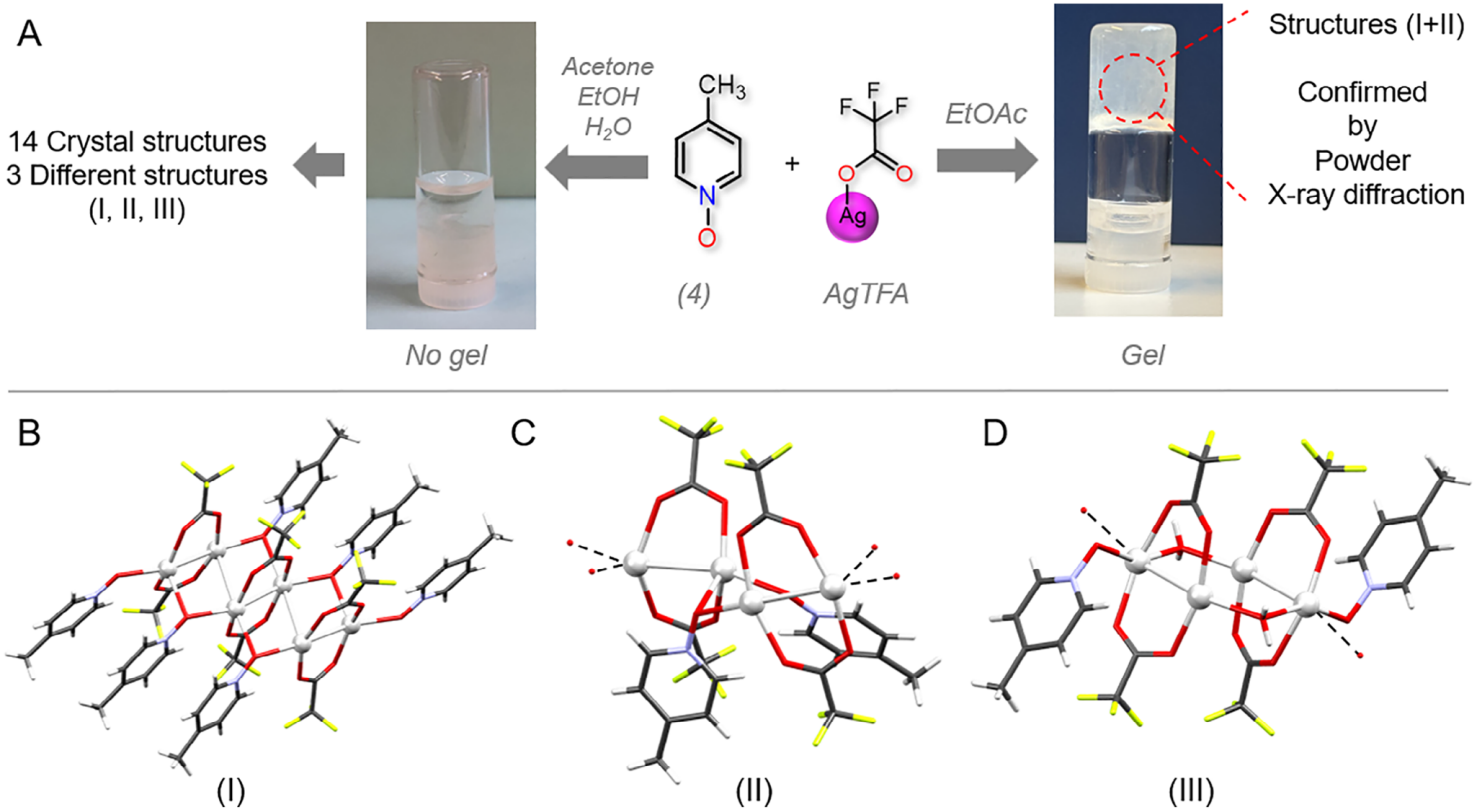
Powder X‐ray diffraction analysis. A) An overview of gelation experiments of **4Ag** in acetone, EtOH, H_2_O, and EtOAc. B,C) Three recurring structures of **4Ag** in acetone, EtOH, and H_2_O. The crystal structures II and III are polymeric, and only partial structures are shown for viewing clarity.

The 4‐methylpyridine *N*‐oxide‐TFA (**4Ag**) gel samples derived from EtOAc are not suitable for XRD analysis (Table 1). However, **4Ag** complexes synthesized in acetone, EtOH and H_2_O under various ligand‐to‐metal ratios yield 14 X‐ray crystal structures, corresponding to three different types of structures, one discrete and two polymeric structures (I‐III, **Figure** **6**). To determine the structure of (**4Ag)EtOAc** prepared at a 1:1 PyNO:AgTFA ratio, powder X‐ray diffraction (PXRD) analysis is performed on the dried gel and compared to simulated PXRD spectra of three known silver(I) complexes (Figures S126–S130, Supporting Information). The (**4Ag)EtOAc** sample contains phases of discrete (type I) and polymer (type II) silver(I) complexes, with the polymeric phase as the dominant component. The formation of mixed phases in gels arises because gelation occurs under non‐equilibrium conditions. As the PyNO and AgTFA components assemble, different discrete and polymeric silver(I) complexes can nucleate and grow simultaneously without reorganizing into a single thermodynamically favorable phase. Additionally, the flexible coordination behavior of silver(I) ions and variations in gelator–gelator and solvent–gelator interactions can promote the formation of multiple PyNO‐AgTFA complexes during gelation, leading to phase heterogeneity of silver(I) complexes.

### 2.5 Computational Studies

**Figure 7:**
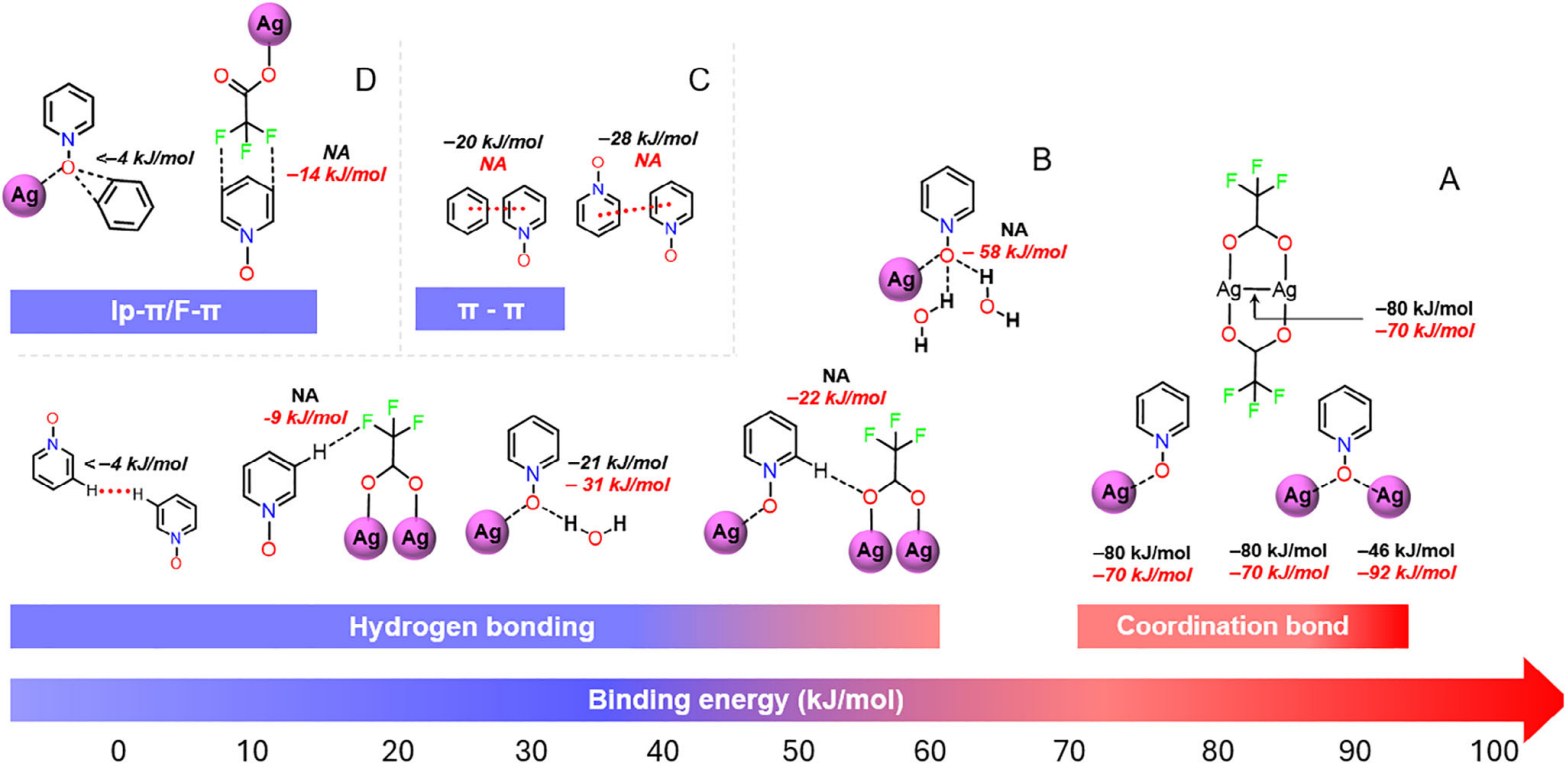
Binding energy map of coordination bonds and various physical crosslinks. Types of non‐covalent interactions and their interaction energies estimated using Density functional theory PBE0‐D3/def2‐TZVP at level of theory. The black font represents energies derived from gas phase optimized structures and red font correspond to those derived from X‐ray crystal structure geometries.

The interaction energies (ΔE_int_) of common NCI motifs observed in crystal structures are estimated using DFT at the PBE0‐D3/def2‐TZVP^[^ ^48^ ^]^ level of theory. Structures corresponding to the strongest NCIs are optimized and ranked as shown in **Figure** **7**. However, not all observed NCIs can be optimized successfully, as stronger interactions often dominate and override weaker interactions during geometry optimizations. Therefore, ΔE_int_ values for weaker interactions are calculated directly from crystal structure geometries. It is important to note that DFT energy partitioning typically treats isolated pairwise interactions and does not fully capture cooperative or synergistic effects between silver(I) and PyNO coordination, and auxiliary interactions such as HB and π–π stacking. In PyNO‐AgTFA metallogels, such cooperative effects play a crucial role in extended network stabilization. However, these interactions are not accounted for in the calculations due to computational complexity and fall beyond the scope of this study. Prior DFT studies have specifically addressed these cooperative interactions in gelation.^[^ ^49^ ^]^ The Ag–Ag interaction energy within the paddlewheel motif is estimated using the quantum theory of atoms in molecules (QTAIM) and the Frontera et al.^[^ ^50^ ^]^ equation based on electron density at bond critical points (ρ_BCP_). The Ag–Ag interaction energy is −68 kJ mol^−1^ (ρ_BCP_ 0.032 a.u.) for the optimized structure and −40 kJ mol^−1^ (ρ_BCP_ 0.024 a.u.) for the crystal structure, reflecting weakening likely due to competition from PyNOs and H_2_O coordination at silver(I) centers. This argentophilic interaction strength is comparable to reported systems, e.g., –50 kJ mol^−1^ in [Ag(μ‐Ph_2_PCH_2_PPh_2_NSiMe_3_)]_2_[SbF_6_]_2_.^[^ ^51^ ^]^

The optimized ΔE_int_ for the Ag···O–N CB of a monodentate *N*‐oxide is −80 kJ mol^−1^, while the second Ag···O–N CB in a μ_2_‐*O*,*O N*‐oxide contributes an additional –46 kJ mol^−1^, consistent with a weakening trend for cumulative binding (Figure 7A). However, ΔE_int_ energies for Ag···O–N CBs calculated from crystal structure geometries suggest a possible cooperative effect in the solid state. The second CB contributes −92 kJ mol^−1^, while the monodentate CB contributes −70 kJ mol^−1^, indicating enhanced interaction in the μ_2_‐*O*,*O* motif. In the optimized μ_2_‐*O*,*O N*‐oxide motif, which features one Ag···O–N CB and one H···O–N HB, the ΔE_int_ of the HB is −21 kJ mol^−1^, significantly weaker than the Ag···O–N CB (Figure 7B). In the μ₃‐*O,O,O* N‐oxide motif containing one Ag···O–N CB and two (HO)H···O–N HBs (Figure 7B), optimization is not feasible. As a result, HB interaction energies are derived from crystal structure geometry. The total Δ*E*_int_ of two HBs is −58 kJ mol⁻¹, comparable to that of a single Ag···O–N CB. This suggests that an excess of strong HB donors can compete with PyNO–AgTFA coordination, potentially disrupting network formation. The *ortho*‐proton of PyNO, being relatively acidic, forms C─H···O(AgTFA) HBs with ΔE_int_ of −22 kJ mol⁻¹, comparable to that of (HO)H···O─N HBs (−31 kJ mol⁻¹). Short H···F contacts between PyNO aromatic protons and CF_3_ groups of AgTFA also contribute to packing, with ΔE_int_ values around −9 kJ mol⁻¹ (e.g., Figure 7B). The (PyNO)H···F(AgTFA) and (PyNO)C···F(AgTFA) interactions are energetically similar. In the **(17Ag)Ben** structure, π···O–N short contacts are observed between benzene and N–O groups, but ΔE_int_ calculations indicate these interactions arise from close molecular packing rather than favorable NCIs. Comparing π–π interactions (Figure 7C,D), (PyNO)π···π(PyNO) interactions (−28 kJ mol⁻¹) are stronger than (ben)π···π(PyNO) interactions (−20 kJ mol⁻¹).

## 3 Conclusions

We systematically investigated the fundamental differences in gelation behavior among a series of pyridine *N*‐oxide‐silver(I) trifluoroacetate (PyNO‐AgTFA) complexes by varying both the substituents on the pyridine N‐oxides and the solvents used. Key findings include that pyridine *N*‐oxides bearing electron‐donating groups readily form gels, whereas those containing only electron‐withdrawing groups, or a combination of electron‐donating and withdrawing groups, generally do not. However, the absence of gelation cannot be attributed solely to the presence of electron‐withdrawing substituents; solvent polarity and hydrogen bonding (HB) capabilities also critically influence gelation outcomes. Among the solvents tested, benzene, toluene, ethyl acetate, and acetone supported gelation, while ethanol, DMF, DMSO, and water did not. The highly polar nature of the latter solvents likely disrupts both gelator–gelator and solvent–gelator interactions, preventing gel network formation. While gelation in aqueous environments would be highly desirable for biomedical applications, the current PyNO‐AgTFA systems fail to form gels in water, limiting their direct use in hydrogel‐based technologies. The *N*‐oxide functional group is essential for gelation. Control experiments using pyridine‐AgTFA complexes lacking the *N*‐oxide group, otherwise chemically similar, confirmed that gelation does not occur without the *N*‐oxide. This underscores the critical role of the *N*‐oxide oxygen in enabling the formation of silver(I) coordination networks that immobilize solvent molecules. Despite the inherent complexity of rational gelator design, owing to the interplay of molecular, supramolecular, and macroscopic factors, our findings reveal that the *N*‐oxide‐silver(I) interactions uniquely promote the formation of out‐of‐equilibrium supramolecular gels. In contrast, their unoxidized pyridine counterparts fail to form such dynamic systems. These experimental insights lay strong groundwork for expanding our research into related areas, including the development of hydrogels based on biocompatible metals and nanoparticle‐infused gels for wound dressing applications. The PyNO‐AgTFA two‐component systems consistently yield self‐healing, fibrous gels with excellent mechanical strength, enabling the fabrication of free‐standing gel objects with precise shape control at the centimeter scale. This mechanical robustness stems from the dense physical crosslinking facilitated by the N‐oxide oxygen within the silver(I) coordination networks, further stabilized by non‐covalent interactions, including hydrogen bonding (e.g., H···H contacts) and fluorine‐based contacts (e.g., H···F interactions), which reinforce the gel matrix. However, these PyNO‐AgTFA gels, while strong, do not yet exhibit the mechanical toughness characteristic of supramolecular elastomers. Improving their mechanical properties (e.g., stiffness and elasticity) is a major future research goal, with applications envisioned in electrospinning, smart film materials, and other advanced technologies.

## 4 Experimental Section

Pyridines, pyridine *N*‐oxides (**1**‐**9**, **14**, **17**, **20**, **21**, and **26**–**27**), silver(I) salts and HPLC grade solvents (>99%) were commercially purchased and used without further purification. Pyridine *N*‐oxides (**10**‐**13**, **15**, **16**, **18**, **19**, **22**–**25**) were synthesized using established H_2_O_2_/AcOH reaction conditions.^[^ ^52^ ^]^ Single crystal data were collected using either a Rigaku SuperNova four‐circle diffractometer equipped with a Hybrid Pixel Array Detector (detector type: HyPix‐Arc 100, Cu‐K_α_ (λ = 1.54184 Å) radiation] or XtaLAB Synergy‐R four‐circle diffractometer equipped with a Hybrid Pixel Array Detector [detector type: HyPix‐Arc 100; Diffraction source type was PhotonJet R (Cu, λ = 1.54184 Å) X‐ray source]. Absorption corrections were applied using a Gaussian/analytical face index method. All structures were solved by intrinsic phasing (SHELXT)^[^ ^53^ ^]^ and refined by full‐matrix least squares on *F* ^2^ using the OLEX2^[^ ^54^ ^]^ with the SHELXL‐2015 module.^[^ ^55^ ^]^ Anisotropic displacement parameters were assigned to non‐H atoms and isotropic displacement parameters for H atoms were constrained to 1.2 times the equivalent displacement parameters of their parent atoms. Powder X‐ray diffraction measurements were performed on a PANalytical X'Pert PRO diffractometer using Cu K_α_ radiation (λ = 1.5418 Å; 45 kV, 40 mA). Samples were prepared on a zero‐background Si‐plate using petrolatum jelly as an adhesive. Diffraction data were recorded using an X'Celerator detector over a 2θ range of 3–60°, with a step size of 0.017° and a counting time of 60 s per step. The instrument alignment was confirmed with a Si powder standard (SRM 640, National Institute of Standards & Technology). Data processing and Pawley whole‐pattern fitting were performed using X'pert HighScore Plus (v. 4.9), with refinement based on single‐crystal unit cell parameters and variables including zero‐offset, polynomial background, sample displacement, unit cell parameters and peak profile parameters, such as peak width, shape, and asymmetry. Rheological characterization was performed using a Malvern Kinexus Prime Lab^+^. Metallogel samples were measured in triplicate with an 8 mm parallel plate at 25 °C under ≈0.05 N force, using oscillatory amplitude sweep, frequency sweep (at 0.1% shear strain) and shear viscosity (with a 2 min ramp) tests. All DFT optimizations were carried out using the Gaussian 16 program package^[^ ^56^ ^]^ with the PBE0 hybrid functional,^[^ ^48a,b,d^ ^]^ def2‐TZVP basis sets,^[^ ^48f,g^ ^]^ and Grimme's empirical D3BJ dispersion correction^[^ ^48d,e^ ^]^ for treating dispersion forces. Quantum theory of atoms‐in‐molecules (QTAIM)^[^ ^57^ ^]^ analyses were carried out using the MultiWFN program.^[^ ^58^ ^]^

### Crystallography Data

CCDC 2420927–2420946, 2420950–2420969, and 2420974–2420999 contain the supplementary crystallographic data for this paper. These data can be obtained free of charge from The Cambridge Crystallographic Data Centre via www.ccdc.cam.ac.uk/data_request/cif. These data can be obtained free of charge via www.ccdc.cam.ac.uk/data_request/cif, or by emailing data_request@ccdc.cam.ac.uk, by contacting The Cambridge Crystallographic Data Centre, 12 Union Road, Cambridge CB2 1EZ, UK; fax: +44 1223 336 033.

### Statistical Analysis

Olex 2‐1.5 and Mercury 2024.2.0 were used to visualize structures and extract bond parameters from X‐ray crystal structures. OriginPro 2017 (b9.4.1.354) was used for data analysis and to prepare histogram charts shown in Figure 4. The Figure 4 presents Ag−O(*N*‐oxide), Ag−O(TFA) and Ag−Ag distances corresponding to 65 silver(I) complexes (sample size, n), while Figure 5 shows Hirshfeld surface analysis of six structures. Hirshfeld surfaces analysis was performed using CrystalExplorer. All Figures were prepared using Microsoft PowerPoint. The mean value of Ag−O(*N*‐oxide) bond distances was 2.4711 ± 0.0867 Å [number of Ag−O(*N*‐oxide) distances, n = 196, variance = 0.00752], Ag−O(TFA) bond distances was 2.2585 ± 0.0574 Å [number of Ag−O(TFA) distances, n = 279, variance = 0.00329], N−O−Ag angle was 117.928±9.499° Å [number of N−O−Ag angles, n = 195, variance = 90.2287], and Ag−Ag distance was 3.0353 ± 0.1491 Å [number of Ag−Ag distances, n = 128, variance = 0.02223].

## Conflict of Interest

The authors declare no conflict of interests.

## Author Contributions

R.P. is responsible for project conception, design, gel experiments, crystallizations, X‐ray crystallography, rheology data processing, preparation of free‐standing gels and manuscript preparation. B.T. carried out the rheology measurements, J.M.R. carried out the computational studies, and E.K. 3D printed the polypropylene moulds. M.L. carried out the PXRD and DSC measurements. R.P. prepared gels for rheology studies in R.H.'s lab. J.L. and K.R. funded the R.P.’s research visit to the R.H. lab. All authors read and approved the manuscript.

## Supporting information



Supporting Information

Supporting Data

## Data Availability

The data that support the findings of this study are available in the supplementary material of this article.
