# Supplementary material for: Free‐Standing Supramolecular Pyridine N‐Oxide‐Silver(I) Metallogels
Source: Adv Mater. 2025 Jul 1;37(37):2502818. doi: 10.1002/adma.202502818 (PMC12447065; doi:10.1002/adma.202502818)
Supplement: Supplementary file 1 — Supporting Information [file ADMA-37-2502818-s002.docx]

Supporting Information

Free-Standing Supramolecular Pyridine *N*-oxide-Silver(I) Metallogels

Rakesh Puttreddy,*^A^ Boonya Thongrom,^B^ J. Mikko Rautiainen,^A^ Manu Lahitnen,^A^ Esa Kukkonen,^A^ Rainer Haag,^B^ Jani O. Moilanen,^A^ Jan Lundell,^A^ Kari Rissanen*^A^

**Table of Contents**

**1. General structural information *Page 2***

**2. Gelation studies  *Page 4***

**3. Scanning electron microscopy (SEM) *Page 14***

**4. Rheology  *Page 56***

**5. X-ray Crystallography *Page 62***

**6. Hirshfeld surface analysis *Page 100***

**7. Powder X-ray diffraction (PXRD) *Page 112***

**8. DFT xyz coordinates files *Page 122***

**1. General information**

**Figure S1.** List of pyridine N-oxides (**1** - **27**) used to prepare silver(I) complexes.

**1.1 General procedure and tips for the preparation of gels**

PyNO (1.0 eq) is dissolved in 0.5 mL of solvent, and AgTFA (1.0 eq) is dissolved in a separate 0.5 mL of the same solvent. The two solutions are then mixed, resulting in gel formation. Although PyNOs appear as clear solutions in non-polar solvents such as benzene and toluene, they are not completely homogeneous due to the polar nature of PyNOs. Therefore, when benzene or toluene is used, the PyNO solution is heated to 80 °C on a hot plate or using a heat gun to ensure complete dissolution and then brought to room temperature before mixing with the AgTFA solution. Small substituent changes lead to significant differences in solubility. For instance, N-oxides such as **21-23** are crystalline materials that require heating in benzene or toluene to dissolve fully. In contrast, N-oxides such as **24** and **25** dissolve readily in the same solvents without the need for heating.

**1.2 Preparation of moulds**

**a) Short-rod mould**: Silver(I) trifluoroacetate (20.6 mg, 0.0934 mmol) dissolved in 1.7 mL of toluene and ligand **19** (13.0 mg, 0. 0934 mmol) was dissolved in 1.7 mL of toluene. Both solutions are heated to 50 °C using heat gun. The solutions were mixed and immediately poured into the 5 mL syringe and allowed to slowly cool at room temperature. One side of the syringe was cut using blade and using piston the mould was pushed out of the syringe.

**b) Long-rod mould**: Silver(I) trifluoroacetate (44.5 mg, 0.2012 mmol) dissolved in 2.5 mL of toluene and ligand **19** (28.0 mg, 0.20122 mmol) was dissolved in 2.5 mL of toluene. Both solutions are heated to 50 °C with heat gun. After mixing the solutions, they were promptly poured into a 5 mL syringe (one end closed) and let to gradually cool at room temperature. One side of the syringe was sliced using a blade, and the mould was forced out using a plastic piston.

**c) Square mould** Silver(I) trifluoroacetate (60.4 mg, 0.2734 mmol) dissolved in 2.6 mL of toluene and ligand **1** (26.0 mg, 0.20122 mmol) was dissolved in 2.6 mL of toluene. Both solutions are heated to 50 °C with heat gun. After mixing the solutions, they were promptly poured into a 3D printed polypropylene square mould and let to gradually cool at room temperature. Using spatula, the gel mould was removed.

**d) Horseshoe mould** Silver(I) trifluoroacetate (57.4 mg, 0.2598 mmol) dissolved in 2.8 mL of toluene and ligand **8** (32.0 mg, 0.2598 mmol) was dissolved in 2.8 mL of toluene. Both solutions are heated to 50 °C with heat gun. After mixing the solutions, they were promptly poured into a 3D printed polypropylene horseshoe mould and let to gradually cool at room temperature. Using spatula, the gel mould was removed.

**e) O-Shape mould** Silver(I) trifluoroacetate (60.7 mg, 0.2598 mmol) dissolved in 2.5 mL of toluene and ligand **2** (30.0 mg, 0.2598 mmol) was dissolved in 2.5 mL of toluene. Both solutions are heated to 50 °C with heat gun. After mixing the solutions, they were promptly poured into a 3D printed polypropylene O-shape mould and let to gradually cool at room temperature. Using spatula, the gel mould was removed.

**2. Gelation studies**

**Table S1.** Gelation experiments using 0.25 w/v% pyridine N-oxides

| **PyNO**  **(0.25w/v%)** | **PyNO:AgTFA ratio** | **Benzene**  **(1.0 mL)** | **Toluene**  **(1.0 mL)** | **EtOAc**  **(1.0 mL)** | **Acetone**  **(1.0 mL)** |
| --- | --- | --- | --- | --- | --- |
| **1** | 1:1 | gel | gel | gel | No gel |
| **2** | 1:1 | gel | gel | gel | No gel |
| **3** | 1:1 | No gel | gel | No gel | No gel |
| **4** | 1:1 | gel | gel | gel | No gel |
| **5** | 1:1 | gel | gel | No gel | No gel |
| **6** | 1:1 | gel | gel | gel | No gel |
| **7** | 1:1 | No gel | No gel | No gel | No gel |
| **8** | 1:1 | gel | gel | gel | gel |
| **9** | 1:1 | No gel | No gel | No gel | No gel |
| **10** | 1:1 | No gel | gel | No gel | No gel |
| **11** | 1:1 | No gel | No gel | No gel | No gel |
| **12** | 1:1 | gel | No gel | gel | No gel |
| **13** | 1:1 | No gel | No gel | No gel | No gel |
| **14** | 1:1 | No gel | No gel | No gel | No gel |
| **15** | 1:1 | No gel | No gel | No gel | No gel |
| **16** | 1:1 | No gel | No gel | No gel | No gel |
| **17** | 1:1 | No gel | No gel | No gel | No gel |
| **18** | 1:1 | No gel | No gel | No gel | No gel |
| **19** | 1:1 | gel | gel | gel | No gel |
| **20** | 1:1 | No gel | No gel | No gel | No gel |
| **21** | 1:1 | No gel | No gel | No gel | No gel |
| **22** | 1:1 | No gel | No gel | No gel | No gel |
| **23** | 1:1 | No gel | No gel | No gel | No gel |
| **24** | 1:1 | No gel | No gel | No gel | No gel |
| **25** | 1:1 | No gel | No gel | No gel | No gel |
| **26** | 1:1 | No gel | No gel | No gel | No gel |
| **27** | 1:1 | gel | gel | gel | No gel |

**Table S2.** Gelation experiments using 0.25 w/v% pyridine N-oxides

| **PyNO**  **(0.25w/v%)** | **PyNO:AgTFA ratio** | **DMSO**  **(1.0 mL)** | **DMF**  **(1.0 mL)** | **EtOH**  **(1.0 mL)** | **H_2_O**  **(1.0 mL)** |
| --- | --- | --- | --- | --- | --- |
| **1** | 1:1 | No gel | No gel | No gel | No gel |
| **2** | 1:1 | No gel | No gel | No gel | No gel |
| **3** | 1:1 | No gel | No gel | No gel | No gel |
| **4** | 1:1 | No gel | No gel | No gel | No gel |
| **5** | 1:1 | No gel | No gel | No gel | No gel |
| **6** | 1:1 | No gel | No gel | No gel | No gel |
| **7** | 1:1 | No gel | No gel | No gel | No gel |
| **8** | 1:1 | No gel | No gel | No gel | No gel |
| **9** | 1:1 | No gel | No gel | No gel | No gel |
| **10** | 1:1 | No gel | No gel | No gel | No gel |
| **11** | 1:1 | No gel | No gel | No gel | No gel |
| **12** | 1:1 | No gel | No gel | No gel | No gel |
| **13** | 1:1 | No gel | No gel | No gel | No gel |
| **14** | 1:1 | No gel | No gel | No gel | No gel |
| **15** | 1:1 | No gel | No gel | No gel | No gel |
| **16** | 1:1 | No gel | No gel | No gel | No gel |
| **17** | 1:1 | No gel | No gel | No gel | No gel |
| **18** | 1:1 | No gel | No gel | No gel | No gel |
| **19** | 1:1 | No gel | No gel | No gel | No gel |
| **20** | 1:1 | No gel | No gel | No gel | No gel |
| **21** | 1:1 | No gel | No gel | No gel | No gel |
| **22** | 1:1 | No gel | No gel | No gel | No gel |
| **23** | 1:1 | No gel | No gel | No gel | No gel |
| **24** | 1:1 | No gel | No gel | No gel | No gel |
| **25** | 1:1 | No gel | No gel | No gel | No gel |
| **26** | 1:1 | No gel | No gel | No gel | No gel |
| **27** | 1:1 | No gel | No gel | No gel | No gel |

**Table S3.** Gelation experiments using 0.5 w/v% pyridine N-oxides

| **PyNO**  **(0.5w/v%)** | **PyNO:AgTFA ratio** | **Benzene**  **(1.0 mL)** | **Toluene**  **(1.0 mL)** | **EtOAc**  **(1.0 mL)** | **Acetone**  **(1.0 mL)** |
| --- | --- | --- | --- | --- | --- |
| **1** | 1:1 | gel | gel | gel | No gel |
| **2** | 1:1 | gel | gel | gel | No gel |
| **3** | 1:1 | gel | gel | No gel | No gel |
| **4** | 1:1 | gel | gel | gel | No gel |
| **5** | 1:1 | gel | gel | No gel | No gel |
| **6** | 1:1 | gel | gel | gel | gel |
| **7** | 1:1 | No gel | No gel | No gel | No gel |
| **8** | 1:1 | gel | gel | gel | gel |
| **9** | 1:1 | No gel | No gel | No gel | No gel |
| **10** | 1:1 | gel | gel | gel | No gel |
| **11** | 1:1 | No gel | gel | No gel | No gel |
| **12** | 1:1 | gel | gel | gel | gel |
| **13** | 1:1 | No gel | No gel | No gel | No gel |
| **14** | 1:1 | No gel | No gel | No gel | No gel |
| **15** | 1:1 | No gel | No gel | No gel | No gel |
| **16** | 1:1 | No gel | No gel | No gel | No gel |
| **17** | 1:1 | No gel | No gel | No gel | No gel |
| **18** | 1:1 | No gel | No gel | No gel | No gel |
| **19** | 1:1 | gel | gel | gel | No gel |
| **20** | 1:1 | No gel | No gel | No gel | No gel |
| **21** | 1:1 | No gel | No gel | No gel | No gel |
| **22** | 1:1 | No gel | No gel | No gel | No gel |
| **23** | 1:1 | No gel | No gel | No gel | No gel |
| **24** | 1:1 | No gel | No gel | No gel | No gel |
| **25** | 1:1 | No gel | No gel | No gel | No gel |
| **26** | 1:1 | No gel | No gel | No gel | No gel |
| **27** | 1:1 | gel | gel | gel | gel |

**Table S4.** Gelation experiments using 0.5 w/v% pyridine N-oxides

| **PyNO**  **(0.5w/v%)** | **PyNO:AgTFA ratio** | **DMSO**  **(1.0 mL)** | **DMF**  **(1.0 mL)** | **EtOH**  **(1.0 mL)** | **H_2_O**  **(1.0 mL)** |
| --- | --- | --- | --- | --- | --- |
| **1** | 1:1 | No gel | No gel | No gel | No gel |
| **2** | 1:1 | No gel | No gel | No gel | No gel |
| **3** | 1:1 | No gel | No gel | No gel | No gel |
| **4** | 1:1 | No gel | No gel | No gel | No gel |
| **5** | 1:1 | No gel | No gel | No gel | No gel |
| **6** | 1:1 | No gel | No gel | No gel | No gel |
| **7** | 1:1 | No gel | No gel | No gel | No gel |
| **8** | 1:1 | No gel | No gel | No gel | No gel |
| **9** | 1:1 | No gel | No gel | No gel | No gel |
| **10** | 1:1 | No gel | No gel | No gel | No gel |
| **11** | 1:1 | No gel | No gel | No gel | No gel |
| **12** | 1:1 | No gel | No gel | No gel | No gel |
| **13** | 1:1 | No gel | No gel | No gel | No gel |
| **14** | 1:1 | No gel | No gel | No gel | No gel |
| **15** | 1:1 | No gel | No gel | No gel | No gel |
| **16** | 1:1 | No gel | No gel | No gel | No gel |
| **17** | 1:1 | No gel | No gel | No gel | No gel |
| **18** | 1:1 | No gel | No gel | No gel | No gel |
| **19** | 1:1 | No gel | No gel | No gel | No gel |
| **20** | 1:1 | No gel | No gel | No gel | No gel |
| **21** | 1:1 | No gel | No gel | No gel | No gel |
| **22** | 1:1 | No gel | No gel | No gel | No gel |
| **23** | 1:1 | No gel | No gel | No gel | No gel |
| **24** | 1:1 | No gel | No gel | No gel | No gel |
| **25** | 1:1 | No gel | No gel | No gel | No gel |
| **26** | 1:1 | No gel | No gel | No gel | No gel |
| **27** | 1:1 | No gel | No gel | No gel | No gel |

**Table S5.** Gelation experiments using 0.25 w/v% pyridines

| **Pyridines** | **Py:AgTFA** | **Py:AgTFA**  **w/v% ratio** | **Benzene**  **(1.0 mL)** | **Toluene**  **(1.0 mL)** | **EtOAc**  **(1.0 mL)** | **Acetone**  **(1.0 mL)** |
| --- | --- | --- | --- | --- | --- | --- |
| **Pyridine** | 1:1 | 0.25:0.70 | No gel | No gel | No gel | No gel |
| **2-methylpyridine** | 1:1 | 0.25:0.70 | No gel | No gel | No gel | No gel |
| **3-methylpyridine** | 1:1 | 0.25:0.60 | No gel | No gel | No gel | No gel |
| **4-methylpyridine** | 1:1 | 0.25:0.60 | No gel | No gel | No gel | No gel |
| **2,4-dimethylpyridine** | 1:1 | 0.25:0.6 | No gel | No gel | No gel | No gel |
| **2,5-dimethylpyridine** | 1:1 | 0.25:0.60 | No gel | No gel | No gel | No gel |
| **2,6-dimethylpyridine** | 1:1 | 0.25:0.51 | No gel | No gel | No gel | No gel |
| **2,5-dimethylpyridine** | 1:1 | 0.25:0.51 | No gel | No gel | No gel | No gel |
| **2,4,6-trimethylpyridine** | 1:1 | 0.25:0.45 | No gel | No gel | No gel | No gel |
| **4-Ethylpyridine** | 1:1 | 0.25: 0.51 | No gel | No gel | No gel | No gel |
| **4-tert-Butylpyridine** | 1:1 | 0.25: 0.51 | No gel | No gel | No gel | No gel |
| **2-Methoypyridine** | 1:1 | 0.25:0.51 | No gel | No gel | No gel | No gel |
| **3-Methoypyridine** | 1:1 | 0.25:0.51 | No gel | No gel | No gel | No gel |
| **4-Methoxypyridine** | 1:1 | 0.25:0.51 | No gel | No gel | No gel | No gel |
| **2-Phenylpyridine** | 1:1 | 0.25:0.36 | No gel | No gel | No gel | No gel |
| **3-Phenylpyridine** | 1:1 | 0.25:0.36 | No gel | No gel | No gel | No gel |
| **4-Phenylpyridine** | 1:1 | 0.25:0.36 | No gel | No gel | No gel | No gel |
| **2-Benzylpyridine** | 1:1 | 0.25:0.32 | No gel | No gel | No gel | No gel |
| **2-methyl-4-methoxypyridine** | 1:1 | 0.25:0.40 | No gel | No gel | No gel | No gel |
| **Quinoline** | 1:1 | 0.25:0.43 | No gel | No gel | No gel | No gel |
| **Isoquinoline** | 1:1 | 0.25:0.43 | No gel | No gel | No gel | No gel |

**Table S6.** Gelation experiments using 0.25 w/v% pyridines

| **Pyridines** | **Py:AgTFA** | **Py:AgTFA**  **w/v% ratio** | **EtOH**  **(1.0 mL)** | **DMF**  **(1.0 mL)** | **DMSO**  **(1.0 mL)** | **H2O**  **(1.0 mL)** |
| --- | --- | --- | --- | --- | --- | --- |
| **Pyridine** | 1:1 | 0.25:0.70 | No gel | No gel | No gel | No gel |
| **2-methylpyridine** | 1:1 | 0.25:0.70 | No gel | No gel | No gel | No gel |
| **3-methylpyridine** | 1:1 | 0.25:0.60 | No gel | No gel | No gel | No gel |
| **4-methylpyridine** | 1:1 | 0.25:0.60 | No gel | No gel | No gel | No gel |
| **2,4-dimethylpyridine** | 1:1 | 0.25:0.6 | No gel | No gel | No gel | No gel |
| **2,5-dimethylpyridine** | 1:1 | 0.25:0.60 | No gel | No gel | No gel | No gel |
| **2,6-dimethylpyridine** | 1:1 | 0.25:0.51 | No gel | No gel | No gel | No gel |
| **2,5-dimethylpyridine** | 1:1 | 0.25:0.51 | No gel | No gel | No gel | No gel |
| **2,4,6-trimethylpyridine** | 1:1 | 0.25:0.45 | No gel | No gel | No gel | No gel |
| **4-Ethylpyridine** | 1:1 | 0.25: 0.51 | No gel | No gel | No gel | No gel |
| **4-tert-Butylpyridine** | 1:1 | 0.25: 0.51 | No gel | No gel | No gel | No gel |
| **2-Methoypyridine** | 1:1 | 0.25:0.51 | No gel | No gel | No gel | No gel |
| **3-Methoypyridine** | 1:1 | 0.25:0.51 | No gel | No gel | No gel | No gel |
| **4-Methoxypyridine** | 1:1 | 0.25:0.51 | No gel | No gel | No gel | No gel |
| **2-Phenylpyridine** | 1:1 | 0.25:0.36 | No gel | No gel | No gel | No gel |
| **3-Phenylpyridine** | 1:1 | 0.25:0.36 | No gel | No gel | No gel | No gel |
| **4-Phenylpyridine** | 1:1 | 0.25:0.36 | No gel | No gel | No gel | No gel |
| **2-Benzylpyridine** | 1:1 | 0.25:0.32 | No gel | No gel | No gel | No gel |
| **2-methyl-4-methoxypyridine** | 1:1 | 0.25:0.40 | No gel | No gel | No gel | No gel |
| **Quinoline** | 1:1 | 0.25:0.43 | No gel | No gel | No gel | No gel |
| **Isoquinoline** | 1:1 | 0.25:0.43 | No gel | No gel | No gel | No gel |

**Table S7.** Gelation experiments using 0.25 w/v% 4-methylpyridine N-oxide (**4**) with AgPF_6_ -AgClO_4_

| **PyNO** | **EtOAc**  **(1.0 mL)** | **Ben**  **(1.0 mL)** | **Tol**  **(1.0 mL)** | **Acet**  **(1.0 mL)** |
| --- | --- | --- | --- | --- |
| **4:AgPF_6_** | No gel | No gel | No gel | No gel |
| **4:AgBF_4_** | No gel | No gel | No gel | No gel |
| **4:AgSbF_6_** | No gel | No gel | No gel | No gel |
| **4:AgClO_4_** | No gel | No gel | No gel | gel |
|  | | | | |
| **PyNO** | **DMSO**  **(1.0 mL** | **DMF**  **(1.0 mL)** | **EtOH**  **(1.0 mL)** | **H_2_O**  **(1.0 mL)** |
| **4:AgPF_6_** | No gel | No gel | No gel | No gel |
| **4:AgBF_4_** | No gel | No gel | No gel | No gel |
| **4:AgSbF_6_** | No gel | No gel | No gel | No gel |
| **4:AgClO_4_** | No gel | No gel | No gel | No gel |

**Table S8.** Gelation experiments of 4-cyano-(**20**) and 4-nitropyridine *N*-oxides (**21**)

| **PyNO**  **(w/v%)** | **AgTFA in**  **w/v%** | **PyNO:AgTFA ratio** | **Toluene**  **(1.0 mL)** | **Benzene**  **(1.0 mL)** | **EtOAc**  **(1.0 mL)** | **Acetone**  **(1.0 mL)** |
| --- | --- | --- | --- | --- | --- | --- |
| **20** (0.25) | 1.8 | 1:4 | No gel | No gel | No gel | No gel |
| **20** (1.1) | 0.5 | 4:1 | No gel | No gel | No gel | No gel |
| **21** (0.25) | 1.5 | 1:4 | No gel | No gel | No gel | No gel |
| **21** (1.3) | 0.5 | 4:1 | No gel | No gel | No gel | No gel |

**Table S9.** Mixed PyNOs (PyNO1+PyNO2) and AgTFA gelation experiments using 0.25 w/v% PyNO concentration

| **PyNO1** | **PyNO2** | **Solvent** | **PyNO1+PyNO2:AgTFA (w/v%)** | **GELS?** |
| --- | --- | --- | --- | --- |
| **1** | **21** | Benzene | 0.25 +0.25:0.58 | Yes |
| **1** | **21** | Toluene | 0.25 +0.25:0.58 | Yes |
| **1** | **21** | EtOAc | 0.25 +0.25:0.58 | Yes |
| **1** | **21** | Acetone | 0.25 +0.25:0.58 | No |
| **1** | **21** | EtOH | 0.25 +0.25:0.58 | No |
| **1** | **23** | Benzene | 0.25 +0.25:0.58 | Yes |
| **1** | **23** | Toluene | 0.25 +0.25:0.58 | Yes |
| **1** | **23** | EtOAc | 0.25 +0.25:0.58 | Yes |
| **1** | **23** | Acetone | 0.25 +0.25:0.58 | No |
| **1** | **23** | EtOH | 0.25 +0.25:0.58 | No |
| **2** | **21** | Benzene | 0.25+0.25:0.50 | Yes |
| **2** | **21** | Toluene | 0.25+0.25:0.50 | Yes |
| **2** | **21** | EtOAc | 0.25+0.25:0.50 | Yes |
| **2** | **21** | Acetone | 0.25+0.25:0.50 | No |
| **2** | **21** | EtOH | 0.25+0.25:0.50 | No |
| **2** | **23** | Benzene | 0.25+0.25:0.50 | Yes |
| **2** | **23** | Toluene | 0.25+0.25:0.50 | Yes |
| **2** | **23** | EtOAc | 0.25+0.25:0.50 | Yes |
| **2** | **23** | Acetone | 0.25+0.25:0.50 | No |
| **2** | **23** | EtOH | 0.25+0.25:0.50 | No |
| **4** | **21** | Benzene | 0.25+0.25:0.50 | Yes |
| **4** | **21** | Toluene | 0.25+0.25:0.50 | Yes |
| **4** | **21** | EtOAc | 0.25+0.25:0.50 | Yes |
| **4** | **21** | Acetone | 0.25+0.25:0.50 | No |
| **4** | **21** | EtOH | 0.25+0.25:0.50 | No |
| **4** | **23** | Benzene | 0.25+0.25:0.50 | Yes |
| **4** | **23** | Toluene | 0.25+0.25:0.50 | Yes |
| **4** | **23** | EtOAc | 0.25+0.25:0.50 | Yes |
| **4** | **23** | Acetone | 0.25+0.25:0.50 | No |
| **4** | **23** | EtOH | 0.25+0.25:0.50 | No |
| **8** | **21** | Benzene | 0.25+0.25:0.45 | Yes |
| **8** | **21** | Toluene | 0.25+0.25:0.45 | Yes |
| **8** | **21** | EtOAc | 0.25+0.25:0.45 | Yes |
| **8** | **21** | Acetone | 0.25+0.25:0.45 | Yes |
| **8** | **21** | EtOH | 0.25+0.25:0.45 | No |
| **8** | **23** | Benzene | 0.25+0.25:0.45 | Yes |
| **8** | **23** | Toluene | 0.25+0.25:0.45 | Yes |
| **8** | **23** | EtOAc | 0.25+0.25:0.45 | Yes |
| **8** | **23** | Acetone | 0.25+0.25:0.45 | Yes |
| **8** | **23** | EtOH | 0.25+0.25:0.45 | No |

**Table S10**. Screening pyridine *N*-oxide (**1**), 2-methylpyridine *N*-oxide (**2**), 4-methylpyridine *N*-oxide (**4**), and 3,4-dimethylpyridine *N*-oxide (**8**) in aprotic and protic solvents to study the influence of solvent polarity on gelation.

| **PyNO (0.25w/v%)** | **2-pentanone** | **3-pentanone** | **2-hexanone** |
| --- | --- | --- | --- |
| **1** | No gel | Gel | No gel |
| **2** | No gel | Gel | No gel |
| **4** | No gel | Gel | Gel |
| **8** | Gel | Gel | Gel |
|  | | | |
| **PyNO (0.5w/v%)** | **2-pentanone** | **3-pentanone** | **2-hexanone** |
| **1** | Gel | Gel | Gel |
| **2** | Gel | Gel | Gel |
| **4** | Gel | Gel | Gel |
| **8** | Gel | Gel | Gel |

| **PyNO (0.25w/v%)** | **6-methyl-2-heptanol** | **1-hexanol** | **1-pentanol** | **1-butanol** | **1-propanol** |
| --- | --- | --- | --- | --- | --- |
| **1** | No gel | No gel | No gel | No gel | No gel |
| **2** | No gel | No gel | No gel | No gel | No gel |
| **4** | No gel | No gel | No gel | No gel | No gel |
| **8** | No gel | No gel | No gel | No gel | No gel |
|  | | | | | |
| **PyNO (0.5w/v%)** | **6-methyl-2-heptanol** | **1-hexanol** | **1-pentanol** | **1-butanol** | **1-propanol** |
| **1** | No gel | No gel | No gel | No gel | No gel |
| **2** | No gel | No gel | No gel | No gel | No gel |
| **4** | No gel | No gel | No gel | No gel | No gel |
| **8** | No gel | No gel | No gel | No gel | No gel |

**Table S11.** Critical gel concentration (CGC) tests of 11 gel-forming pyridine *N*-oxides

| **PyNO** | **Toluene (1.0 mL)** | **CGC (w/v% of PyNO)** |
| --- | --- | --- |
| **1** | gel | 0.10 |
| **2** | gel | 0.20 |
| **3** | gel | 0.25 |
| **4** | gel | 0.15 |
| **5** | gel | 0.30 |
| **6** | gel | 0.20 |
| **8** | gel | 0.25 |
| **10** | gel | 0.25 |
| **12** | gel | 0.09 |
| **19** | gel | 0.10 |
| **27** | gel | 0.10 |

**
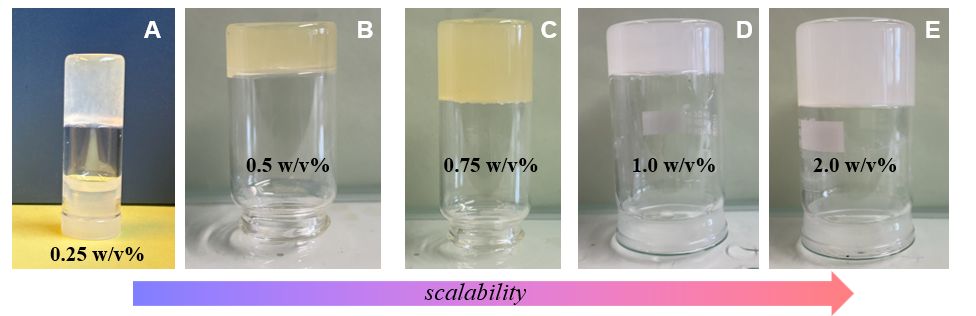
**

**Figure S2.** Scalability of **(2Ag)Tol** at A) 0.25 w/v% PyNO (2.5 mg of PyNO, 1 mL of toluene), B) 0.5 w/v% PyNO (10.0 mg, 2 mL of toluene), C) 0.75 w/v% PyNO (60 mg of PyNO, 8 mL of toluene), D) 1.0 w/v% PyNO (200 mg of PyNO, 20 mL of toluene), and E) 2.0 w/v% PyNO (900 mg of PyNO, 45 mL of toluene). The given amount of toluene is distributed evenly to dissolve PyNO and AgTFA and then mixed to prepare gels. The PyNO in toluene is warmed at 80 °C to dissolve the PyNO homogeneously.

**3. Scanning electron microscopy photographs**

**
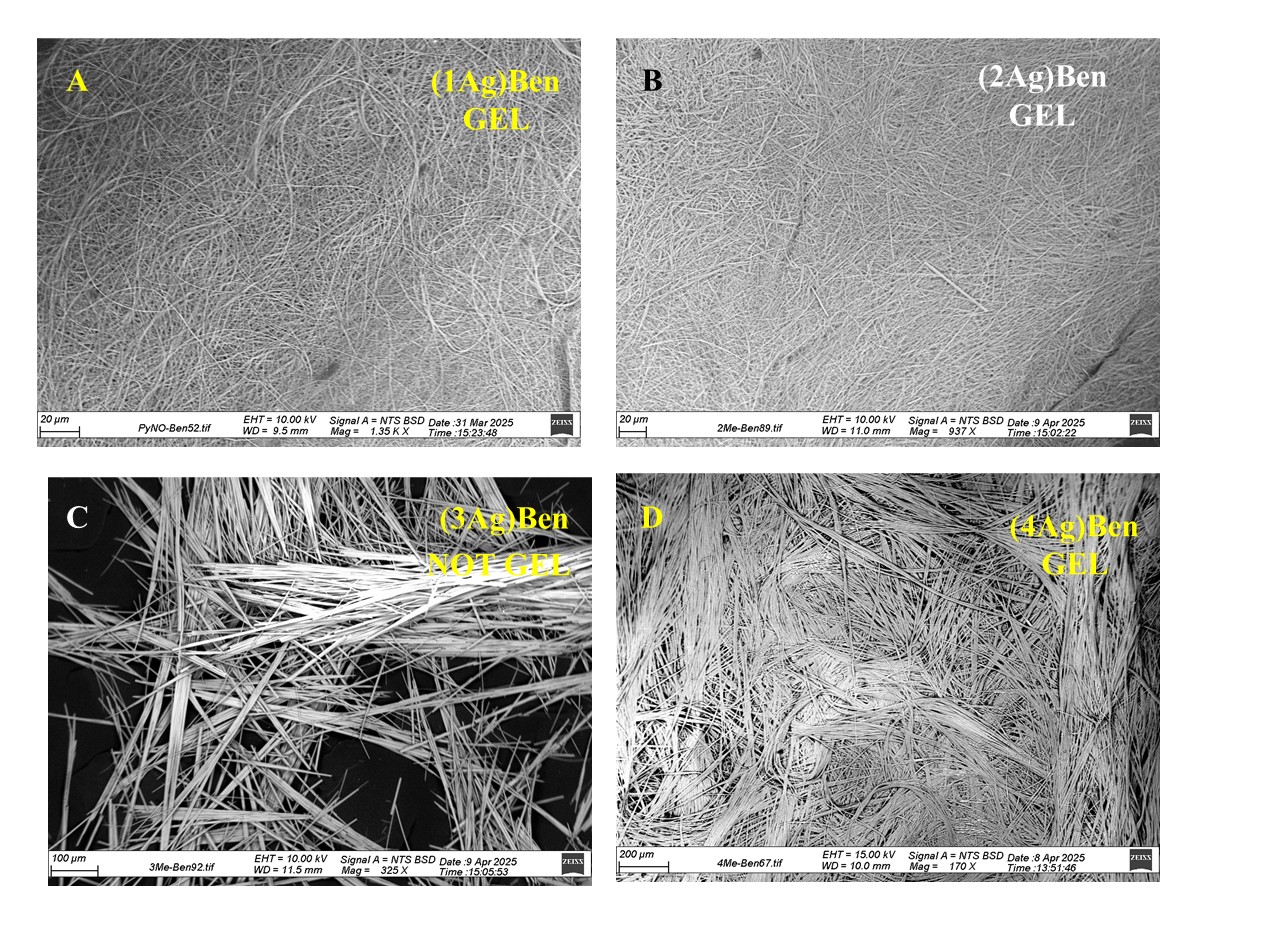
**

**Figure S3.** SEM photographs of A) **(1Ag)Ben** B) **(2Ag)Ben**, C) **(3Ag)Ben**, and D) **(4Ag)Ben** at 0.25 w/v% of PyNO

**
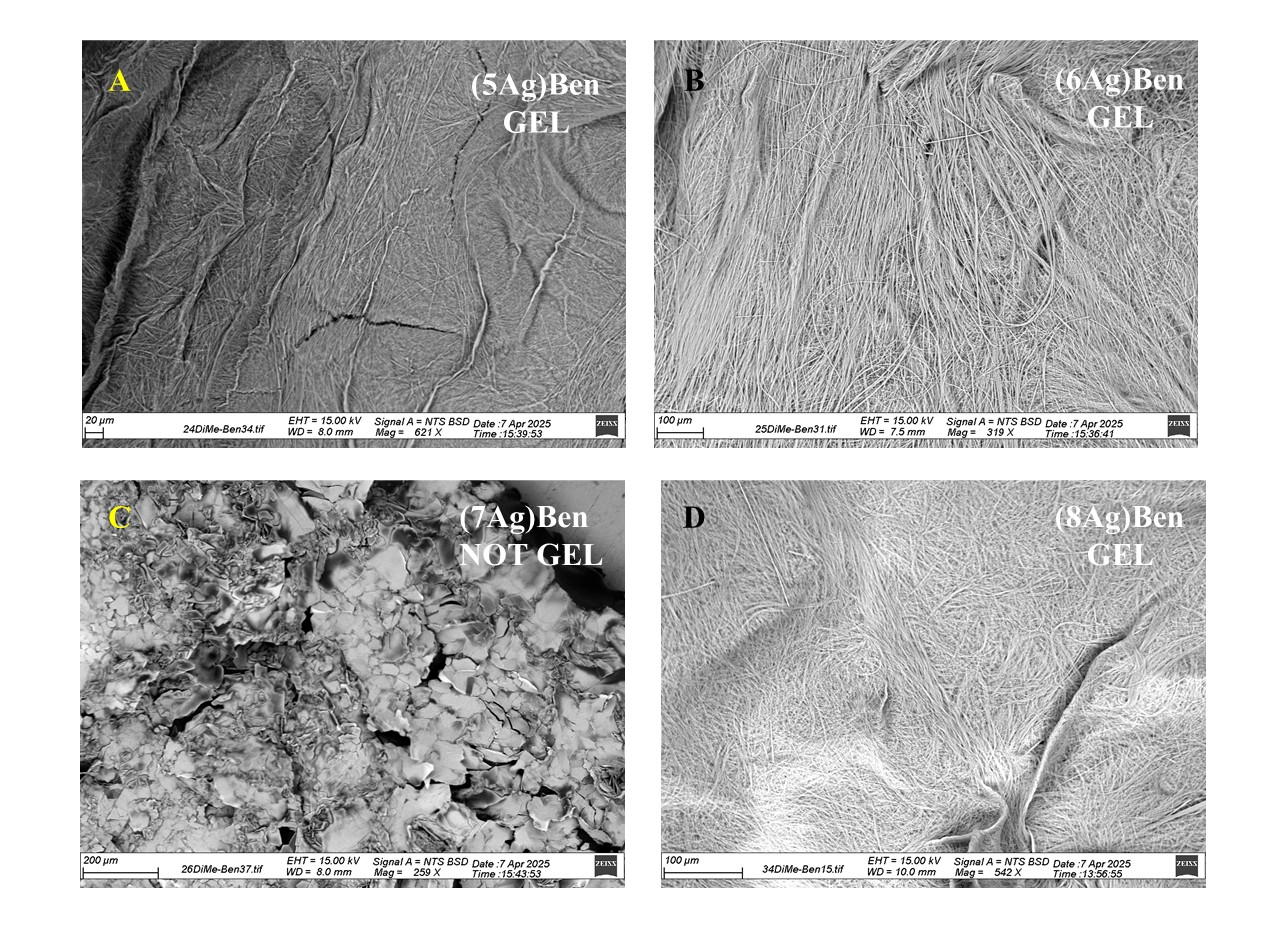
**

**Figure S4.** SEM photographs of A) **(5Ag)Ben** B) **(6Ag)Ben**, C) **(7Ag)Ben**, and D) **(8Ag)Ben** at 0.25 w/v% of PyNO

**
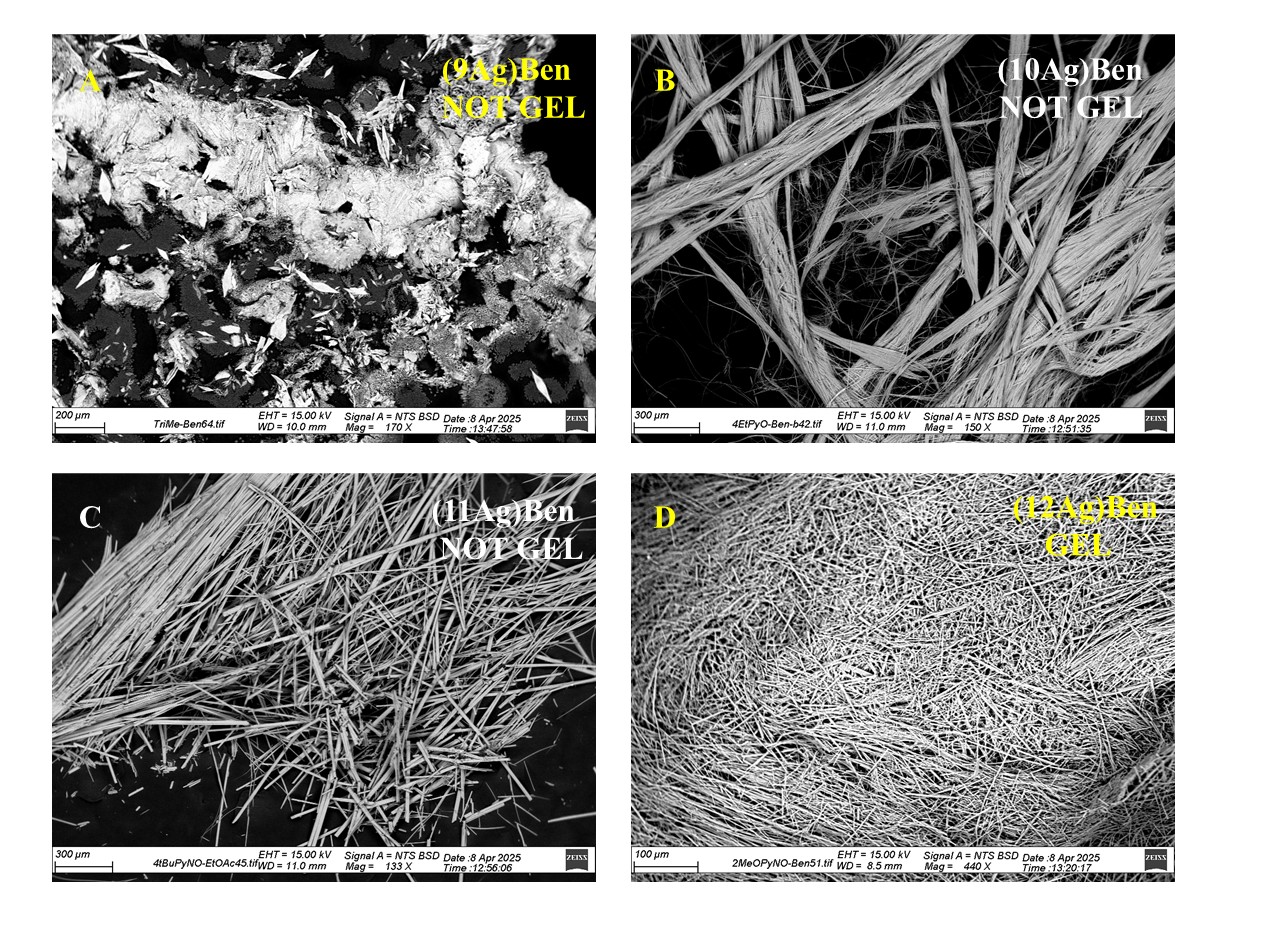
**

**Figure S5.** SEM photographs of A) **(9Ag)Ben** B) **(10Ag)Ben**, C) **(11Ag)Ben**, and D) **(12Ag)Ben** at 0.25 w/v% of PyNO

**
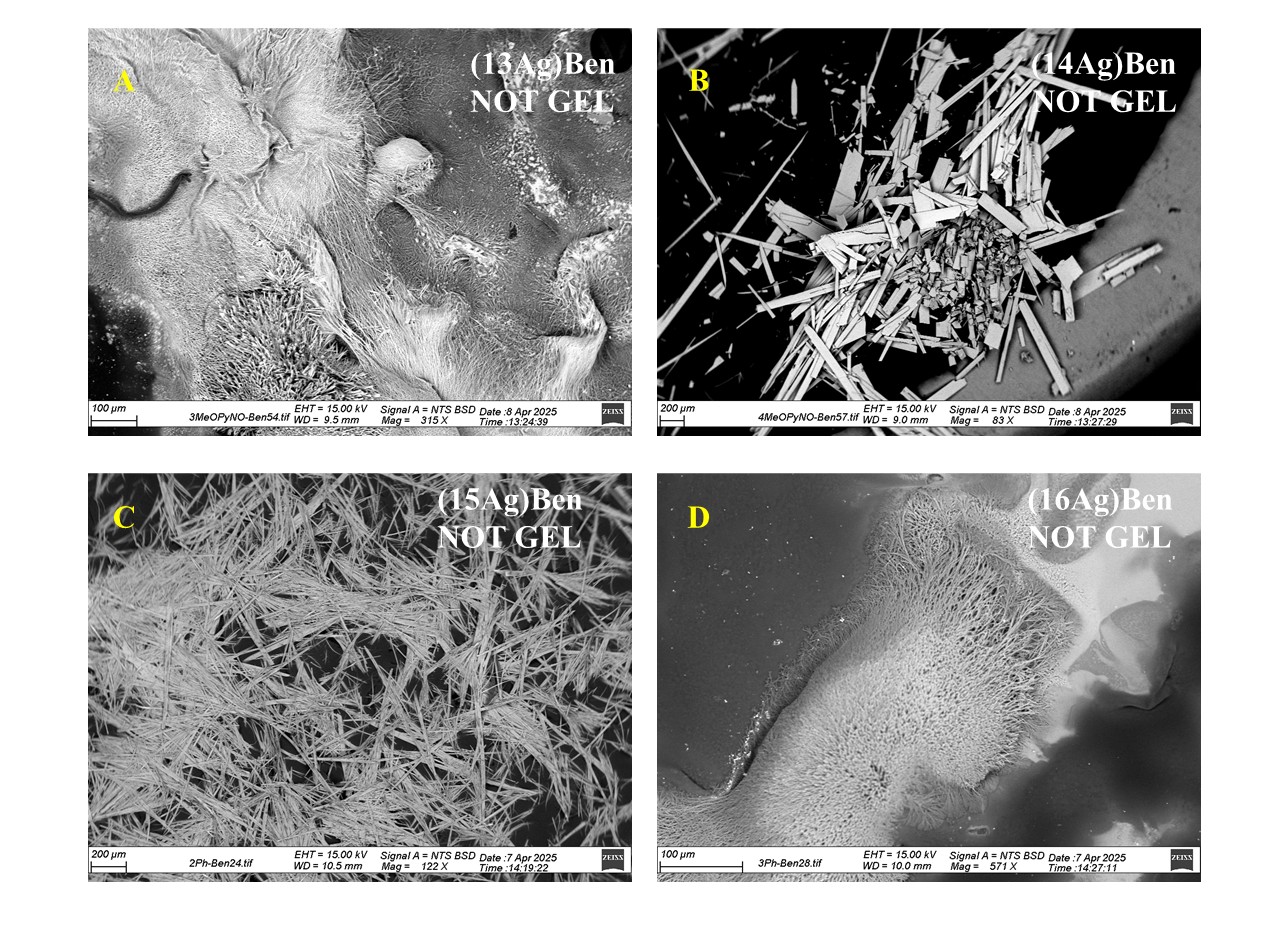
**

**Figure S6.** SEM photographs of A) **(13Ag)Ben** B) **(14Ag)Ben**, C) **(15Ag)Ben**, and D) **(16Ag)Ben** at 0.25 w/v% of PyNO

**
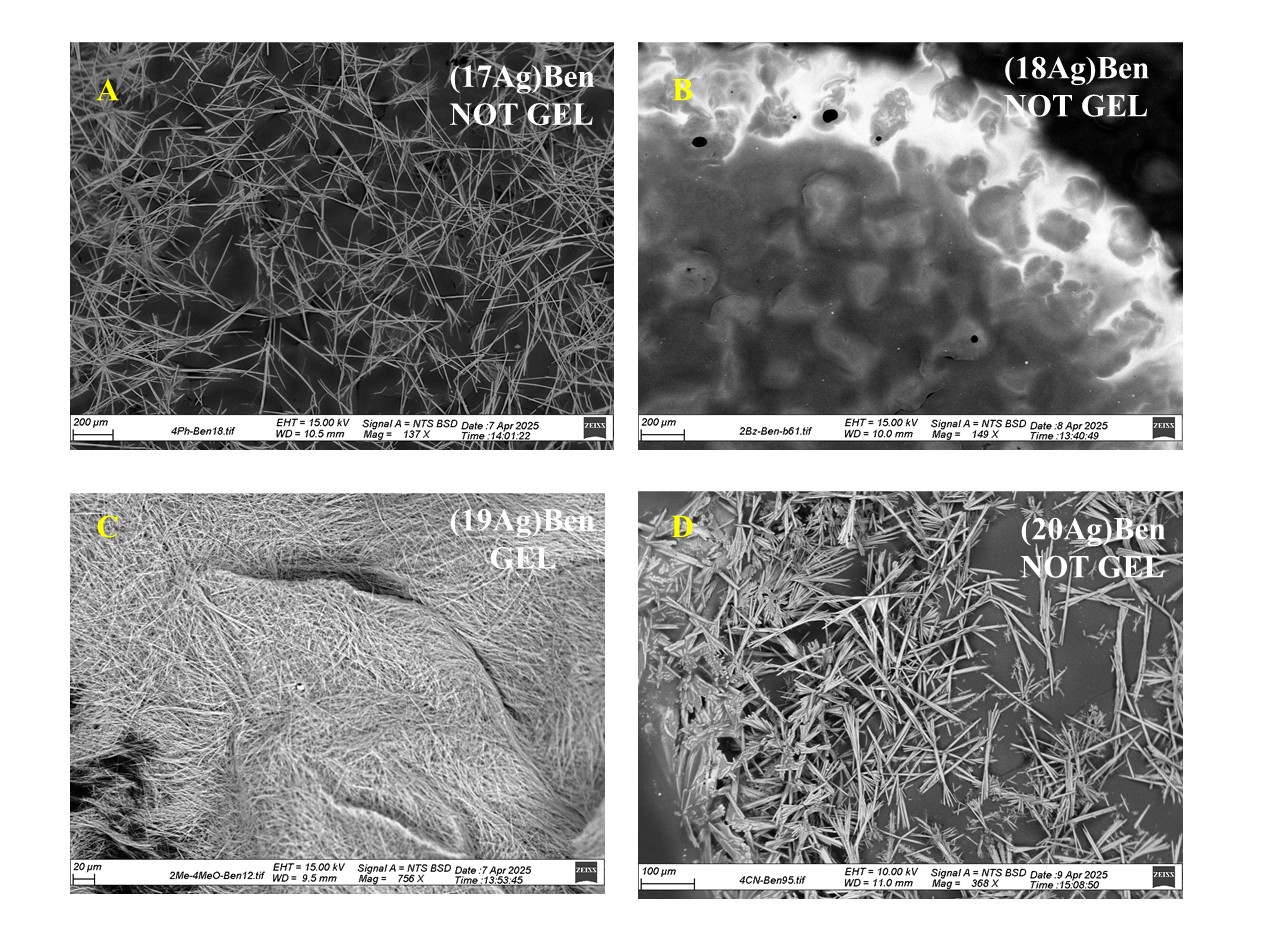
**

**Figure S7.** SEM photographs of A) **(17Ag)Ben** B) **(18Ag)Ben**, C) **(19Ag)Ben**, and D) **(20Ag)Ben** at 0.25 w/v% of PyNO

**
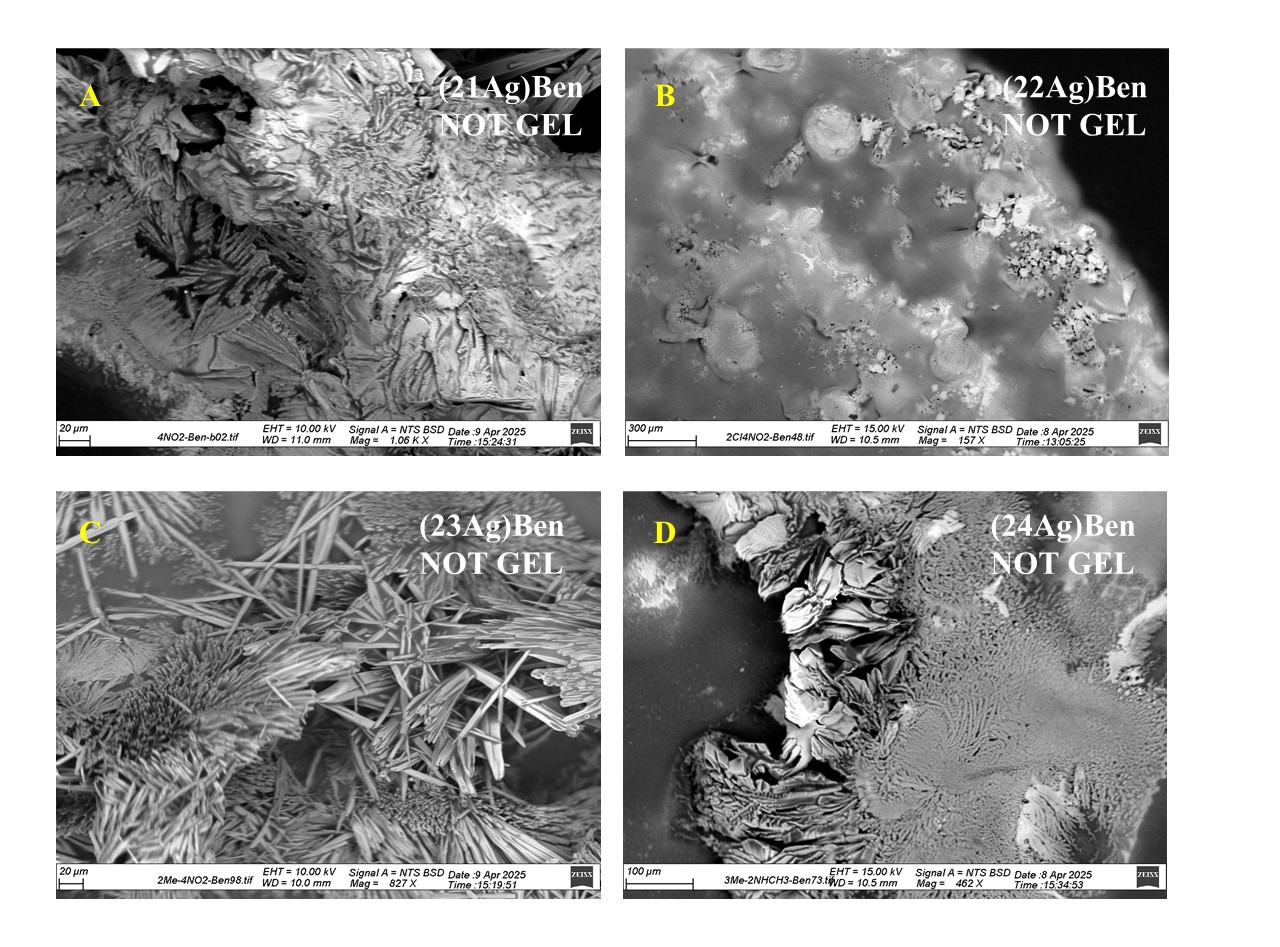
**

**Figure S8.** SEM photographs of A) **(21Ag)Ben** B) **(22Ag)Ben**, C) **(23Ag)Ben**, and D) **(24Ag)Ben** at 0.25 w/v% of PyNO

**
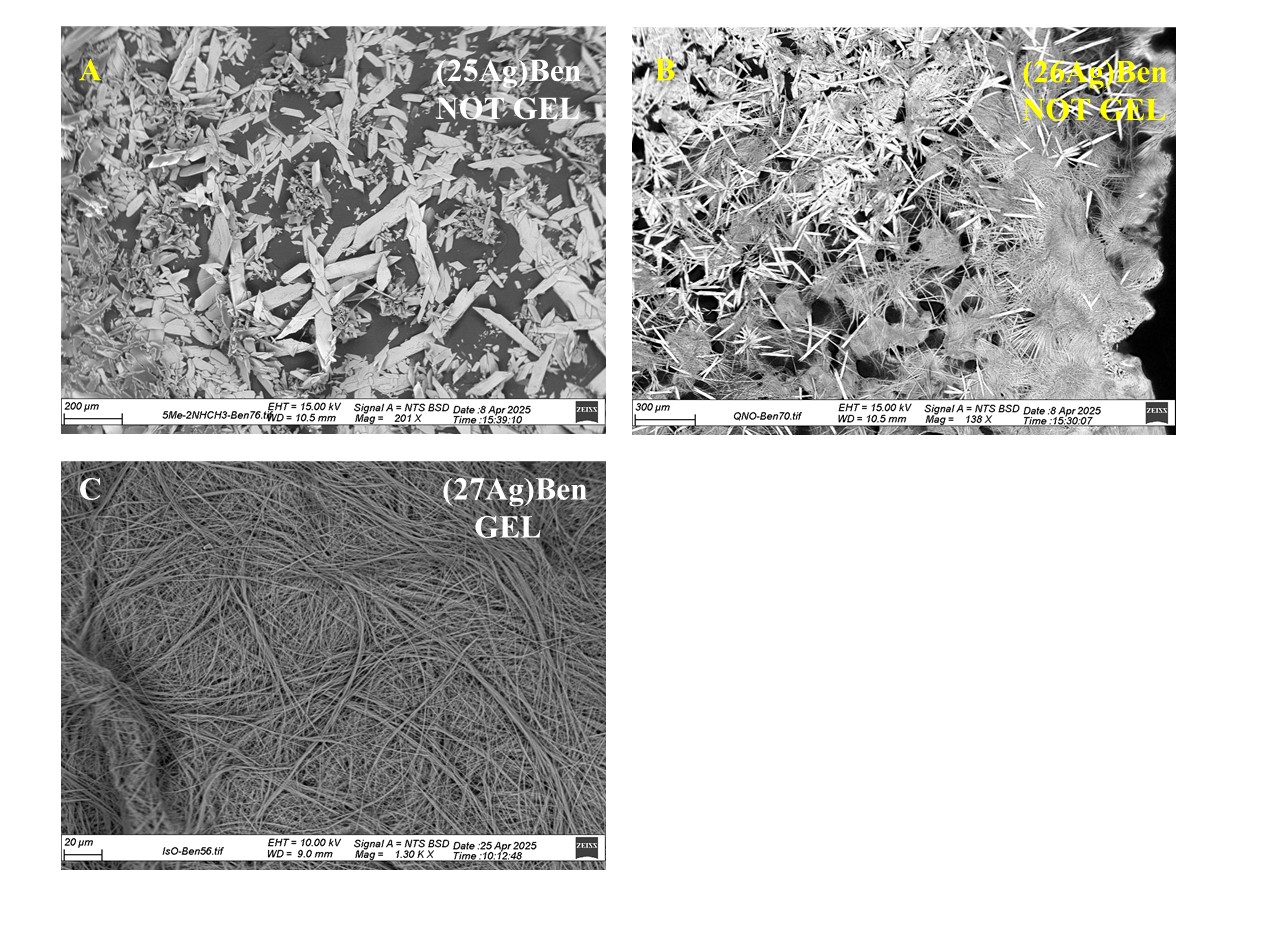
**

**Figure S9.** SEM photographs of A) **(25Ag)Ben** B) **(26Ag)Ben**, and D) **(27Ag)Ben** at 0.25 w/v% of PyNO

**
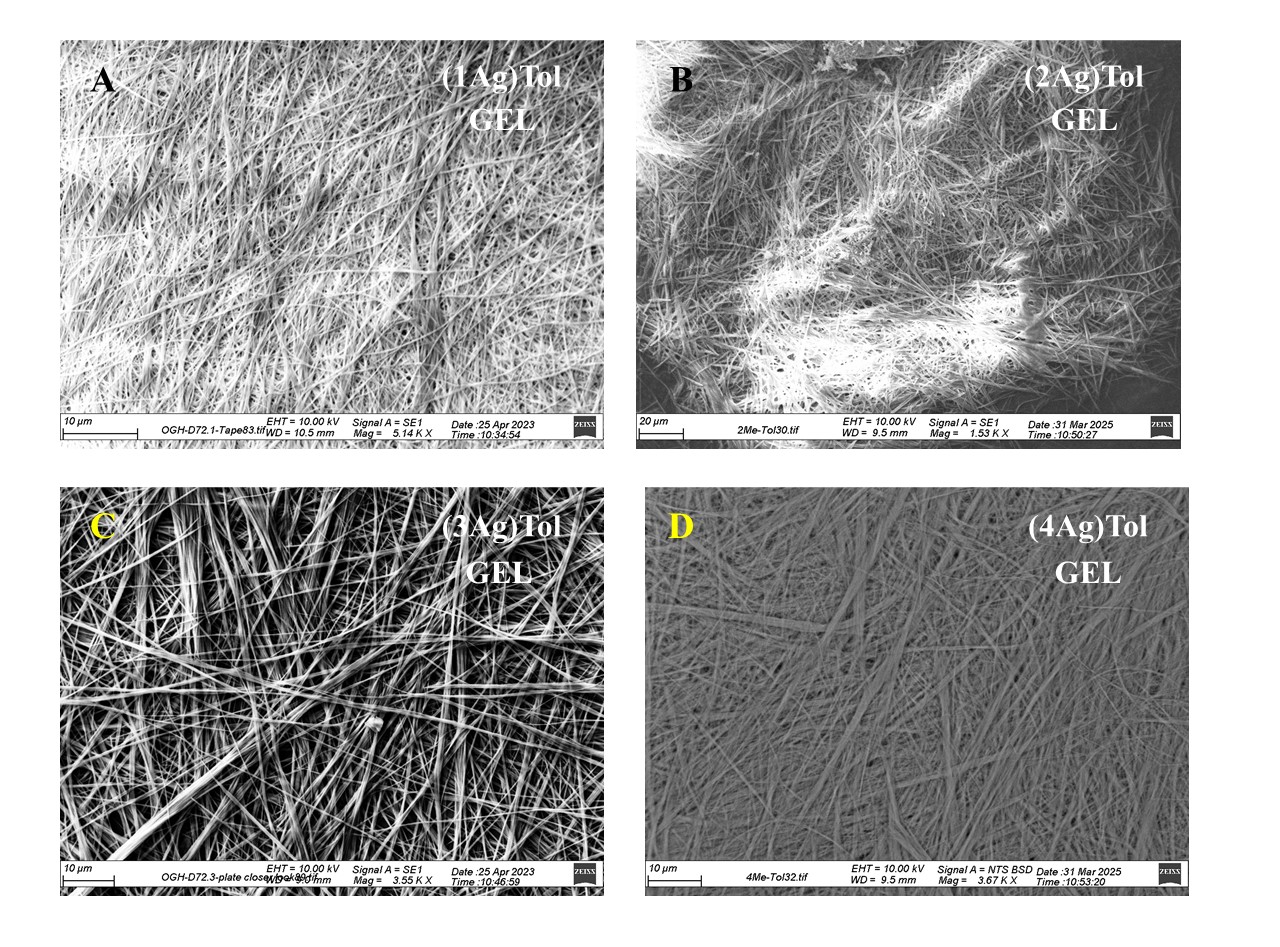
**

**Figure S10.** SEM photographs of A) **(1Ag)Tol** B) **(2Ag)Tol**, C) **(3Ag)Tol**, and D) **(4Ag)Tol** at 0.25 w/v% of PyNO

**
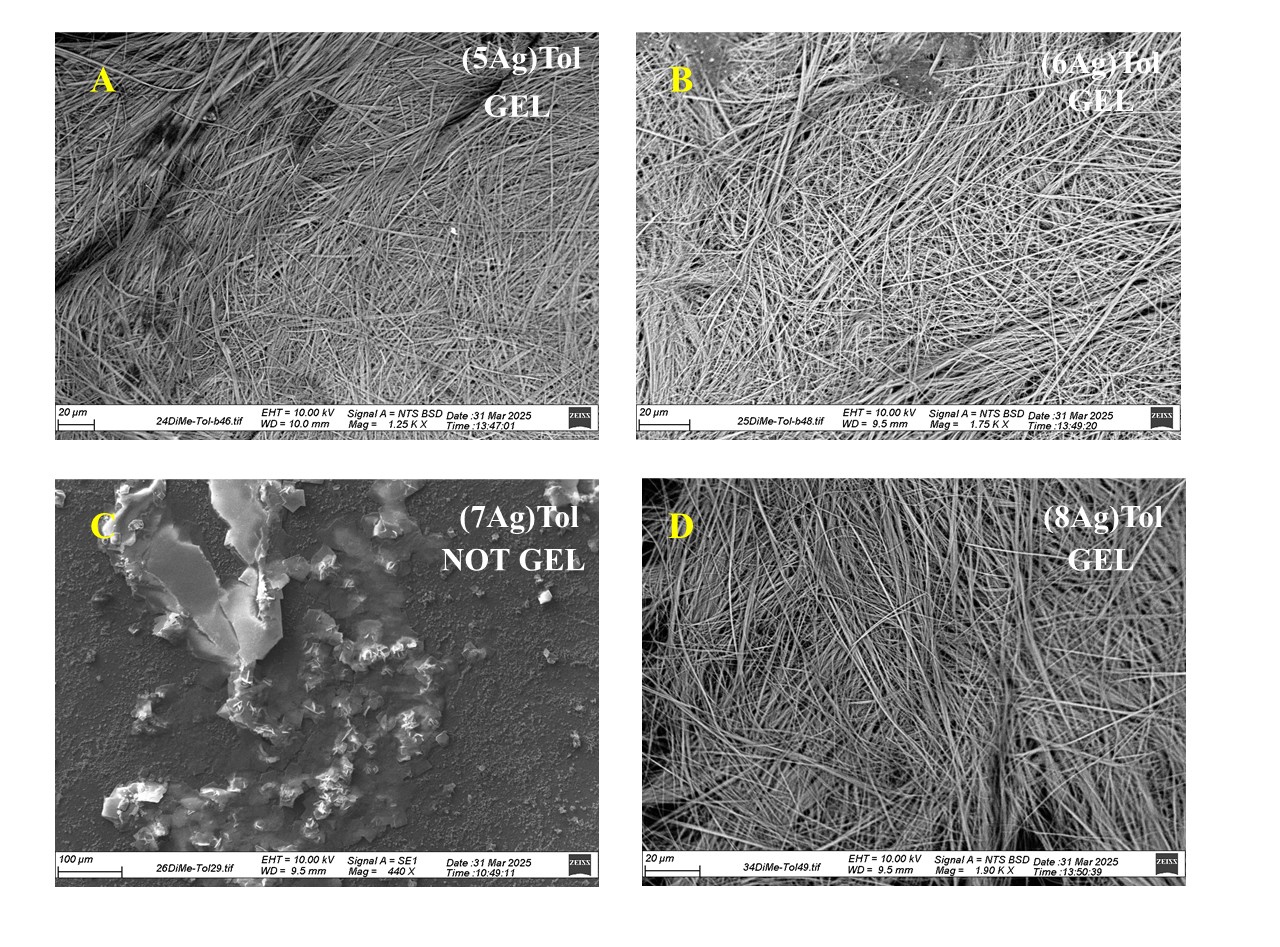
**

**Figure S11.** SEM photographs of A) **(5Ag)Tol** B) **(6Ag)Tol**, C) **(7Ag)Tol**, and D) **(8Ag)Tol** at 0.25 w/v% of PyNO

**
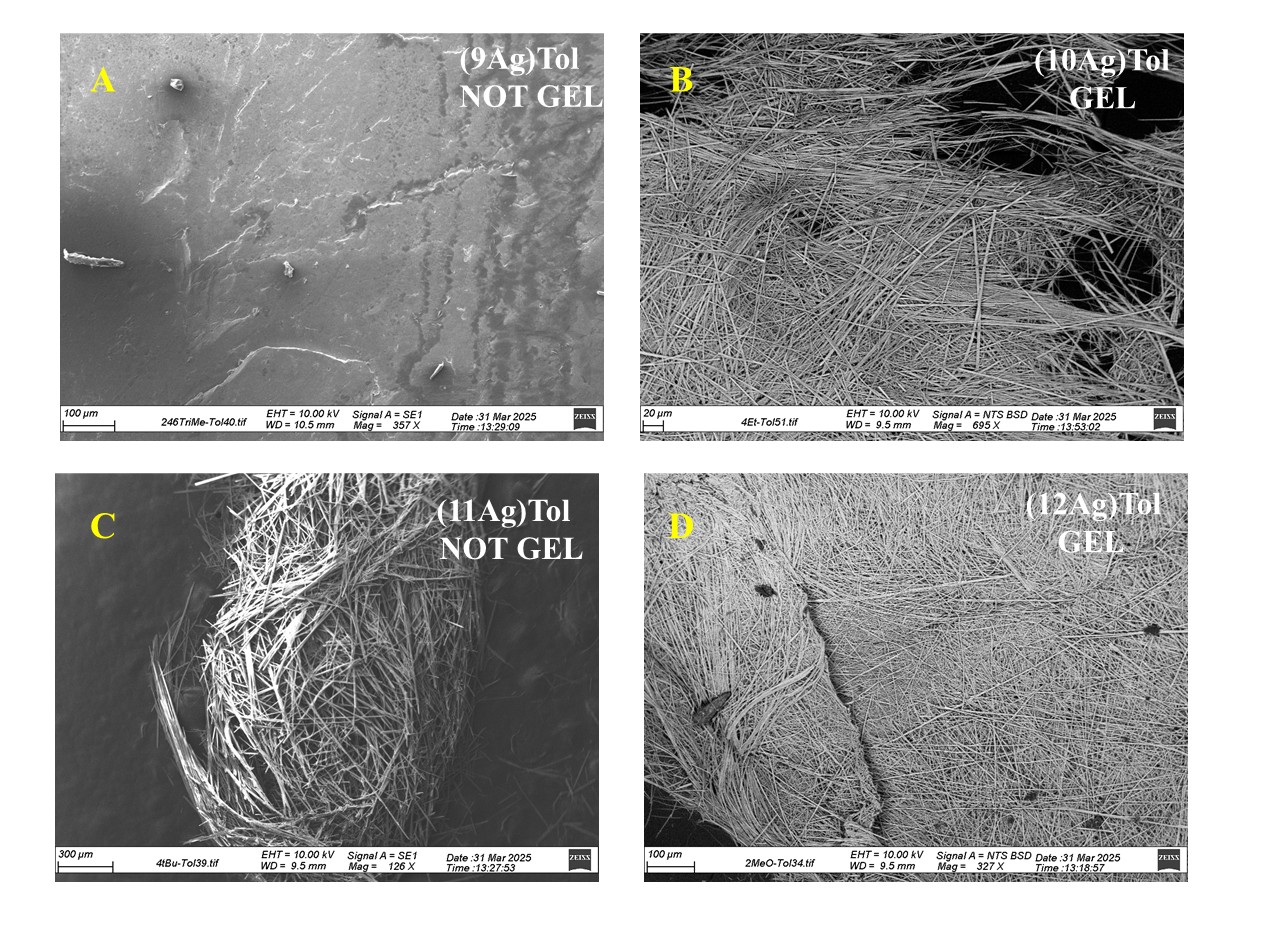
**

**Figure S12.** SEM photographs of A) **(9Ag)Tol** B) **(10Ag)Tol**, C) **(11Ag)Tol**, and D) **(12Ag)Tol** at 0.25 w/v% of PyNO

**
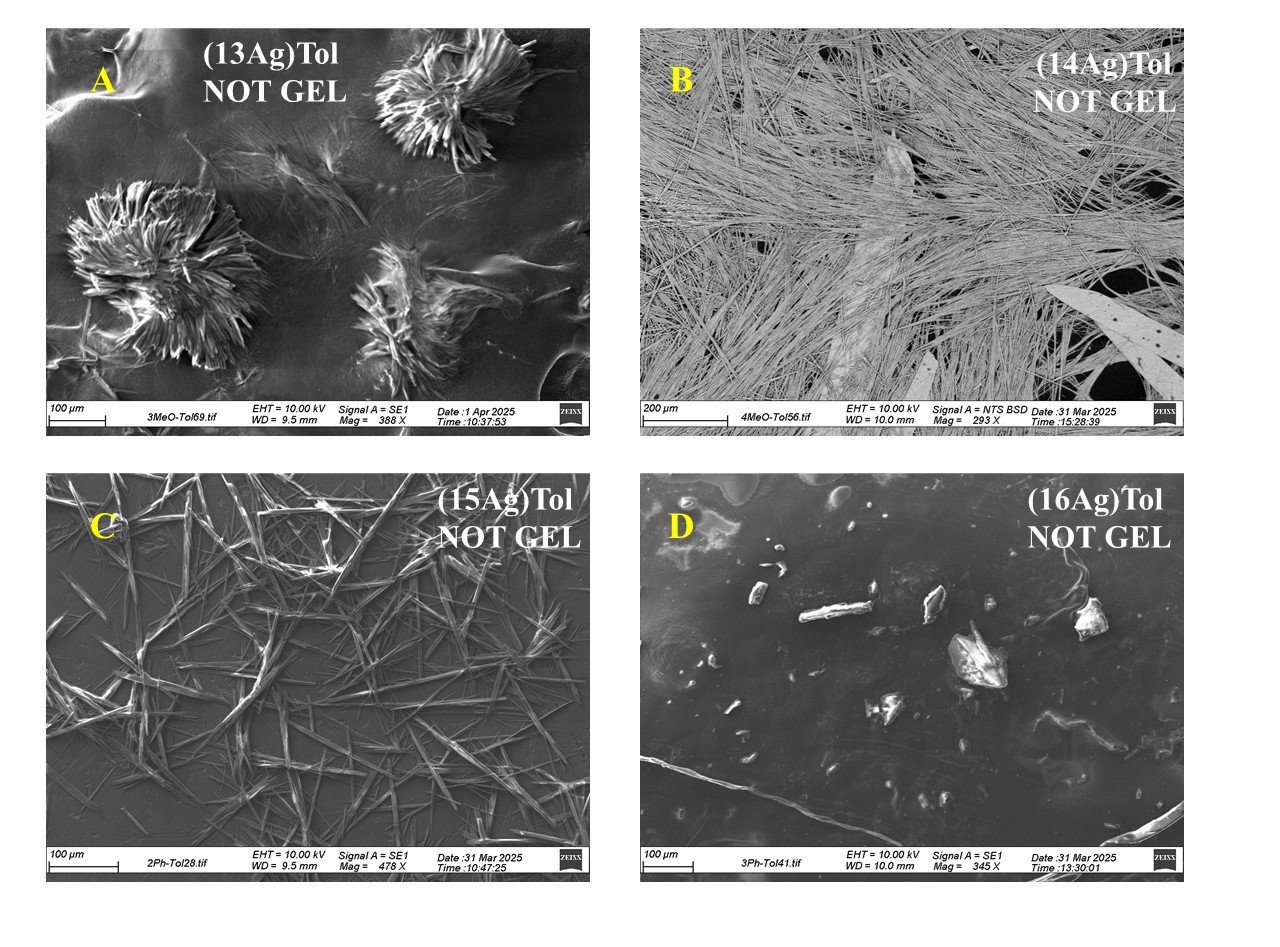
**

**Figure S13.** SEM photographs of A) **(13Ag)Tol** B) **(14Ag)Tol**, C) **(15Ag)Tol**, and D) **(16Ag)Tol** at 0.25 w/v% of PyNO

**
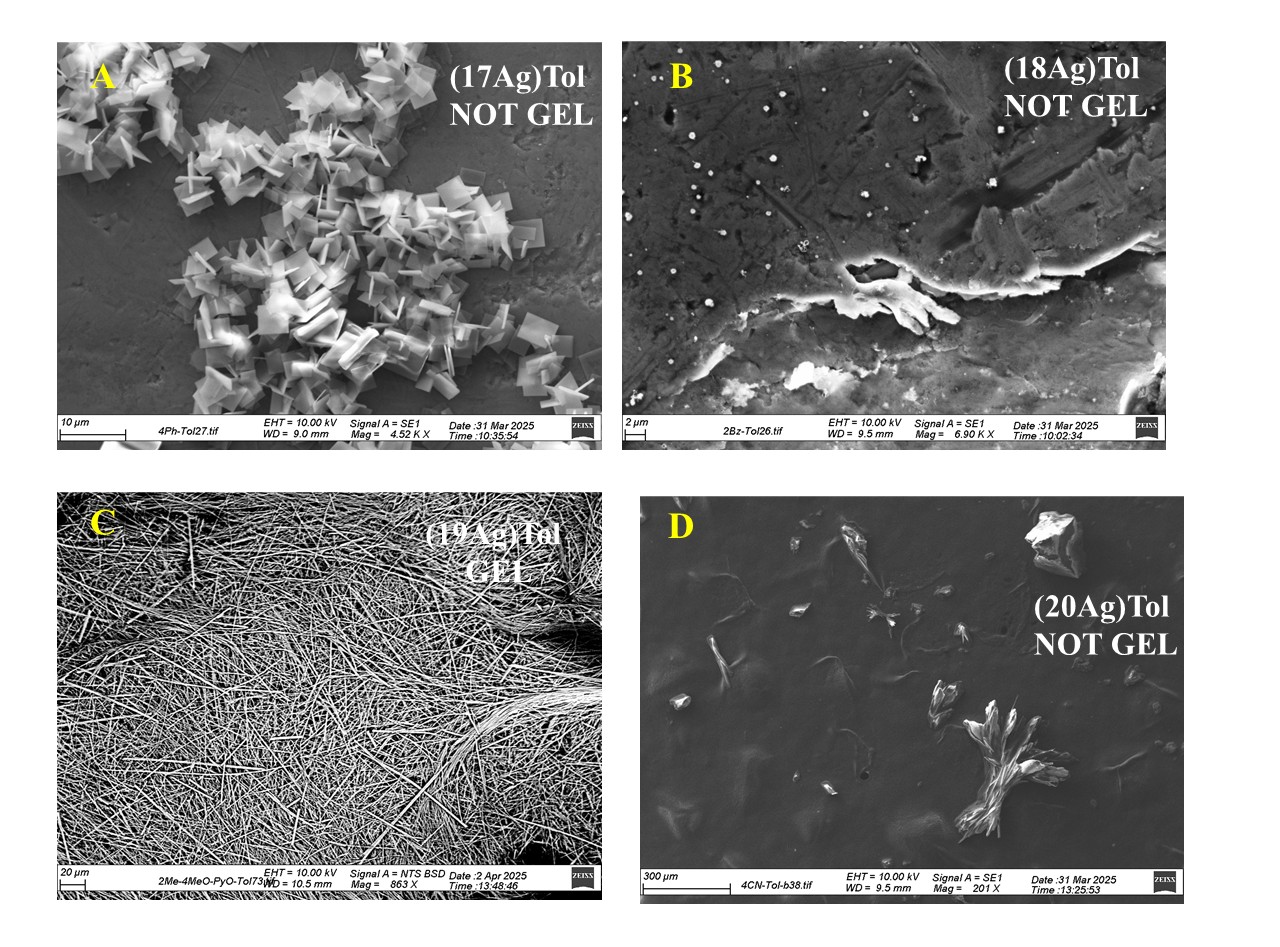
**

**Figure S14.** SEM photographs of A) **(17Ag)Tol** B) **(18Ag)Tol**, C) **(19Ag)Tol**, and D) **(20Ag)Tol** at 0.25 w/v% of PyNO

**
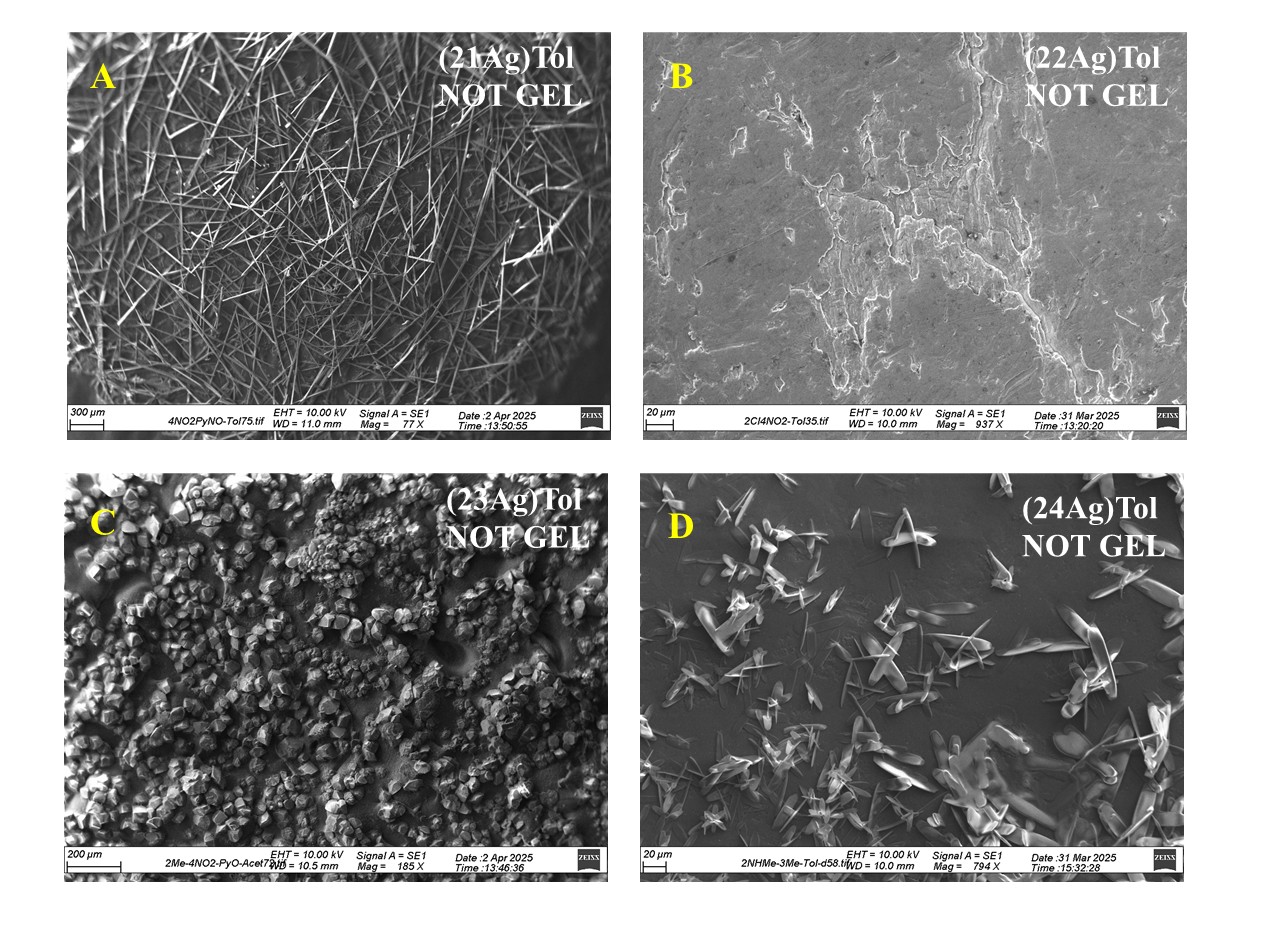
**

**Figure S15.** SEM photographs of A) **(21Ag)Tol** B) **(22Ag)Tol**, C) **(23Ag)Tol**, and D) **(24Ag)Tol** at 0.25 w/v% of PyNO

**
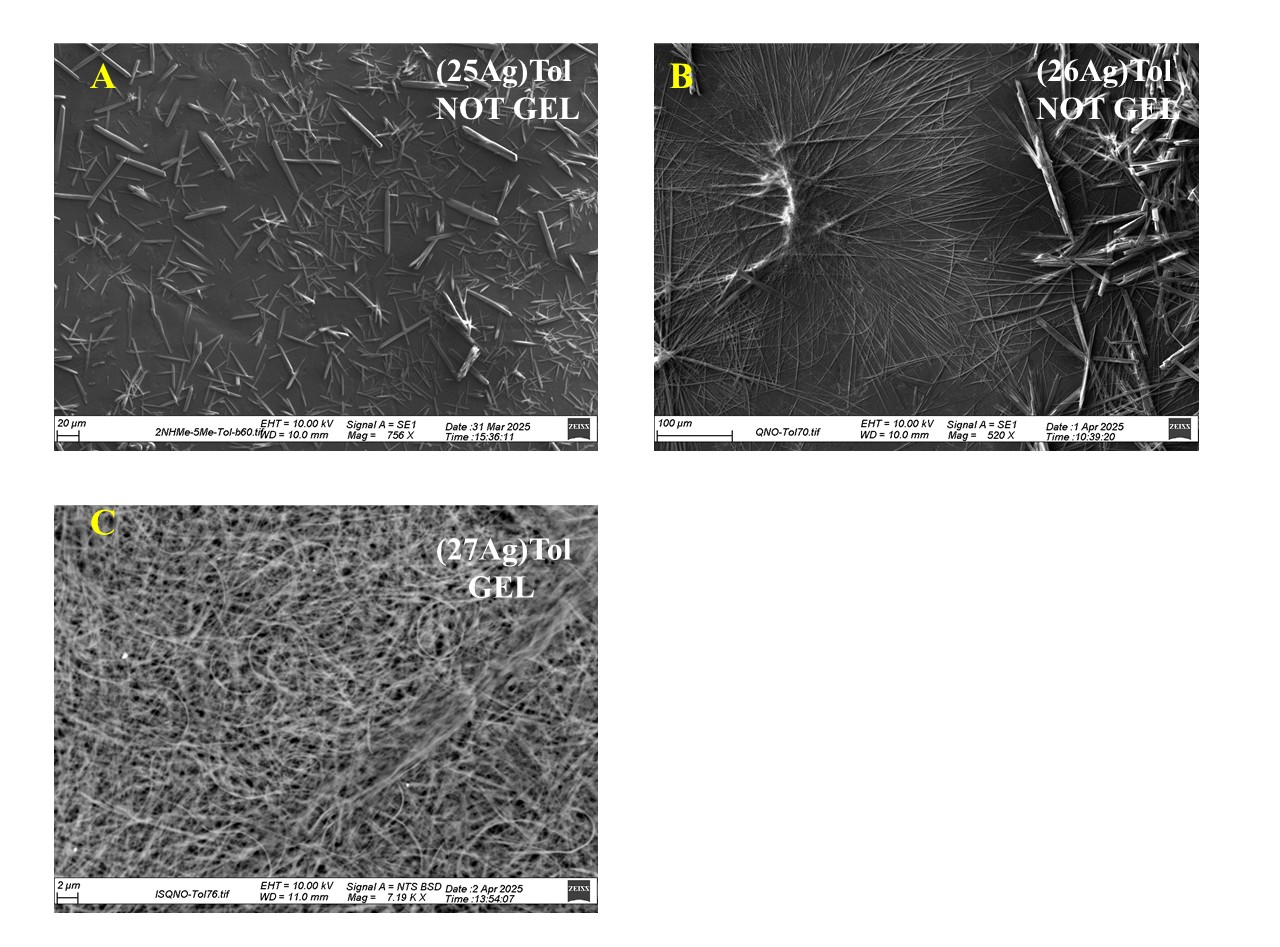
**

**Figure S16.** SEM photographs of A) **(25Ag)Tol** B) **(26Ag)Tol**, and C) **(27Ag)Tol** at 0.25 w/v% of PyNO

**
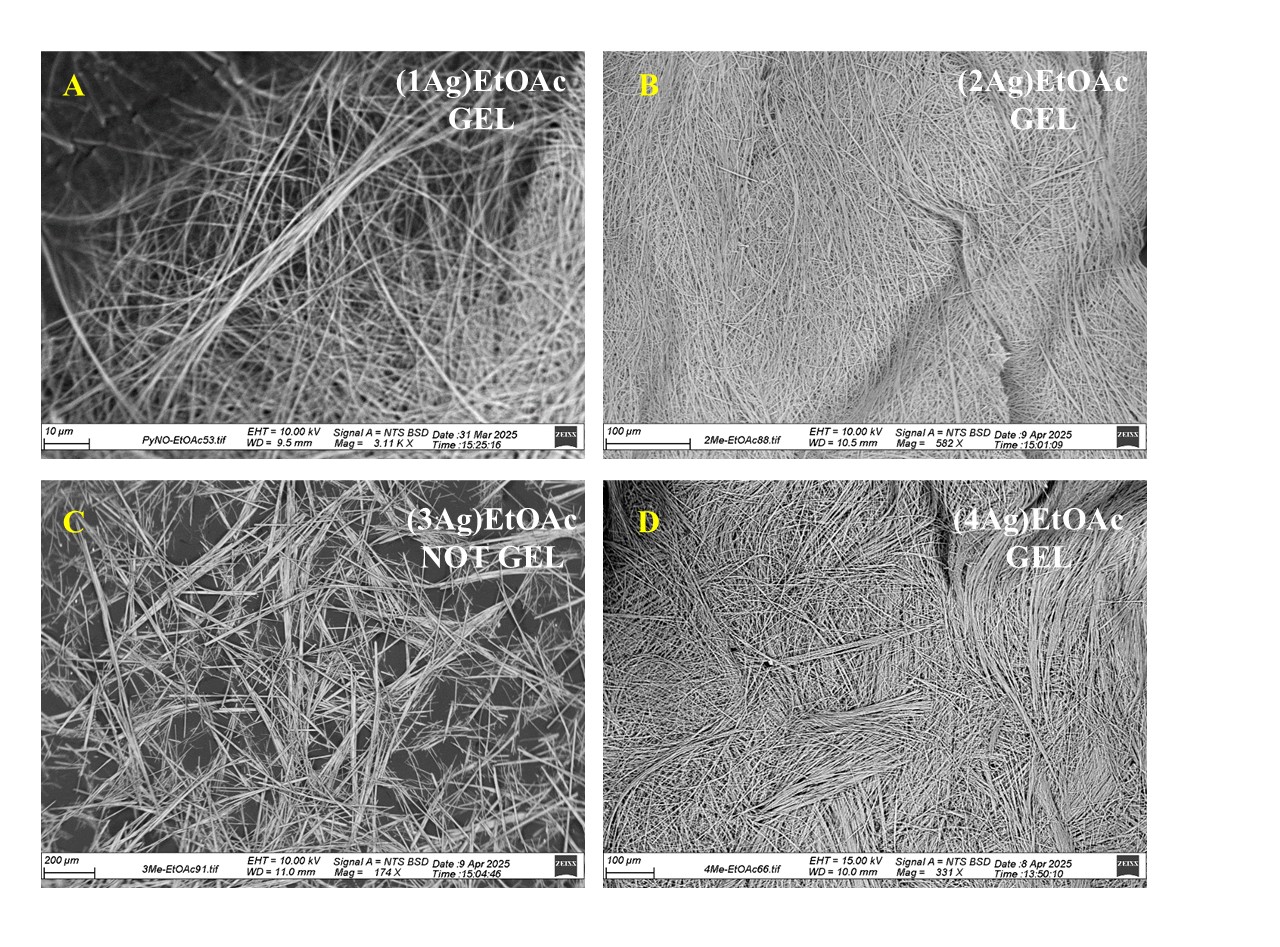
**

**Figure S17.** SEM photographs of A) **(1Ag)EtOAc**, B) **(2Ag)EtOAc**, C) **(3Ag)EtOAc,** and D) **(4Ag)EtOAc** at 0.25 w/v% of PyNO

**
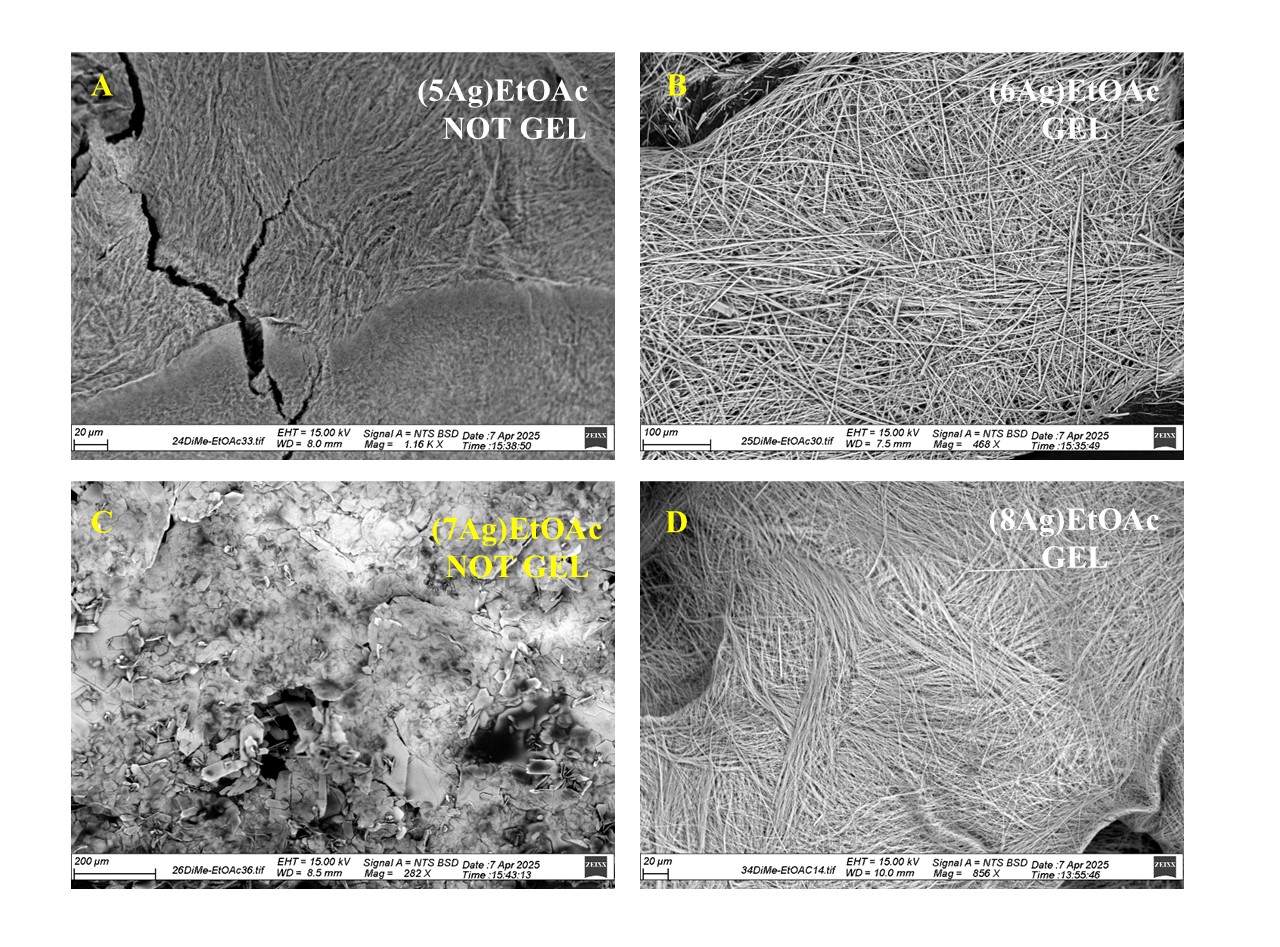
**

**Figure S18.** SEM photographs of A) **(5Ag)EtOAc**, B) **(6Ag)EtOAc**, C) **(7Ag)EtOAc,** and D) **(8Ag)EtOAc** at 0.25 w/v% of PyNO

**
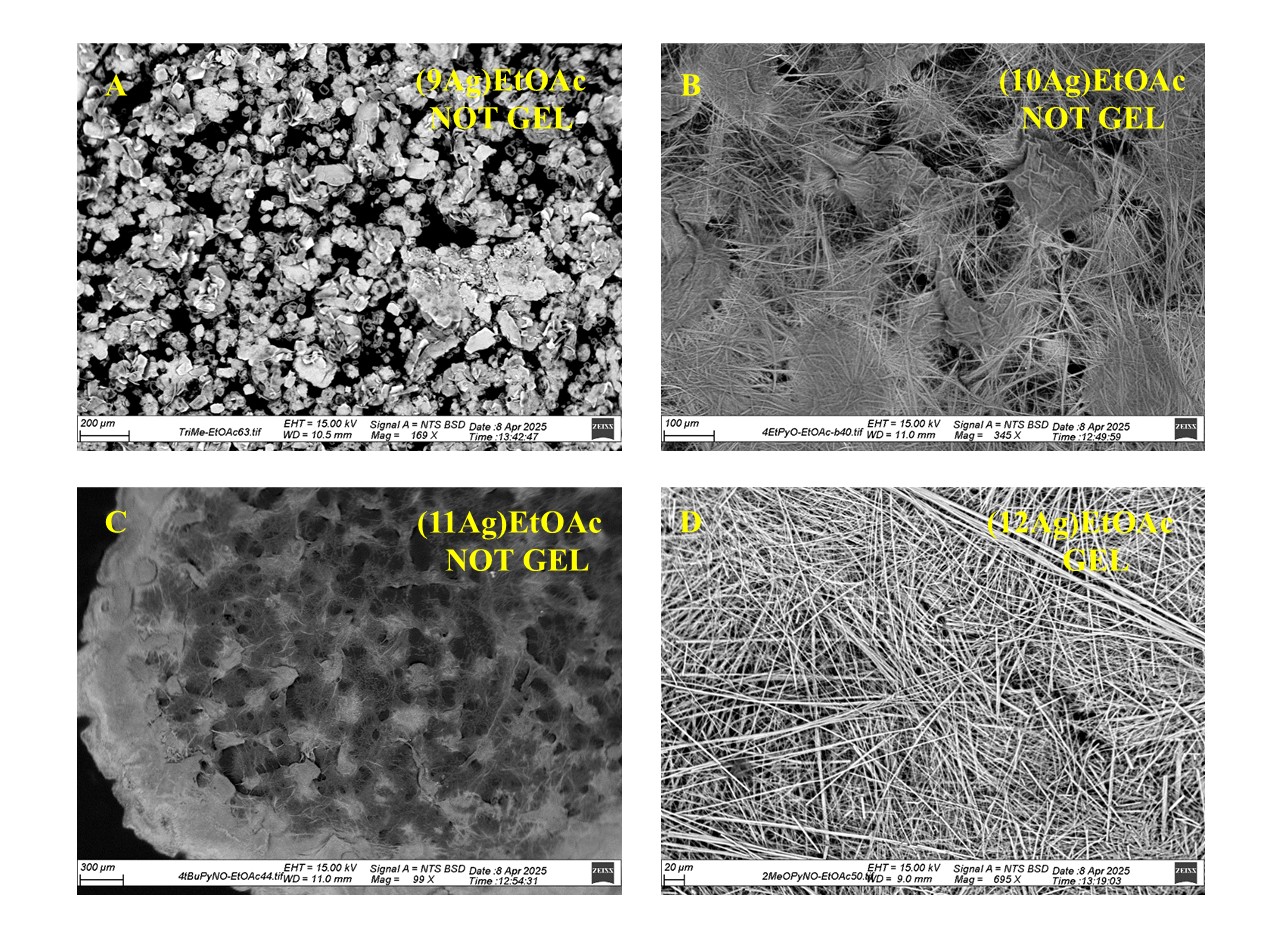
**

**Figure S19.** SEM photographs of A) **(9Ag)EtOAc**, B) **(10Ag)EtOAc**, C) **(11Ag)EtOAc,** and D) **(12Ag)EtOAc** at 0.25 w/v% of PyNO

**
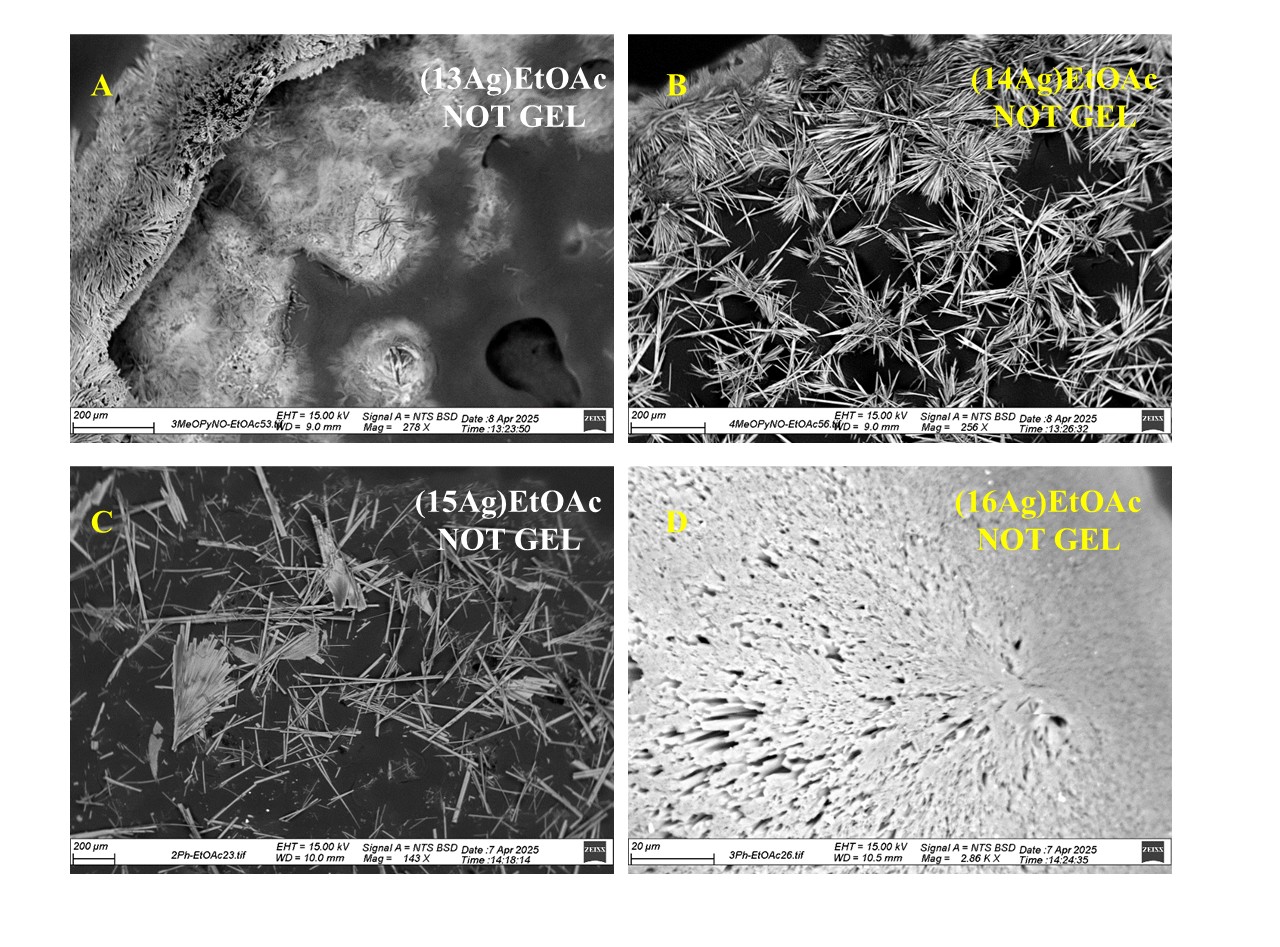
**

**Figure S20.** SEM photographs of A) **(13Ag)EtOAc**, B) **(14Ag)EtOAc**, C) **(15Ag)EtOAc,** and D) **(16Ag)EtOAc** at 0.25 w/v% of PyNO

**
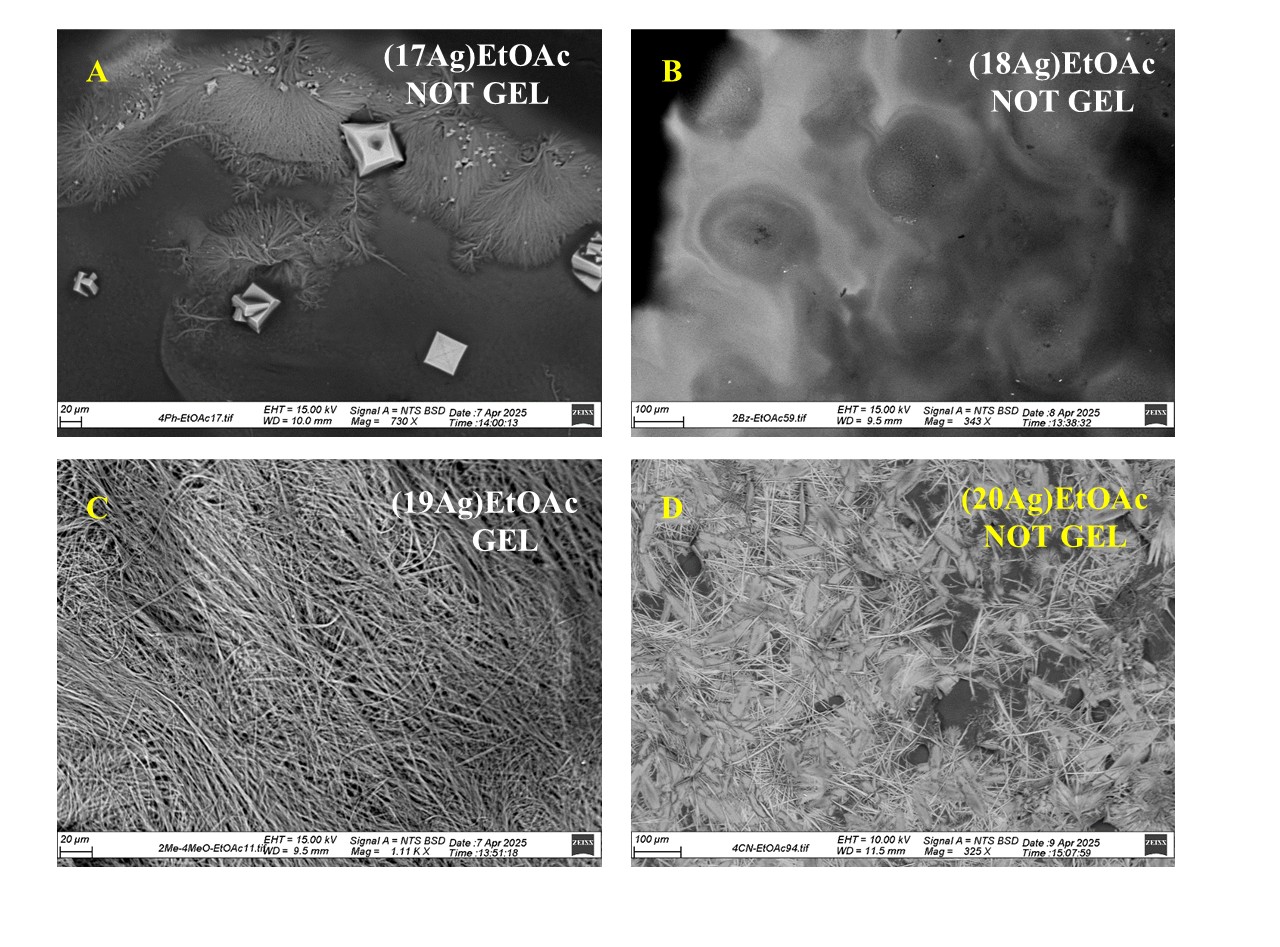
**

**Figure S21.** SEM photographs of A) **(17Ag)EtOAc**, B) **(18Ag)EtOAc**, C) **(19Ag)EtOAc,** and D) **(20Ag)EtOAc** at 0.25 w/v% of PyNO

**
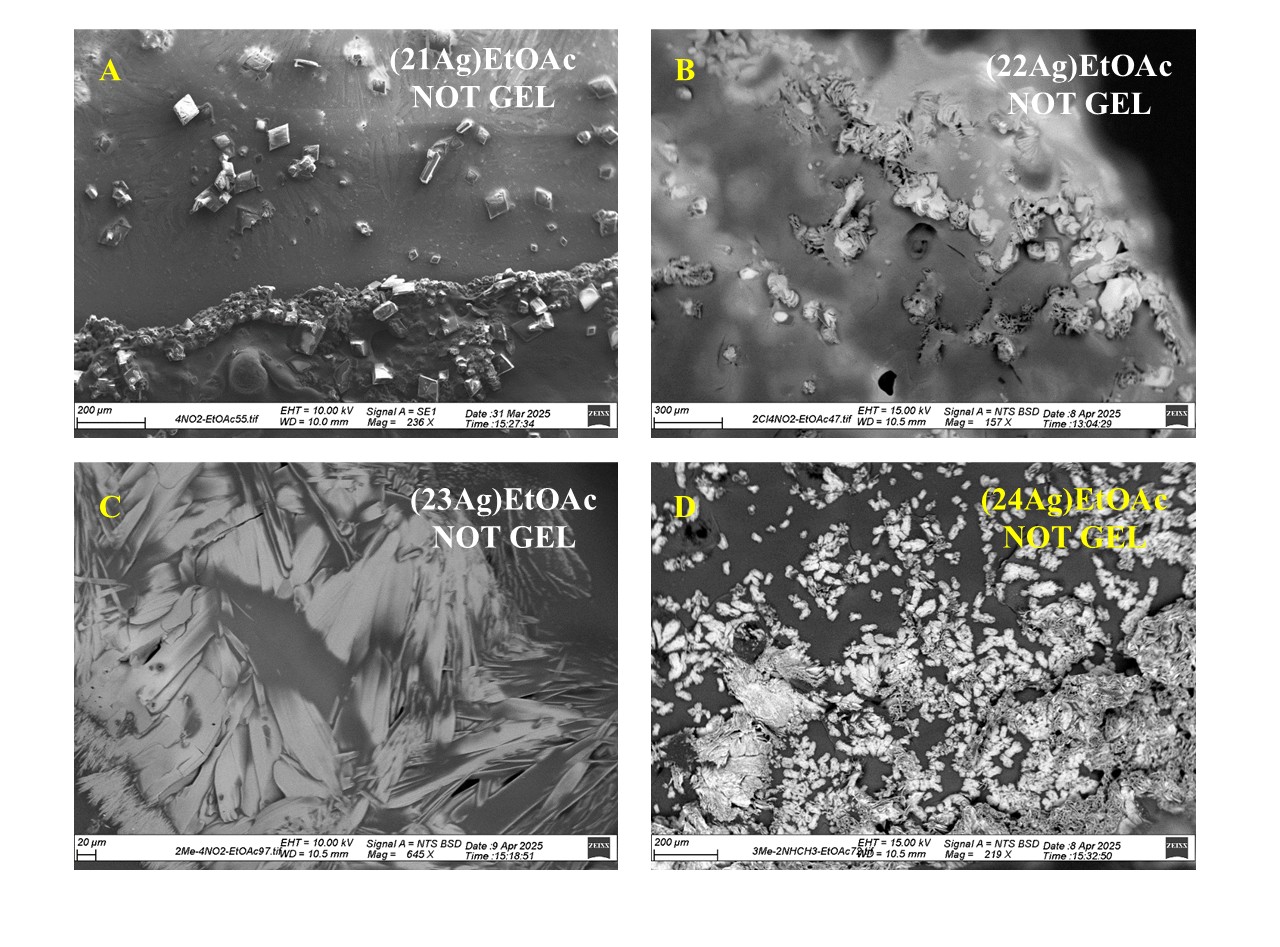
**

**Figure S22.** SEM photographs of A) **(21Ag)EtOAc**, B) **(22Ag)EtOAc**, C) **(23Ag)EtOAc,** and D) **(24Ag)EtOAc** at 0.25 w/v% of PyNO

**
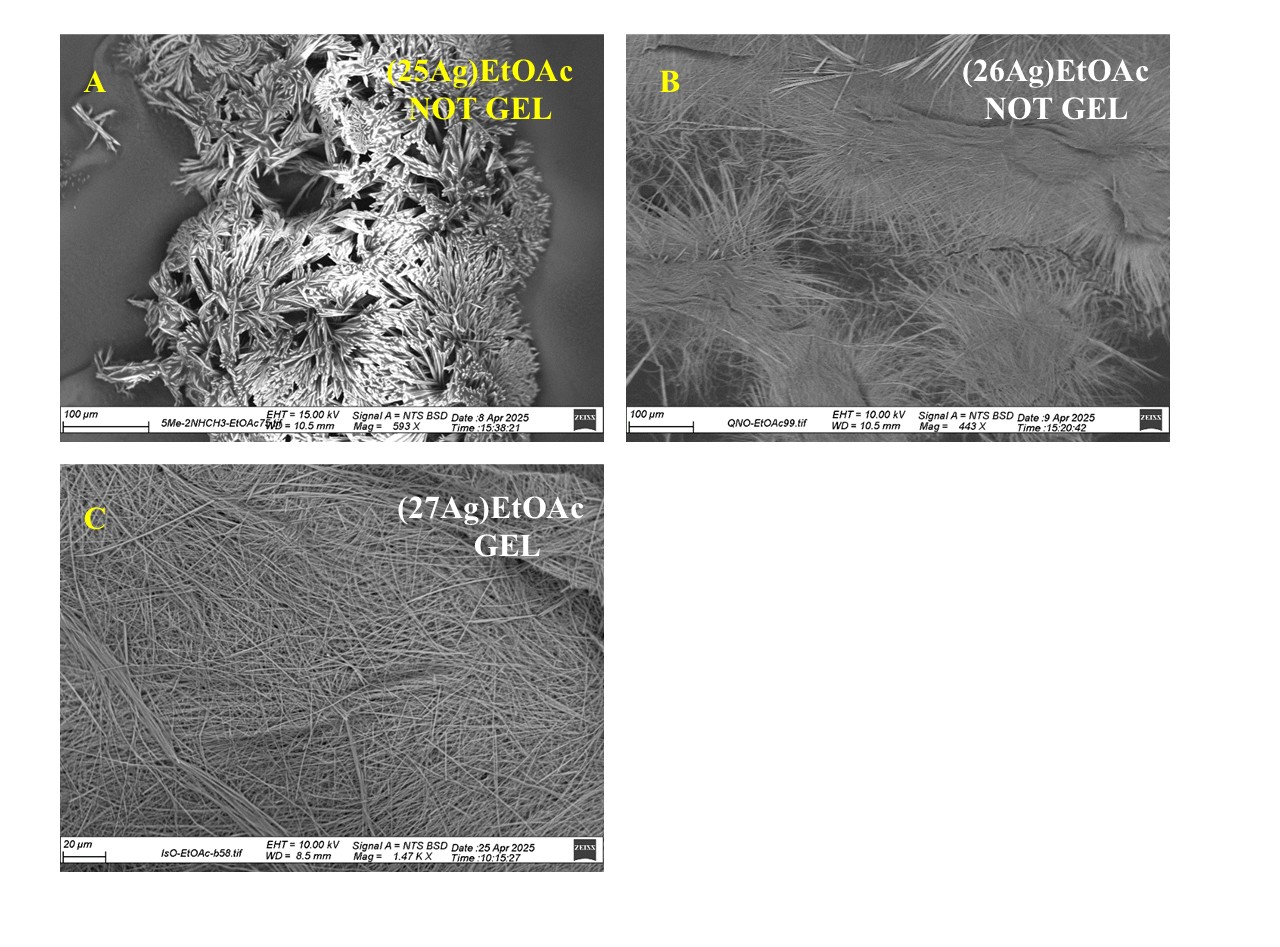
**

**Figure S23.** SEM photographs of A) **(25Ag)EtOAc**, B) **(26Ag)EtOAc**, and C) **(27Ag)EtOAc** at 0.25 w/v% of PyNO

**
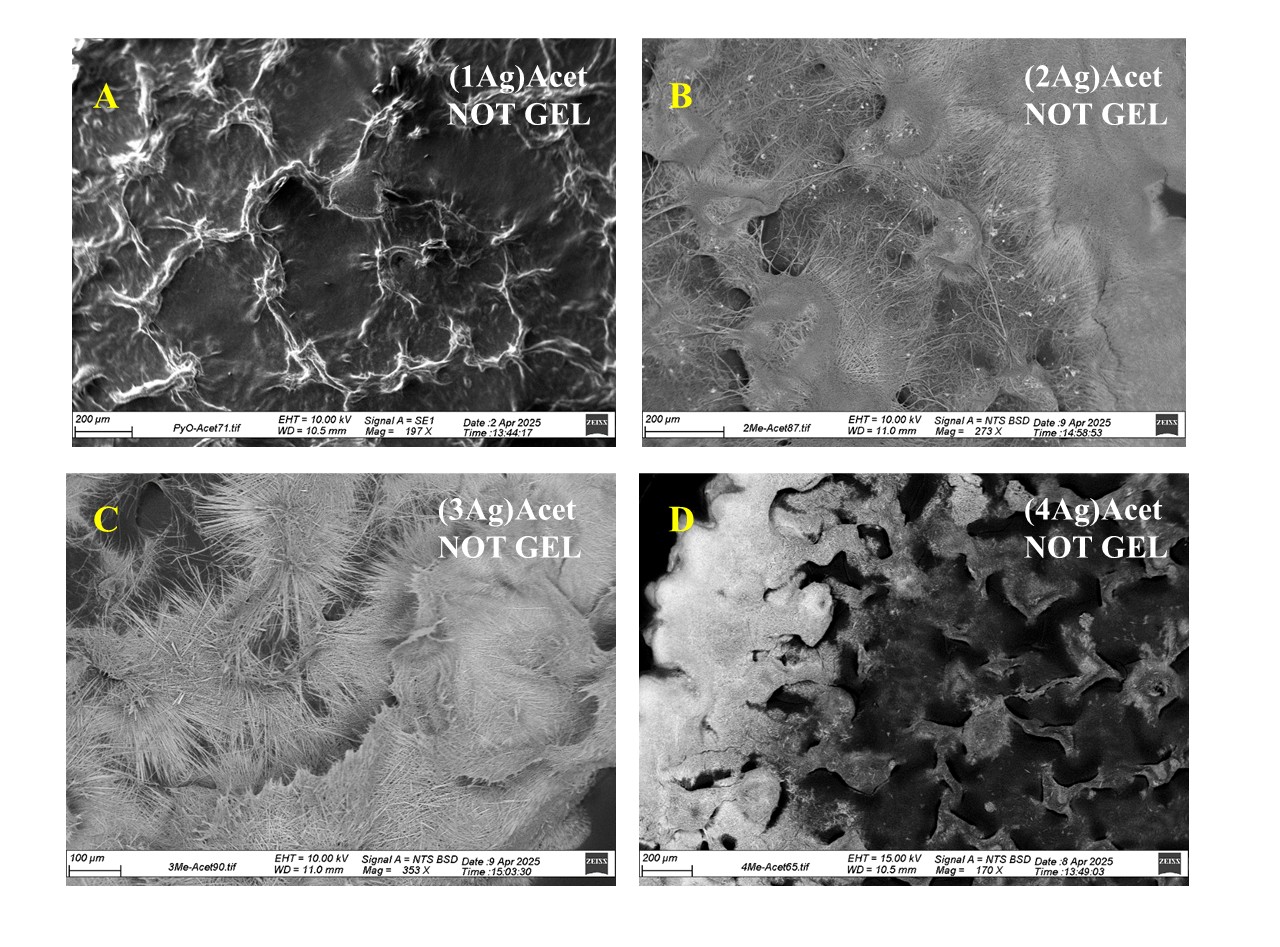
**

**Figure S24.** SEM photographs of A) **(1Ag)Acet**, B) **(2Ag)Acet**, C) **(3Ag)Acet**, and D) **(4Ag)Acet** at 0.25 w/v% of PyNO

**
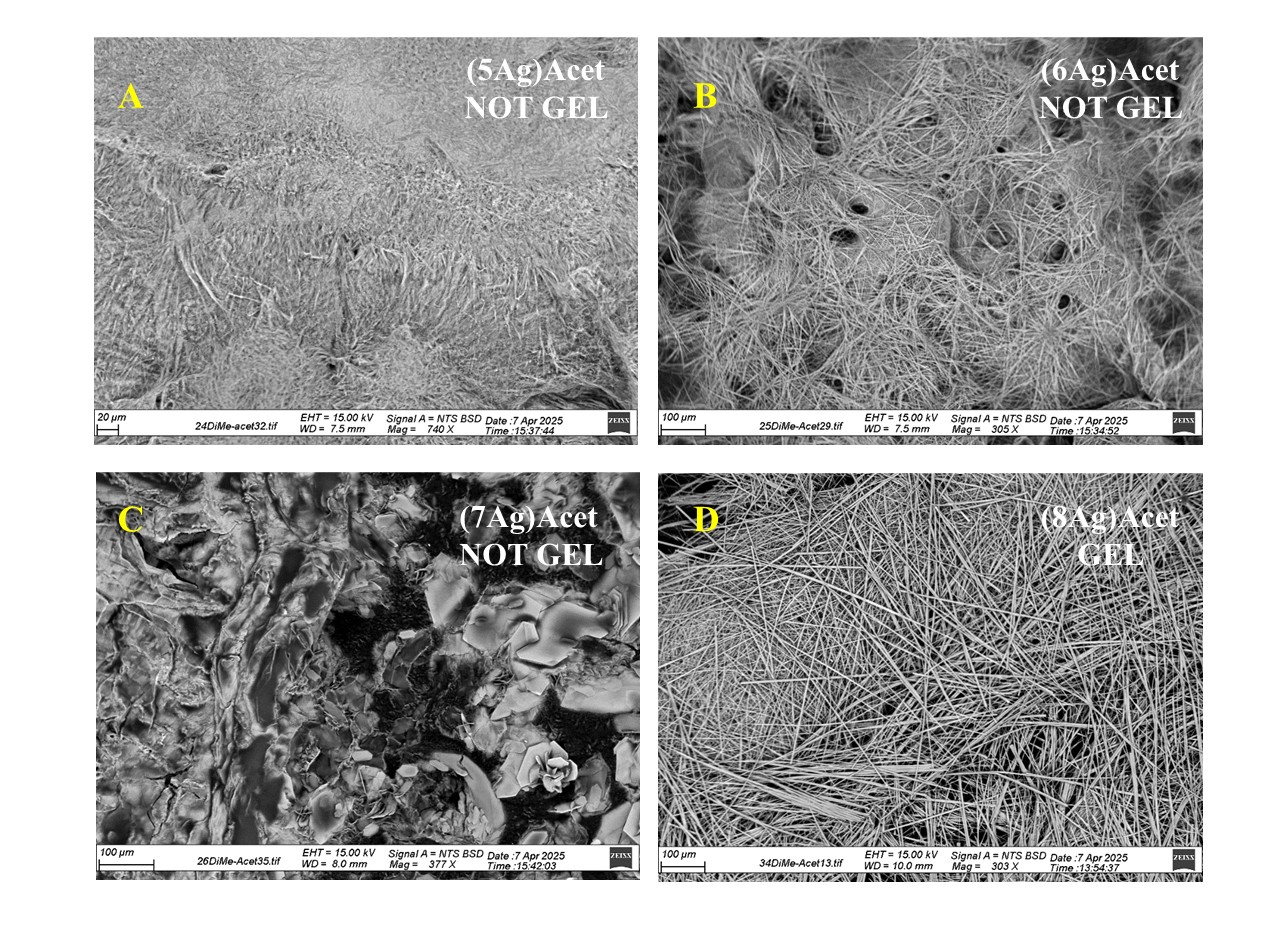
**

**Figure S25.** SEM photographs of A) **(5Ag)Acet**, B) **(6Ag)Acet**, C) **(7Ag)Acet**, and D) **(8Ag)Acet** at 0.25 w/v% of PyNO

**
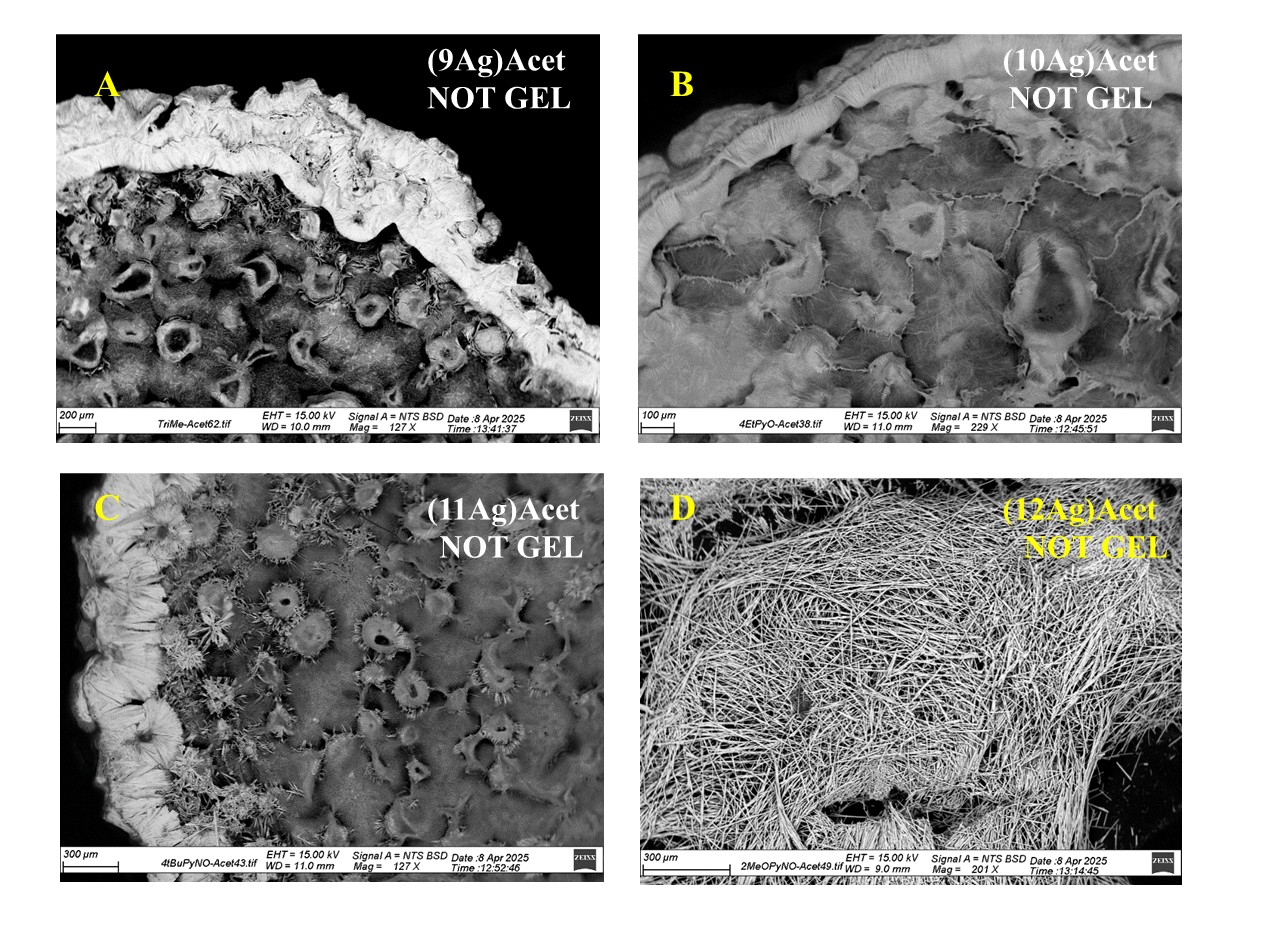
**

**Figure S26.** SEM photographs of A) **(9Ag)Acet**, B) **(10Ag)Acet**, C) **(11Ag)Acet**, and D) **(12Ag)Acet** at 0.25 w/v% of PyNO

**
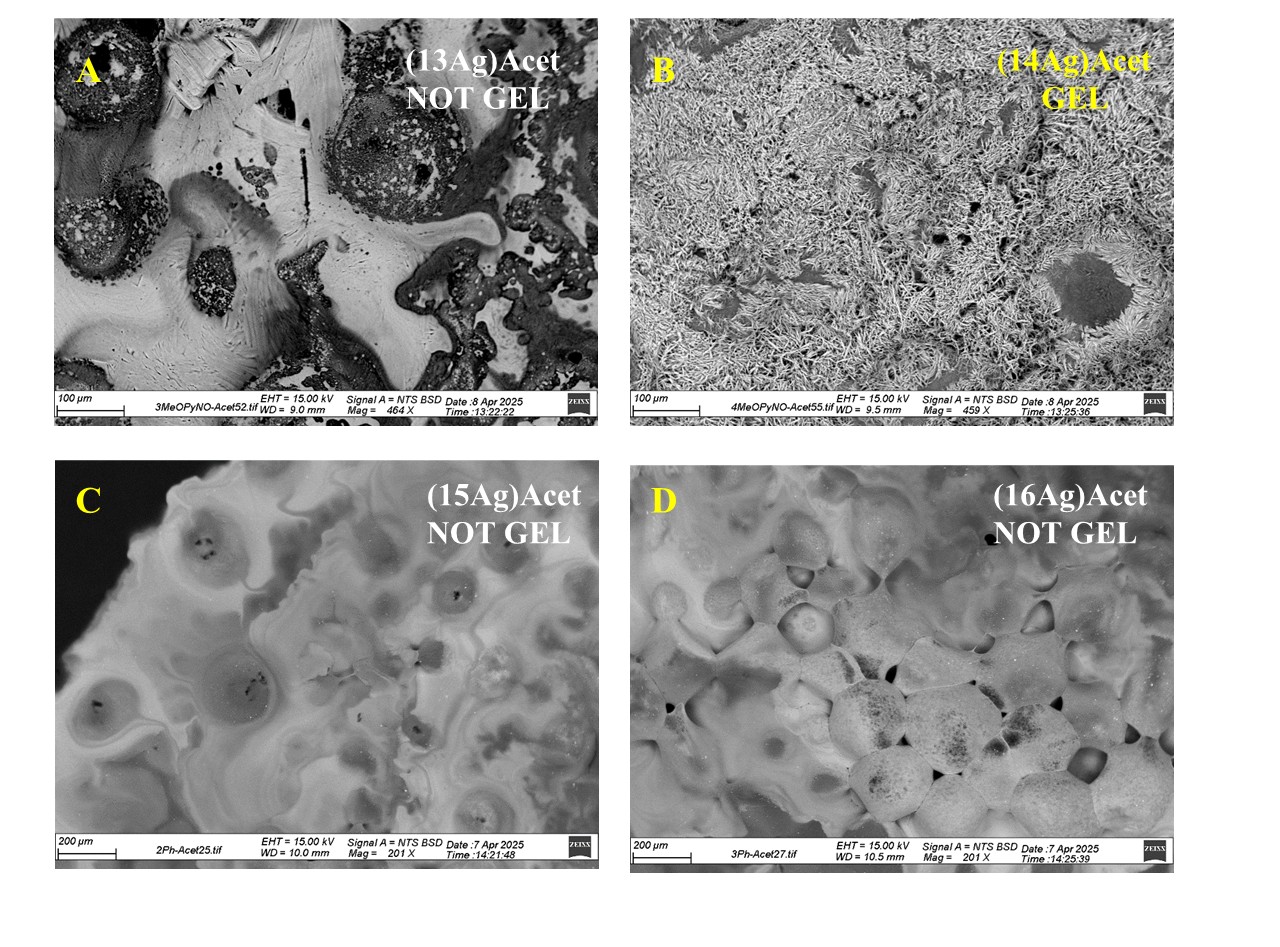
**

**Figure S27.** SEM photographs of A) **(13Ag)Acet**, B) **(14Ag)Acet**, C) **(15Ag)Acet**, and D) **(16Ag)Acet** at 0.25 w/v% of PyNO

**
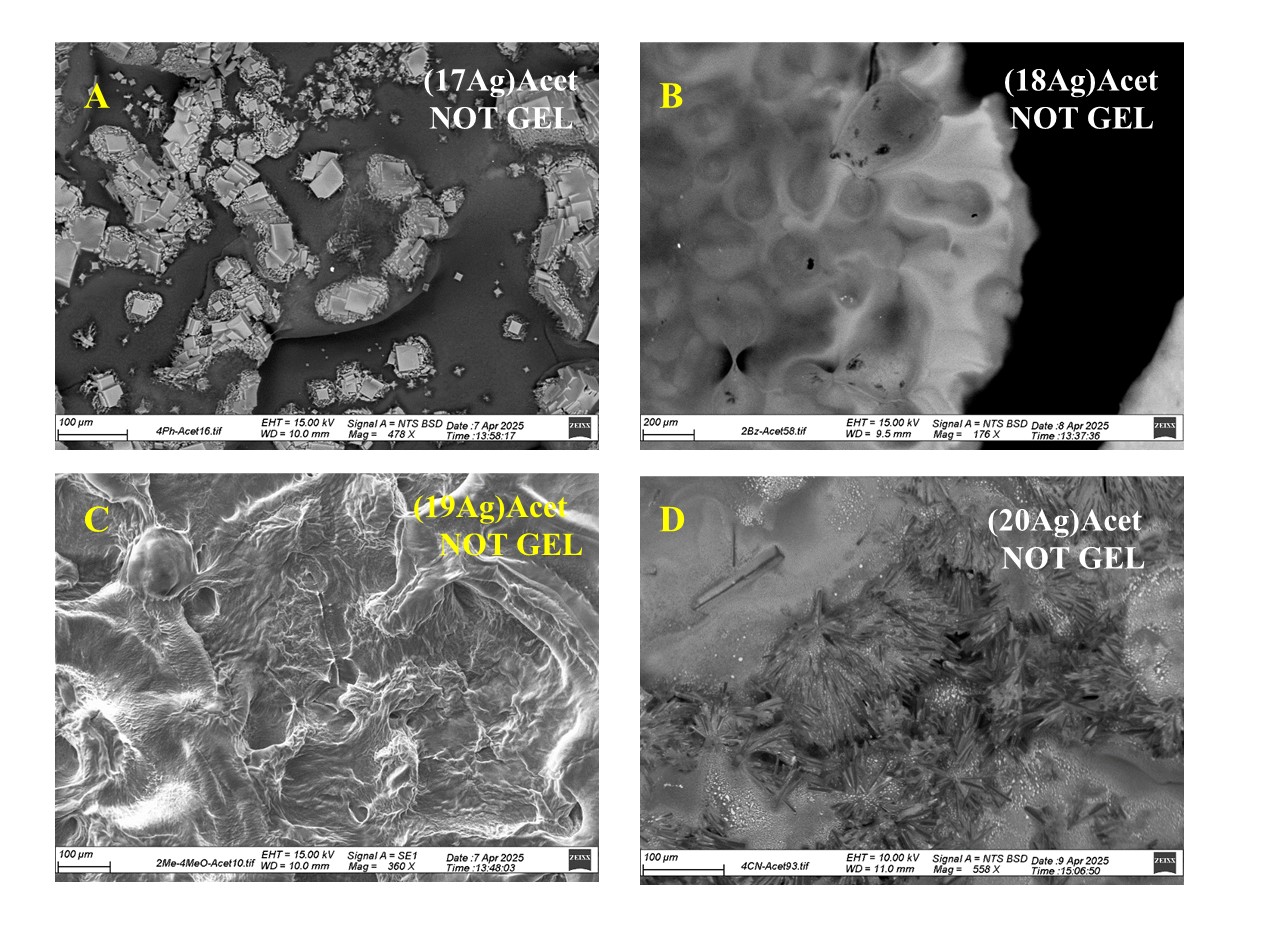
**

**Figure S28.** SEM photographs of A) **(17Ag)Acet**, B) **(18Ag)Acet**, C) **(19Ag)Acet**, and D) **(20Ag)Acet** at 0.25 w/v% of PyNO

**
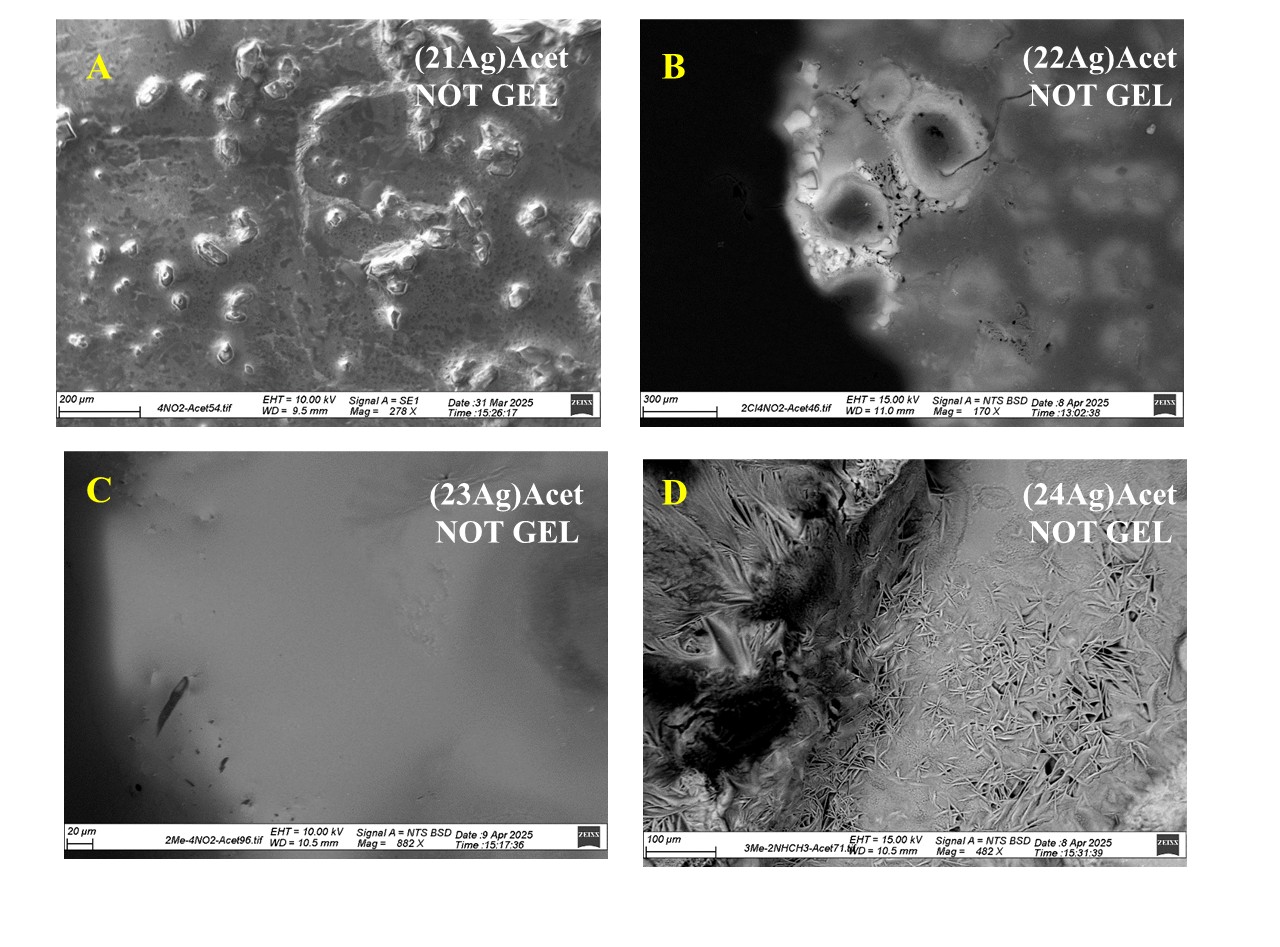
**

**Figure S29.** SEM photographs of A) **(21Ag)Acet**, B) **(22Ag)Acet**, C) **(23Ag)Acet**, and D) **(24Ag)Acet** at 0.25 w/v% of PyNO

**
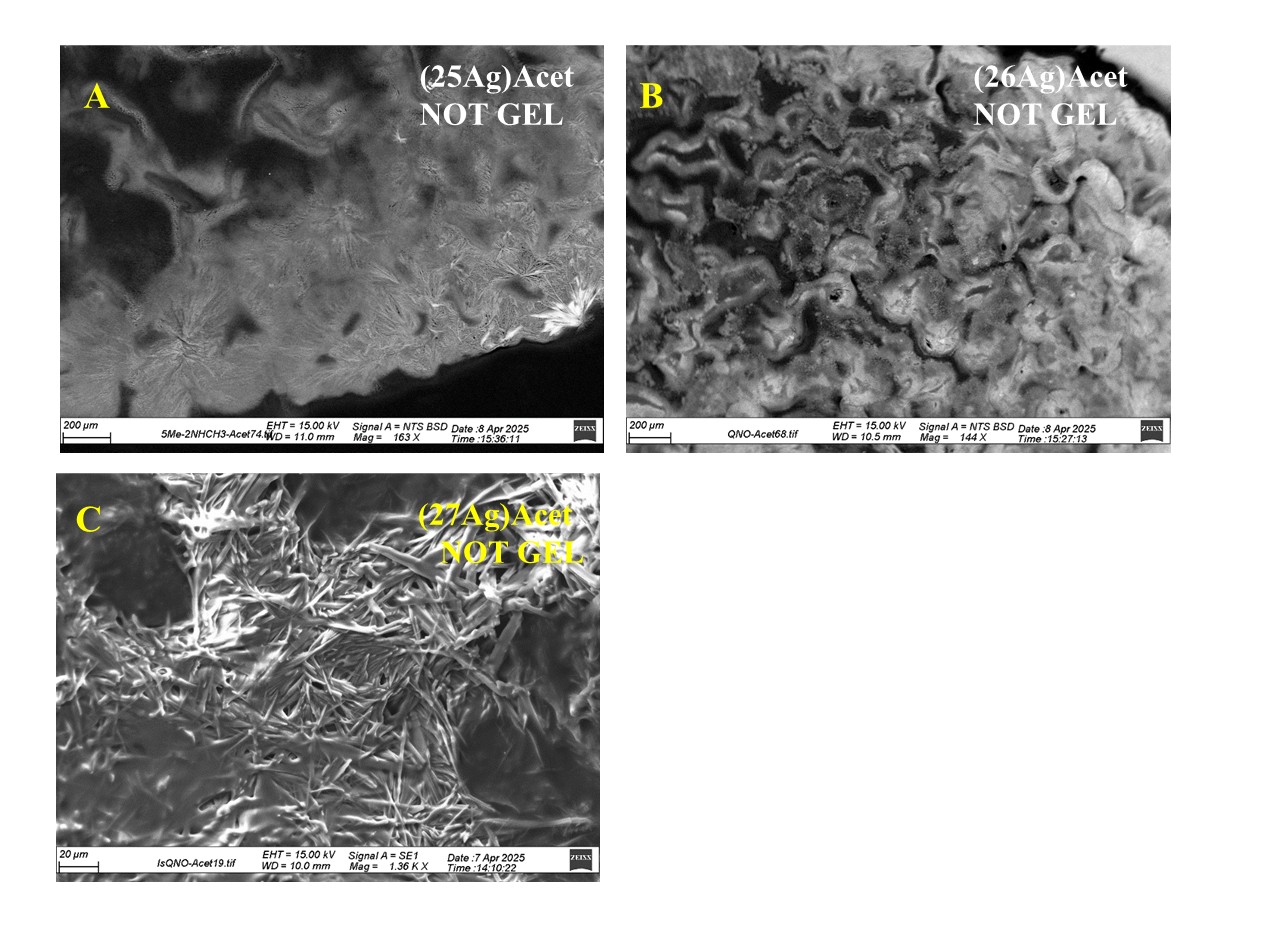
**

**Figure S30.** SEM photographs of A) **(25Ag)Acet**, B) **(26Ag)Acet**, and C) **(27Ag)Acet** at 0.25 w/v% of PyNO

**
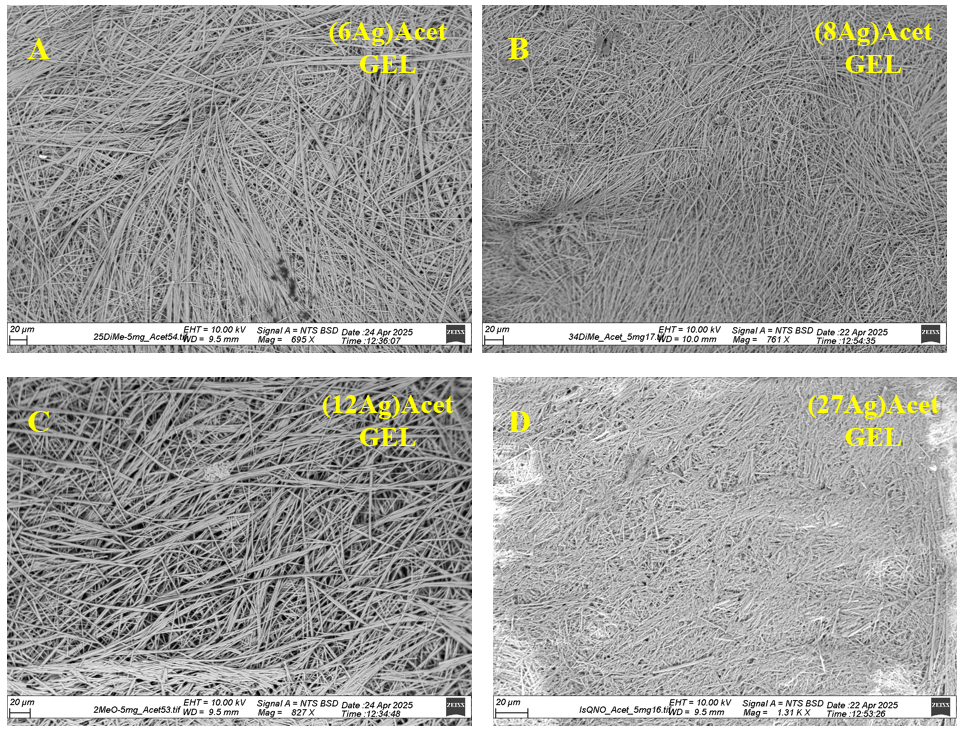
**

**Figure S31.** SEM photographs of A) **(6Ag)Acet** and B) **(8Ag)Acet**, C) **(12Ag)Acet** and D) **(27Ag)Acet** at 0.5 w/v% of PyNO.

**
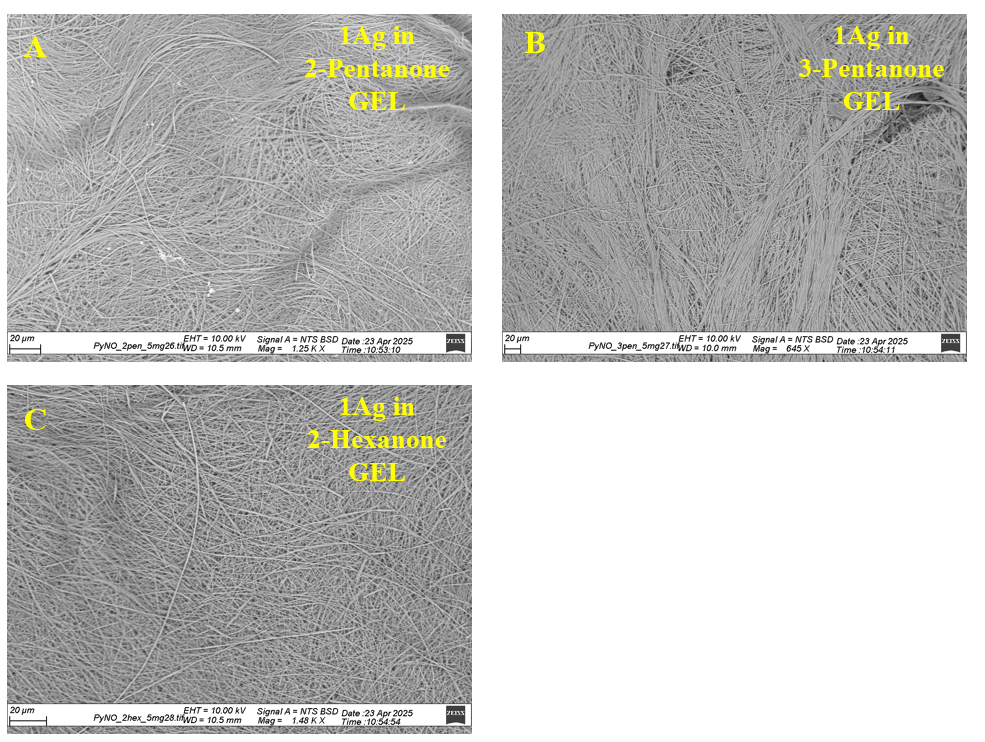
**

**Figure S32.** SEM photographs of A) **1Ag** in 2-pentanone, B) **1Ag** in 3-pentanone, C) **1Ag** in 2-hexanone at 0.5 w/v% of PyNO

**
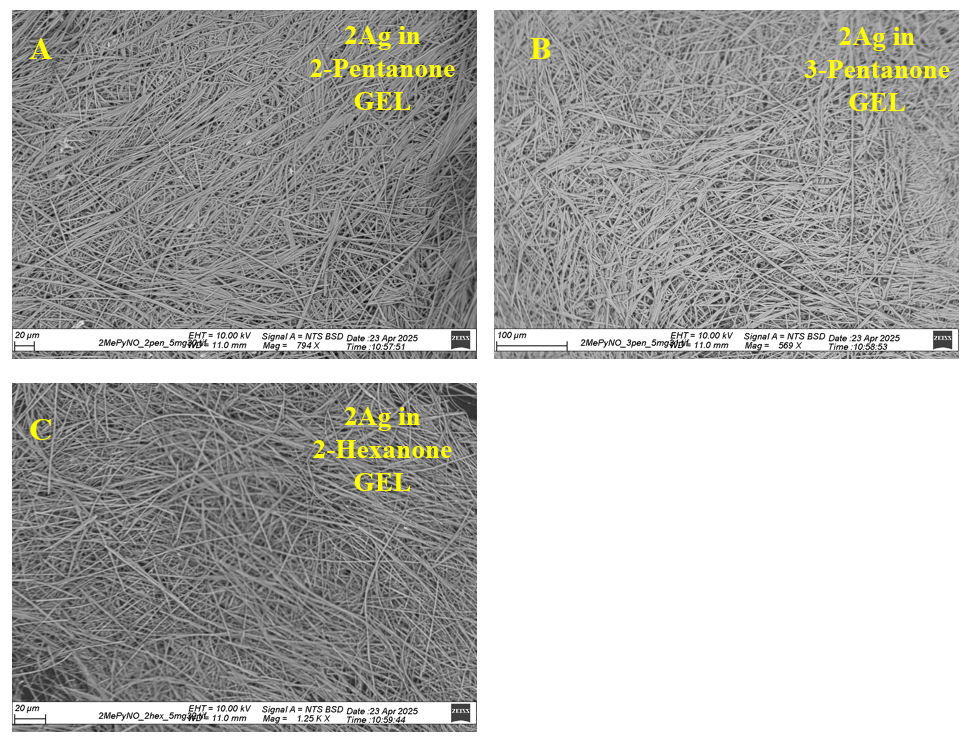
**

**Figure S33.** SEM photographs of A) **2Ag** in 2-pentanone, B) **2Ag** in 3-pentanone, C) **2Ag** in 2-hexanone at 0.5 w/v% of PyNO

**
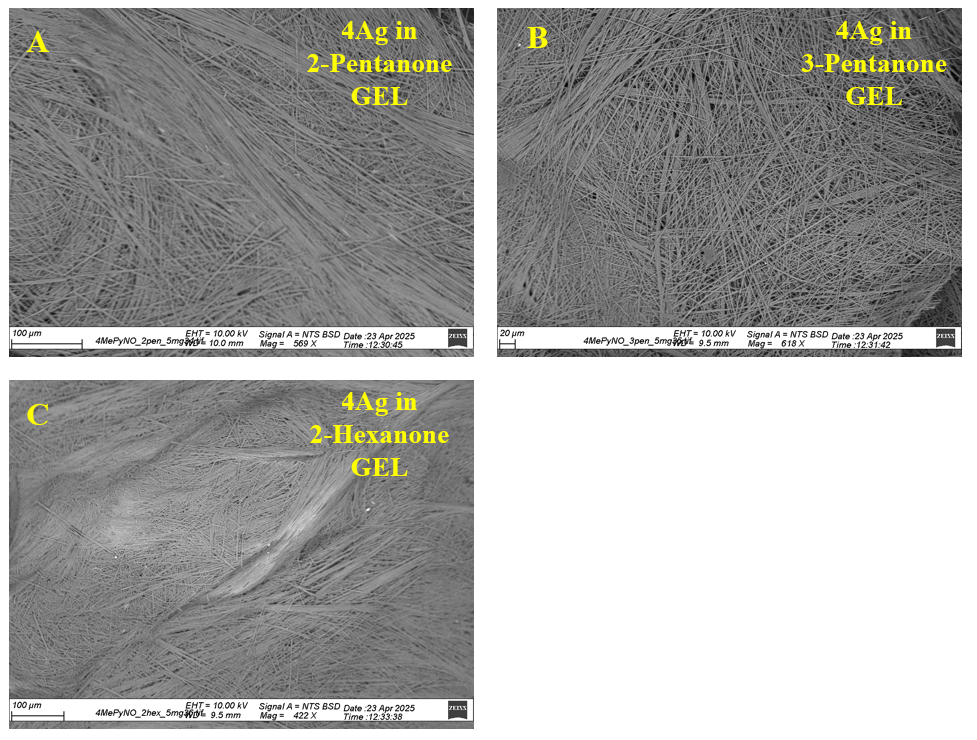
**

**Figure S34.** SEM photographs of A) **4Ag** in 2-pentanone, B) **4Ag** in 3-pentanone, C) **4Ag** in 2-hexanone at 0.5 w/v% of PyNO

**
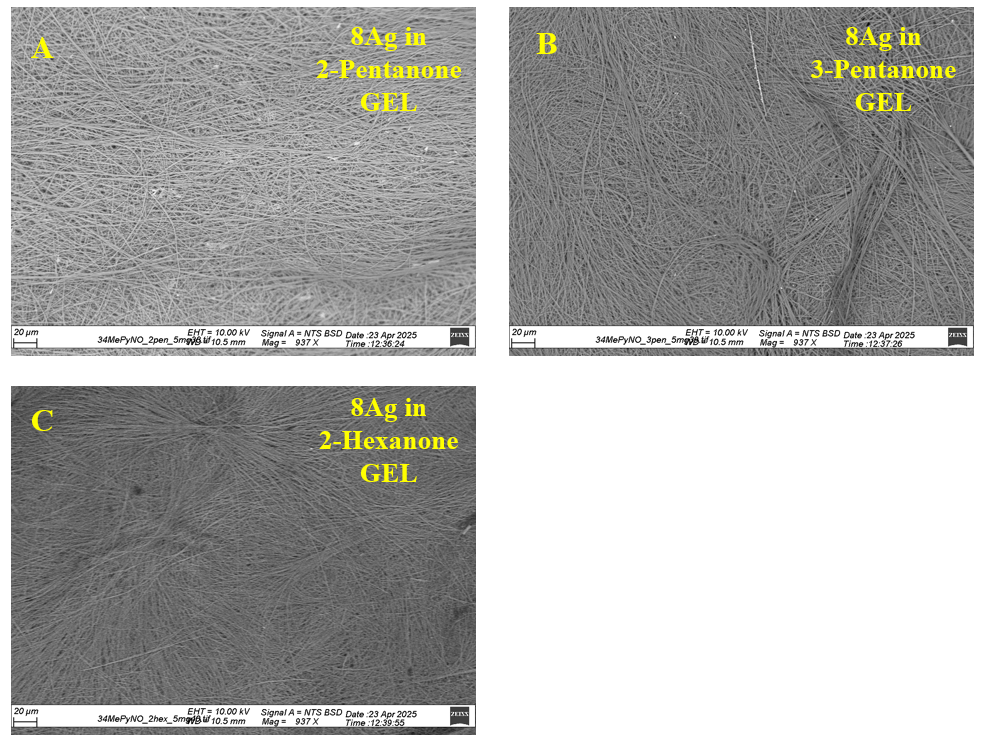
**

**Figure S35.** SEM photographs of A) **8Ag** in 2-pentanone, B) **8Ag** in 3-pentanone, C) **8Ag** in 2-hexanone at 0.5 w/v% of PyNO

**
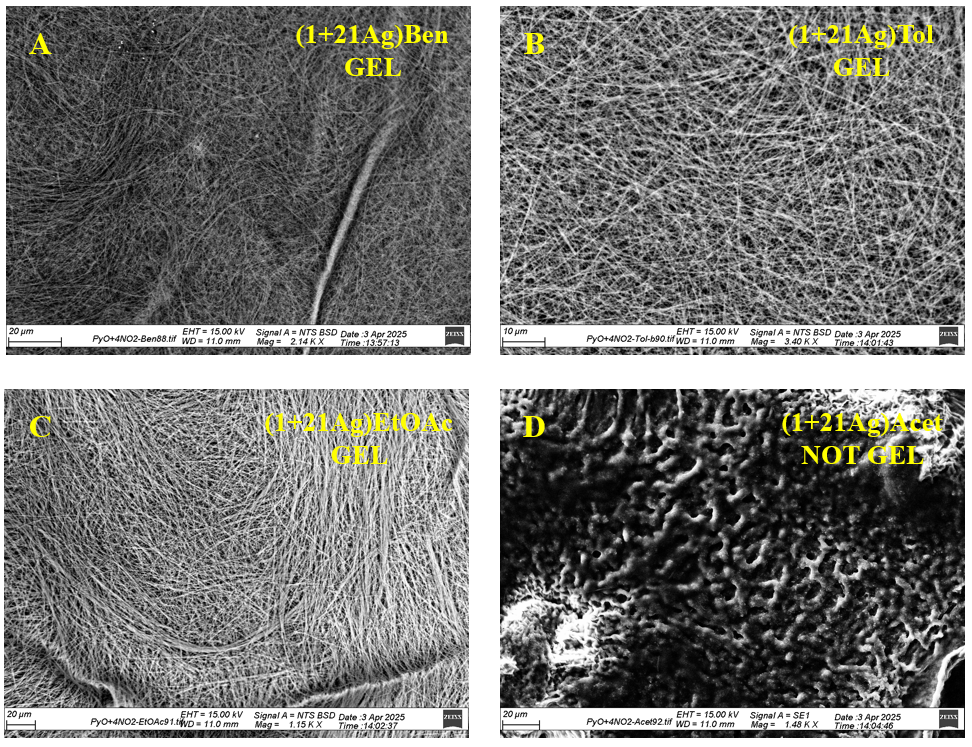
**

**Figure S36.** SEM photographs of A) **(1+21Ag)Ben**, B) **(1+21Ag)Tol**, C) **(1+21Ag)EtOAc,** and D) **(1+21Ag)Acet** at 0.25 w/v% of PyNO

**
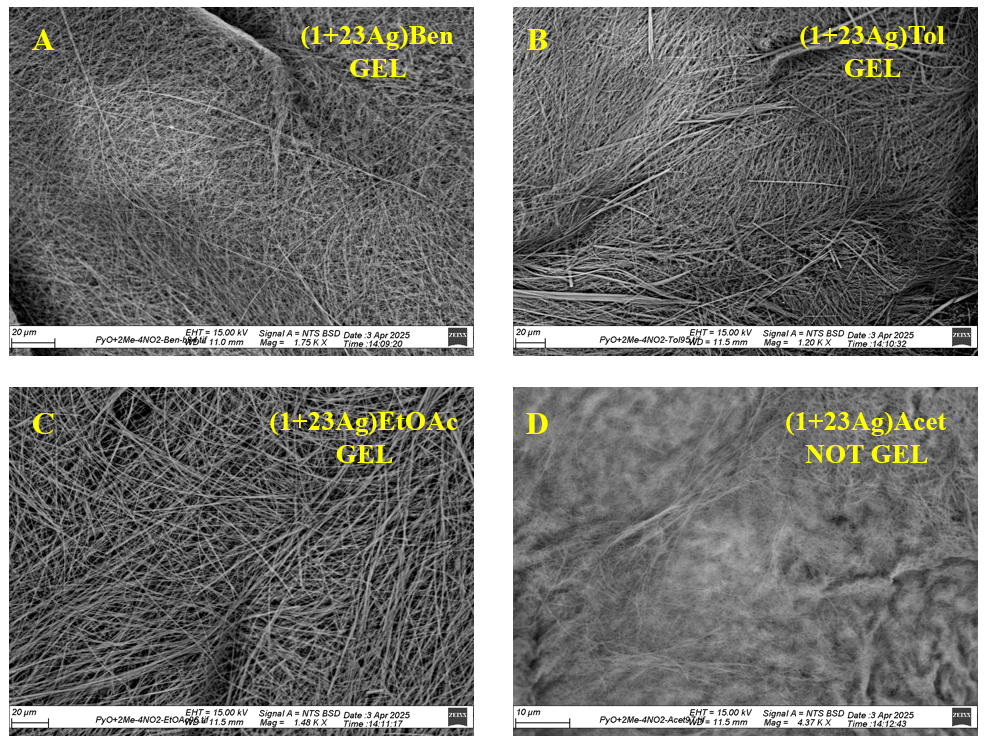
**

**Figure S37.** SEM photographs of A) **(1+23Ag)Ben**, B) **(1+23Ag)Tol**, C) **(1+23Ag)EtOAc,** and D) **(1+23Ag)Acet** at 0.25 w/v% of PyNO

**
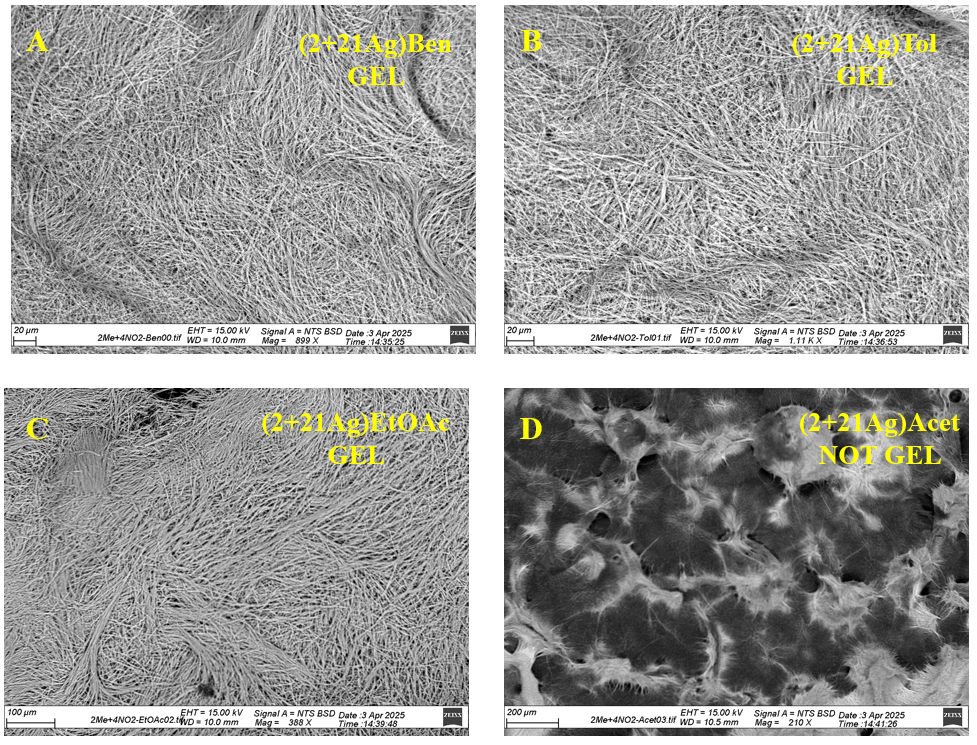
**

**Figure S38.** SEM photographs of A) **(2+21Ag)Ben**, B) **(2+21Ag)Tol**, C) **(2+21Ag)EtOAc,** and D) **(2+21Ag)Acet** at 0.25 w/v% of PyNO

**
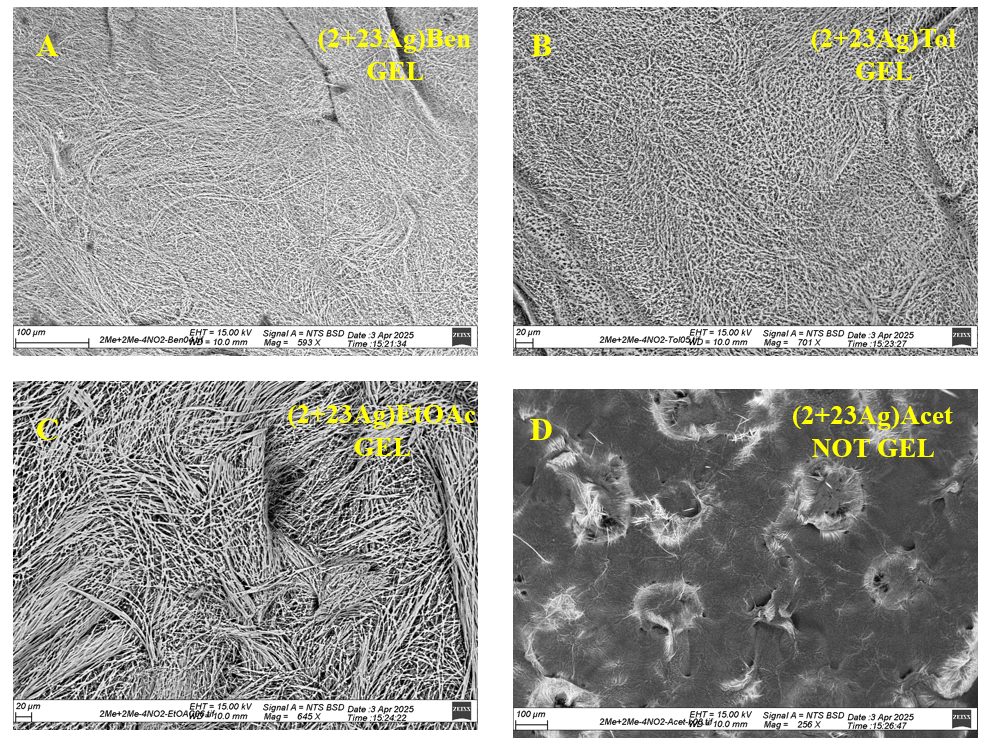
**

**Figure S39.** SEM photographs of A) **(2+23Ag)Ben**, B) **(2+23Ag)Tol**, C) **(2+23Ag)EtOAc,** and D) **(2+23Ag)Acet** at 0.25 w/v% of PyNO

**
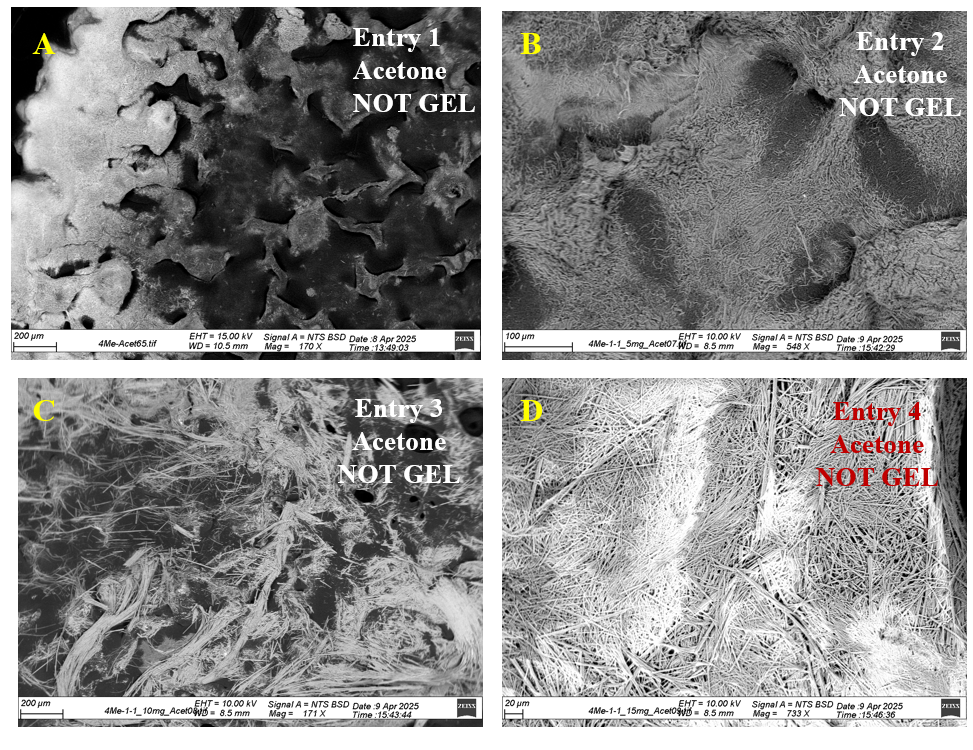
**

**Figure S40.** SEM photographs of ligand-to-AgTFA ratio study corresponding to Table 1 in the manuscript A) Entry 1 of Acetone, B) Entry 2 of Acetone, C) Entry 3 of Acetone, and D) Entry 4 of Acetone

**
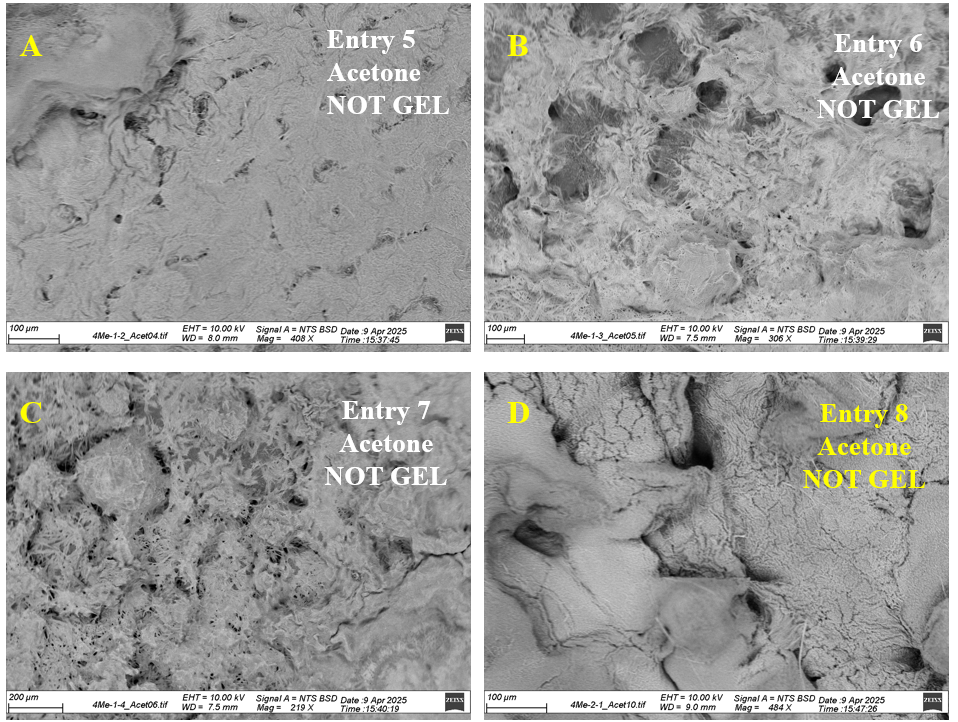
**

**Figure S41.** SEM photographs of ligand-to-AgTFA ratio study corresponding to Table 1 in manuscript A) Entry 5 of Acetone, B) Entry 6 of Acetone, C) Entry 7 of Acetone, and D) Entry 8 of Acetone

**
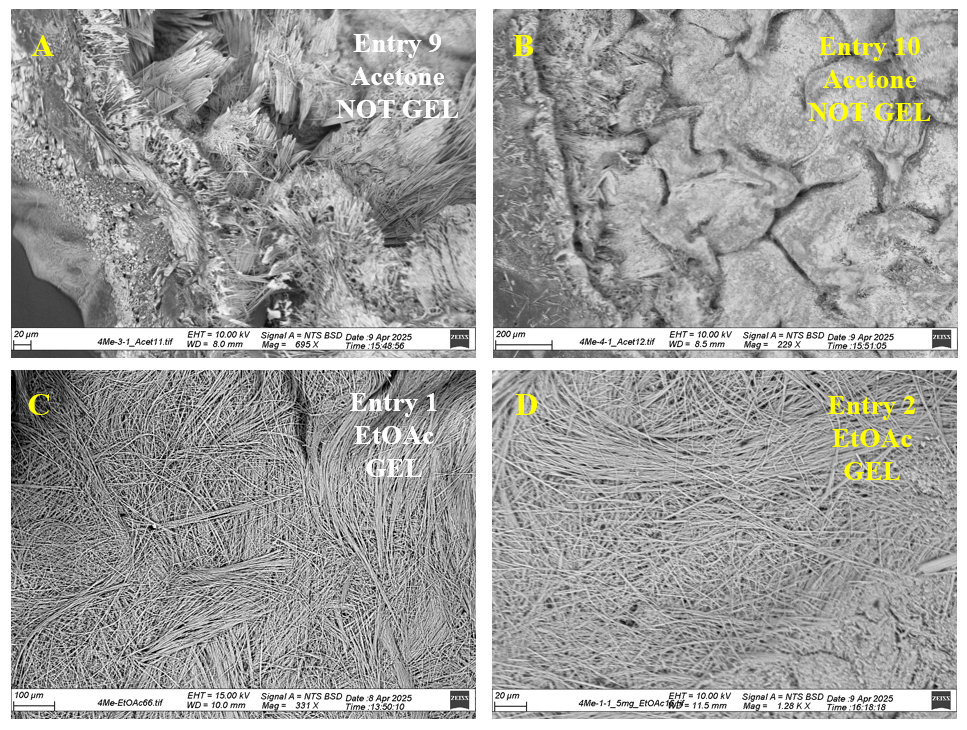
**

**Figure S42.** SEM photographs of ligand-to-AgTFA ratio study corresponding to Table 1 in the manuscript A) Entry 9 of Acetone, B) Entry 10 of Acetone, C) Entry 1 of EtOAc, and D) Entry 8 of EtOAc.

**
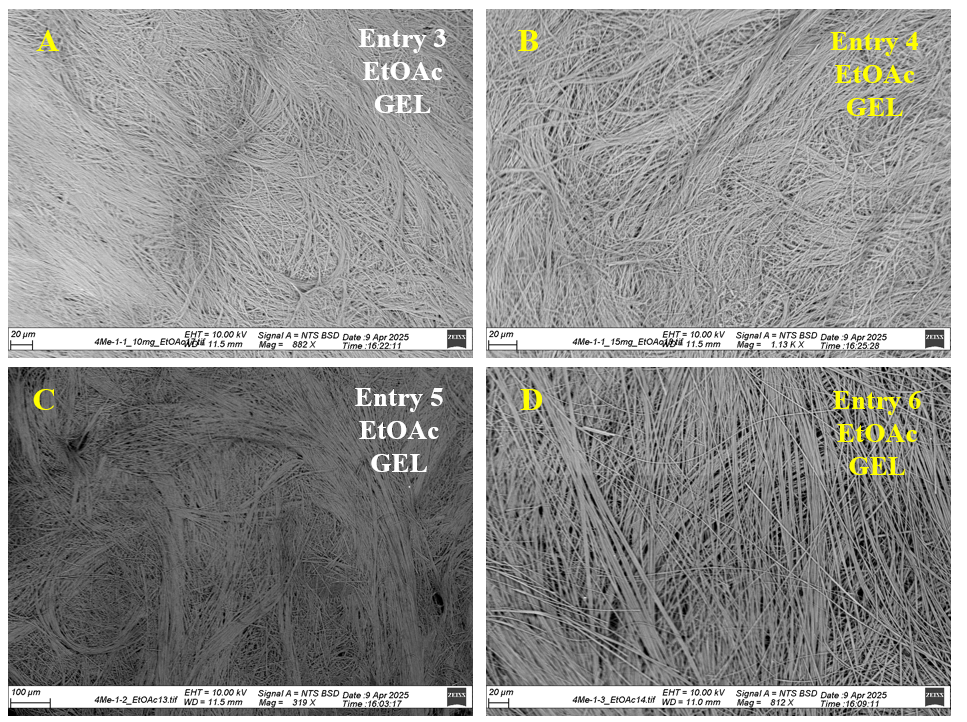
**

**Figure S43.** SEM photographs of ligand-to-AgTFA ratio study corresponding to Table 1 in the manuscript A) Entry 3 of EtOAc, B) Entry 4 of EtOAc, C) Entry 5 of EtOAc, and D) Entry 6 of EtOAc.

**
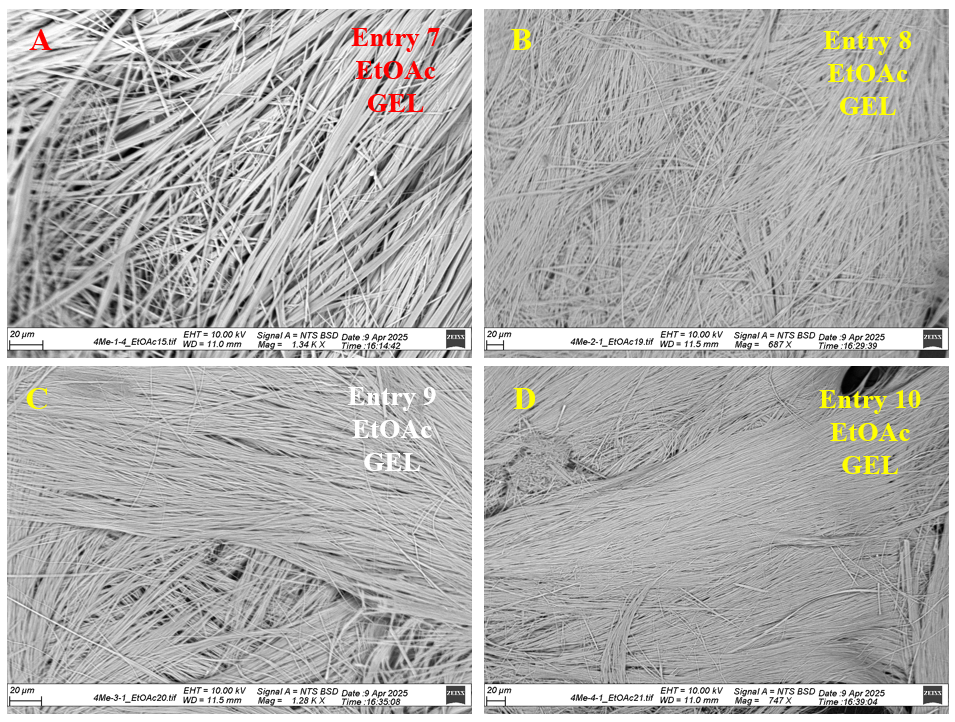
**

**Figure S44.** SEM photographs of ligand-to-AgTFA ratio study corresponding to Table 1 in the manuscript A) Entry 7 of EtOAc, B) Entry 8 of EtOAc, C) Entry 9 of EtOAc, and D) Entry 10 of EtOAc.

**4. Rheology**


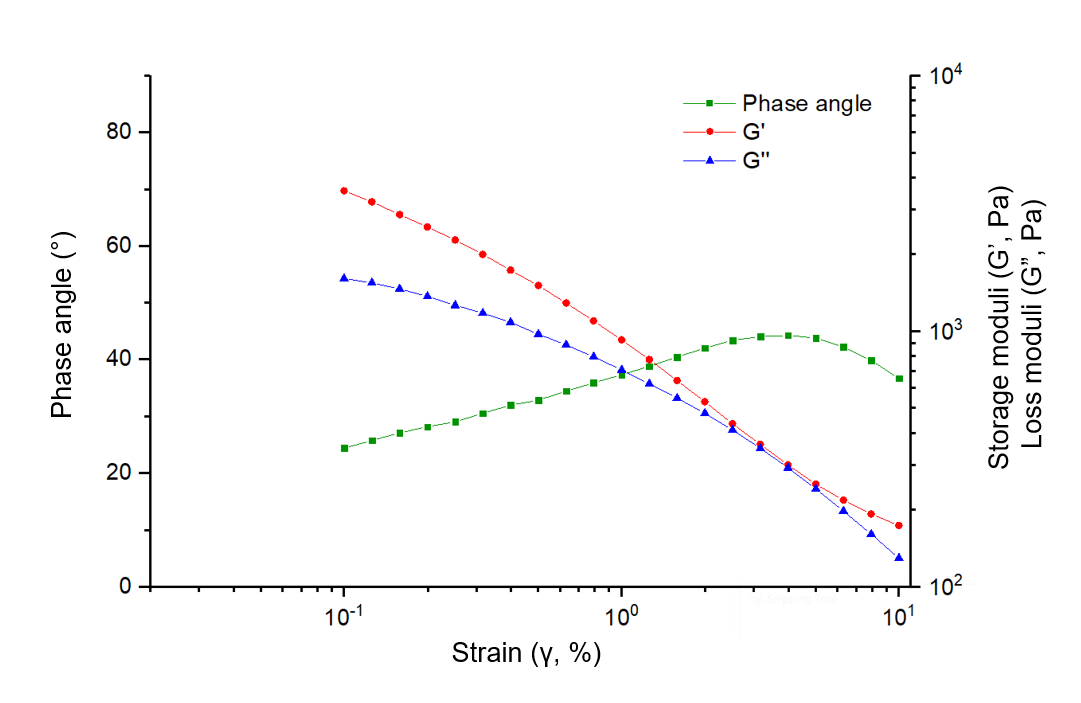


**Figure S45.** Strain-sweep analysis of **(1Ag)Tol** with 0.25 w/v% of PyNO


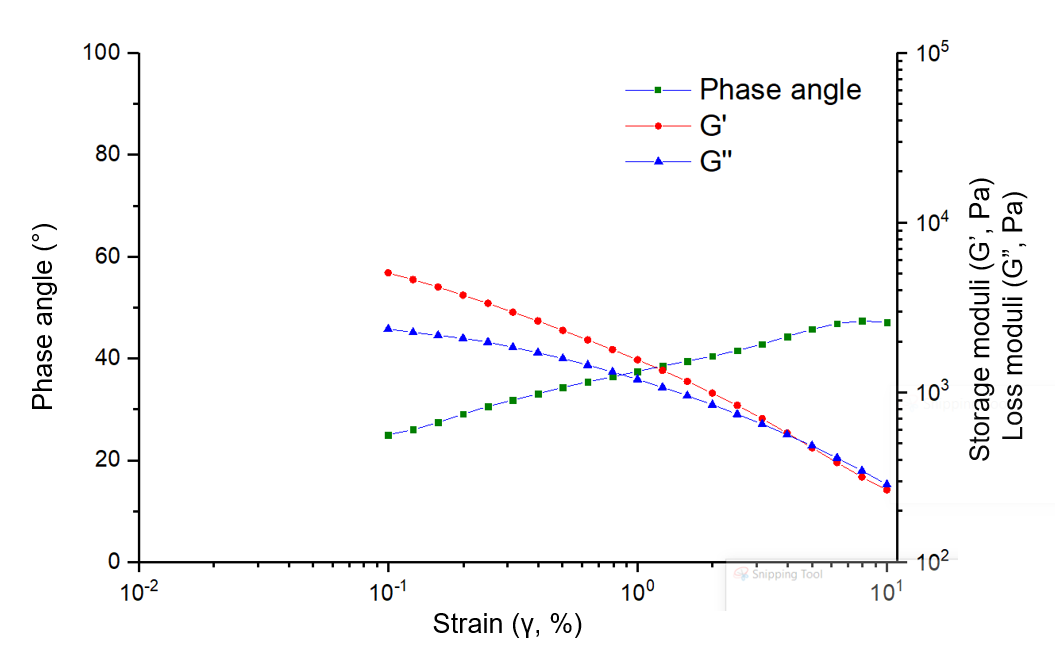


**Figure S46.** Strain-sweep analysis of **(2Ag)Tol** with 0.25 w/v% of PyNO


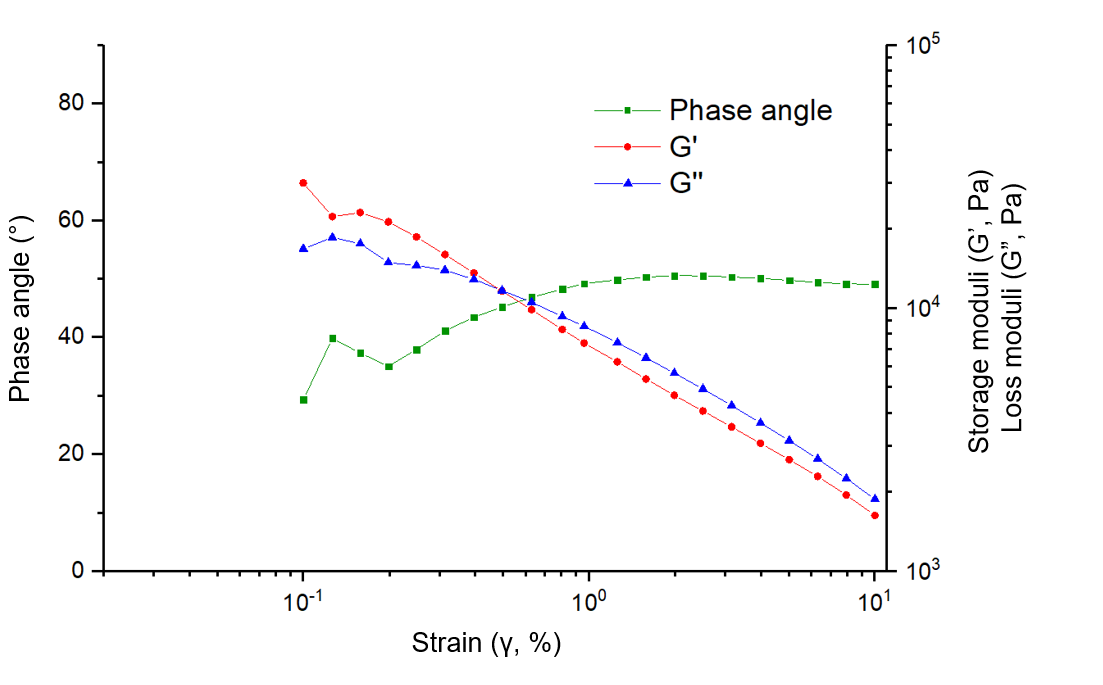


**Figure S47.** Strain-sweep analysis of **(4Ag)Tol** with 0.25 w/v% of PyNO


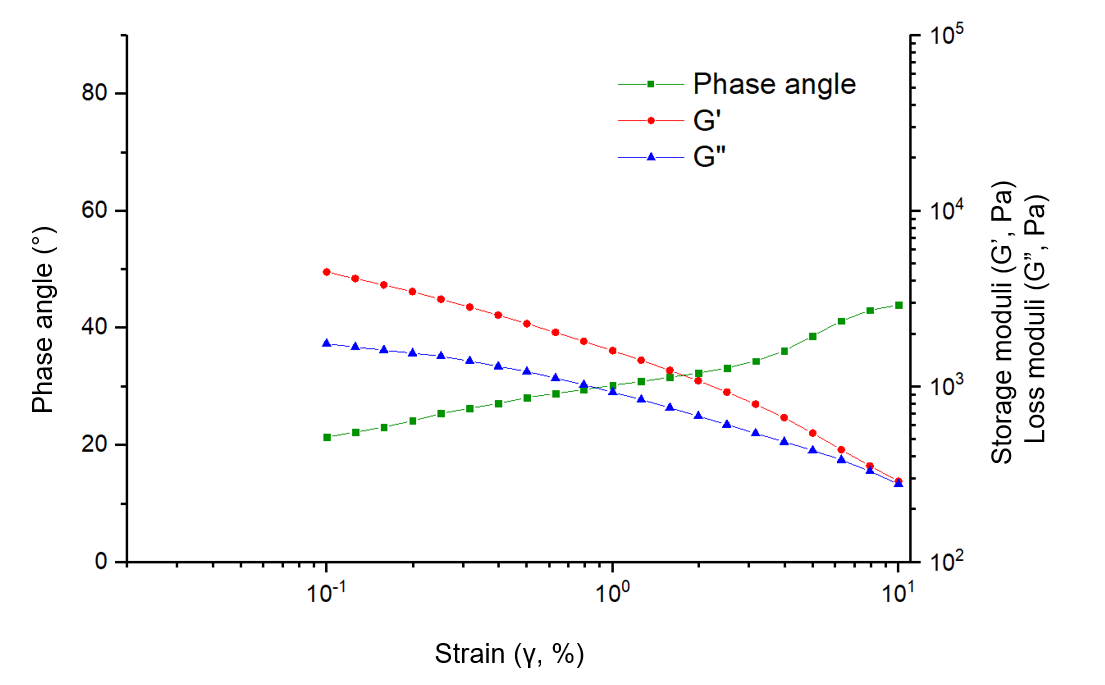


**Figure S48.** Strain-sweep analysis of **(8Ag)Tol** with 0.25 w/v% of PyNO


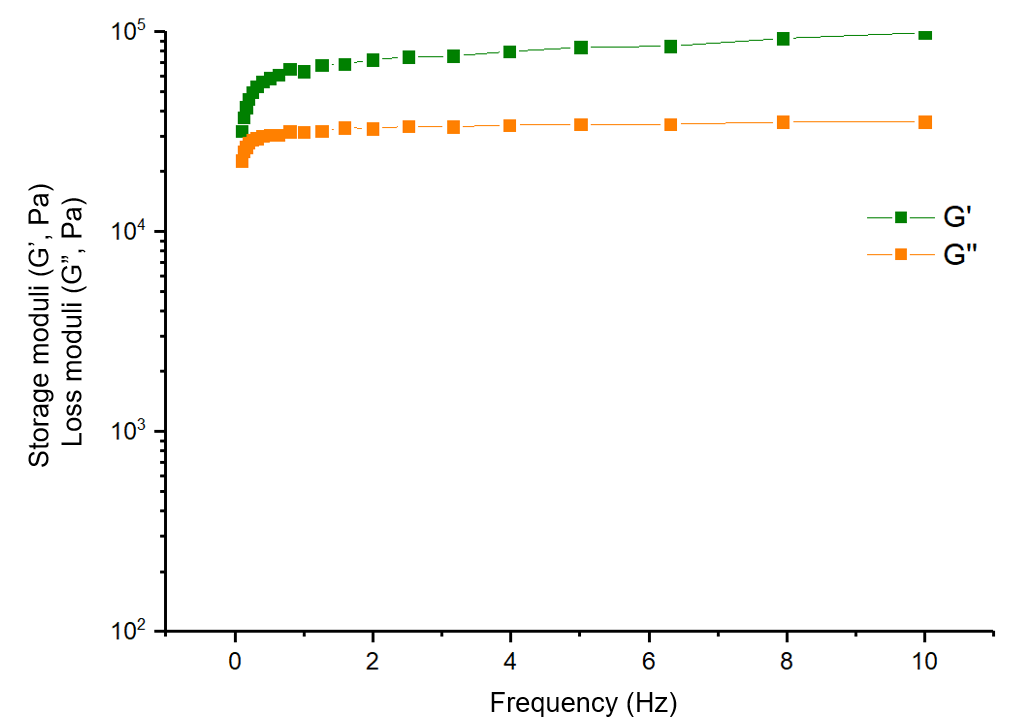


**Figure S49.** Frequency-sweep analysis of **(1Ag)Tol** with 0.25 w/v% of PyNO


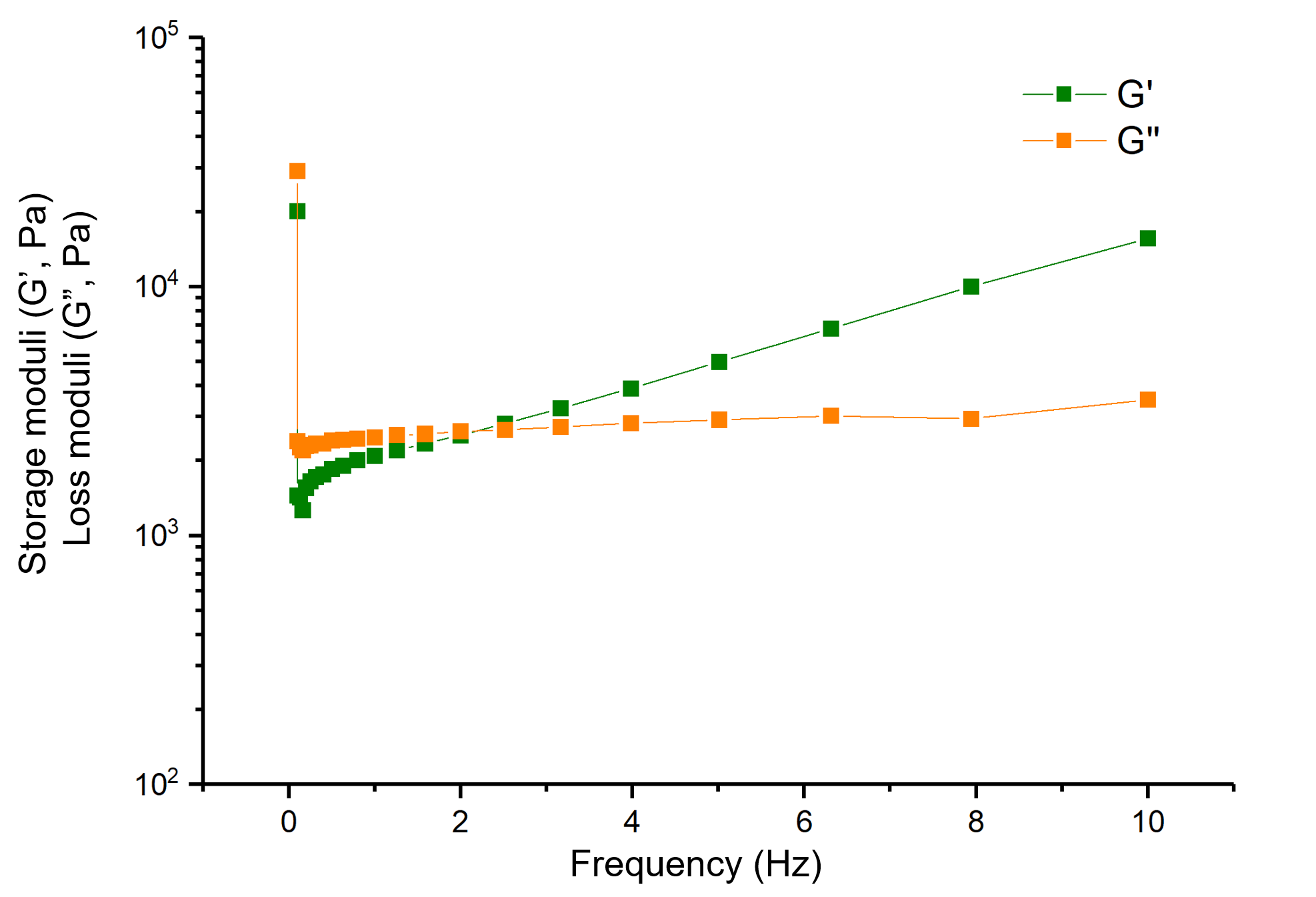


**Figure S50.** Frequency-sweep analysis of **(2Ag)Tol** with 0.25 w/v% of PyNO


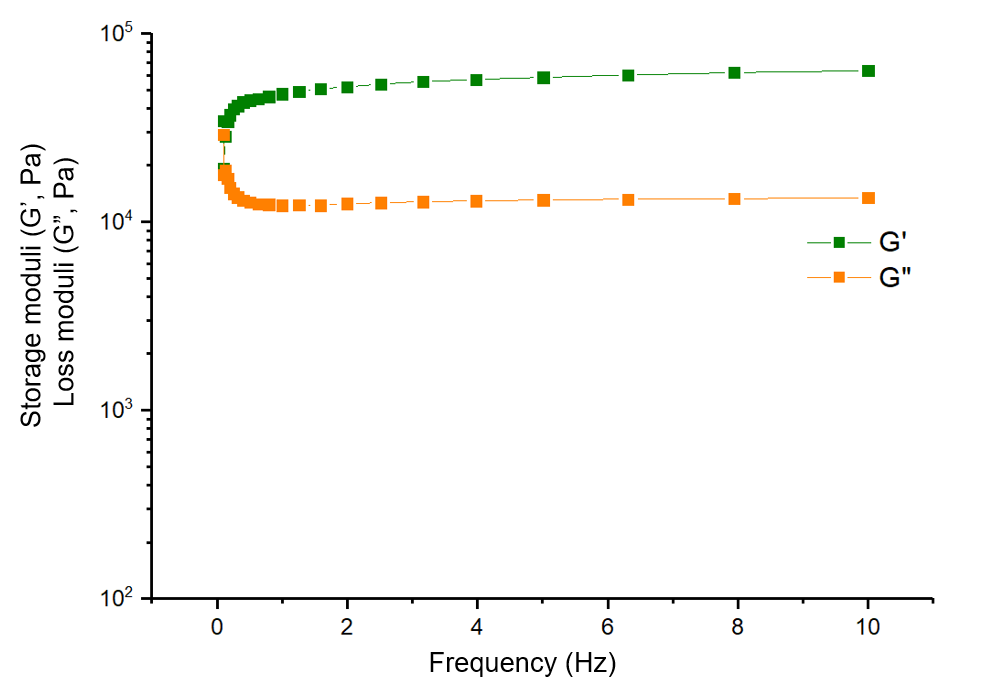


**Figure S51.** Frequency-sweep analysis of **(4Ag)Tol** with 0.25 w/v% of PyNO


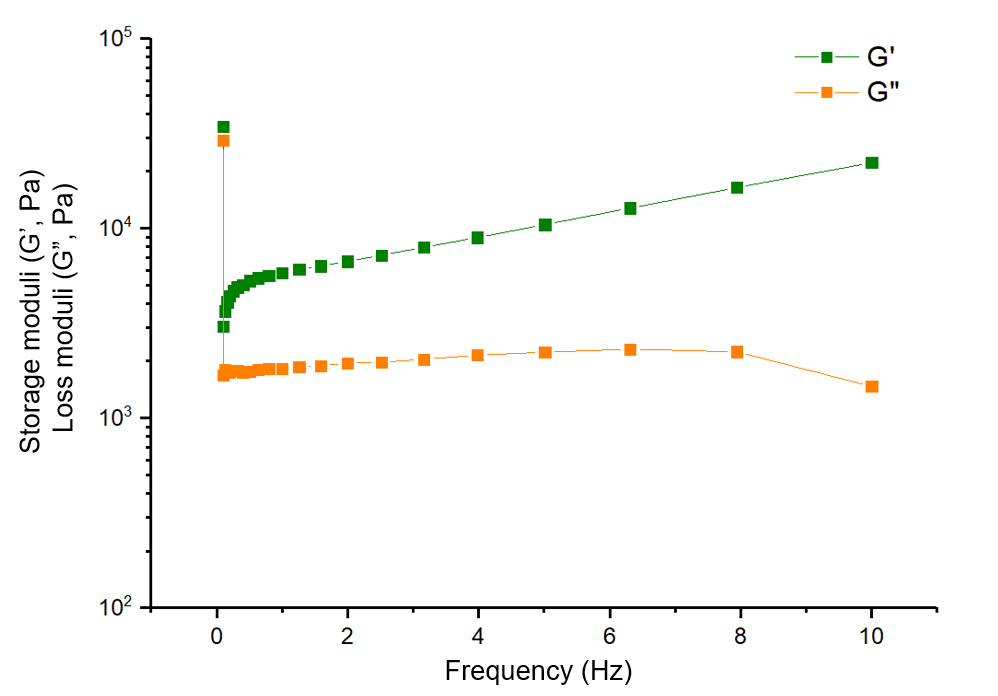


**Figure S52.** Frequency-sweep analysis of **(8Ag)Tol** with 0.25 w/v% of PyNO


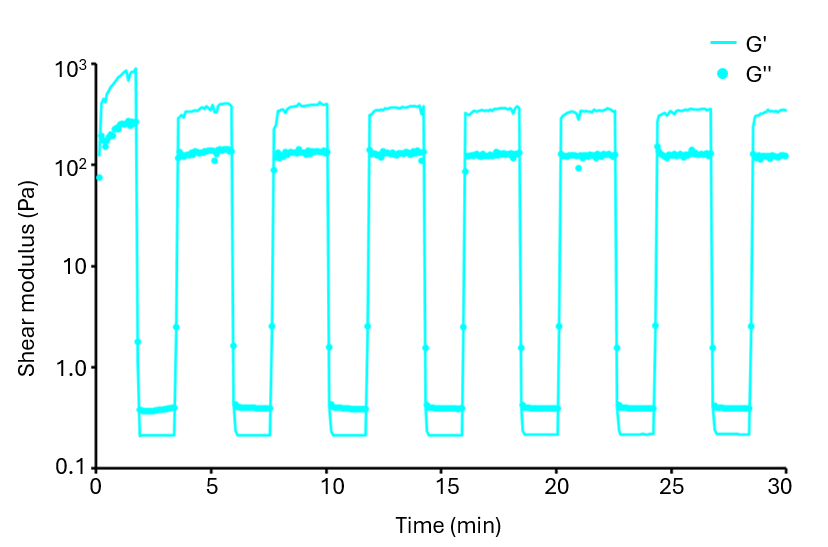


**Figure S53.** Self-healing test of **(2Ag)Tol** by step-strain analysis at 600% and 1% strain in 7 cycles.

**
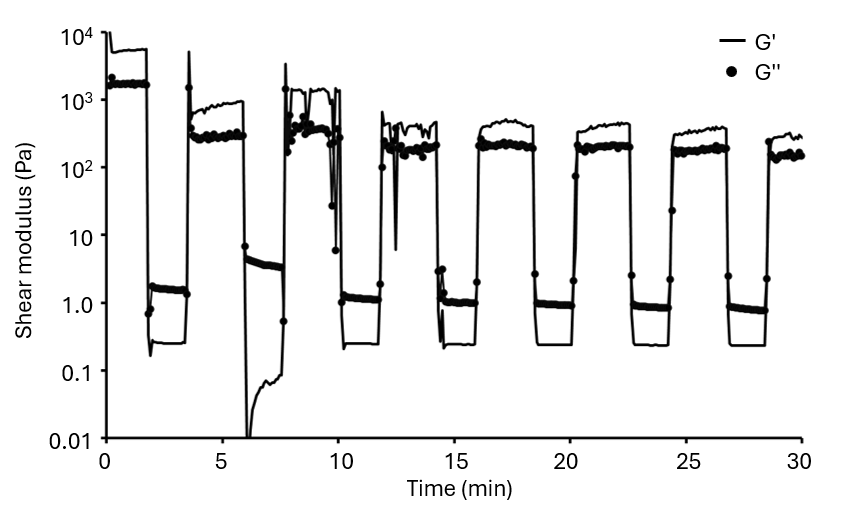
**

**Figure S54.** Self-healing test of **(4Ag)Tol** by step-strain analysis at 600% and 1% strain in 7 cycles.

**
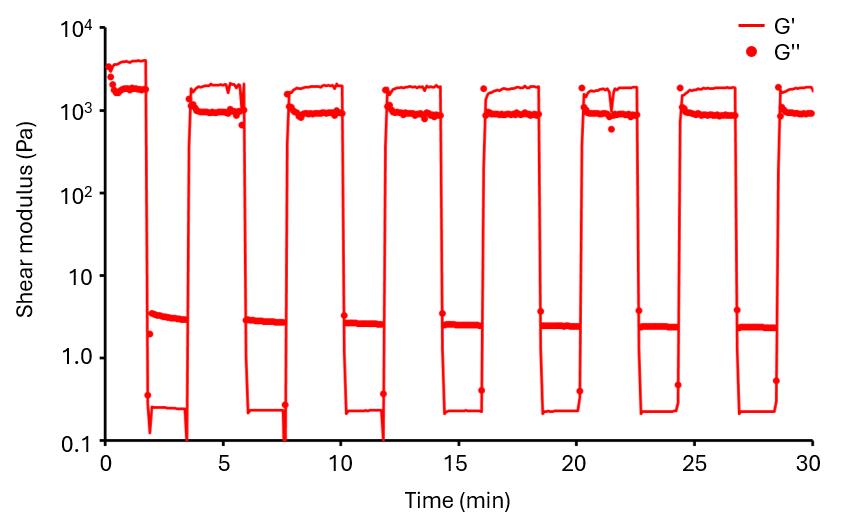
**

**Figure S55.** Self-healing test of **(8Ag)Tol** by step-strain analysis at 600% and 1% strain in 7 cycles.

**5. X-ray Crystallography**


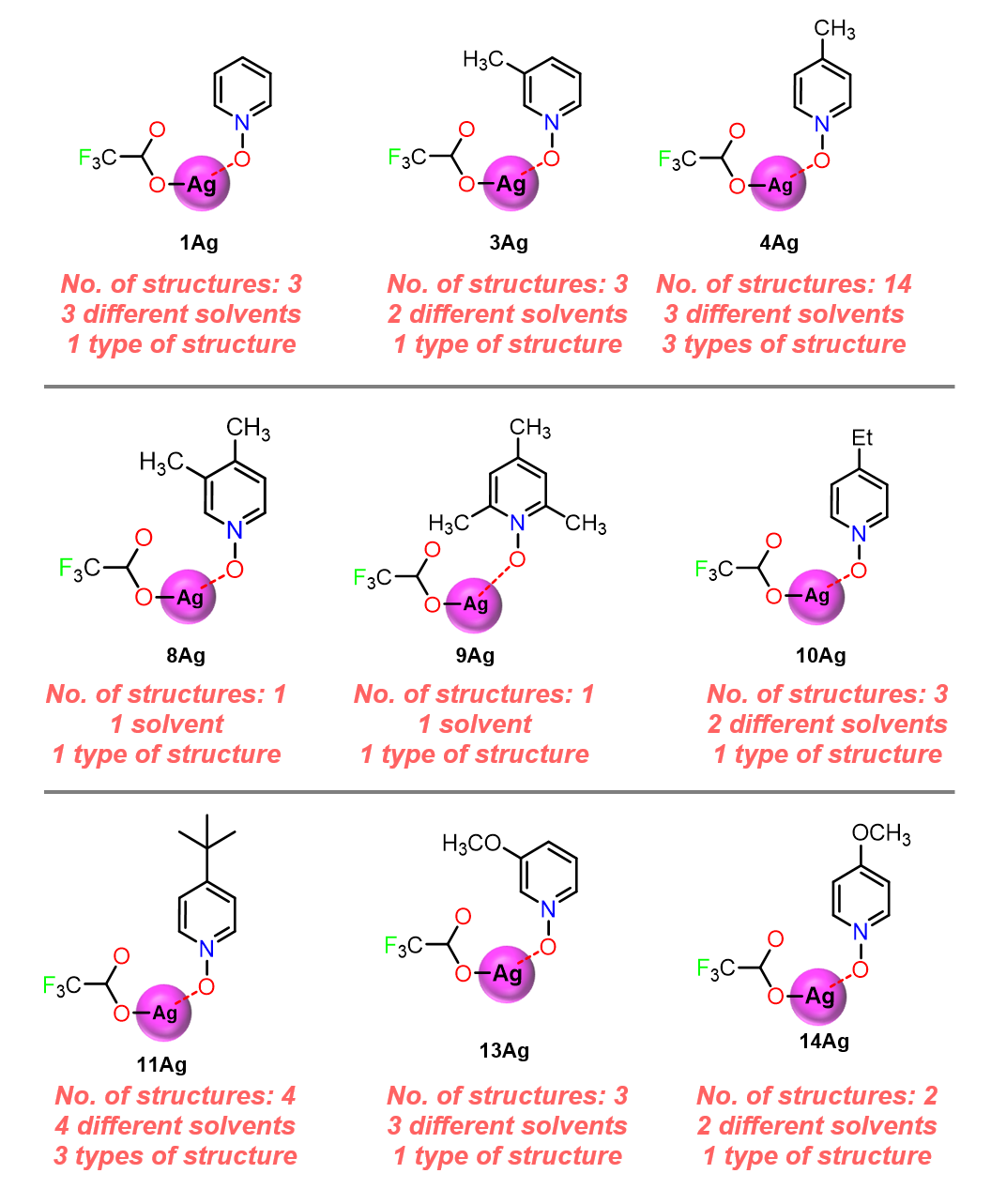


**Figure S56**. A list of PyNO-AgTFA complexes showing number of structures obtained from different solvents.


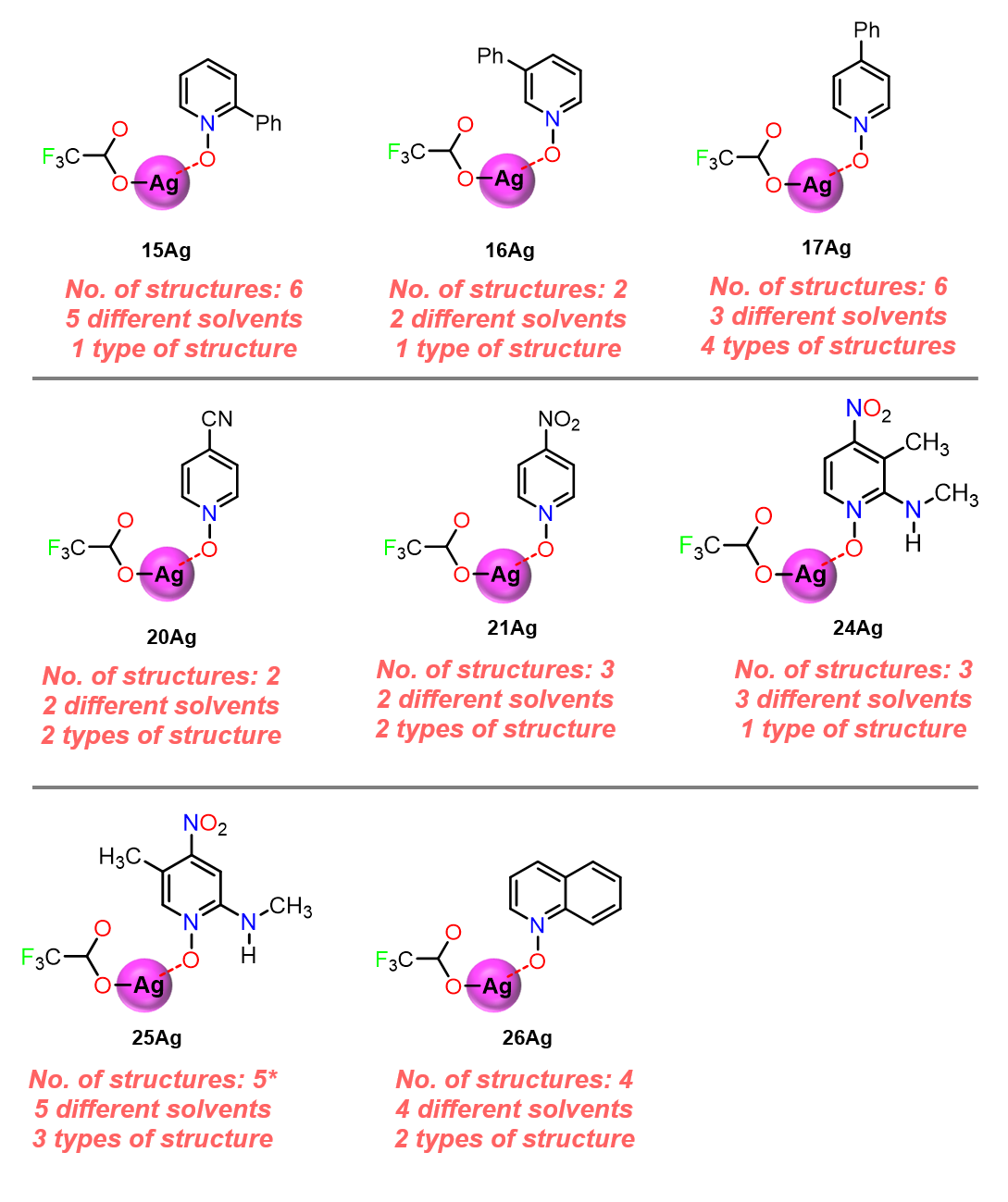


**Figure S57.** A list of PyNO-AgTFA complexes showing number of structures obtained from different solvents. *Although these complexes have different unit cell diameters due to the solvents in the lattices, they are all identical and regarded as one due to their common silver(I) skeleton.

**Table S12.** Coordination modes of *N*-oxide oxygens in silver(I) complexes. Note: For instance, 1Ag, 1H = *N*-oxide oxygen is displaying one coordination bond and one hydrogen bond.

| **S.No** | **Complex** | **1Ag** | **1Ag, 1H** | **1Ag, 2H** | **2Ag** | **2Ag, 1H** | **2Ag, 1H** |
| --- | --- | --- | --- | --- | --- | --- | --- |
| 1 | **(1Ag)Ben** |  |  | 1 |  |  |  |
| 2 | **(1Ag)Tol** |  |  | 1 |  |  |  |
| 3 | **(1Ag)H_2_O** |  |  | 1 |  |  |  |
| 4 | **(3Ag)EtOH** |  |  |  |  | 2 |  |
| 5 | **(3Ag)H_2_O** |  |  |  |  | 2 |  |
| 6 | **(3Ag) H_2_O_a** |  |  |  |  | 2 |  |
| 7 | **(4Ag)Acet** |  | 1 |  |  | 3 |  |
| 8 | **(4Ag)Acet_2** |  |  | 1 |  |  |  |
| 9 | **(4Ag)Acet_3** |  | 1 |  |  | 3 |  |
| 10 | **(4Ag)Acet_4** |  |  | 1 |  |  |  |
| 11 | **(4Ag)EtOH_2** |  |  | 1 |  |  |  |
| 12 | **(4Ag) EtOH_3** |  |  | 1 |  |  |  |
| 13 | **(4Ag)EtOH_4** |  |  | 1 |  |  |  |
| 14 | **(4Ag) EtOH_7** |  | 1 |  |  | 3 |  |
| 15 | **(4Ag)H_2_O** |  | 1 |  |  | 2 |  |
| 16 | **(4Ag)H_2_O_2** |  |  | 1 |  |  |  |
| 17 | **(4Ag)H_2_O_3** |  |  | 1 |  |  |  |
| 18 | **(4Ag)H_2_O_4** |  |  | 1 |  |  |  |
| 19 | **(4Ag)H_2_O_5** |  | 1 |  |  | 2 |  |
| 20 | **(4Ag)H_2_O_a** |  |  | 1 |  | 2 |  |
| 21 | **(8Ag)H_2_O** |  |  |  |  | 4 |  |
| 22 | **(9Ag)Tol** |  |  |  | 2 |  |  |
| 23 | **(10Ag)EtOH** |  | 1 |  |  | 3 |  |
| 24 | **(10Ag)H_2_O** |  | 1 |  |  | 3 |  |
| 25 | **(10Ag)H_2_O_a** |  | 1 |  |  | 3 |  |
| 26 | **(11Ag)EtOAc** |  |  | 1 |  |  |  |
| 27 | **(11Ag)EtOH** |  |  | 1 |  |  |  |
| 28 | **(11Ag)H_2_O** |  | 2 |  | 2 | 2 |  |
| 29 | **(11Ag)Tol** |  |  |  |  | 2 |  |
| 30 | **(13Ag)Acet** |  |  | 2 |  |  | 1 |
| 31 | **(13Ag)EtOAc_a** |  |  | 2 |  |  | 1 |
| 32 | **(13Ag)Tol** |  |  | 2 |  |  | 1 |
| 33 | **(14Ag)Ben** |  |  | 3 |  |  |  |
| 34 | **(14Ag)EtOAc_a** |  |  | 3 |  |  |  |
| 35 | **(15Ag)Acet** |  | 1 |  |  |  |  |
| 36 | **(15Ag)EtOAc** |  | 1 |  |  |  |  |
| 37 | **(15Ag)EtOAc_a** |  | 1 |  |  |  |  |
| 38 | **(15Ag)EtOH** |  | 1 |  |  |  |  |
| 39 | **(15Ag)H_2_O** |  | 1 |  |  |  |  |
| 40 | **(15Ag)Tol** |  | 1 |  |  |  |  |
| 41 | **(16Ag)EtOAc** |  |  | 1 |  | 2 |  |
| 42 | **(16Ag)Tol** |  |  | 1 |  | 2 |  |
| 43 | **(17Ag)Ben** |  | 1 |  | 2 |  |  |
| 44 | **(17Ag)Ben_a** |  | 1 |  |  |  |  |
| 45 | **(17Ag)EtOAc** |  |  |  |  | 2 |  |
| 46 | **(17Ag)EtOAc_a** |  |  | 2 |  |  |  |
| 47 | **(17Ag)Tol** |  |  | 2 |  |  |  |
| 48 | **(17Ag)Tol_a** |  | 1 |  |  |  |  |
| 49 | **(20Ag)Ben** |  | 1 |  |  | 1 |  |
| 50 | **(20Ag)Tol** |  | 3 |  |  | 2 |  |
| 51 | **(21Ag)H_2_O** |  |  | 1 |  |  |  |
| 52 | **(21Ag)Tol** |  |  | 1 |  |  |  |
| 53 | **(21Ag)Tol_a** |  |  | 2 |  |  |  |
| 54 | **(24Ag)Ben** |  |  |  | 2 |  |  |
| 55 | **(24Ag)EtOAc** |  |  |  | 2 |  |  |
| 56 | **(24Ag)Tol_a** |  |  |  | 2 |  |  |
| 57 | **(25Ag)Ben** |  |  |  | 2 |  |  |
| 58 | **(25Ag)Acet** | 1 |  |  | 6 |  |  |
| 59 | **(25Ag)EtOAc** | 1 |  |  | 6 |  |  |
| 60 | **(25Ag)EtOH** |  |  |  | 2 |  |  |
| 61 | **(25Ag)Tol_a** |  |  |  | 2 |  |  |
| 62 | **(26Ag)Acet** |  |  |  | 8 |  |  |
| 63 | **(26Ag)Ben** |  | 4 |  |  |  |  |
| 64 | **(26Ag)EtOAc** |  | 1 |  |  |  |  |
| 65 | **(26Ag)Tol** |  | 4 |  |  |  |  |
| **Subtotals** | | 2 | 32 | 36 | 38 | 47 | 3 |
| **Grand total** | | 158 | | | | | |

**Table S13.** List of silver(I) geometries. Note: For instance, AgO_3_ = silver is coordinated to one silver(I) and three oxygen atoms.

| **S.No** | **Complex** | **AgO_3_** | **AgO_4_** | **AgO_5_** | **Ag_2_O_2_** | **Ag_2_O_3_** | **Ag_2_O_4_** | **Ag_2_O_5_** | **Ag_3_O_3_** | **Ag_3_O_4_** | **AgNO_3_** | **AgNO_4_** | **Ag_2_NO_3_** |
| --- | --- | --- | --- | --- | --- | --- | --- | --- | --- | --- | --- | --- | --- |
| 1 | **(1Ag)Ben** |  | 2 |  |  |  |  |  |  |  |  |  |  |
| 2 | **(1Ag)Tol** |  | 2 |  |  |  |  |  |  |  |  |  |  |
| 3 | **(1Ag)H_2_O** |  | 2 |  |  |  |  |  |  |  |  |  |  |
| 4 | **(3Ag)EtOH** |  | 2 |  |  |  |  |  |  |  |  |  |  |
| 5 | **(3Ag)H_2_O** |  | 2 |  |  |  |  |  |  |  |  |  |  |
| 6 | **(3Ag) H_2_O_a** |  | 2 |  |  |  |  |  |  |  |  |  |  |
| 7 | **(4Ag)Acet** |  | 2 |  |  | 1 |  |  |  | 1 |  |  |  |
| 8 | **(4Ag)Acet_2** |  | 1 |  |  | 1 |  |  |  |  |  |  |  |
| 9 | **(4Ag)Acet_3** |  | 2 |  |  | 1 |  |  |  | 1 |  |  |  |
| 10 | **(4Ag)Acet_4** |  | 1 |  |  | 1 |  |  |  |  |  |  |  |
| 11 | **(4Ag)EtOH_2** |  | 1 |  |  | 1 |  |  |  |  |  |  |  |
| 12 | **(4Ag) EtOH_3** |  | 1 |  |  | 1 |  |  |  |  |  |  |  |
| 13 | **(4Ag)EtOH_4** |  | 1 |  |  | 1 |  |  |  |  |  |  |  |
| 14 | **(4Ag) EtOH_7** |  | 2 |  |  | 1 |  |  |  | 1 |  |  |  |
| 15 | **(4Ag)H_2_O** |  | 1 |  |  |  |  |  | 1 | 1 |  |  |  |
| 16 | **(4Ag)H_2_O_2** | 1 | 1 |  |  |  |  |  |  |  |  |  |  |
| 17 | **(4Ag)H_2_O_3** | 1 | 1 |  |  |  |  |  |  |  |  |  |  |
| 18 | **(4Ag)H_2_O_4** | 1 | 1 |  |  |  |  |  |  |  |  |  |  |
| 19 | **(4Ag)H_2_O_5** |  | 1 |  |  |  |  |  | 1 | 1 |  |  |  |
| 20 | **(4Ag)H_2_O_a** |  | 1 |  |  |  |  |  | 1 | 1 |  |  |  |
| 21 | **(8Ag)H_2_O** |  | 4 |  |  |  |  |  |  |  |  |  |  |
| 22 | **(9Ag)Tol** |  | 2 |  |  |  |  |  |  |  |  |  |  |
| 23 | **(10Ag)EtOH** |  | 4 |  |  |  |  |  |  |  |  |  |  |
| 24 | **(10Ag)H_2_O** |  | 4 |  |  |  |  |  |  |  |  |  |  |
| 25 | **(10Ag)H_2_O_a** |  | 4 |  |  |  |  |  |  |  |  |  |  |
| 26 | **(11Ag)EtOAc** |  |  |  |  |  | 2 |  |  |  |  |  |  |
| 27 | **(11Ag)EtOH** |  |  |  |  |  | 2 |  |  |  |  |  |  |
| 28 | **(11Ag)H_2_O** | 1 |  | 2 |  |  | 1 |  |  |  |  |  |  |
| 29 | **(11Ag)Tol** |  | 2 |  |  |  |  |  |  |  |  |  |  |
| 30 | **(13Ag)Acet** |  | 1 |  |  |  |  |  | 2 |  |  |  |  |
| 31 | **(13Ag)EtOAc_a** |  | 1 |  |  |  |  |  | 2 |  |  |  |  |
| 32 | **(13Ag)Tol** |  | 1 |  |  |  |  |  | 2 |  |  |  |  |
| 33 | **(14Ag)Ben** | 2 |  |  |  | 3 |  |  | 1 |  |  |  |  |
| 34 | **(14Ag)EtOAc_a** | 2 |  |  |  | 3 |  |  | 1 |  |  |  |  |
| 35 | **(15Ag)Acet** | 1 |  |  |  |  |  |  |  |  |  |  |  |
| 36 | **(15Ag)EtOAc** | 1 |  |  |  |  |  |  |  |  |  |  |  |
| 37 | **(15Ag)EtOAc_a** | 1 |  |  |  |  |  |  |  |  |  |  |  |
| 38 | **(15Ag)EtOH** | 1 |  |  |  |  |  |  |  |  |  |  |  |
| 39 | **(15Ag)H_2_O** | 1 |  |  |  |  |  |  |  |  |  |  |  |
| 40 | **(15Ag)Tol** | 1 |  |  |  |  |  |  |  |  |  |  |  |
| 41 | **(16Ag)EtOAc** |  |  |  |  |  | 1 |  | 1 | 1 |  |  |  |
| 42 | **(16Ag)Tol** |  |  |  |  |  | 1 |  | 1 | 1 |  |  |  |
| 43 | **(17Ag)Ben** |  | 1 |  |  | 1 | 1 |  |  |  |  |  |  |
| 44 | **(17Ag)Ben_a** | 1 | 1 |  |  |  |  |  |  |  |  |  |  |
| 45 | **(17Ag)EtOAc** |  | 2 |  |  |  |  |  |  |  |  |  |  |
| 46 | **(17Ag)EtOAc_a** |  | 2 |  |  |  |  |  |  |  |  |  |  |
| 47 | **(17Ag)Tol** |  | 2 |  |  |  |  |  |  |  |  |  |  |
| 48 | **(17Ag)Tol_a** | 1 | 1 |  |  |  |  |  |  |  |  |  |  |
| 49 | **(20Ag)Ben** |  |  |  |  |  |  |  |  |  |  | 1 | 1 |
| 50 | **(20Ag)Tol** | 1 |  |  |  | 1 | 1 | 1 |  |  | 1 |  |  |
| 51 | **(21Ag)H_2_O** |  |  |  |  |  | 1 |  | 1 |  |  |  |  |
| 52 | **(21Ag)Tol** |  | 1 |  |  | 2 |  |  | 1 |  |  |  |  |
| 53 | **(21Ag)Tol_a** |  |  |  |  |  | 1 |  | 1 |  |  |  |  |
| 54 | **(24Ag)Ben** |  | 2 |  |  |  |  |  |  |  |  |  |  |
| 55 | **(24Ag)EtOAc** |  | 2 |  |  |  |  |  |  |  |  |  |  |
| 56 | **(24Ag)Tol_a** |  | 2 |  |  |  |  |  |  |  |  |  |  |
| 57 | **(25Ag)Ben** |  | 1 |  |  |  |  |  |  |  |  |  |  |
| 58 | **(25Ag)Acet** |  | 2 |  |  |  |  |  | 1 | 1 |  |  |  |
| 59 | **(25Ag)EtOAc** |  | 2 |  |  |  |  |  | 1 | 1 |  |  |  |
| 60 | **(25Ag)EtOH** |  | 1 |  |  |  |  |  |  |  |  |  |  |
| 61 | **(25Ag)Tol_a** |  | 1 |  |  |  |  |  |  |  |  |  |  |
| 62 | **(26Ag)Acet** |  | 4 |  |  |  |  |  |  |  |  |  |  |
| 63 | **(26Ag)Ben** | 2 | 2 |  |  |  |  |  |  |  |  |  |  |
| 64 | **(26Ag)EtOAc** | 1 | 1 |  |  |  |  |  |  |  |  |  |  |
| 65 | **(26Ag)Tol** | 2 | 2 |  |  |  |  |  |  |  |  |  |  |
| **Subtotals** | | 22 | 84 | 2 | 0 | 18 | 11 | 1 | 18 | 10 | 1 | 1 | 1 |
| **Grand total** | | 169 | | | | | | | | | | | |

**1. Complex (1Ag)Ben**


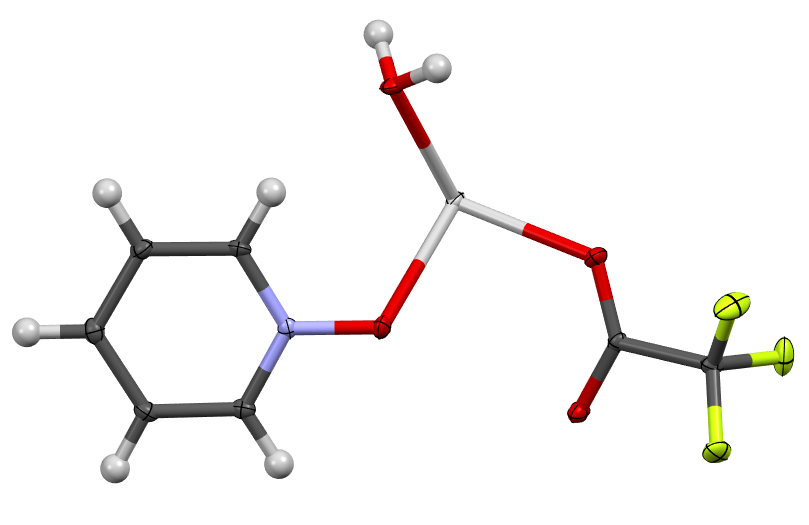


**Figure S58**. The asymmetric unit of **(1Ag)Ben** with the thermal displacement parameter at 50% probability level. Colour Key: green = fluorine, ash white = silver, blue = nitrogen, dark grey = carbon, white = hydrogen. Disordered TFA part is omitted for viewing clarity.

**Crystal data for (1Ag)Ben**: CCDC-2420927, C_14_H_14_Ag_2_F_6_N_2_O_8_, M = 668.01 gmol^‑1^, colourless block, 0.14 × 0.12 × 0.05 mm^3^, monoclinic, space group *P2_1_/c*, a = 5.86810(10) Å, b = 22.8751(3) Å, c = 7.48600(10) Å, α = 90°, β = 106.1800(10) °, γ = 90°, V = 965.07(2) Å^3^, Z = 2, D_calc_ = 2.299 gcm^‑3^, F(000) = 648, µ = 17.291 mm^‑1^, T = 120(1) K, θ_max_ = 66.748°, 11487 total reflections, 1666 with I_o_ > 2σ(I_o_), R_int_ = 0.0459, 1699 data, 146 parameters, 0 restraints, GooF = 1.110, R = 0.0229 and wR = 0.0604 [I_o_ > 2σ(I_o_)], R = 0.0233 and wR = 0.0607 (all reflections), 0.690 <d∆ρ <-0.715 eÅ^‑3^.

**2. Complex (1Ag)Tol**


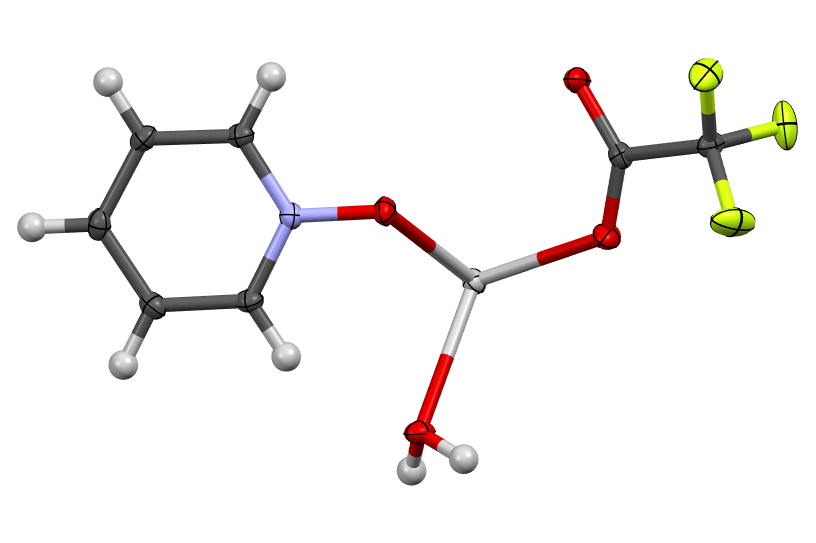


**Figure S59**. The asymmetric unit of **(1Ag)Tol** with the thermal displacement parameter at 50% probability level. Colour Key: green = fluorine, ash white = silver, blue = nitrogen, dark grey = carbon, white = hydrogen. Disordered TFA part is omitted for viewing clarity.

**Crystal data for (1Ag)Tol**: CCDC-2420932, C_14_H_14_Ag_2_F_6_N_2_O_8_, M = 668.01 gmol^‑1^, colourless plate, 0.19 × 0.11 × 0.05 mm^3^, monoclinic, space group *P2_1_/c*, a = 5.87840(10) Å, b = 22.9155(3) Å, c = 7.50580(10) Å, α = 90°, β = 106.035(2)°, γ = 90°, V = 971.74(3) Å^3^, Z = 2, D_calc_ = 2.283 gcm^‑3^, F(000) = 648, µ = 17.172 mm^‑1^, T = 120(1) K, θ_max_ = 66.744°, 11568 total reflections, 1673 with I_o_ > 2σ(I_o_), R_int_ = 0.0440, 1713 data, 146 parameters, 0 restraints, GooF = 1.095, R = 0.0212 and wR = 0.0543 [I_o_ > 2σ(I_o_)], R = 0.0217 and wR = 0.0547 (all reflections), 0.343 <d∆ρ < -0.597 eÅ^‑3^.

**3. Complex (1Ag)H_2_O**


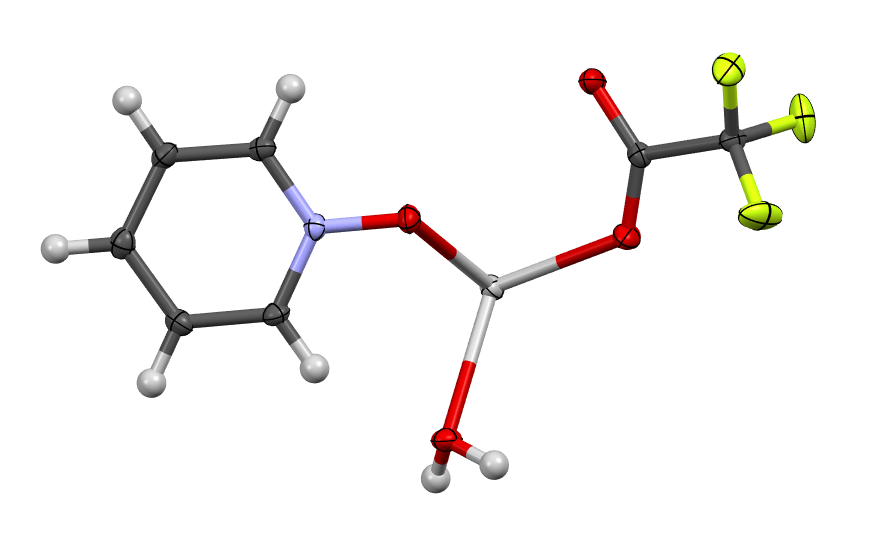


**Figure S60**. The asymmetric unit of **(1Ag)H_2_O** with the thermal displacement parameter at 50% probability level. Colour Key: green = fluorine, ash white = silver, blue = nitrogen, dark grey = carbon, white = hydrogen. Disordered TFA part is omitted for viewing clarity.

**Crystal data for (1Ag)H_2_O**: CCDC-2420928, C_14_H_14_Ag_2_F_6_N_2_O_8_, M = 668.01 gmol^‑1^, colourless block, 0.08 × 0.05 × 0.04 mm^3^, monoclinic, space group *P2_1_/c*, a = 5.87720(10) Å, b = 22.9278(5) Å, c = 7.5145(2) Å, α = 90°, β = 106.028(3)°, γ = 90°, V = 973.23(4)Å^3^, Z = 2, D_calc_ = 2.280 gcm^‑3^, F(000) = 648, µ = 17.146 mm^‑1^, T = 150(1) K, θ_max_ = 66.736°, 6852 total reflections, 1631 with I_o_ > 2σ(I_o_), R_int_ = 0.0241, 1716 data, 146 parameters, 0 restraints, GooF = 1.081, R = 0.0207 and wR = 0.0528 [I_o_ > 2σ(I_o_)], R = 0.0220 and wR = 0.0534 (all reflections), 0.536 <d∆ρ < -0.663 eÅ^‑3^.

**4. Complex (3Ag)EtOH**


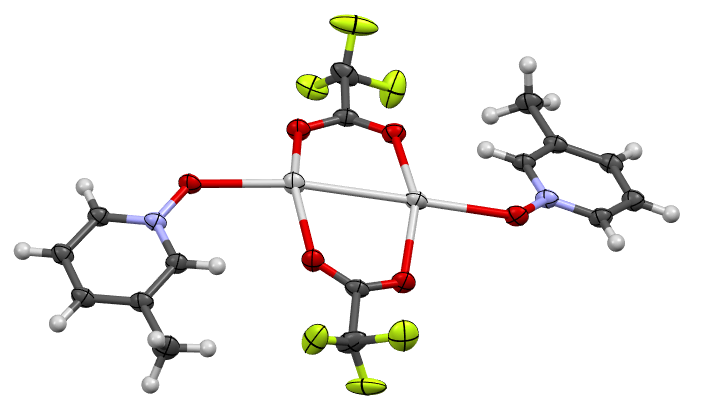


**Figure S61**. The asymmetric unit of **(3Ag)EtOH** with the thermal displacement parameter at 50% probability level. Colour Key: green = fluorine, ash white = silver, blue = nitrogen, dark grey = carbon, white = hydrogen. Disordered TFA part is omitted for viewing clarity.

**Crystal data for (3Ag)EtOH**: CCDC-2420930, C_16_H_14_Ag_2_F_6_N_2_O_6_, M = 660.03 gmol^‑1^, colourless plates/block, 0.08 × 0.05 × 0.04 mm^3^, monoclinic, space group *P2_1_/c*, a = 8.02010(10) Å, b = 11.1173(2) Å, c = 23.3131(4) Å, α = 90°, β = 98.774(2)°, γ = 90°, V = 2054.31(6) Å^3^, Z = 4, D_calc_ = 2.134 gcm^‑3^, F(000) = 1280, µ = 16.162 mm^‑1^, T = 120(1) K, θ_max_ = 66.744°, 21310 total reflections, 3357 with I_o_ > 2σ(I_o_), R_int_ = 0.0409, 3648 data, 345 parameters, 0 restraints, GooF = 1.060, R = 0.0273 and wR = 0.0683 [I_o_ > 2σ(I_o_)], R = 0.0302 and wR = 0.0708 (all reflections), 0.859 <d∆ρ <-0.567 eÅ^‑3^.

**5. Complex (3Ag)H_2_O**


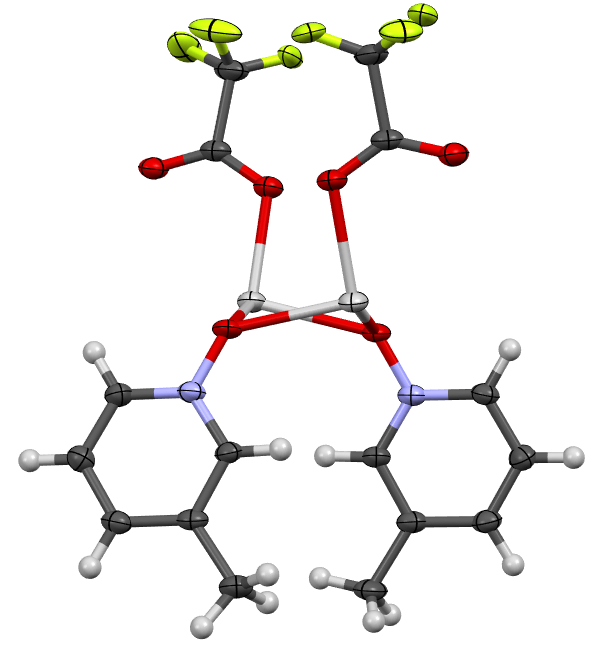


**Figure S62**. The asymmetric unit of **(3Ag)H_2_O** with the thermal displacement parameter at 50% probability level. Colour Key: green = fluorine, ash white = silver, blue = nitrogen, dark grey = carbon, white = hydrogen. Disordered TFA part is omitted for viewing clarity.

**Crystal data for (3Ag)H_2_O**: CCDC-2420934, C_16_H_14_Ag_2_F_6_N_2_O_6_, M = 660.03 gmol^‑1^, colourless block, 0.11 × 0.05 × 0.01 mm^3^, monoclinic, space group *P2_1_/c*, a = 8.0214(3) Å, b = 11.1220(4) Å, c = 23.2844(9) Å, α = 90°, β = 98.813(9)°, γ = 90°, V = 2052.77(13) Å^3^, Z = 4, D_calc_ = 2.136 gcm^‑3^, F(000) = 1280, µ = 16.174 mm^‑1^, T = 120(1) K, θ_max_ = 66.749°, 13812 total reflections, 2969 with I_o_ > 2σ(I_o_), R_int_ = 0.0523, 3647 data, 345 parameters, 0 restraints, GooF = 1.110, R = 0.0388 and wR = 0.0996 [I_o_ > 2σ(I_o_)], R = 0.0492 and wR = 0.1050 (all reflections), 1.337 <d∆ρ <-1.075 eÅ^‑3^.

**6. Complex (3Ag)H_2_O_2**


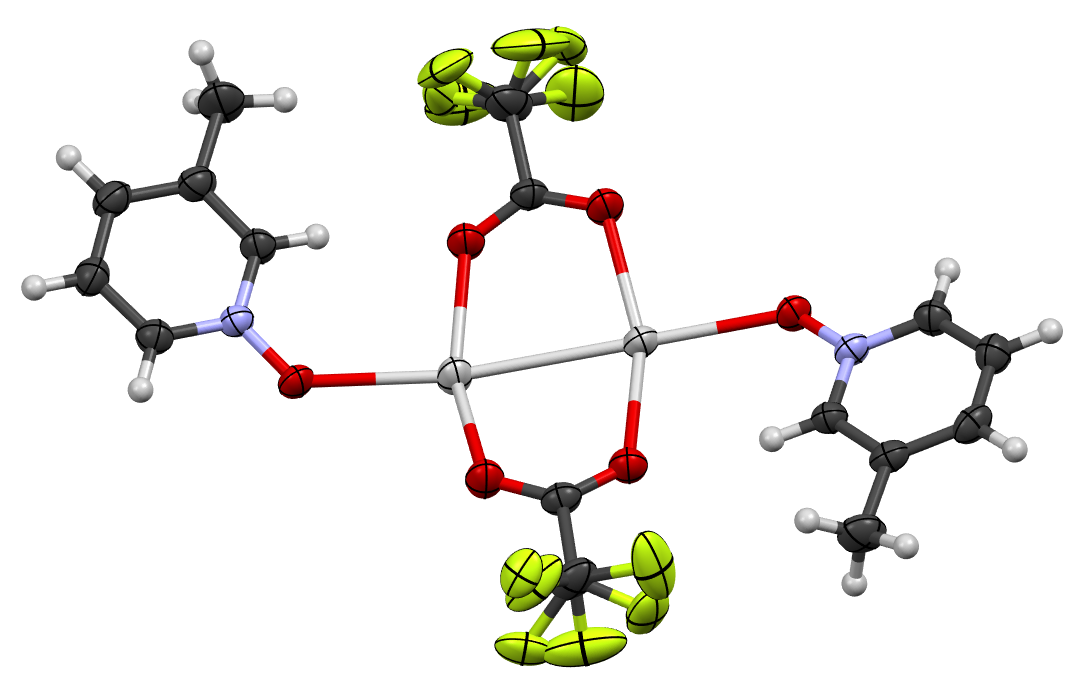


**Figure S63**. The asymmetric unit of **(3Ag)H_2_O_2** with the thermal displacement parameter at 50% probability level. Colour Key: green = fluorine, ash white = silver, blue = nitrogen, dark grey = carbon, white = hydrogen. Disordered TFA part is omitted for viewing clarity.

**Crystal data for (3Ag)H_2_O_2**: CCDC-2420931, C_16_H_14_Ag_2_F_6_N_2_O_6_, M = 660.03 gmol^‑1^, colourless block, 0.14 × 0.07 × 0.04 mm^3^, monoclinic, space group *P2_1_/c*, a = 8.05130(10) Å, b = 11.11950(10) Å, c = 23.3189(3) Å, α = 90°, β = 99.0900(10)°, γ = 90°, V = 2061.44(4) Å^3^, Z = 4, D_calc_ = 2.127 gcm^‑3^, F(000) = 1280, µ = 16.106 mm^‑1^, T = 150(1) K, θ_max_ = 66.747°, 15915 total reflections, 3427 with I_o_ > 2σ(I_o_), R_int_ = 0.0308, 3668 data, 345 parameters, 12 restraints, GooF = 1.035, R = 0.0243 and wR = 0.0610 [I_o_ > 2σ(I_o_)], R = 0.0259 and wR = 0.0620 (all reflections), 0.516 <d∆ρ < -0.788 eÅ^‑3^.

**7. Complex (4Ag)Acet**


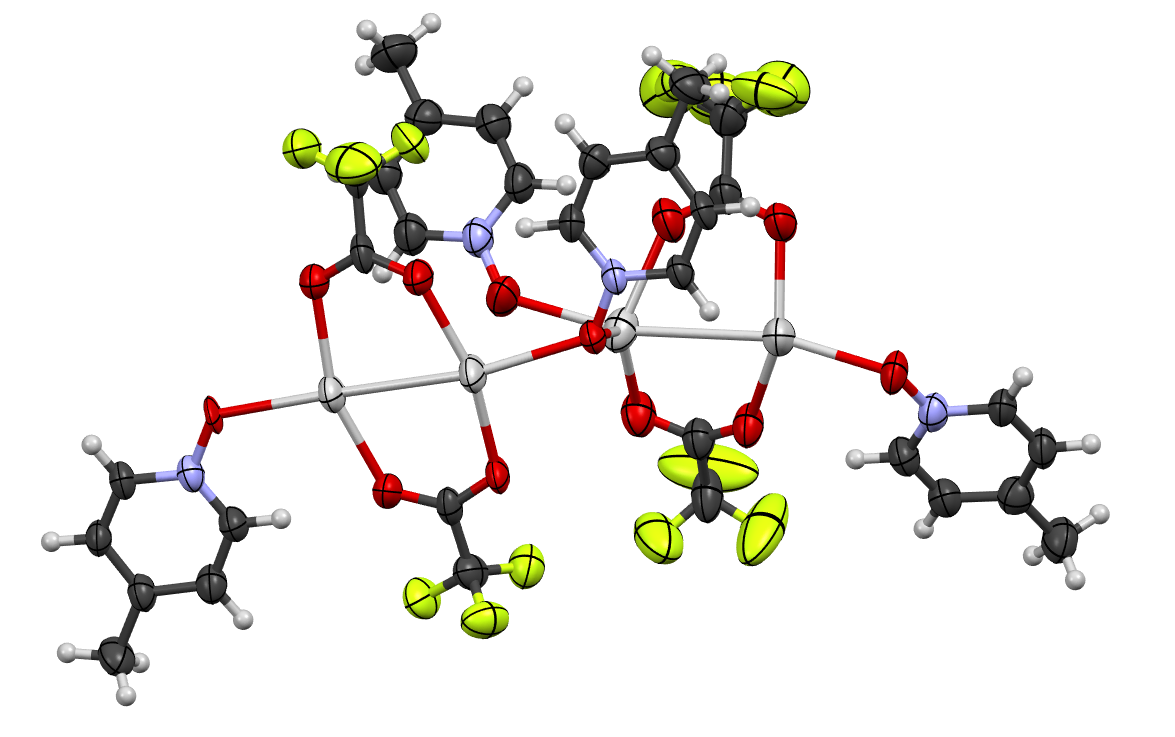


**Figure S64**. The asymmetric unit of **(4Ag)Acet** with the thermal displacement parameter at 50% probability level. Colour Key: green = fluorine, ash white = silver, blue = nitrogen, dark grey = carbon, white = hydrogen. Disordered TFA part is omitted for viewing clarity.

**Crystal data for (4Ag)Acet**: CCDC-2420936, C_32_H_28_Ag_4_F_12_N_4_O_12_, M = 1320.06 gmol^‑1^, colourless plate, 0.13 × 0.1 × 0.03 mm^3^, triclinic, space group *P-1*, a = 11.1408(4) Å, b = 14.2693(10) Å, c = 15.8845(9) Å, α = 115.148(7)°, β = 91.377(4)°, γ = 95.088(4)°, V = 2271.5(3) Å^3^, Z = 2, D_calc_ = 1.930 gcm^‑3^, F(000) = 1280, µ = 14.617 mm^‑1^, T = 120(1) K, θ_max_ = 66.733°, 28279 total reflections, 5612 with I_o_ > 2σ(I_o_), R_int_ = 0.1227, 8006 data, 608 parameters, 36 restraints, GooF = 1.040, R = 0.0712 and wR = 0.1910 [I_o_ > 2σ(I_o_)], R = 0.0950 and wR = 0.2146 (all reflections), 1.207 <d∆ρ <-2.042 eÅ^‑3^.

**8. Complex (4Ag)Acet_2**


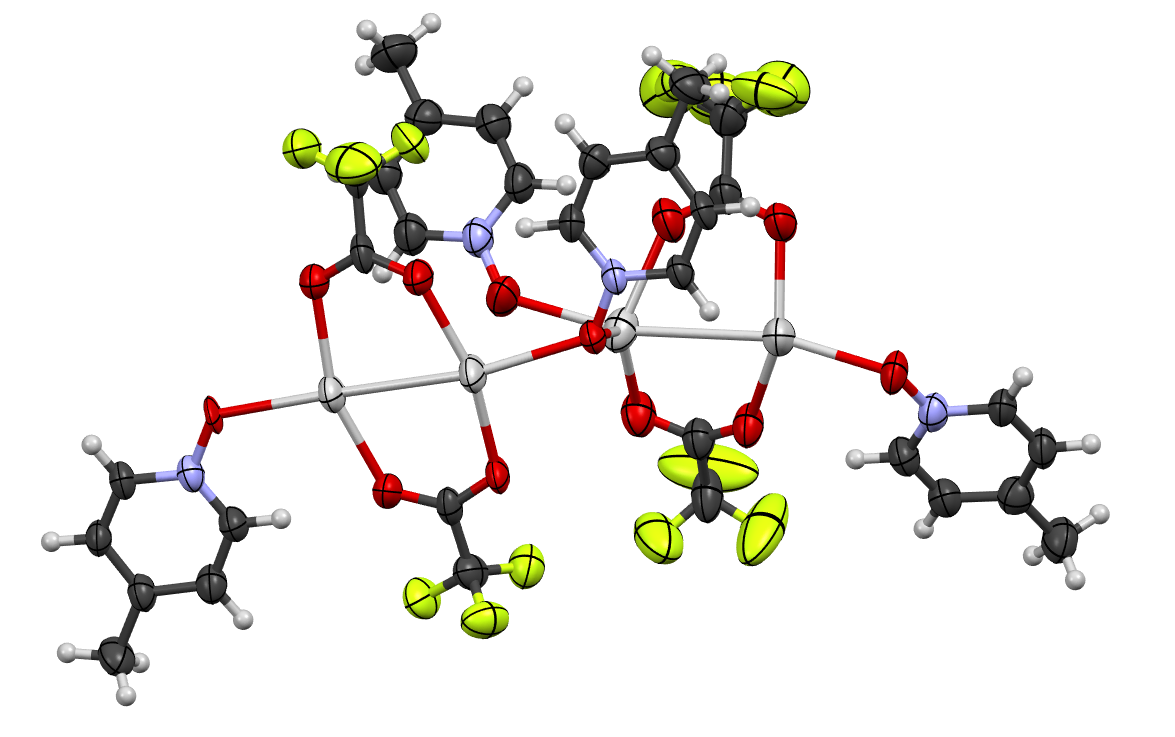


**Figure S65**. The asymmetric unit of **(4Ag)Acet_2** with the thermal displacement parameter at 50% probability level. Colour Key: green = fluorine, ash white = silver, blue = nitrogen, dark grey = carbon, white = hydrogen. Disordered TFA part is omitted for viewing clarity.

**Crystal data for (4Ag)Acet_2**: CCDC-2420940, C_10_H_9_Ag_2_F_6_NO_6_, M = 568.92 gmol^‑1^, colourless plate, 0.25 × 0.09 × 0.03 mm^3^, triclinic, space group *P-1*, a = 7.1272(3) Å, b = 7.9649(4) Å, c = 13.8340(8) Å, α = 91.870(4)°, β = 94.100(4)°, γ = 104.262(4)°, V = 758.14(7) Å^3^, Z = 2, D_calc_ = 2.492 gcm^‑3^, F(000) = 544, µ = 21.708 mm^‑1^, T = 170(1) K, θ_max_ = 66.739°, 2692 total reflections, 2448 with I_o_ > 2σ(I_o_), R_int_ = 0.0565, 2692 data, 262 parameters, 2 restraints, GooF = 1.089, R = 0.0422 and wR = 0.1060 [I_o_ > 2σ(I_o_)], R = 0.0461 and wR = 0.1091 (all reflections), 1.261<d∆ρ <-2.101 eÅ^‑3^.

**9. Complex (4Ag)Acet_3**


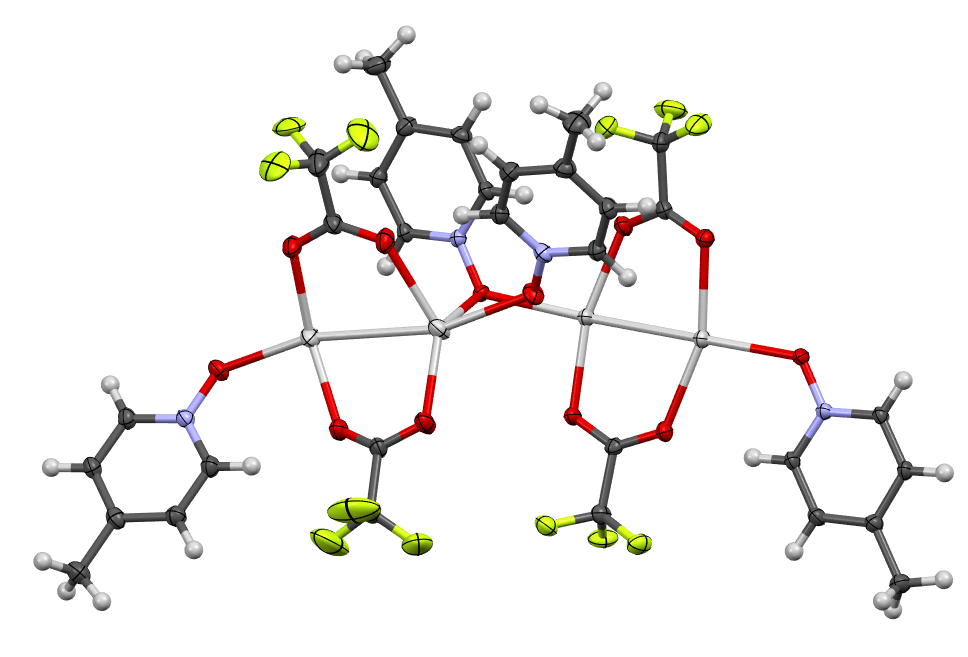


**Figure S66**. The asymmetric unit of **(4Ag)Acet_3** with the thermal displacement parameter at 50% probability level. Colour Key: green = fluorine, ash white = silver, blue = nitrogen, dark grey = carbon, white = hydrogen. Disordered TFA part is omitted for viewing clarity.

**Crystal data for (4Ag)Acet_3**: CCDC-2420935, C_32_H_28_Ag_4_F_12_N_4_O_12_, M = 1320.06 gmol^‑1^, colourless plate, 0.15 × 0.07 × 0.03 mm^3^, triclinic, space group *P-1*, a = 11.1539(2) Å, b = 14.2692(3) Å, c = 15.8190(3) Å, α = 115.687(2)°, β = 91.3170(10)°, γ = 95.049(2)°, V = 2254.84(8) Å^3^, Z = 2, D_calc_ = 1.944 gcm^‑3^, F(000) = 1280, µ = 14.725 mm^‑1^, T = 120(1) K, θ_max_ = 66.742°, 30608 total reflections, 7056 with I_o_ > 2σ(I_o_), R_int_ = 0.0418, 7973 data, 608 parameters, 36 restraints, GooF = 1.062, R = 0.0350 and wR = 0.0897 [I_o_ > 2σ(I_o_)], R = 0.0394 and wR = 0.0939 (all reflections), 2.200 <d∆ρ < -1.745 eÅ^‑3^.

**10. Complex (4Ag)Acet_4**


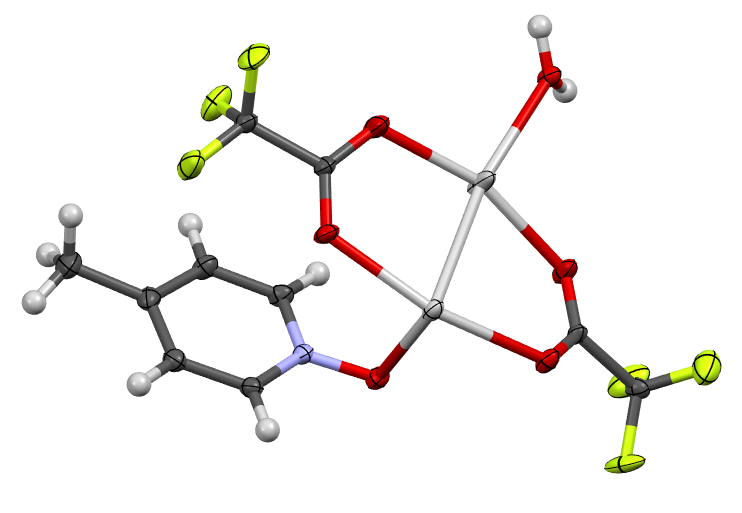


**Figure S67**. The asymmetric unit of **(4Ag)Acet_4** with the thermal displacement parameter at 50% probability level. Colour Key: green = fluorine, ash white = silver, blue = nitrogen, dark grey = carbon, white = hydrogen.

**Crystal data for (4Ag)Acet_4**: CCDC-2420944, C_10_H_9_Ag_2_F_6_NO_6_, M = 568.92 gmol^‑1^, colourless plate, 0.16 × 0.14 × 0.05 mm^3^, triclinic, space group *P-1*, a = 7.1501(3) Å, b = 7.9656(3) Å, c = 13.8632(4) Å, α = 91.776(3)°, β = 94.182(3)°, γ = 104.160(3)°, V = 762.57(5) Å^3^, Z = 2, D_calc_ = 2.478 gcm^‑3^, F(000) = 544, µ = 21.582 mm^‑1^, T = 150(1) K, θ_max_ = 66.712°, 8590 total reflections, 2430 with I_o_ > 2σ(I_o_), R_int_ = 0.0608, 2689 data, 262 parameters, 26 restraints, GooF = 1.062, R = 0.0438 and wR = 0.1210 [I_o_ > 2σ(I_o_)], R = 0.0473 and wR = 0.1236 (all reflections), 1.006 <d∆ρ < -1.568 eÅ^‑3^.

**11. Complex (4Ag)EtOH_2**


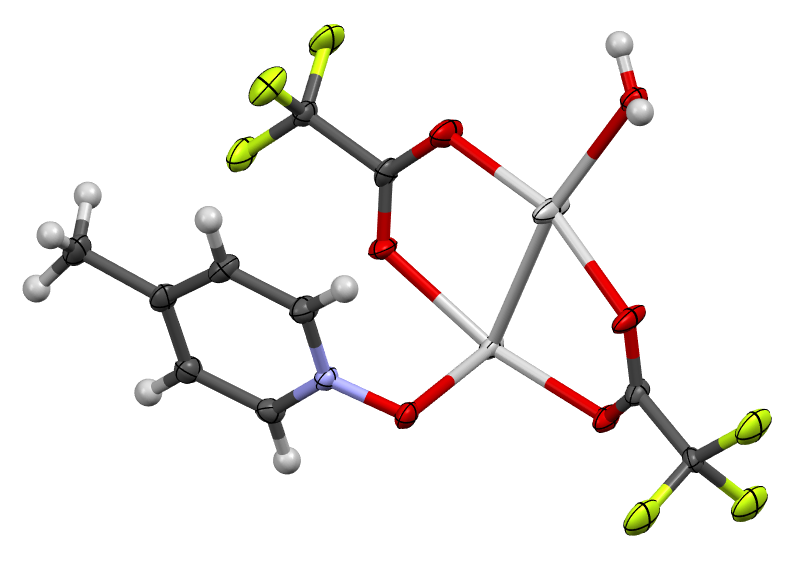


**Figure S68**. The asymmetric unit of **(4Ag)EtOH_2** with the thermal displacement parameter at 50% probability level. Colour Key: green = fluorine, ash white = silver, blue = nitrogen, dark grey = carbon, white = hydrogen.

**Crystal data for (4Ag)EtOH_2**: CCDC-2420933, C_10_H_9_Ag_2_F_6_NO_6_, M = 568.92 gmol^‑1^, colourless plate, 0.07 × 0.05 × 0.02 mm^3^, triclinic, space group *P-1*, a = 7.1617(4) Å, b = 7.9631(4) Å, c = 13.8654(5) Å, α = 91.780(3)°, β = 94.209(3)°, γ = 104.191(4)°, V = 763.55(7) Å^3^, Z = 2, D_calc_ = 2.475 gcm^‑3^, F(000) = 544, µ = 21.555 mm^‑1^, T = 150(1) K, θ_max_ = 66.709°, 8627 total reflections, 2230 with I_o_ > 2σ(I_o_), R_int_ = 0.0571, 2705 data, 238 parameters, 20 restraints, GooF = 1.058, R = 0.0471 and wR = 0.1131 [I_o_ > 2σ(I_o_)], R = 0.0594 and wR = 0.1196 (all reflections), 1.422 <d∆ρ < -1.308 eÅ^‑3^.

**12. Complex (4Ag)EtOH_3**


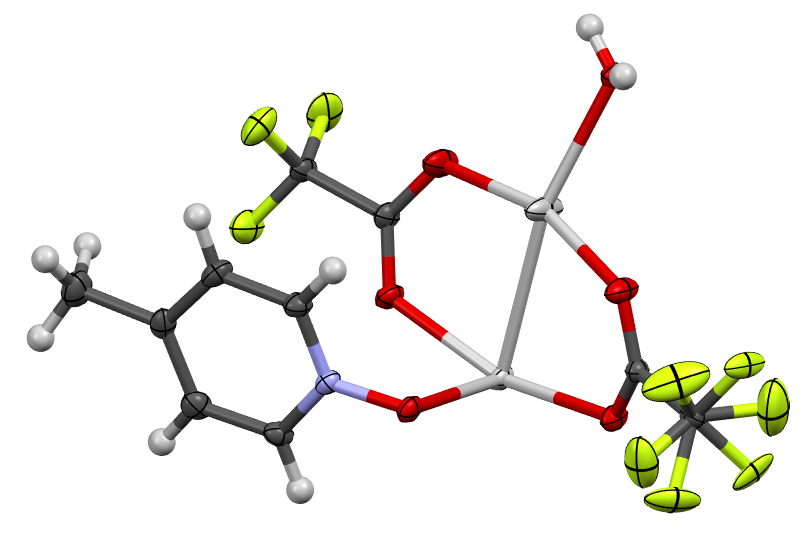


**Figure 69**. The asymmetric unit of **(4Ag)EtOH_3** with the thermal displacement parameter at 50% probability level. Colour Key: green = fluorine, ash white = silver, blue = nitrogen, dark grey = carbon, white = hydrogen.

**Crystal data for (4Ag)EtOH_3**: CCDC-2420943, C_10_H_9_Ag_2_F_6_NO_6_, M = 568.92 gmol^‑1^, colourless plate, 0.07 × 0.06 × 0.02 mm^3^, triclinic, space group *P-1*, a = 7.1608(2) Å, b = 7.9601(3) Å, c = 13.8606(5) Å, α = 91.765(3)°, β = 94.249(3)°, γ = 104.123(3)°, V = 763.10(5) Å^3^, Z = 2, D_calc_ = 2.476 gcm^‑3^, F(000) = 544, µ = 21.567 mm^‑1^, T = 150(1) K, θ_max_ = 66.725°, 8824 total reflections, 2293 with I_o_ > 2σ(I_o_), R_int_ = 0.0554, 2706 data, 262 parameters, 20 restraints, GooF = 1.044, R = 0.0347 and wR = 0.0821 [I_o_ > 2σ(I_o_)], R = 0.0432 and wR = 0.0860 (all reflections), 0.623 <d∆ρ < -0.965 eÅ^‑3^.

**13. Complex (4Ag)EtOH_4**


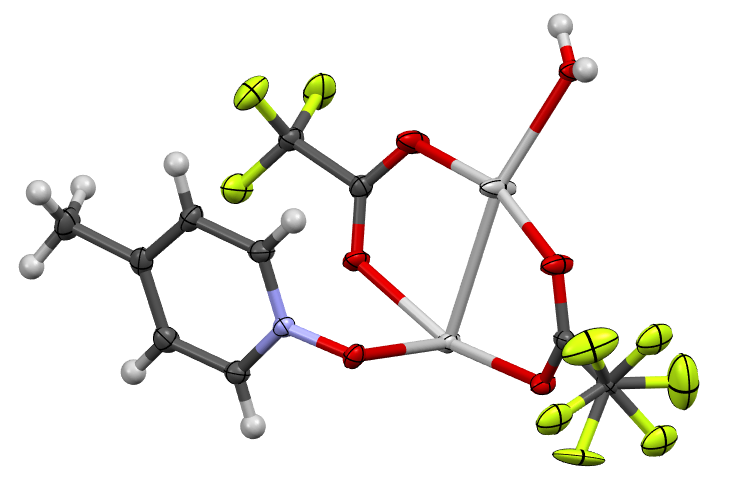


**Figure S70**. The asymmetric unit of **(4Ag)EtOH_4** with the thermal displacement parameter at 50% probability level. Colour Key: green = fluorine, ash white = silver, blue = nitrogen, dark grey = carbon, white = hydrogen.

**Crystal data for (4Ag)EtOH_4**: CCDC-2420946, C_10_H_9_Ag_2_F_6_NO_6_, M = 568.92 gmol^‑1^, colourless plate, 0.12 × 0.08 × 0.03 mm^3^, triclinic, space group *P-1*, a = 7.1570(3) Å, b = 7.9619(2) Å, c = 13.8624(3) Å, α = 91.775(2)°, β = 94.183(3)°, γ = 104.142(3)°, V = 762.97(4) Å^3^, Z = 2, D_calc_ = 2.476 gcm^‑3^, F(000) = 544, µ = 21.571 mm^‑1^, T = 155(4) K, θ_max_ = 66.749°, 9182 total reflections, 2360 with I_o_ > 2σ(I_o_), R_int_ = 0.0494, 2702 data, 250 parameters, 26 restraints, GooF = 1.063, R = 0.0402 and wR = 0.1015 [I_o_ > 2σ(I_o_)], R = 0.0465 and wR = 0.1051 (all reflections), 1.126 <d∆ρ < -1.162 eÅ^‑3^.

**14. Complex (4Ag)EtOH_7**


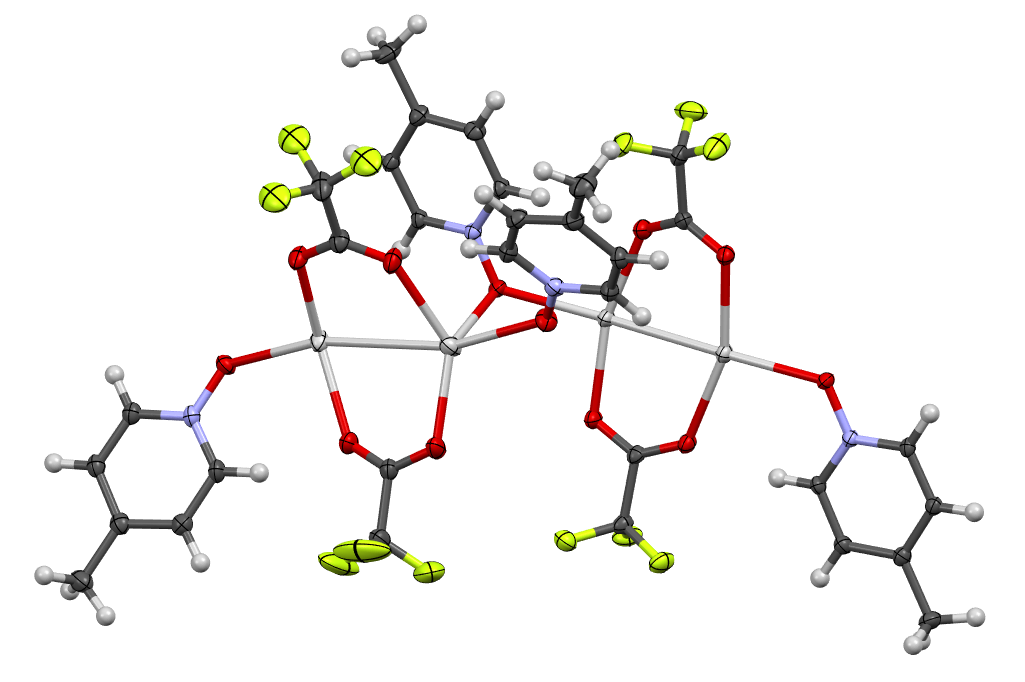


**Figure S71**. The asymmetric unit of **(4Ag)EtOH_7** with the thermal displacement parameter at 50% probability level. Colour Key: green = fluorine, ash white = silver, blue = nitrogen, dark grey = carbon, white = hydrogen.

**Crystal data for (4Ag)EtOH_7**: CCDC-2420938, C_32_H_28_Ag_4_F_12_N_4_O_12_, M = 1320.06 gmol^‑1^, colourless needle, 0.19 × 0.04 × 0.02 mm^3^, triclinic, space group *P-1*, a = 11.1552(2) Å, b = 14.2685(4) Å, c = 15.8003(4) Å, α = 115.894(3)°, β = 91.318(2)°, γ = 94.932(2)°, V = 2248.89(11) Å^3^, Z = 2, D_calc_ = 1.949 gcm^‑3^, F(000) = 1280, µ = 14.764 mm^‑1^, T = 150(1) K, θ_max_ = 66.745°, 33718 total reflections, 6775 with I_o_ > 2σ(I_o_), R_int_ = 0.0754, 7976 data, 605 parameters, 37 restraints, GooF = 1.037, R = 0.0392 and wR = 0.0972 [I_o_ > 2σ(I_o_)], R = 0.0463 and wR = 0.1006 (all reflections), 1.157 <d∆ρ < -0.953 eÅ^‑3^.

**15. Complex (4Ag)H_2_O**


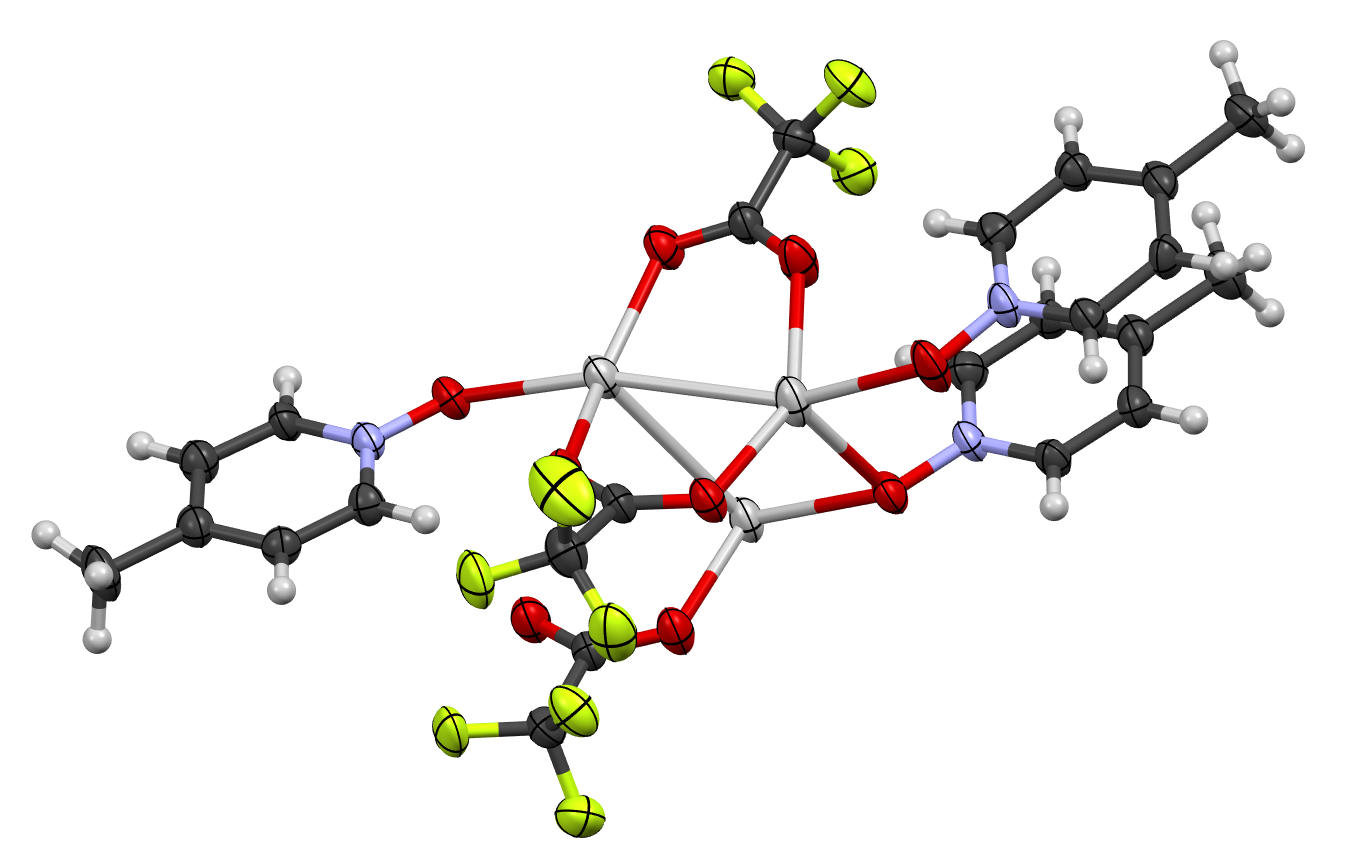


**Figure S72**. The asymmetric unit of **(4Ag)H_2_O** with the thermal displacement parameter at 50% probability level. Colour Key: green = fluorine, ash white = silver, blue = nitrogen, dark grey = carbon, white = hydrogen.

**Crystal data for (4Ag)H_2_O**: CCDC-2420942, C_48_H_42_Ag_6_F_18_N_6_O_18_, M = 1980.09 gmol^‑1^, colourless needle, 0.15 × 0.03 × 0.02 mm^3^, triclinic, space group *P-1*, a = 11.2131(7) Å, b = 11.2287(8) Å, c = 14.3328(6) Å, α = 108.770(5)°, β = 97.243(4)°, γ = 112.480(7)°, V = 1513.46(18) Å^3^, Z = 2, D_calc_ = 2.173 gcm^‑3^, F(000) = 960, µ = 16.454 mm^‑1^, T = 150(1) K, θ_max_ = 66.738°, 16661 total reflections, 3882 with I_o_ > 2σ(I_o_), R_int_ = 0.0805, 5316 data, 436 parameters, 0 restraints, GooF = 0.981, R = 0.0531 and wR = 0.1326 [I_o_ > 2σ(I_o_)], R = 0.0741 and wR = 0.1448 (all reflections), 1.075 <d∆ρ < -1.774 eÅ^‑3^.

**16. Complex (4Ag)H_2_O_2**


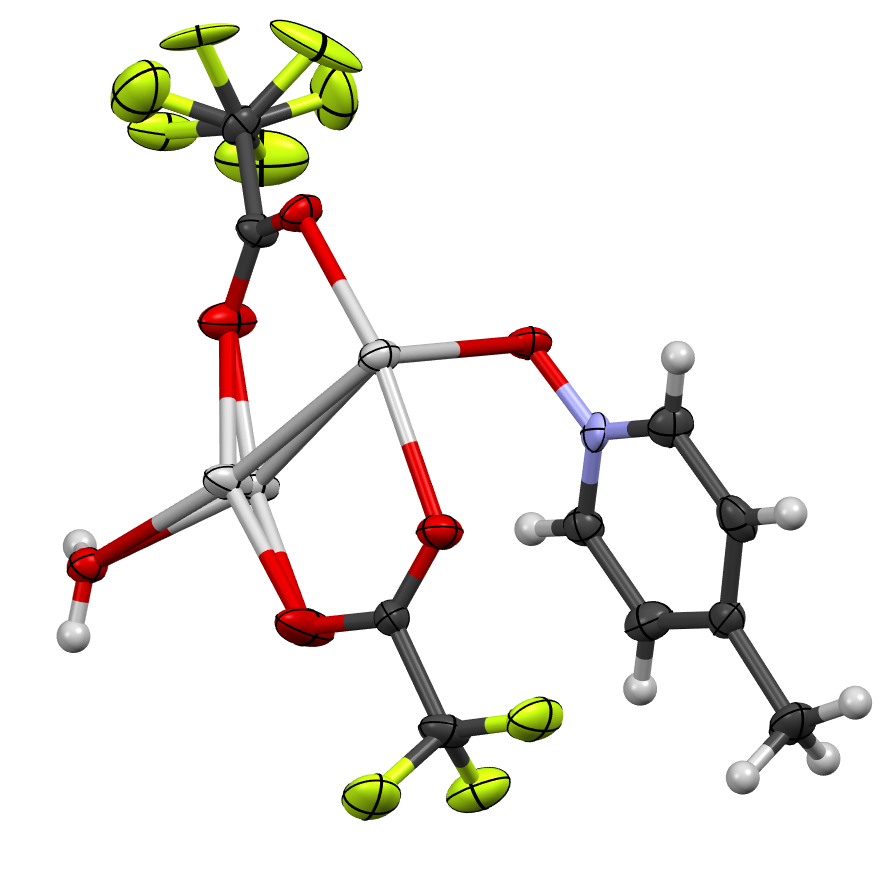


**Figure S73**. The asymmetric unit of **(4Ag)H_2_O_2** with the thermal displacement parameter at 50% probability level. Colour Key: green = fluorine, ash white = silver, blue = nitrogen, dark grey = carbon, white = hydrogen.

**Crystal data for (4Ag)H_2_O_2**: CCDC-2420929, C_10_H_9_Ag_2_F_6_NO_6_, M = 568.92 gmol^‑1^, colourless block, 0.11 × 0.1 × 0.05 mm^3^, triclinic, space group *P-1*, a = 7.11713(15) Å, b = 7.95327(14) Å, c = 13.8561(2) Å, α = 91.8415(14)°, β = 94.1133(16)°, γ = 104.2122(17)°, V = 757.35(3) Å^3^, Z = 2, D_calc_ = 2.495 gcm^‑3^, F(000) = 544, µ = 21.731 mm^‑1^, T = 120(1) K, θ_max_ = 66.728°, 8906 total reflections, 2493 with I_o_ > 2σ(I_o_), R_int_ = 0.0379, 2691 data, 272 parameters, 14 restraints, GooF = 1.035, R = 0.0646 and wR = 0.2057 [I_o_ > 2σ(I_o_)], R = 0.0668 and wR = 0.2089 (all reflections), 4.684 <d∆ρ < -0.966 eÅ^‑3^.

**17. Complex (4Ag)H_2_O_3**


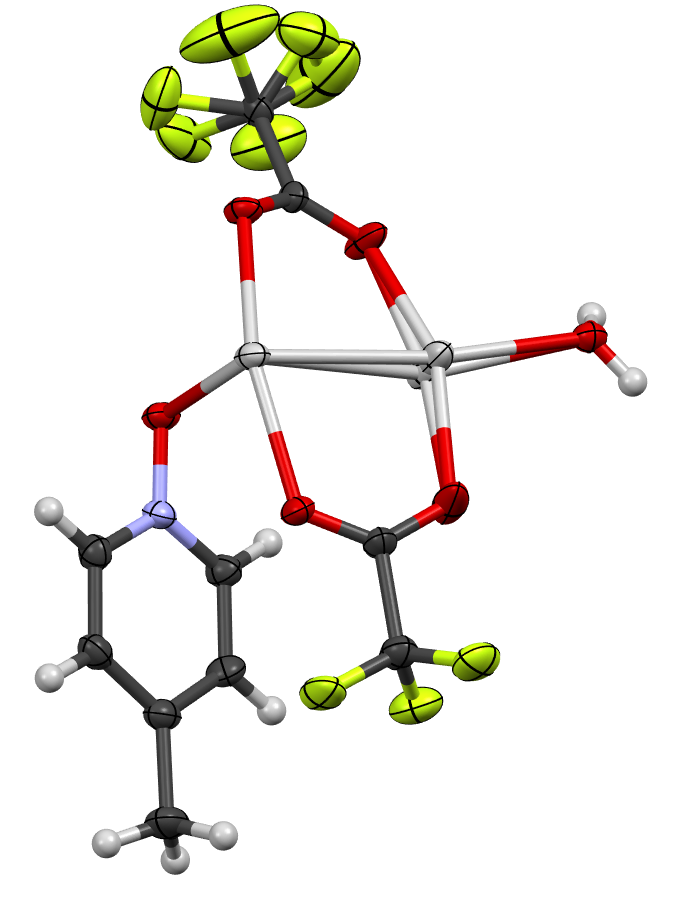


**Figure S74**. The asymmetric unit of **(4Ag)H_2_O_3** with the thermal displacement parameter at 50% probability level. Colour Key: green = fluorine, ash white = silver, blue = nitrogen, dark grey = carbon, white = hydrogen.

**Crystal data for (4Ag)H_2_O_3**: CCDC-2420937, C_20_H_17_Ag_4_F_14_N_2_O_12_, M = 1174.83 gmol^‑1^, colourless block, 0.19 × 0.16 × 0.04 mm^3^, triclinic, space group *P-1*, a = 7.1192(2) Å, b = 7.95340(10) Å, c = 13.8586(3) Å, α = 91.815(2)°, β = 94.056(2)°, γ = 104.276(2)°, V = 757.58(3) Å^3^, Z = 1, D_calc_ = 2.575 gcm^‑3^, F(000) = 561, µ = 21.856 mm^‑1^, T = 120(1) K, θ_max_ = 77.431°, 11421 total reflections, 3051 with I_o_ > 2σ(I_o_), R_int_ = 0.0535, 3163 data, 268 parameters, 2 restraints, GooF = 1.035, R = 0.0477 and wR = 0.1316 [I_o_ > 2σ(I_o_)], R = 0.0486 and wR = 0.1328 (all reflections), 1.599 <d∆ρ < -1.832 eÅ^‑3^.

**18. Complex (4Ag)H_2_O_4**


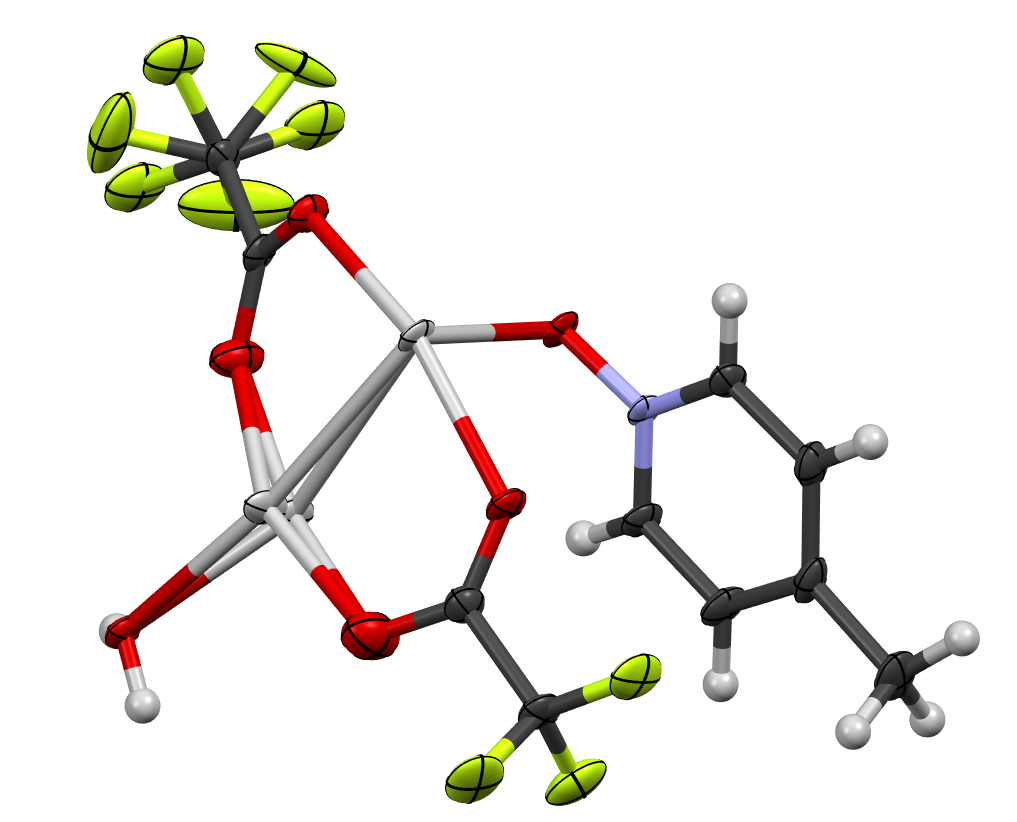


**Figure S75**. The asymmetric unit of **(4Ag)H_2_O_4** with the thermal displacement parameter at 50% probability level. Colour Key: green = fluorine, ash white = silver, blue = nitrogen, dark grey = carbon, white = hydrogen.

**Crystal data for (4Ag)H_2_O_4**: CCDC-2420939, C_10_H_9_Ag_2_F_6_NO_6_, M = 568.92 gmol^‑1^, colourless block, 0.18 × 0.18 × 0.02 mm^3^, triclinic, space group *P-1*, a = 7.1148(3) Å, b = 7.9590(3) Å, c = 13.8565(4) Å, α = 91.856(2)°, β = 93.974(3)°, γ = 104.307(4)°, V = 757.49(5) Å^3^, Z = 2, D_calc_ = 2.494 gcm^‑3^, F(000) = 544, µ = 21.727 mm^‑1^, T = 120(1) K, θ_max_ = 77.568°, 8665 total reflections, 2847 with I_o_ > 2σ(I_o_), R_int_ = 0.0562, 3133 data, 259 parameters, 50 restraints, GooF = 1.066, R = 0.0477 and wR = 0.1542 [I_o_ > 2σ(I_o_)], R = 0.0614 and wR = 0.1575 (all reflections), 1.471<d∆ρ < -4.393eÅ^‑3^.

**19. Complex (4Ag)H_2_O_5**


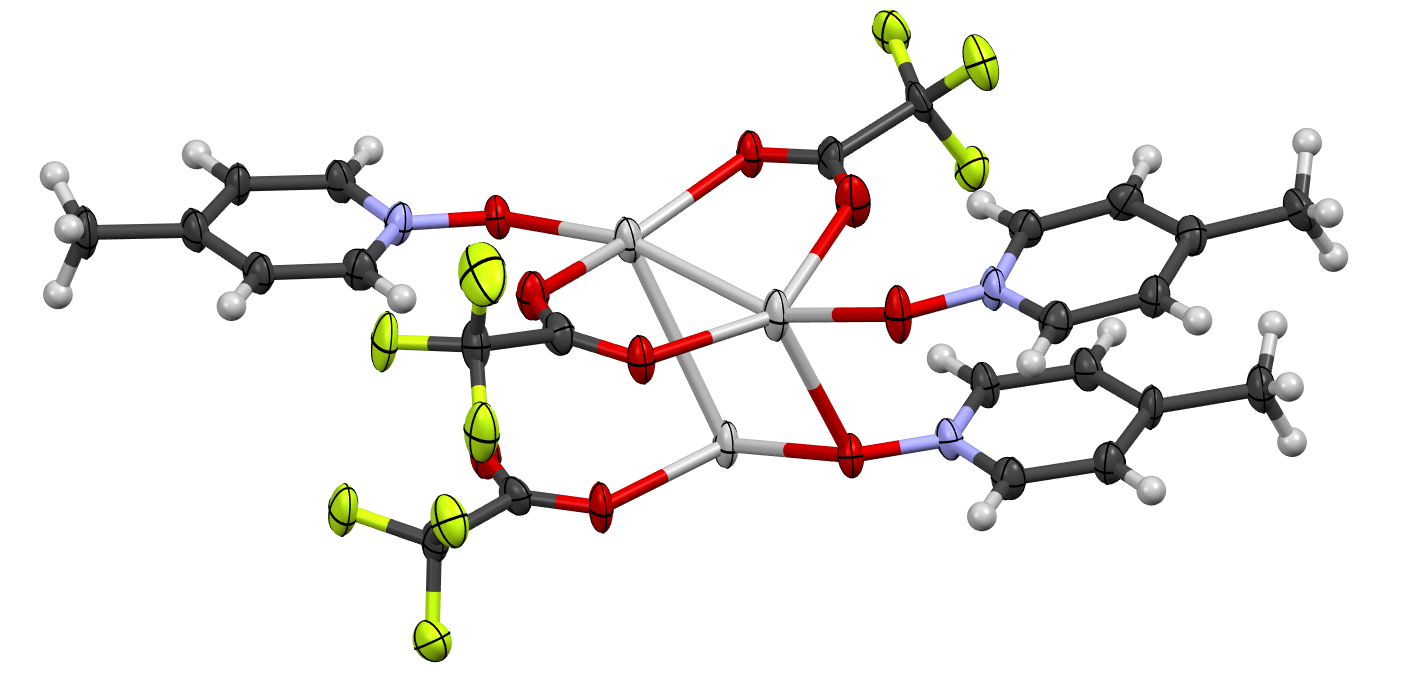


**Figure S76**. The asymmetric unit of **(4Ag)H_2_O_5** with the thermal displacement parameter at 50% probability level. Colour Key: green = fluorine, ash white = silver, blue = nitrogen, dark grey = carbon, white = hydrogen.

**Crystal data for (4Ag)H_2_O_5**: CCDC-2420941, C_48_H_42_Ag_6_F_18_N_6_O_18_, M = 1980.09 gmol^‑1^, colourless needle, 0.13 × 0.04 × 0.03 mm^3^, triclinic, space group *P-1*, a = 11.1686(8) Å, b = 11.2239(6) Å, c = 14.3218(6) Å, α = 108.773(4)°, β = 97.281(5)°, γ = 112.532(7)°, V = 1504.50(17) Å^3^, Z = 1, D_calc_ = 2.185 gcm^‑3^, F(000) = 960, µ = 16.551 mm^‑1^, T = 120(1) K, θ_max_ = 66.748°, 15237 total reflections, 4116 with I_o_ > 2σ(I_o_), R_int_ = 0.0775, 5271 data, 436 parameters, 0 restraints, GooF = 0.990, R = 0.0561 and wR = 0.1457 [I_o_ > 2σ(I_o_)], R = 0.0705 and wR = 0.1555 (all reflections), 2.250<d∆ρ < -1.647eÅ^‑3^.

**20. Complex (4Ag)H_2_O_a**


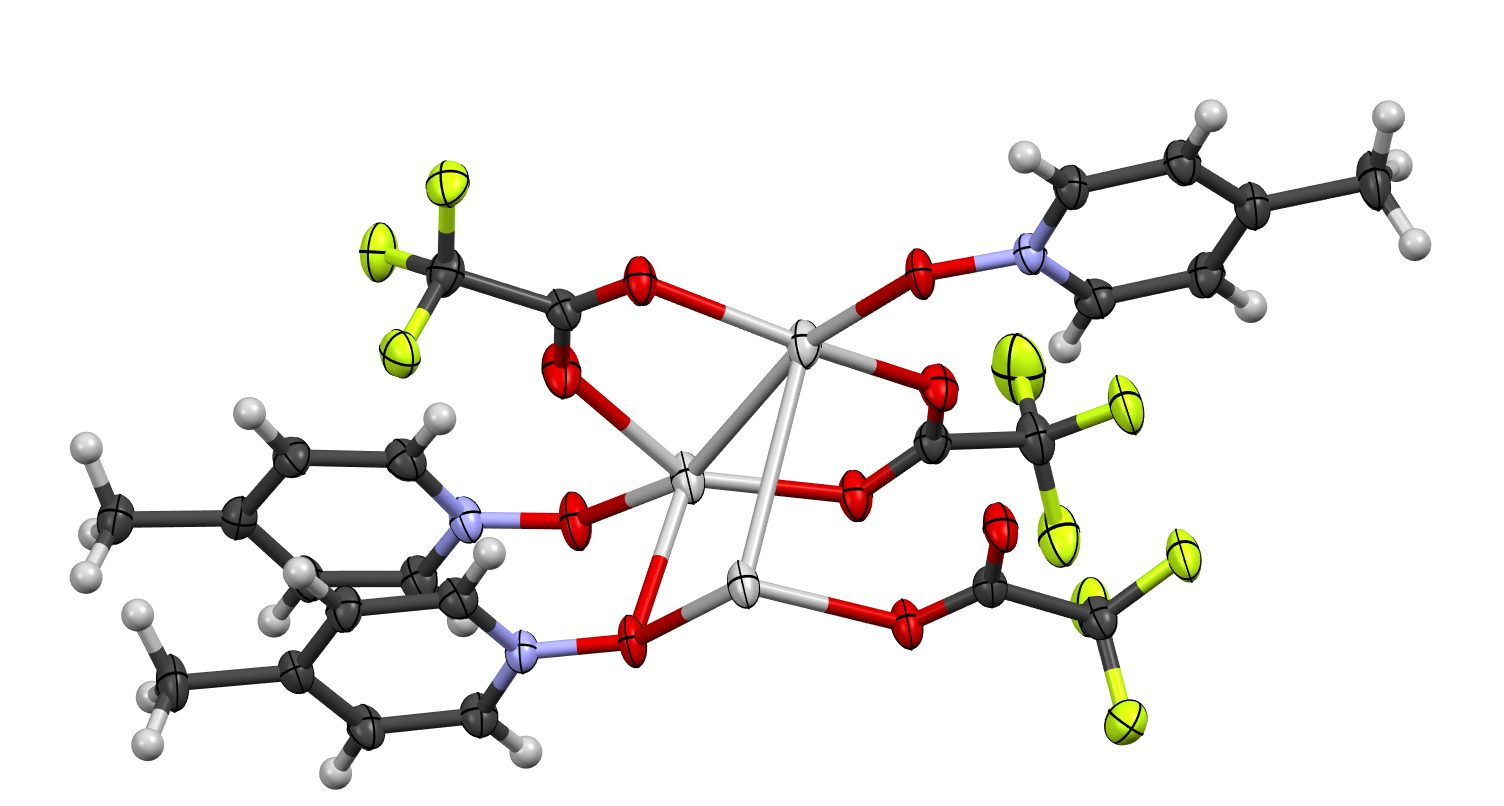


**Figure S77**. The asymmetric unit of **(4Ag)H_2_O_a** with the thermal displacement parameter at 50% probability level. Colour Key: green = fluorine, ash white = silver, blue = nitrogen, dark grey = carbon, white = hydrogen.

**Crystal data for (4Ag)H_2_O_a**: CCDC-2420945, C_48_H_42_Ag_6_F_18_N_6_O_18_, M = 1980.09 gmol^‑1^, colourless plate, 0.18 × 0.06 × 0.04 mm^3^, triclinic, space group *P-1*, a = 11.1393(4) Å, b = 11.2269(4) Å, c = 14.3271(3) Å, α = 108.791(2)°, β = 97.202(2)°, γ = 112.587(3)°, V = 1501.20(9) Å^3^, Z = 1, D_calc_ = 2.190 gcm^‑3^, F(000) = 960, µ = 16.588 mm^‑1^, T = 120(1) K, θ_max_ = 66.749°, 18994 total reflections, 4590 with I_o_ > 2σ(I_o_), R_int_ = 0.0644, 5322 data, 436 parameters, 0 restraints, GooF = 1.019, R = 0.0443 and wR = 0.1044 [I_o_ > 2σ(I_o_)], R = 0.0504 and wR = 0.1080 (all reflections), 2.213<d∆ρ < -1.752eÅ^‑3^.

**21. Complex (8Ag)H_2_O**


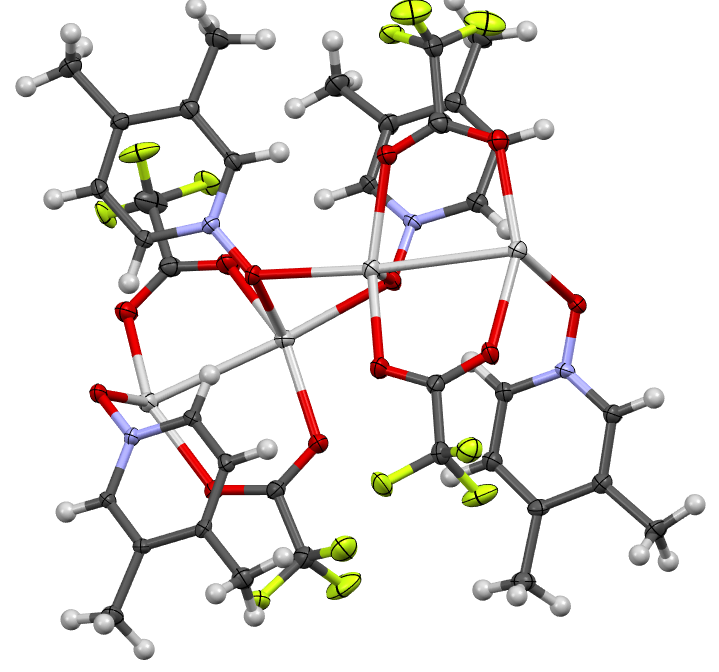


**Figure S78**. The asymmetric unit of **(8Ag)H_2_O** with the thermal displacement parameter at 50% probability level. Colour Key: green = fluorine, ash white = silver, blue = nitrogen, dark grey = carbon, white = hydrogen. Disordered TFA part is omitted for viewing clarity.

**Crystal data for (8Ag)H_2_O**: CCDC-2420963, C_36_H_36_Ag_4_F_12_N_4_O_12_, M = 1376.17 gmol^‑1^, colourless plate, 0.09 × 0.05 × 0.03 mm^3^, triclinic, space group *P-1*, a = 11.3149(4) Å, b = 11.4935(6) Å, c = 18.4400(8) Å, α = 89.795(4)°, β = 74.058(3)°, γ = 74.110(4)°, V = 2211.08(18) Å^3^, Z =2, D_calc_ = 2.067 gcm^‑3^, F(000) = 1344, µ = 15.049 mm^‑1^, T = 120(1) K, θ_max_ = 66.733°, 21518 total reflections, 5748 with I_o_ > 2σ(I_o_), R_int_ = 0.0585, 7601 data, 675 parameters, 30 restraints, GooF = 1.015, R = 0.0526 and wR = 0.1173 [I_o_ > 2σ(I_o_)], R = 0.0742 and wR = 0.1280 (all reflections), 1.105 <d∆ρ < -1.526 eÅ^‑3^.

**22. Complex (9Ag)Tol**


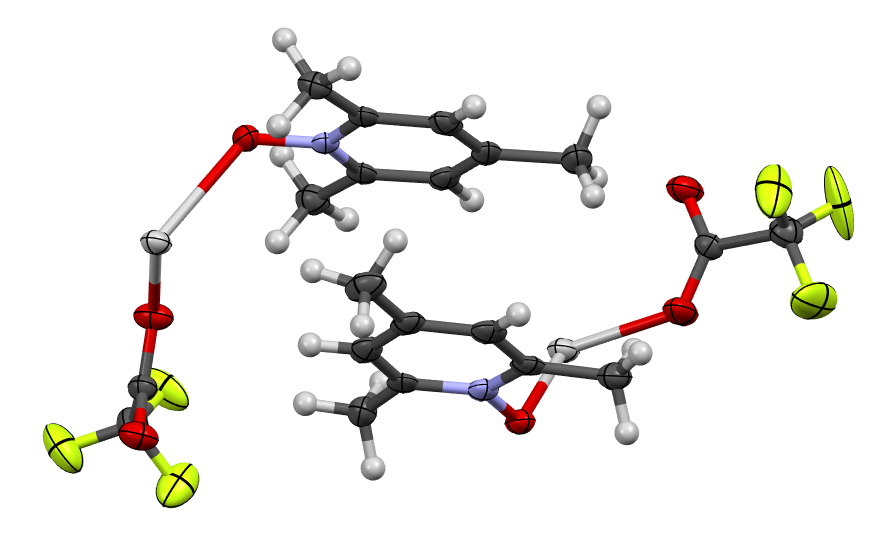


**Figure S79**. The asymmetric unit of **(9Ag)Tol** with the thermal displacement parameter at 50% probability level. Colour Key: green = fluorine, ash white = silver, blue = nitrogen, dark grey = carbon, white = hydrogen. Disordered TFA part is omitted for viewing clarity.

**Crystal data for (9Ag)Tol**: CCDC-2420955, C_10_H_11_AgF_3_NO_3_, M = 358.07 gmol^‑1^, colourless plates, 0.21 × 0.2 × 0.07 mm^3^, triclinic, space group *P-1*, a = 7.0173(3) Å, b = 11.2202(7) Å, c = 16.2482(4) Å, α = 85.928(4)°, β = 89.834(3)°, γ = 81.721(4)°, V = 1262.75(10)Å^3^, Z = 4, D_calc_ = 1.883 gcm^‑3^, F(000) = 704, µ = 13.205 mm^‑1^, T = 120(1) K, θ_max_ = 66.725°, 14903 total reflections, 3552 with I_o_ > 2σ(I_o_), R_int_ = 0.0630, 4477 data, 385 parameters, 0 restraints, GooF = 1.222, R = 0.1039 and wR = 0.2771 [I_o_ > 2σ(I_o_)], R = 0.1108 and wR = 0.2876 (all reflections), 6.927 <d∆ρ < -1.498 eÅ^‑3^.

**23. Complex (10Ag)EtOH**


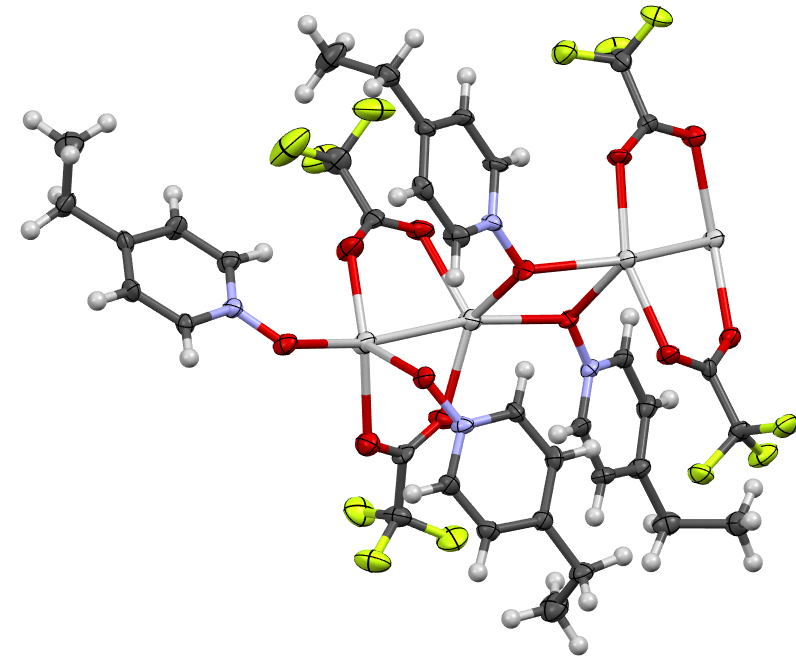


**Figure S80**. The asymmetric unit of **(10Ag)EtOH** with the thermal displacement parameter at 50% probability level. Colour Key: green = fluorine, ash white = silver, blue = nitrogen, dark grey = carbon, white = hydrogen.

**Crystal data for (10Ag)EtOH**: CCDC-2420960, C_36_H_36_Ag_4_F_12_N_4_O_12_, M = 1376.17 gmol^‑1^, colourless needle, 0.21 × 0.03 × 0.02 mm^3^, triclinic, space group *P-1*, a = 10.9749(6) Å, b = 14.1387(9) Å, c = 15.3885(7) Å, α = 91.639(4)°, β = 110.226(5) °, γ = 93.817(5) °, V = 2232.2(2) Å^3^, Z = 2, D_calc_ = 2.047 gcm^‑3^, F(000) = 1344, µ = 14.907 mm^‑1^, T = 120(1) K, θ_max_ = 66.748°, 25415 total reflections, 14356 with I_o_ > 2σ(I_o_), R_int_ = twinned, 25415 data, 618 parameters, 0 restraints, GooF = 1.045, R = 0.0980 and wR = 0.2560 [I_o_ > 2σ(I_o_)], R = 0.1585 and wR = 0.2897 (all reflections), 1.693 <d∆ρ < - -1.347 eÅ^‑3^.

**24. Complex (10Ag)H_2_O**


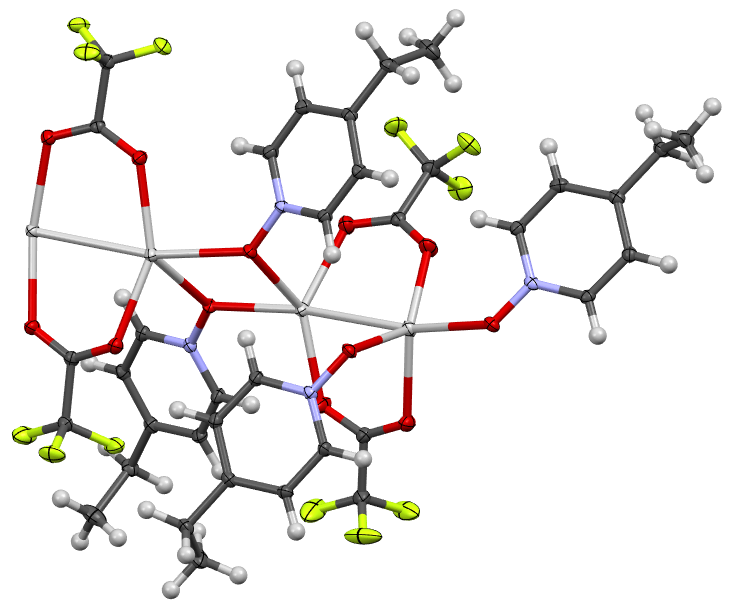


**Figure S81**. The asymmetric unit of **(10Ag)H_2_O** with the thermal displacement parameter at 50% probability level. Colour Key: green = fluorine, ash white = silver, blue = nitrogen, dark grey = carbon, white = hydrogen.

**Crystal data for (10Ag)H_2_O**: CCDC-2420967, C_36_H_36_Ag_4_F_12_N_4_O_12_, M = 1376.17 gmol^‑1^, colourless block, 0.08 × 0.07 × 0.05 mm^3^, triclinic, space group *P-1*, a = 11.00080(10) Å, b = 14.12270(10) Å, c = 15.3511(2) Å, α = 91.7680(10) °, β = 110.4130(10) °, γ = 93.7980(10) °, V = 2226.57(4) Å^3^, Z = 2, D_calc_ = 2.053 gcm^‑3^, F(000) = 1344, µ = 14.945 mm^‑1^, T = 120(1) K, θ_max_ = 66.749°, 31557 total reflections, 7304 with I_o_ > 2σ(I_o_), R_int_ = 0.0367, 7879 data, 617 parameters, 0 restraints, GooF = 1.040, R = 0.0239 and wR = 0.0563 [I_o_ > 2σ(I_o_)], R = 0.0264 and wR = 0.0574 (all reflections), 1.253 <d∆ρ < -0.630 eÅ^‑3^.

**25. Complex (10Ag)H_2_O_2**


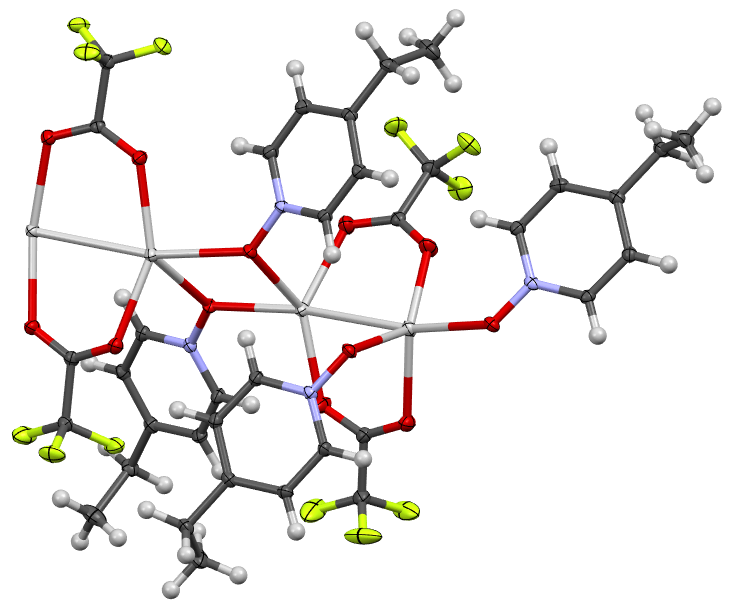


**Figure S82**. The asymmetric unit of **(10Ag)H_2_O_2** with the thermal displacement parameter at 50% probability level. Colour Key: green = fluorine, ash white = silver, blue = nitrogen, dark grey = carbon, white = hydrogen.

**Crystal data for (10Ag)H_2_O_2**: CCDC-2420964, C_36_H_36_Ag_4_F_12_N_4_O_12_, M = 1376.17 gmol^‑1^, colourless plate, 0.24 × 0.07 × 0.05 mm^3^, triclinic, space group *P-1*, a = 11.0125(2) Å, b = 14.1534(2) Å, c = 15.3855(3) Å, α = 91.6880(10) °, β = 110.343(2) °, γ = 93.7490(10) °, V = 2240.17(7) Å^3^, Z = 2, D_calc_ = 2.040 gcm^‑3^, F(000) = 1344, µ = 14.854 mm^‑1^, T = 120(1) K, θ_max_ = 66.746°, 30842 total reflections, 7599 with I_o_ > 2σ(I_o_), R_int_ = 0.0292, 7929 data, 644 parameters, 12 restraints, GooF = 1.041, R = 0.0211 and wR = 0.0520 [I_o_ > 2σ(I_o_)], R = 0.0223 and wR = 0.0525 (all reflections), 0.636 <d∆ρ < -0.551 eÅ^‑3^.

**26. Complex (11Ag)EtOAc**


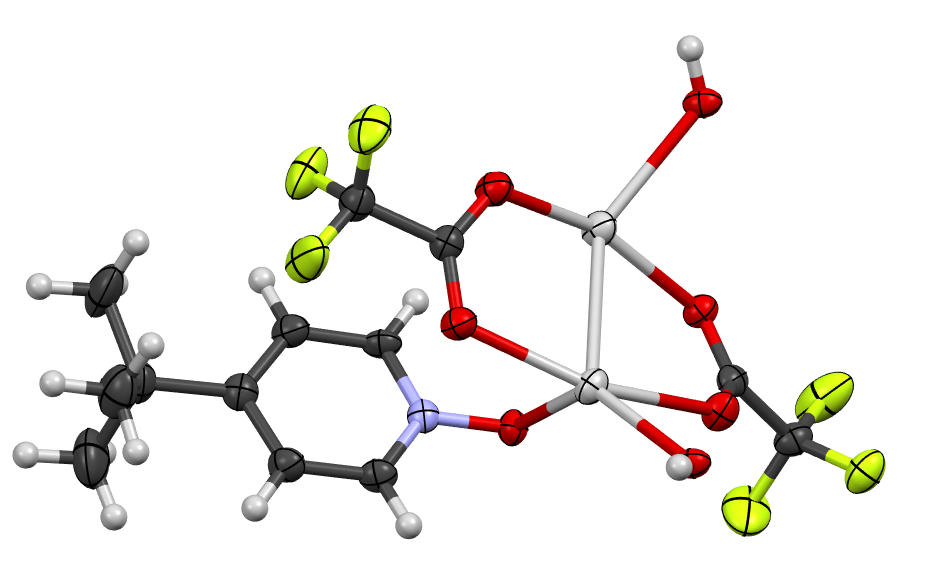


**Figure S83**. The asymmetric unit of **(11Ag)EtOAc** with the thermal displacement parameter at 50% probability level. Colour Key: green = fluorine, ash white = silver, blue = nitrogen, dark grey = carbon, white = hydrogen.

**Crystal data for (11Ag)EtOAc**: CCDC-2420959, C_13_H_15_Ag_2_F_6_NO_6_, M = 611.00 gmol^‑1^, colourless plate, 0.17 × 0.11 × 0.02 mm^3^, monoclinic, space group *C2/c*, a = 34.0400(6) Å, b = 10.0250(2) Å, c = 10.7599(2) Å, α = 90°, β = 90.462(2)°, γ = 90°, V = 3671.71(12) Å^3^, Z = 8, D_calc_ = 2.211 gcm^‑3^, F(000) = 2368, µ = 17.989 mm^‑1^, T = 120(1) K, θ_max_ = 66.733°, 25147 total reflections, 2985 with I_o_ > 2σ(I_o_), R_int_ = 0.0626, 3249 data, 265 parameters, 2 restraints, GooF = 1.057, R = 0.0335 and wR = 0.0895 [I_o_ > 2σ(I_o_)], R = 0.0357 and wR = 0.0915 (all reflections), 0.516 <d∆ρ < -1.179 eÅ^‑3^.

**27. Complex (11Ag)EtOH**


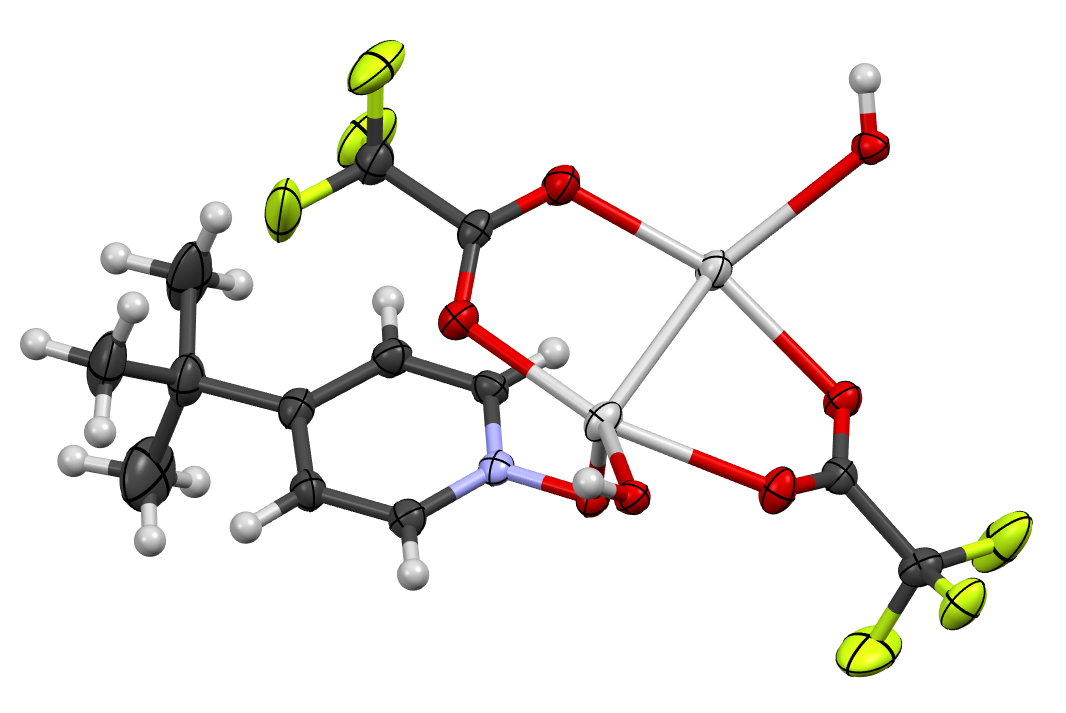


**Figure S84**. The asymmetric unit of **(11Ag)EtOH** with the thermal displacement parameter at 50% probability level. Colour Key: green = fluorine, ash white = silver, blue = nitrogen, dark grey = carbon, white = hydrogen.

**Crystal data for (11Ag)EtOH**: CCDC-2420962, C_13_H_15_Ag_2_F_6_NO_6_, M = 611.00 gmol^‑1^, colourless plate, 0.29 × 0.13 × 0.04 mm^3^, monoclinic, space group *C2/c*, a = 34.0523(3) Å, b = 10.03040(10) Å, c = 10.79540(10) Å, α = 90°, β = 90.4240(10)°, γ = 90°, V = 3687.16(6) Å^3^, Z = 8, D_calc_ = 2.201 gcm^‑3^, F(000) = 2368, µ = 17.914 mm^‑1^, T = 150(1) K, θ_max_ = 66.724 °, 26818 total reflections, 3175 with I_o_ > 2σ(I_o_), R_int_ = 0.0496, 3265 data, 265 parameters, 2 restraints, GooF = 1.090, R = 0.0242 and wR = 0.0647 [I_o_ > 2σ(I_o_)], R = 0.0249 and wR = 0.0652 (all reflections), 0.711 <d∆ρ < -0.659 eÅ^‑3^.

**28. Complex (11Ag)H_2_O**


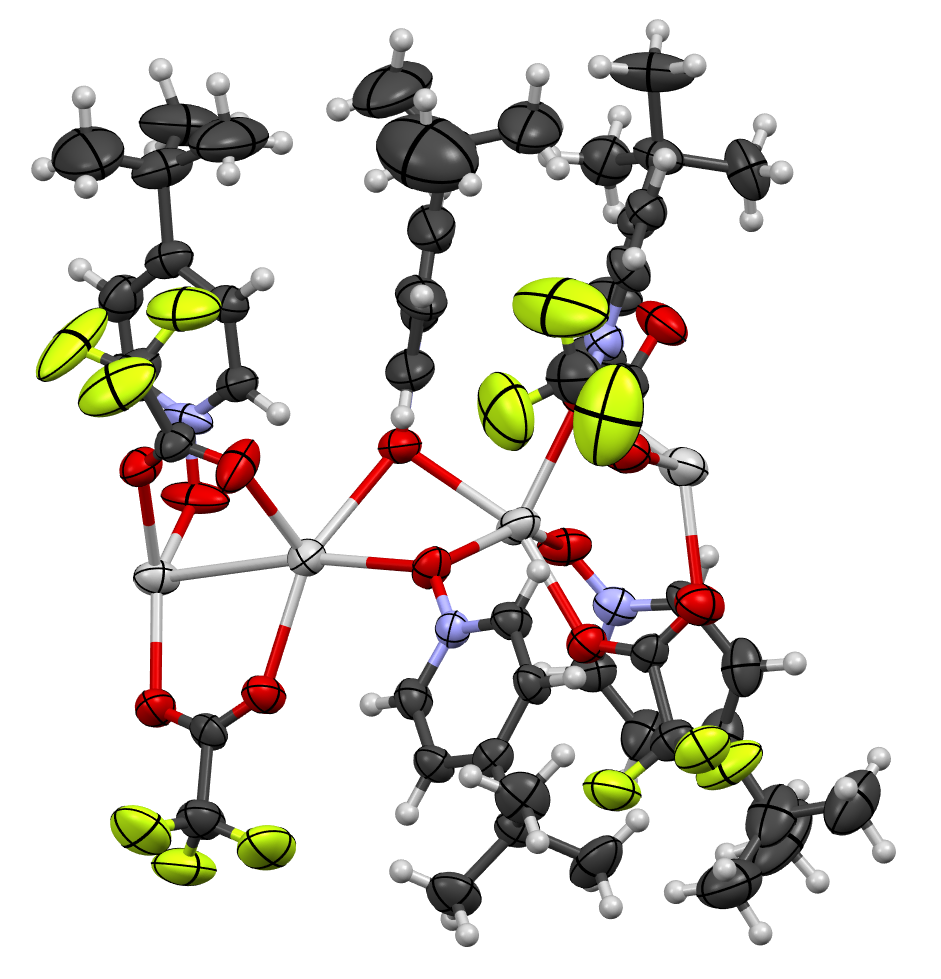


**Figure S85**. The asymmetric unit of **(11Ag)H_2_O** with the thermal displacement parameter at 50% probability level. Colour Key: green = fluorine, ash white = silver, blue = nitrogen, dark grey = carbon, white = hydrogen.

**Crystal data for (11Ag)H_2_O**: CCDC-2420965, C_53_H_65_Ag_4_F_12_N5O_13_, M = 1639.58 gmol^‑1^, colourless plate, 0.11 × 0.07 × 0.02 mm^3^, monoclinic, space group *P2_1_/n*, a = 15.4109(5) Å, b = 17.2367(6) Å, c = 24.7454(6) Å, α = 90°, β = 102.949(3)°, γ = 90°, V = 3687.16(6) Å^3^, Z = 8, D_calc_ = 1.700 gcm^‑3^, F(000) = 3272, µ = 10.515 mm^‑1^, T = 150(1) K, θ_max_ = 66.745°, 44220 total reflections, 7869 with I_o_ > 2σ(I_o_), R_int_ = 0.0934, 11259 data, 799 parameters, 24 restraints, GooF = 1.067, R = 0.0617 and wR = 0.1576 [I_o_ > 2σ(I_o_)], R = 0.0892 and wR = 0.1733 (all reflections), 1.646 <d∆ρ < -1.478 eÅ^‑3^.

**29. Complex (11Ag)Tol**


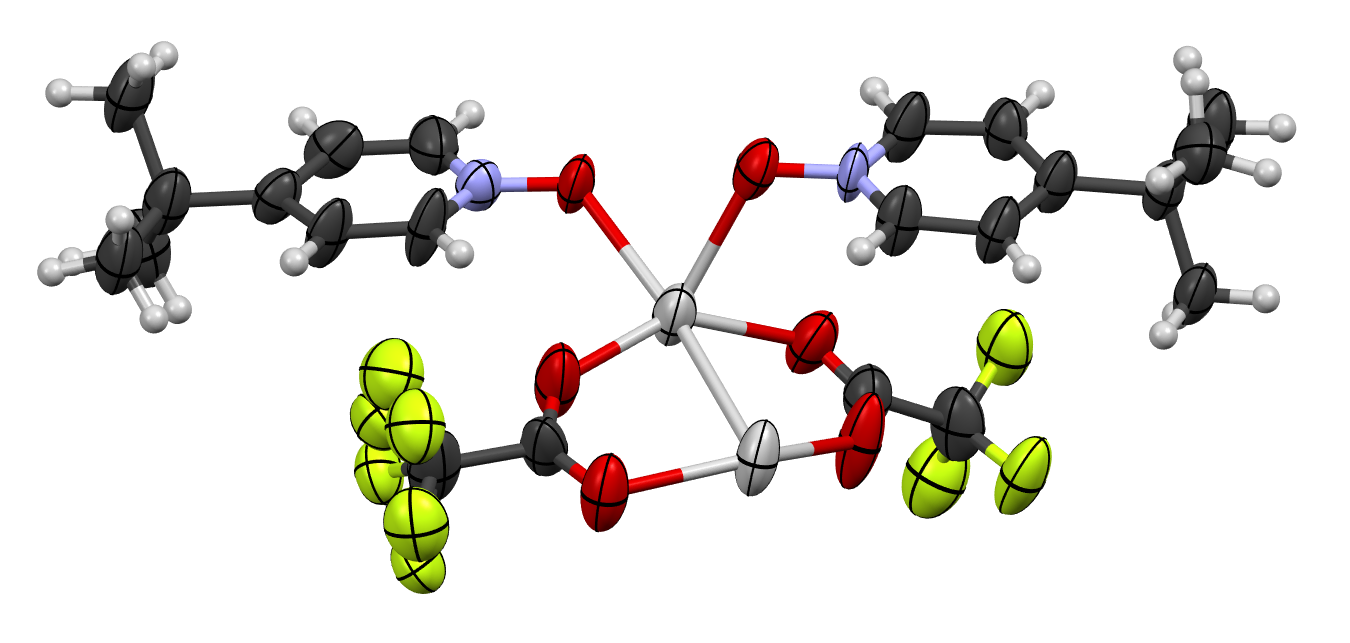


**Figure S86**. The asymmetric unit of **(11Ag)Tol** with the thermal displacement parameter at 50% probability level. Colour Key: green = fluorine, ash white = silver, blue = nitrogen, dark grey = carbon, white = hydrogen.

**Crystal data for (11Ag)Tol**: CCDC-2420968, C_22_H_26_Ag_2_F_6_N_2_O_6_, M = 744.19 gmol^‑1^, colourless needle, 0.20 × 0.08 × 0.02 mm^3^, monoclinic, space group *P2_1_/c*, a = 15.5001(8)Å, b = 18.740(2)Å, c = 11.0170(5)Å, α = 90°, β = 106.597(5)°, γ = 90°, V = 3066.9(4) Å^3^, Z = 4, D_calc_ = 1.612 gcm^‑3^, F(000) = 1472, µ = 10.898 mm^‑1^, T = 120(1) K, θ_max_ = 66.746°, 29258 total reflections, 3229 with I_o_ > 2σ(I_o_), R_int_ = 0.1888, 5432 data, 376 parameters, 48 restraints, GooF = 1.088, R = 0.0987 and wR = 0.2796 [I_o_ > 2σ(I_o_)], R = 0.1389and wR = 0.3152 (all reflections), 2.383<d∆ρ < -1.715eÅ^‑3^.

**30. Complex (13Ag)Acet**


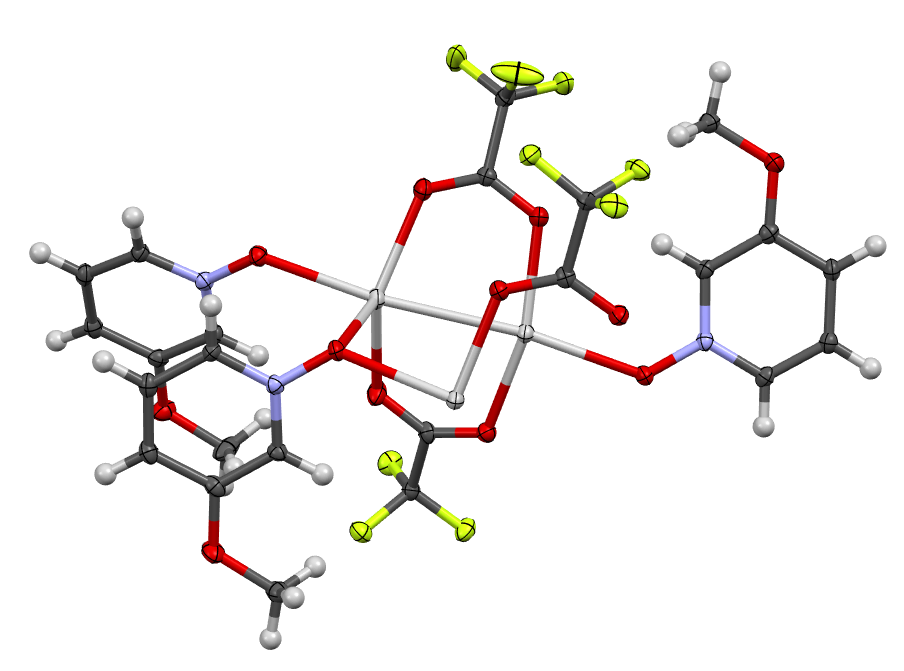


**Figure S87**. The asymmetric unit of **(13Ag)Acet** with the thermal displacement parameter at 50% probability level. Colour Key: green = fluorine, ash white = silver, blue = nitrogen, dark grey = carbon, white = hydrogen.

**Crystal data for (13Ag)Acet**: CCDC-2420956, C_48_H_42_Ag_6_F_18_N_6_O_24_, M = 2076.09 gmol^‑1^, colourless needle, 0.12 × 0.1 × 0.04 mm, triclinic, space group *P-1*, a = 10.8836(3) Å, b = 11.6714(2) Å, c = 12.5081(3) Å, α = 94.104(2) °, β = 103.839(2)°, γ = 99.049(2) °, V = 1513.63(6) Å^3^, Z = 1, D_calc_ = 2.278 gcm^‑3^, F(000) = 1008, µ = 16.572 mm^‑1^, T = 120(1) K, θ_max_ = 66.731°, 19251 total reflections, 4863 with I_o_ > 2σ(I_o_), R_int_ = 0.0401, 5344 data, 463 parameters, 0 restraints, GooF = 1.058, R = 0.0353 and wR = 0.0965 [I_o_ > 2σ(I_o_)], R = 0.0387 and wR = 0.1009 (all reflections), 1.112 <d∆ρ <-1.668 eÅ^‑3^.

**31. Complex (13Ag)EtOAc_a**


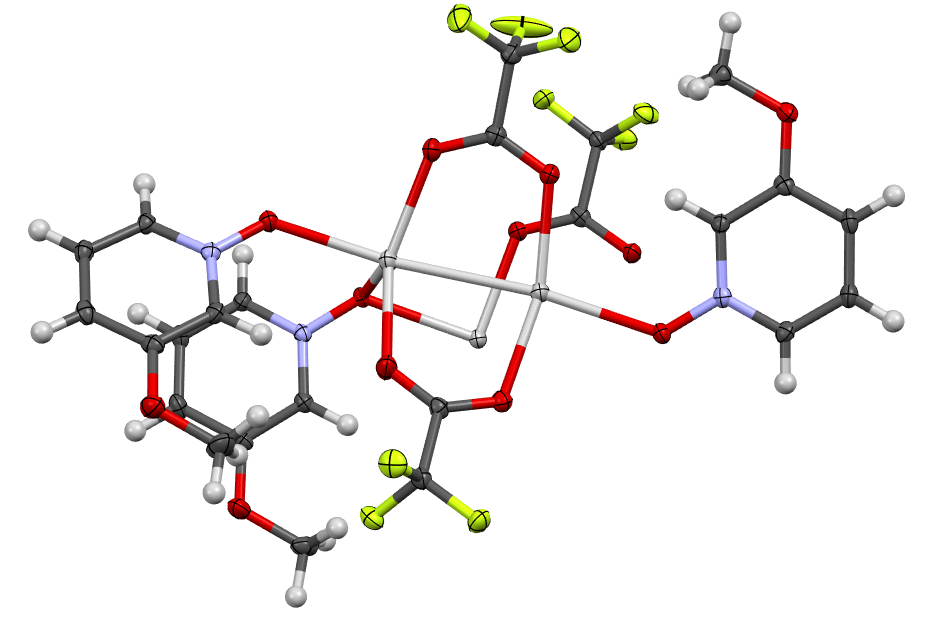


**Figure S88**. The asymmetric unit of **(13Ag)EtOAc_a** with the thermal displacement parameter at 50% probability level. Colour Key: green = fluorine, ash white = silver, blue = nitrogen, dark grey = carbon, white = hydrogen.

**Crystal data for (13Ag)EtOAc_a**: CCDC-2420958, C_48_H_42_Ag_6_F_18_N_6_O_24_, M = 2076.09 gmol^‑1^, colourless plates, 0.26 × 0.15 × 0.04 mm, triclinic, space group *P-1*, a = 10.9196(2) Å, b = 11.6797(2) Å, c = 12.5422(3) Å, α = 94.122(2) °, β = 103.968(2) °, γ = 99.123(2) °, V = 1522.50(6) Å^3^, Z = 1, D_calc_ = 2.264 gcm^‑3^, F(000) = 1008, µ = 16.476 mm^‑1^, T = 150(1) K, θ_max_ = 66.747°, 20440 total reflections, 5013 with I_o_ > 2σ(I_o_), R_int_ = 0.0516, 5378 data, 463 parameters, 0 restraints, GooF = 1.037, R = 0.0365 and wR = 0.0983 [I_o_ > 2σ(I_o_)], R = 0.0385 and wR = 0.1008 (all reflections), 1.735 <d∆ρ <-0.952 eÅ^‑3^.

**32. Complex (13Ag)Tol**


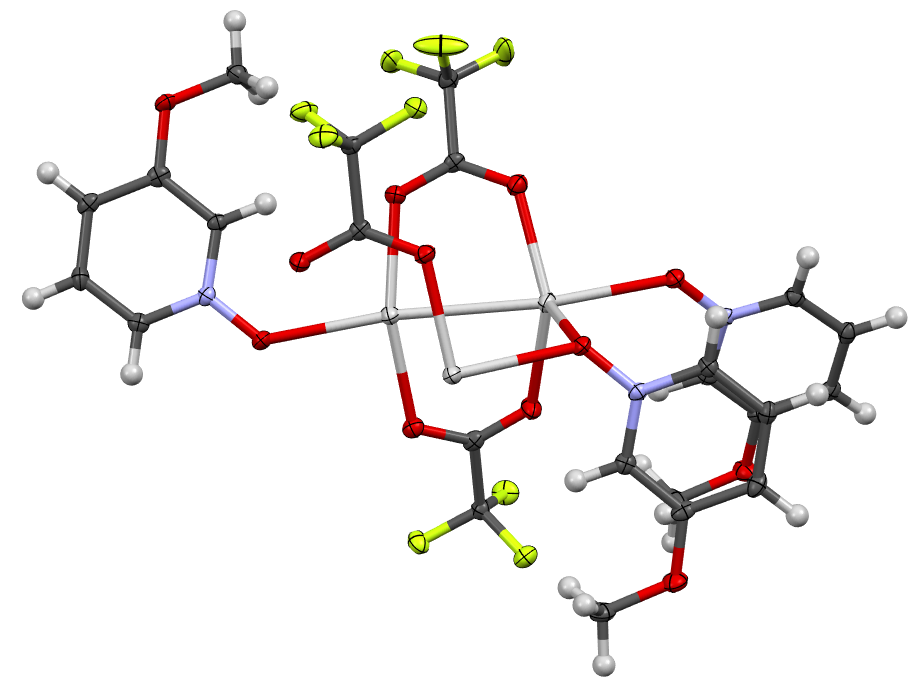


**Figure S89**. The asymmetric unit of **(13Ag)Tol** with the thermal displacement parameter at 50% probability level. Colour Key: green = fluorine, ash white = silver, blue = nitrogen, dark grey = carbon, white = hydrogen.

**Crystal data for (13Ag)Tol**: CCDC-2420961, C_48_H_42_Ag_6_F_18_N_6_O_24_, M = 2076.09 gmol^‑1^, colourless needle, 0.25 × 0.08 × 0.06 mm, triclinic, space group *P-1*, a = 10.8842(5) Å, b = 11.6686(4) Å, c = 12.5143(3) Å, α = 94.055(2) °, β = 103.880(3)°, γ = 98.997(3)°, V = 1514.23(10) Å^3^, Z = 1, D_calc_ = 2.277 gcm^‑3^, F(000) = 1008, µ = 16.566 mm^‑1^, T = 120(1) K, θ_max_ = 66.744°, 18939 total reflections, 4819 with I_o_ > 2σ(I_o_), R_int_ = 0.0660, 5349 data, 463 parameters, 0 restraints, GooF = 1.047, R = 0.0411 and wR = 0.1085 [I_o_ > 2σ(I_o_)], R = 0.0445 and wR = 0.1124 (all reflections), 1.198 <d∆ρ < -1.217 eÅ^‑3^.

**33. Complex (14Ag)Ben**


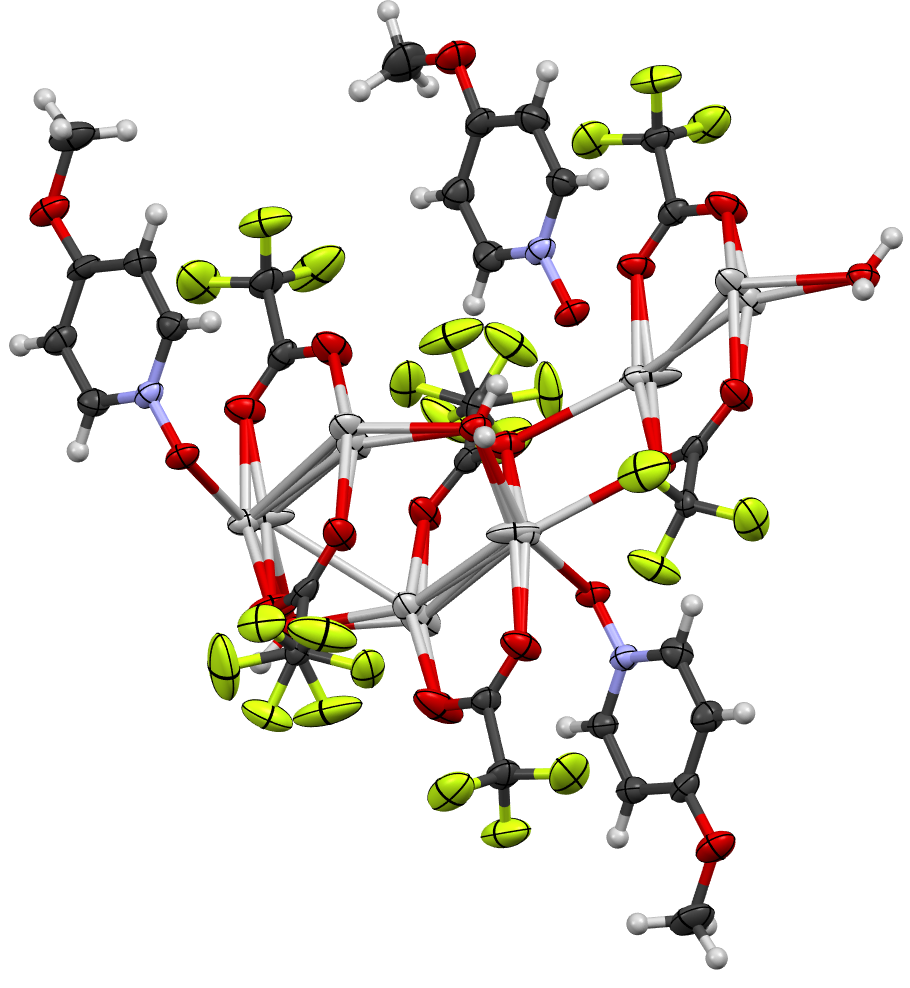


**Figure S90**. The asymmetric unit of **(14Ag)Ben** with the thermal displacement parameter at 50% probability level. Colour Key: green = fluorine, ash white = silver, blue = nitrogen, dark grey = carbon, white = hydrogen.

**Crystal data for (14Ag)Ben**: CCDC-2420969, C_30_H_27_Ag_6_F_18_N_3_O_21_, M = 1754.76 gmol^‑1^, colourless needle, 0.17 × 0.08 × 0.04 mm, monoclinic, space group *P2_1_/n*, a = 19.2285(2) Å, b = 9.63860(10) Å, c = 25.3545(3) Å, α = 90 °, β = 95.6690(10) °, γ = 90 °, V = 4676.11(9) Å^3^, Z = 4, D_calc_ = 2.493 gcm^‑3^, F(000) = 3360, µ = 21.196 mm^‑1^, T = 120(1) K, θ_max_ = 66.746°, 40493 total reflections, 6983 with I_o_ > 2σ(I_o_), R_int_ = 0.0675, 8298 data, 832 parameters, 65 restraints, GooF = 1.070, R = 0.0413 and wR = 0.1134 [I_o_ > 2σ(I_o_)], R = 0.0481 and wR = 0.1177 (all reflections), 0.771 <d∆ρ < -1.293 eÅ^‑3^.

**34. Complex (14Ag)EtOAc_a**


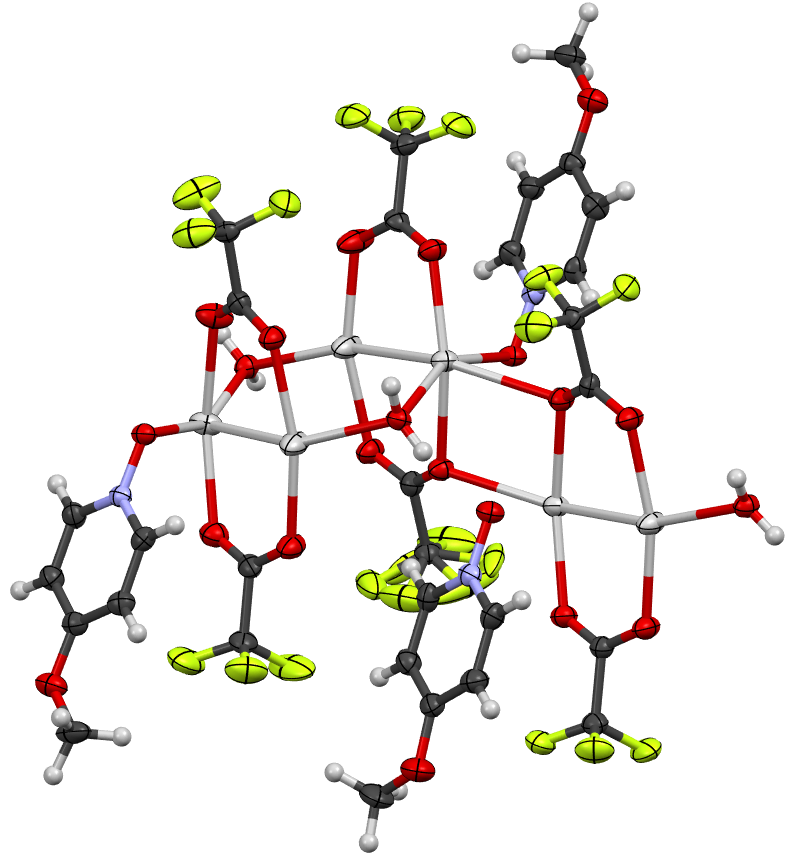


**Figure S91**. The asymmetric unit of **(14Ag)EtOAc_a** with the thermal displacement parameter at 50% probability level. Colour Key: green = fluorine, ash white = silver, blue = nitrogen, dark grey = carbon, white = hydrogen.

**Crystal data for (14Ag)EtOAc_a**: CCDC-2420966, C_48_H_42_Ag_6_F_18_N_6_O_24_, M = 2076.09 gmol^‑1^, colourless plate, 0.35 × 0.11 × 0.03 mm, monoclinic, space group *P2_1_/n*, a = 19.1575(2) Å, b = 9.59980(10) Å, c = 25.4337(3) Å, α = 90°, β = 95.7290(10) °, γ = 90°, V = 4654.10(9) Å^3^, Z = 1, D_calc_ = 2.504 gcm^‑3^, F(000) = 3360, µ = 21.296 mm^‑1^, T = 120(1) K, θ_max_ = 76.880 °, 52481 total reflections, 8471 with I_o_ > 2σ(I_o_), R_int_ = 0.0695, 9634 data, 751 parameters, 6 restraints, GooF = 1.047, R = 0.0632 and wR = 0.1823 [I_o_ > 2σ(I_o_)], R = 0.0680 and wR = 0.1884 (all reflections), 4.291 <d∆ρ < -2.787 eÅ^‑3^.

**35. Complex (15Ag)Acet**


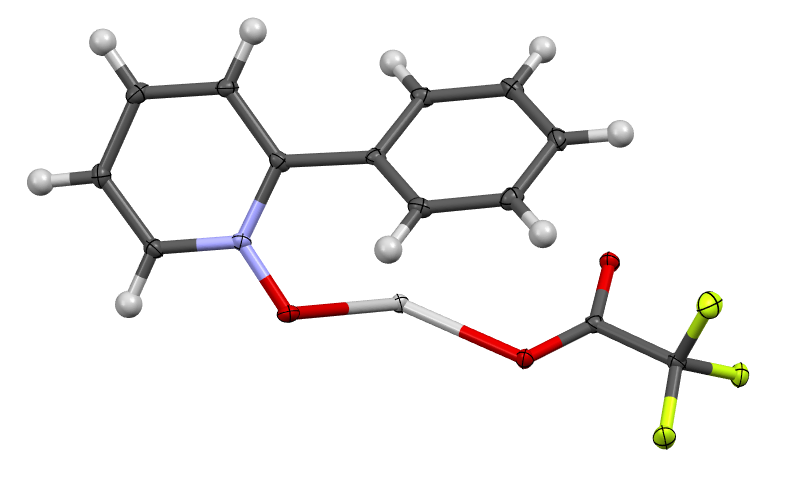


**Figure S92**. The asymmetric unit of **(15Ag)Acet** with the thermal displacement parameter at 50% probability level. Colour Key: green = fluorine, ash white = silver, blue = nitrogen, dark grey = carbon, white = hydrogen.

**Crystal data for (15Ag)Acet**: CCDC-2420951, C_13_H_9_AgF_3_NO_3_, M = 392.08 gmol^‑1^, colourless needle, 0.44 × 0.08 × 0.03 mm, triclinic, space group *P-1*, a = 5.90600(10) Å, b = 10.7288(3) Å, c = 11.5271(3) Å, α = 64.306(2) °, β = 80.323(2) °, γ = 74.078(2) °, V = 631.92(3) Å^3^, Z = 2, D_calc_ = 2.061 gcm^‑3^, F(000) = 384, µ = 13.278 mm^‑1^, T = 120(1) K, θ_max_ = 77.654 °, 7348 total reflections, 2543 with I_o_ > 2σ(I_o_), R_int_ = 0.0478, 2637 data, 190 parameters, 0 restraints, GooF = 1.052, R = 0.0345 and wR = 0.0891 [I_o_ > 2σ(I_o_)], R = 0.0352 and wR = 0.0898 (all reflections), 1.152 <d∆ρ < -1.172 eÅ^‑3^.

**36. Complex (15Ag)EtOAc**


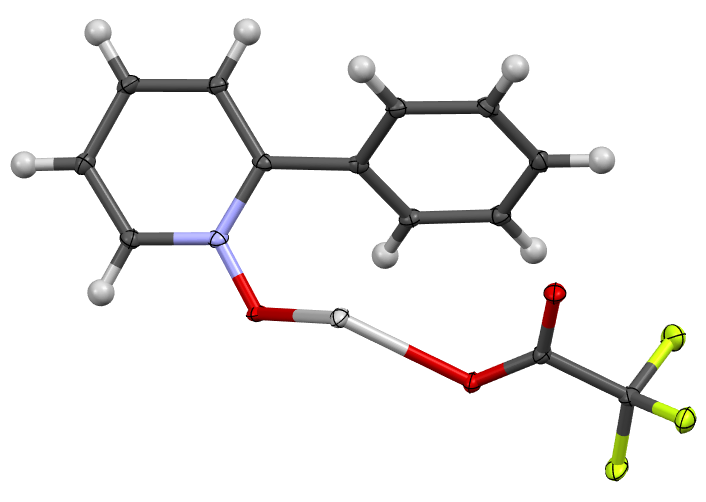


**Figure S93**. The asymmetric unit of **(15Ag)EtOAc** with the thermal displacement parameter at 50% probability level. Colour Key: green = fluorine, ash white = silver, blue = nitrogen, dark grey = carbon, white = hydrogen.

**Crystal data for (15Ag)EtOAc**: CCDC-2420953, C_13_H_9_AgF_3_NO_3_, M = 392.08 gmol^‑1^, colourless needle, 0.38 × 0.04 × 0.03 mm, triclinic, space group *P-1*, a = 5.9018(2) Å, b = 10.7295(4) Å, c = 11.5360(4) Å, α = 64.325(4)°, β = 80.308(3) °, γ = 74.061(3) °, V = 632.03(4) Å^3^, Z = 2, D_calc_ = 2.060 gcm^‑3^, F(000) = 384, µ = 13.276 mm^‑1^, T = 120(1) K, θ_max_ = 76.196°, 7970 total reflections, 2498 with I_o_ > 2σ(I_o_), R_int_ = 0.0385, 2613 data, 190 parameters, 0 restraints, GooF = 1.072, R = 0.0244 and wR = 0.0646 [I_o_ > 2σ(I_o_)], R = 0.0254 and wR = 0.0651 (all reflections), 0.449 <d∆ρ < -1.003 eÅ^‑3^.

**37. Complex (15Ag)EtOAc_a**


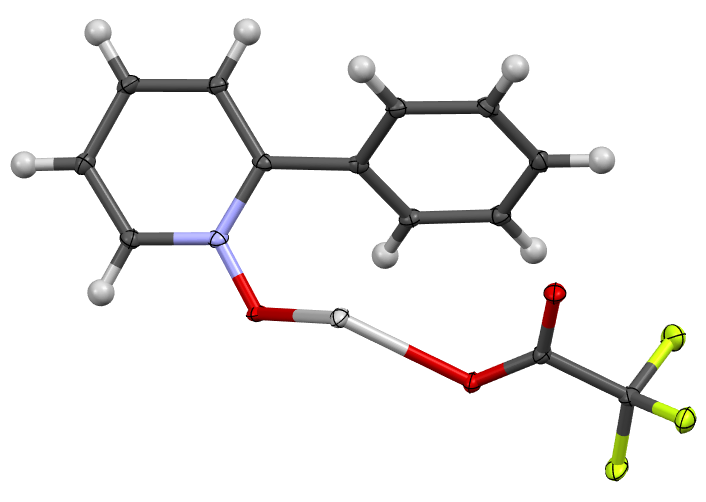


**Figure S94**. The asymmetric unit of  **(15Ag)EtOAc_a** with the thermal displacement parameter at 50% probability level. Colour Key: green = fluorine, ash white = silver, blue = nitrogen, dark grey = carbon, white = hydrogen.

**Crystal data for (15Ag)EtOAc_a**: CCDC-2420957, C_13_H_9_AgF_3_NO_3_, M = 392.08 gmol^‑1^, colourless needle, 0.29 × 0.04 × 0.02 mm, triclinic, space group *P-1*, a = 5.9062(3) Å, b = 10.7238(7) Å, c = 11.5295(8) Å, α = 64.261(6)°, β = 80.360(5)°, γ = 74.075(5)°, V = 631.56(8) Å^3^, Z = 2, D_calc_ = 2.062 gcm^‑3^, F(000) = 384, µ = 13.286 mm^‑1^, T = 120(1) K, θ_max_ = 66.712°, 6843 total reflections, 1991 with I_o_ > 2σ(I_o_), R_int_ = 0.0878, 2224 data, 190 parameters, 0 restraints, GooF = 1.108, R = 0.0648 and wR = 0.1647 [I_o_ > 2σ(I_o_)], R = 0.0727 and wR = 0.1910 (all reflections), 1.853 <d∆ρ < -3.433 eÅ^‑3^.

**38. Complex (15Ag)EtOH**


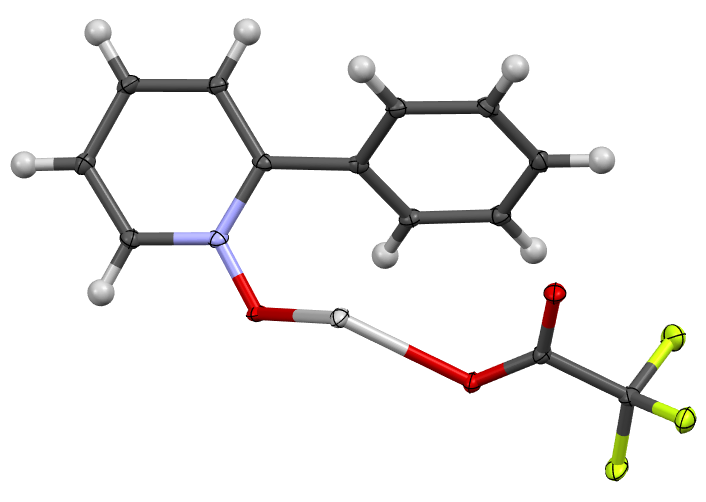


**Figure S95**. The asymmetric unit of  **(15Ag)EtOH** with the thermal displacement parameter at 50% probability level. Colour Key: green = fluorine, ash white = silver, blue = nitrogen, dark grey = carbon, white = hydrogen.

**Crystal data for (15Ag)EtOH**: CCDC-2420950, C_13_H_9_AgF_3_NO_3_, M = 392.08 gmol^‑1^, colourless plate, 0.16 × 0.11 × 0.03 mm, triclinic, space group *P-1*, a = 5.9098(2) Å, b = 10.7220(3) Å, c = 11.5220(2) Å, α = 64.288(2) °, β = 80.344(2) °, γ = 74.073(2) °, V = 631.55(3) Å^3^, Z = 2, D_calc_ = 2.062 gcm^‑3^, F(000) = 384, µ = 13.286 mm^‑1^, T = 120(1) K, θ_max_ = 66.718°, 7079 total reflections, 2172 with I_o_ > 2σ(I_o_), R_int_ = 0.0347, 2233 data, 190 parameters, 0 restraints, GooF = 1.027, R = 0.0218 and wR = 0.0560 [I_o_ > 2σ(I_o_)], R = 0.0224 and wR = 0.0563 (all reflections), 0.517 <d∆ρ <-0.492 eÅ^‑3^.

**39. Complex (15Ag)H_2_O**


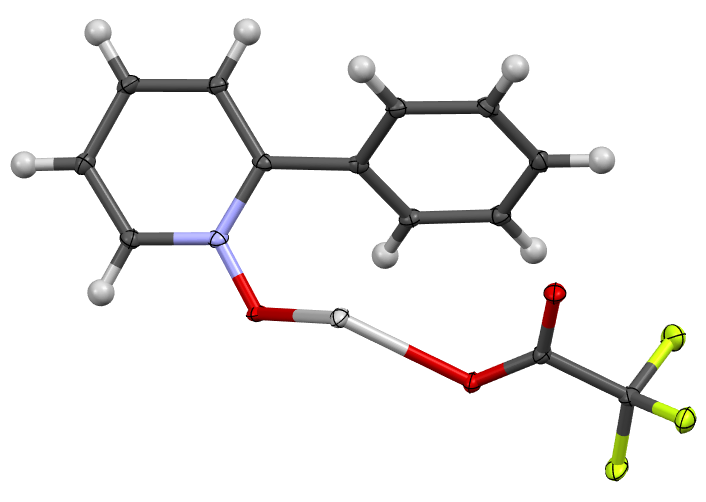


**Figure S96**. The asymmetric unit **(15Ag)H_2_O** with the thermal displacement parameter at 50% probability level. Colour Key: green = fluorine, ash white = silver, blue = nitrogen, dark grey = carbon, white = hydrogen.

**Crystal data for (15Ag)H_2_O**: CCDC-2420952, C_13_H_9_AgF_3_NO_3_, M = 392.08 gmol^‑1^, colourless blocks, 0.14 × 0.11 × 0.06 mm, triclinic, space group *P-1*, a = 5.90670(10) Å, b = 10.7291(2) Å, c = 11.5264(2) Å, α = 64.272(2) °, β = 80.304(2) °, γ = 74.082(2) °, V = 631.79(2) Å^3^, Z = 2, D_calc_ = 2.061 gcm^‑3^, F(000) = 384, µ = 13.281 mm^‑1^, T = 120(1) K, θ_max_ = 66.737°, 6792 total reflections, 2198 with I_o_ > 2σ(I_o_), R_int_ = 0.0294, 2239 data, 190 parameters, 0 restraints, GooF = 1.077, R = 0.0212 and wR = 0.0510 [I_o_ > 2σ(I_o_)], R = 0.0217 and wR = 0.0513 (all reflections), 0.504 <d∆ρ < -0.705 eÅ^‑3^.

**40. Complex (15Ag)Tol**


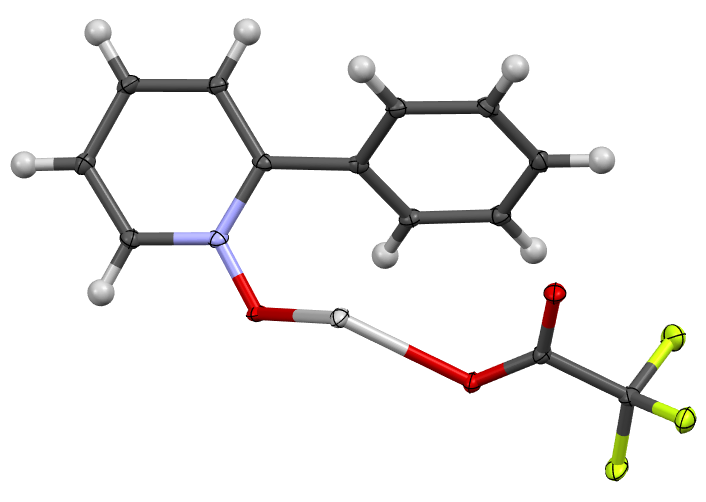


**Figure S97**. The asymmetric unit **(15Ag)Tol** with the thermal displacement parameter at 50% probability level. Colour Key: green = fluorine, ash white = silver, blue = nitrogen, dark grey = carbon, white = hydrogen.

**Crystal data for (15Ag)Tol**: CCDC-2420954, C_13_H_9_AgF_3_NO_3_, M = 392.08 gmol^‑1^, colourless needles, 0.19 × 0.03 × 0.02 mm, triclinic, space group *P-1*, a = 5.9111(2) Å, b = 10.7408(4) Å, c = 11.5202(3) Å, α = 64.293(3)°, β = 80.309(3)°, γ = 74.064(3)°, V = 632.67(4) Å^3^, Z = 2, D_calc_ = 2.058 gcm^‑3^, F(000) = 384, µ = 13.262 mm^‑1^, T = 120(1) K, θ_max_ = 66.748°, 7361 total reflections, 2038 with I_o_ > 2σ(I_o_), R_int_ = 0.0793, 2236 data, 190 parameters, 0 restraints, GooF = 1.023, R = 0.0383 and wR = 0.0906 [I_o_ > 2σ(I_o_)], R = 0.0421 and wR = 0.0923 (all reflections), 0.982 <d∆ρ <-0.959 eÅ^‑3^.

**41. Complex (16Ag)EtOAc**


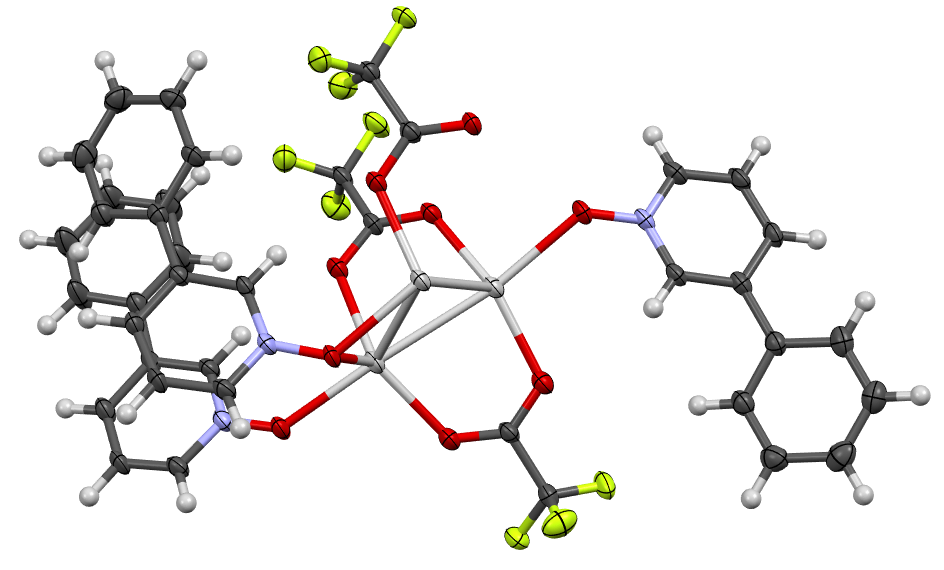


**Figure S98**. The asymmetric unit **(16Ag)EtOAc** with the thermal displacement parameter at 50% probability level. Colour Key: green = fluorine, ash white = silver, blue = nitrogen, dark grey = carbon, white = hydrogen.

**Crystal data for (16Ag)EtOAc**: CCDC-2420986, C_78_H_54_Ag_6_F_18_N_6_O_18_, M = 2352.49 gmol^‑1^, colourless needle, 0.23 × 0.05 × 0.05 mm, triclinic, space group *P-1*, a = 11.4980(4) Å, b = 12.0228(4) Å, c = 15.9645(5) Å, α = 82.337(3) °, β = 84.239(3) °, γ = 62.243(4) °, V = 1933.67(13) Å^3^, Z = 1, D_calc_ = 2.020 gcm^‑3^, F(000) = 1152 µ = 13.018 mm^‑1^, T = 150(1) K, θ_max_ = 66.749 °, 26397 total reflections, 6021 with I_o_ > 2σ(I_o_), R_int_ = 0.0755, 6864 data, 556 parameters, 0 restraints, GooF = 1.042, R = 0.0650 and wR = 0.1699 [I_o_ > 2σ(I_o_)], R = 0.0691 and wR = 0.1752 (all reflections), 2.704 <d∆ρ < -1.620 eÅ^‑3^.

**42. Complex (16Ag)Tol**


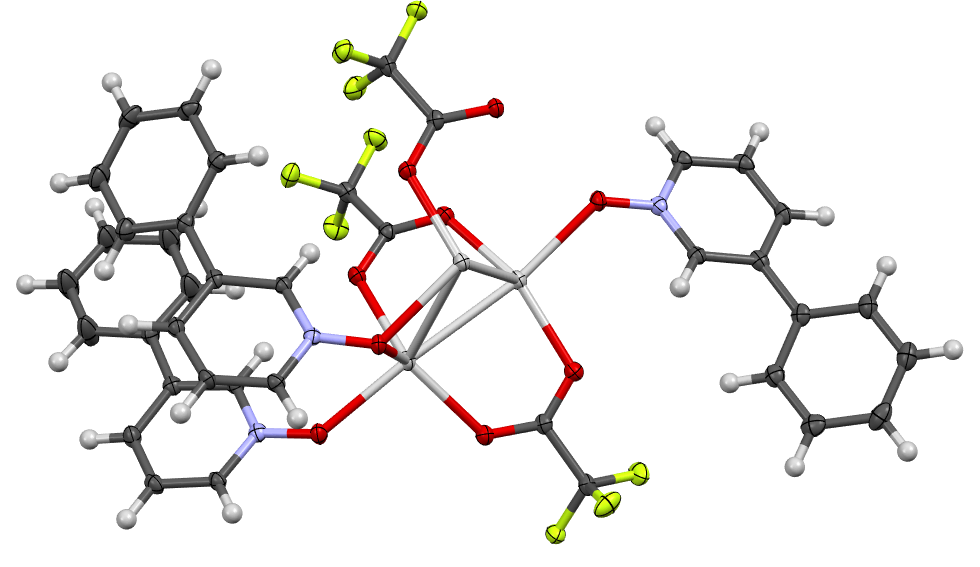


**Figure S99**. The asymmetric unit **(16Ag)Tol** with the thermal displacement parameter at 50% probability level. Colour Key: green = fluorine, ash white = silver, blue = nitrogen, dark grey = carbon, white = hydrogen.

**Crystal data for (16Ag)Tol**: CCDC-2420994, C_78_H_54_Ag_6_F_18_N_6_O_18_, M = 2352.49 gmol^‑1^, colourless needles, 0.46 × 0.07 × 0.03 mm, triclinic, space group *P-1*, a = 11.4813(3) Å, b = 11.9628(3) Å, c = 15.9748(4) Å, α = 82.093(2)°, β = 84.277(2) °, γ = 62.106(2)°, V = 1919.19(9) Å^3^, Z = 1, D_calc_ = 2.035gcm^‑3^, F(000) = 1152 µ =13.116 mm^‑1^, T = 120(1) K, θ_max_ = 66.750 °, 24845 total reflections, 6288 with I_o_ > 2σ(I_o_), R_int_ = 0.0490, 6805 data, 550 parameters, 0 restraints, GooF = 1.077, R = 0.0417 and wR = 0.1166 [I_o_ > 2σ(I_o_)], R = 0.0438 and wR = 0.1184 (all reflections), 1.717 <d∆ρ < -2.010 eÅ^‑3^.

**43. Complex (17Ag)Ben**


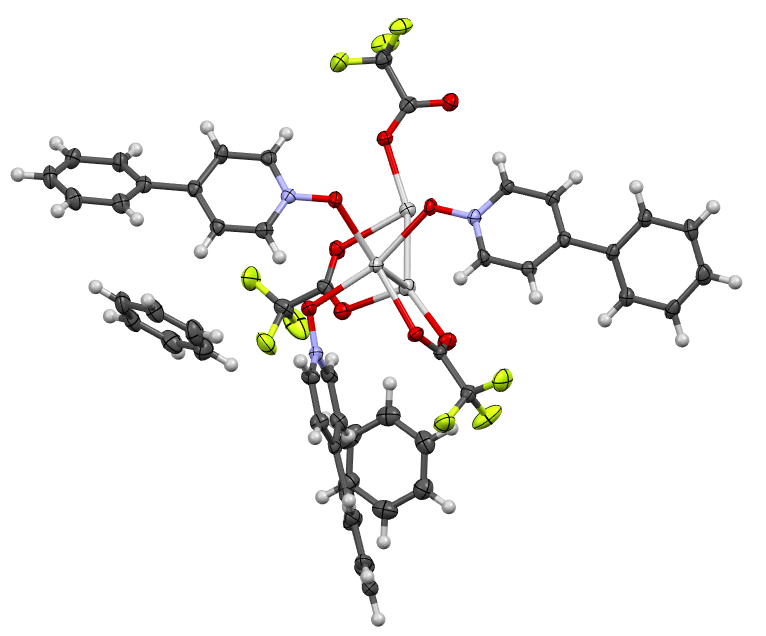


**Figure S100**. The asymmetric unit **(17Ag)Ben** with the thermal displacement parameter at 50% probability level. Colour Key: green = fluorine, ash white = silver, blue = nitrogen, dark grey = carbon, white = hydrogen.

**Crystal data for (17Ag)Ben**: CCDC-2420979, C_51_H_39_Ag_3_F_9_N_3_O_9_, M = 1332.46 gmol^‑1^, colourless needle, 0.23 × 0.03 × 0.02 mm, triclinic, space group *P-1*, a = 9.7032(6) Å, b = 17.1583(9) Å, c = 17.4229(6) Å, α = 108.439(4)°, β = 102.172(4)°, γ = 105.217(5)°, V = 2514.8(2) Å^3^, Z = 2, D_calc_ = 1.760 gcm^‑3^, F(000) = 1320, µ = 10.096 mm^‑1^, T = 120(1) K, θ_max_ = 66.748°, 25323 total reflections, 6355 with I_o_ > 2σ(I_o_), R_int_ = 0.0858, 8805 data, 676 parameters, 0 restraints, GooF = 0.976, R = 0.0685 and wR = 0.1716 [I_o_ > 2σ(I_o_)], R = 0.0897 and wR = 0.1867 (all reflections), 1.372 <d∆ρ < -2.173 eÅ^‑3^.

**44. Complex (17Ag)Ben_a**


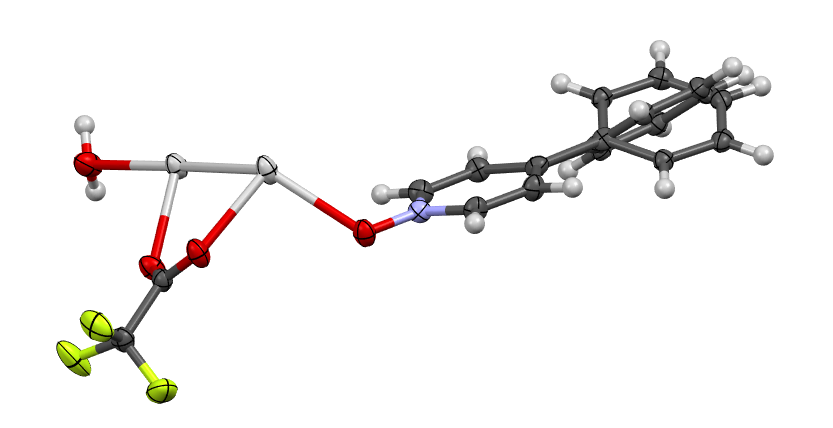


**Figure S101**. The asymmetric unit **(17Ag)Ben_a** with the thermal displacement parameter at 50% probability level. Colour Key: green = fluorine, ash white = silver, blue = nitrogen, dark grey = carbon, white = hydrogen.

**Crystal data for (17Ag)Ben_a**: CCDC-2420981, C_26_H_20_Ag_2_F_6_N_2_O_7_, M = 802.18 gmol^‑1^, colourless plates, 0.18 × 0.14 × 0.05 mm, monoclinic, space group *C2/c*, a = 32.3189(6) Å, b = 11.0359(2) Å, c = 7.6713(2) Å, α = 90°, β = 94.073(2)°, γ = 90°, V = 2729.20(10) Å^3^, Z = 4, D_calc_ = 1.952 gcm^‑3^, F(000) = 1576, µ = 12.342 mm^‑1^, T = 150(1) K, θ_max_ = 66.720°, 19733 total reflections, 2238 with I_o_ > 2σ(I_o_), R_int_ = 0.0582, 2423 data, 231 parameters, 0 restraints, GooF = 1.042, R = 0.0313 and wR = 0.0848 [I_o_ > 2σ(I_o_)], R = 0.0331 and wR = 0.0864 (all reflections), 0.929 <d∆ρ < -0.554 eÅ^‑3^.

**45. Complex (17Ag)EtOAc**

**Figure S102**. The asymmetric unit **(17Ag)EtOAc** with the thermal displacement parameter at 50% probability level. Colour Key: green = fluorine, ash white = silver, blue = nitrogen, dark grey = carbon, white = hydrogen.

**Crystal data for (17Ag)EtOAc**: CCDC-2420980, C_26_H_18_Ag_2_F_6_N_2_O_6_, M = 784.16 gmol^‑1^, colourless blocks, 0.11 × 0.1 × 0.08 mm, tetragonal, space group *P 4_1_2_1_2*, a = 10.92710(10) Å, b = 10.92710(10) Å, c = 44.5084(4) Å, α = 90°, β = 90°, γ = 90°, V = 5314.37(11) Å^3^, Z = 8, D_calc_ = 1.960 gcm^‑3^, F(000) = 3072, µ = 12.631 mm^‑1^, T = 120(1) K, θ_max_ = 66.728 °, 45604 total reflections, 4610 with I_o_ > 2σ(I_o_), R_int_ = 0.0495, 4697 data, 379 parameters, 0 restraints, GooF = 1.051, R = 0.0185 and wR = 0.0424 [I_o_ > 2σ(I_o_)], R = 0.0191 and wR = 0.0426 (all reflections), 0.508 <d∆ρ < -0.336 eÅ^‑3^.

**46. Complex (17Ag)EtOAc_a**

**Figure S103**. The asymmetric unit **(17Ag)EtOAc_a** with the thermal displacement parameter at 50% probability level. Colour Key: green = fluorine, ash white = silver, blue = nitrogen, dark grey = carbon, white = hydrogen.

**Crystal data for (17Ag)EtOAc_a**: CCDC-2420997, C_26_H_22_Ag_2_F_6_N_2_O_8_, M = 820.19 gmol^‑1^, colourless blocks, 0.185 × 0.093 × 0.027 mm, triclinic, space group *P-1*, a = 5.9104(2) Å, b = 7.4971(2) Å, c = 15.8891(6) Å, α = 89.236(3)°, β = 87.448(3)°, γ = 73.247(3)°, V = 673.51(4) Å^3^, Z = 1, D_calc_ = 2.022 gcm^‑3^, F(000) = 404, µ = 12.549 mm^‑1^, T = 150(1) K, θ_max_ = 66.729 °, 8666 total reflections, 2328 with I_o_ > 2σ(I_o_), R_int_ = 0.0384, 2378 data, 236 parameters, 0 restraints, GooF = 0.762, R = 0.0274 and wR = 0.0810 [I_o_ > 2σ(I_o_)], R = 0.0278 and wR = 0.0818 (all reflections), 1.045 <d∆ρ < -0.576 eÅ^‑3^

**47. Complex (17Ag)Tol**

**Figure S104**. The asymmetric unit **(17Ag)Tol** with the thermal displacement parameter at 50% probability level. Colour Key: green = fluorine, ash white = silver, blue = nitrogen, dark grey = carbon, white = hydrogen.

**Crystal data for (17Ag)Tol**: CCDC-2420974, C_26_H_22_Ag_2_F_6_N_2_O_8_, M = 820.19 gmol^‑1^, colourless blocks, 0.225 × 0.036 × 0.025 mm, triclinic, space group *P-1*, a = 5.9026(2) Å, b = 7.4869(4) Å, c = 15.8666(8) Å, α = 89.210(4)°, β = 87.455(4)°, γ = 73.217(4)°, V = 670.65(6) Å^3^, Z = 1, D_calc_ = 2.031 gcm^‑3^, F(000) = 404, µ = 12.602 mm^‑1^, T = 120(1) K, θ_max_ = 66.724 °, 8091 total reflections, 2243 with I_o_ > 2σ(I_o_), R_int_ = 0.0828, 2361 data, 235 parameters, 0 restraints, GooF = 1.047, R = 0.0467 and wR = 0.1288 [I_o_ > 2σ(I_o_)], R = 0.0482 and wR = 0.1307 (all reflections), 0.958 <d∆ρ < -1.877 eÅ^‑3^.

**48. Complex (17Ag)Tol_a**

**Figure S105**. The asymmetric unit **(17Ag)Tol_a** with the thermal displacement parameter at 50% probability level. Colour Key: green = fluorine, ash white = silver, blue = nitrogen, dark grey = carbon, white = hydrogen.

**Crystal data for (17Ag)Tol_a**: CCDC-2420987, C_26_H_20_Ag_2_F_6_N_2_O_7_, M = 802.18 gmol^‑1^, colourless plates, 0.18 × 0.08 × 0.03 mm, monoclinic, space group *C2/c*, a = 32.3082(5)Å, b = 11.03250(10) Å, c = 7.63850(10) Å, α = 90°, β = 94.140(2)°, γ = 90°, V = 2715.56(6) Å^3^, Z = 4, D_calc_ = 1.962 gcm^‑3^, F(000) = 1576, µ = 12.404 mm^‑1^, T = 120(1) K, θ_max_ = 66.727 °, 20058 total reflections, 2207 with I_o_ > 2σ(I_o_), R_int_ = 0.0562, 2407 data, 237 parameters, 0 restraints, GooF = 1.046, R = 0.0253 and wR = 0.0621 [I_o_ > 2σ(I_o_)], R = 0.0278 and wR = 0.0634 (all reflections), 0.381 <d∆ρ < -0.622 eÅ^‑3^.

**49. Complex (20Ag)Ben**

**Figure S106**. The asymmetric unit **(20Ag)Ben** with the thermal displacement parameter at 50% probability level. Colour Key: green = fluorine, ash white = silver, blue = nitrogen, dark grey = carbon, white = hydrogen.

**Crystal data for (20Ag)Ben**: CCDC-2420998, C_19_H_11_Ag_2_F_6_N_4_O_6_, M = 721.06 gmol^‑1^, colourless block, 0.16 × 0.08 × 0.03 mm, monoclinic, space group *P2_1_/n*, a = 13.0378(3) Å, b = 10.8128(3) Å, c = 16.6037(5) Å, α = 90°, β = 100.464(3)°, γ = 90°, V = 2301.78(11) Å^3^, Z = 4, D_calc_ = 2.081 gcm^‑3^, F(000) = 1396, µ = 14.531 mm^‑1^, T = 120(1) K, θ_max_ = 76.723 °, 9125 total reflections, 8399 with I_o_ > 2σ(I_o_), R_int_ = twinned, 9125 data, 335 parameters, 0 restraints, GooF = 1.063, R = 0.0536 and wR = 0.1555 [I_o_ > 2σ(I_o_)], R = 0.0566 and wR = 0.1603 (all reflections), 0.939 <d∆ρ < -1.201 eÅ^‑3^.

**50. Complex (20Ag)Tol**

**Figure S107.** The asymmetric unit **(20Ag)Tol** with the thermal displacement parameter at 50% probability level. Colour Key: green = fluorine, ash white = silver, blue = nitrogen, dark grey = carbon, white = hydrogen.

**Crystal data for (20Ag)Tol**: CCDC-2420990, C_40_H_20_Ag_5_F_15_N_10_O_15_, M = 1705.01 gmol^‑1^, colourless plate, 0.13 × 0.06 × 0.04 mm, monoclinic, space group *C2/n*, a = 38.8613(13) Å, b = 10.8321(3) Å, c = 24.7346(6) Å, α = 90°, β = 96.127(2)°, γ = 90°, V = 10352.5(5) Å^3^, Z = 4, D_calc_ = 2.188 gcm^‑3^, F(000) = 6560, µ = 16.102 mm^‑1^, T = 150(1) K, θ_max_ = 66.746°, 44903 total reflections, 6547 with I_o_ > 2σ(I_o_), R_int_ = 0.1111, 9143 data, 820 parameters, 18 restraints, GooF = 1.014, R = 0.0419 and wR = 0.0881 [I_o_ > 2σ(I_o_)], R = 0.0693 and wR = 0.0997 (all reflections), 0.640 <d∆ρ < -0.903 eÅ^‑3^.

**51. Complex (21Ag)H_2_O**

**Figure S108**. The asymmetric unit **(21Ag)H_2_O** with the thermal displacement parameter at 50% probability level. Colour Key: green = fluorine, ash white = silver, blue = nitrogen, dark grey = carbon, white = hydrogen.

**Crystal data for (21Ag)H_2_O**: CCDC-2420995, C_9_H_6_Ag_2_F_6_N_2_O_8_, M = 599.90 gmol^‑1^, yellow block, 0.13 × 0.09 × 0.05 mm, monoclinic, space group *P2_1_/c*, a = 14.15500(10) Å, b = 7.35410(10) Å, c = 14.2521(2) Å, α = 90°, β = 96.0500(10)°, γ = 90°, V = 1475.34(3) Å^3^, Z = 4, D_calc_ = 2.701 gcm^‑3^, F(000) = 1144, µ = 22.498 mm^‑1^, T = 120(1) K, θ_max_ = 66.717 °, 15879 total reflections, 2554 with I_o_ > 2σ(I_o_), R_int_ = 0.0452, 2612 data, 252 parameters, 2 restraints, GooF = 1.074, R = 0.0214 and wR = 0.0527 [I_o_ > 2σ(I_o_)], R = 0.0220 and wR = 0.0531 (all reflections), 0.725 <d∆ρ <-0.564 eÅ^‑3^.

**52. Complex (21Ag)Tol**

**Figure S109**. The asymmetric unit **(21Ag)Tol** with the thermal displacement parameter at 50% probability level. Colour Key: green = fluorine, ash white = silver, blue = nitrogen, dark grey = carbon, white = hydrogen.

**Crystal data for (21Ag)Tol**: CCDC-2420978, C_18_H_12_Ag_4_F_12_N_4_O_16_, M = 1199.80 gmol^‑1^, yellow block, 0.11 × 0.09 × 0.09 mm, triclinic, space group *P-1*, a = 7.55130(9) Å, b = 14.26573(14) Å, c = 14.29049(15) Å, α = 82.3251(8)°, β = 81.5022(9)°, γ = 88.5312(9)°, V = 1508.87(3) Å^3^, Z = 2, D_calc_ = 2.641 gcm^‑3^, F(000) = 1144, µ = 21.998 mm^‑1^, T = 120(1) K, θ_max_ = 77.477°, 20979 total reflections, 5903 with I_o_ > 2σ(I_o_), R_int_ = 0.0572, 6319 data, 489 parameters, 2 restraints, GooF = 1.010, R = 0.0503 and wR = 0.1438 [I_o_ > 2σ(I_o_)], R = 0.0518 and wR = 0.1460 (all reflections), 3.397<d∆ρ < -2.717 eÅ^‑3^.

**53. Complex (21Ag)Tol_a**

**Figure S110**. The asymmetric unit **(21Ag)Tol_a** with the thermal displacement parameter at 50% probability level. Colour Key: green = fluorine, ash white = silver, blue = nitrogen, dark grey = carbon, white = hydrogen.

**Crystal data for (21Ag)Tol_a**: CCDC-2420999, C_9_H_6_Ag_2_F_6_N_2_O_8_, M = 599.90 gmol^‑1^, yellow plate, 0.22 × 0.13 × 0.03 mm, monoclinic, space group *P2_1_/c*, a = 14.1587(2) Å, b = 7.35190(10) Å, c = 14.2542(2) Å, α = 90°, β = 96.049(2)°, γ = 90°, V = 1475.34(3) Å^3^, Z = 4, D_calc_ = 2.701 gcm^‑3^, F(000) = 1144, µ = 22.495 mm^‑1^, T = 120(1) K, θ_max_ = 66.737 °, 10149 total reflections, 2489 with I_o_ > 2σ(I_o_), R_int_ = 0.0513, 2612 data, 245 parameters, 0 restraints, GooF = 1.037, R = 0.0359 and wR = 0.0983 [I_o_ > 2σ(I_o_)], R = 0.0370 and wR = 0.0996 (all reflections), 1.630 <d∆ρ <-1.022 eÅ^‑3^.

**54. Complex (24Ag)Ben**

**Figure S111**. The asymmetric unit **(24Ag)Ben** with the thermal displacement parameter at 50% probability level. Colour Key: green = fluorine, ash white = silver, blue = nitrogen, dark grey = carbon, white = hydrogen.

**Crystal data for (24Ag)Ben**: CCDC-2420992, C_18_H_18_Ag_2_F_6_N_6_O_10_, M = 808.12 gmol^‑1^, yellow plates, 0.17 × 0.1 × 0.04 mm, monoclinic, space group *P2_1_/n*, a = 14.5092(2) Å, b = 7.62810(10) Å, c = 22.6895(4) Å, α = 90°, β = 90.1080(10) °, γ = 90°, V = 2511.22(7) Å^3^, Z = 4, D_calc_ = 13.555 gcm^‑3^, F(000) = 1584, µ = 13.555 mm^‑1^, T = 120(1) K, θ_max_ = 66.750°, 18333 total reflections, 4099 with I_o_ > 2σ(I_o_), R_int_ = 0.0467, 4446 data, 401 parameters, 0 restraints, GooF = 1.056, R = 0.0368 and wR = 0.0981 [I_o_ > 2σ(I_o_)], R = 0.0395 and wR = 0.0997 (all reflections), 1.200 <d∆ρ <-1.343 eÅ^‑3^.

**55. Complex (24Ag)EtOAc**

**Figure S112**. The asymmetric unit **(24Ag)EtOAc** with the thermal displacement parameter at 50% probability level. Colour Key: green = fluorine, ash white = silver, blue = nitrogen, dark grey = carbon, white = hydrogen.

**Crystal data for (24Ag)EtOAc**: CCDC-2420977, C_18_H_18_Ag_2_F_6_N_6_O_10_, M = 808.12 gmol^‑1^, yellow plate, 0.1 × 0.09 × 0.05 mm, monoclinic, space group *P2_1_/n*, a = 14.5017(2) Å, b = 7.62890(10) Å, c = 22.6908(2) Å, α = 90°, β = 90.1620(10) °, γ = 90°, V = 2510.32(5) Å^3^, Z = 4, D_calc_ = 2.138 gcm^‑3^, F(000) = 1584, µ = 13.559 mm^‑1^, T = 120(1) K, θ_max_ = 66.739 °, 19707 total reflections, 4091 with I_o_ > 2σ(I_o_), R_int_ = 0.0354, 4378 data, 401 parameters, 0 restraints, GooF = 1.041, R = 0.0283 and wR = 0.0677 [I_o_ > 2σ(I_o_)], R = 0.0307 and wR = 0.0689 (all reflections), 0.668 <d∆ρ <-0.737eÅ^‑3^.

**56. Complex (24Ag)Tol_a**

**Figure S113**. The asymmetric unit **(24Ag)Tol_a** with the thermal displacement parameter at 50% probability level. Colour Key: green = fluorine, ash white = silver, blue = nitrogen, dark grey = carbon, white = hydrogen.

**Crystal data for (24Ag)Tol_a**: CCDC-2420985, C_18_H_18_Ag_2_F_6_N_6_O_10_, M = 808.12 gmol^‑1^, yellow block, 0.08 × 0.07 × 0.05 mm, monoclinic, space group *P2_1_/n*, a = 14.4983(2) Å, b = 7.63300(10) Å, c = 22.6719(2) Å, α = 90 °, β = 90.0900(10) °, γ = 90 °, V = 2508.99(5) Å^3^, Z = 4, D_calc_ = 2.139 gcm^‑3^, F(000) = 1584, µ = 13.567 mm^‑1^, T = 120(1) K, θ_max_ = 66.749°, 26892 total reflections, 4310 with I_o_ > 2σ(I_o_), R_int_ = 0.0371, 4437 data, 401 parameters, 0 restraints, GooF = 1.278, R = 0.0467 and wR = 0.1155 [I_o_ > 2σ(I_o_)], R = 0.0478 and wR = 0.1159 (all reflections), 0.756 <d∆ρ < -0.949 eÅ^‑3^.

**57. Complex (25Ag)Ben**

**Figure S114**. The asymmetric unit **(25Ag)Ben** with the thermal displacement parameter at 50% probability level. Colour Key: green = fluorine, ash white = silver, blue = nitrogen, dark grey = carbon, white = hydrogen.

**Crystal data for (25Ag)Ben**: CCDC-2420988, C_12_H_12_AgF_3_N_3_O_5_, M = 443.12 gmol^‑1^, yellow block, 0.16 × 0.07 × 0.06 mm, triclinic, space group *P-1*, a = 6.7059(2) Å, b = 10.1466(3)Å, c = 12.2726(3) Å, α = 66.839(3)°, β = 82.450(2)°, γ = 78.640(2)°, V = 751.36(4) Å^3^, Z = 2, D_calc_ = 1.959 gcm^‑3^, F(000) = 438, µ = 11.398 mm^‑1^, T = 120(1) K, θ_max_ = 66.747°, 9110 total reflections, 2576 with I_o_ > 2σ(I_o_), R_int_ = 0.0306, 2653 data, 234 parameters, 0 restraints, GooF = 1.045, R = 0.0230 and wR = 0.0571 [I_o_ > 2σ(I_o_)], R = 0.0237 and wR = 0.0574 (all reflections), 0.734 <d∆ρ < -0.595 eÅ^‑3^.

**58. Complex (25Ag)Acet**

**Figure S115**. The asymmetric unit **(25Ag)Acet** with the thermal displacement parameter at 50% probability level. Colour Key: green = fluorine, ash white = silver, blue = nitrogen, dark grey = carbon, white = hydrogen.

**Crystal data for (25Ag)Acet**: CCDC-2420983, C_36_H_36_Ag_4_F_12_N_12_O_20_, M = 1616.25 gmol^‑1^, yellow plate, 0.15 × 0.11 × 0.03 mm, triclinic, space group *P-1*, a = 13.5513(3) Å, b = 14.9282(2)Å, c = 14.9494(2) Å, α = 64.104(2)°, β = 78.015(2)°, γ = 85.513(2)°, V = 2660.98(9) Å^3^, Z = 2, D_calc_ = 2.017 gcm^‑3^, F(000) = 1584, µ = 12.792 mm^‑1^, T = 120(1) K, θ_max_ = 66.746°, 37899 total reflections, 8381 with I_o_ > 2σ(I_o_), R_int_ = 0.0347, 9419 data, 765 parameters, 0 restraints, GooF = 1.042, R = 0.0277 and wR = 0.0705 [I_o_ > 2σ(I_o_)], R = 0.0320 and wR = 0.0727 (all reflections), 0.613 <d∆ρ < -0.742eÅ^‑3^.

**59. Complex (25Ag)EtOAc**

**Figure S116**. The asymmetric unit **(25Ag)EtOAc** with the thermal displacement parameter at 50% probability level. Colour Key: green = fluorine, ash white = silver, blue = nitrogen, dark grey = carbon, white = hydrogen.

**Crystal data for (25Ag)EtOAc**: CCDC-2420984, C_36_H_36_Ag_4_F_12_N_12_O_20_, M = 1616.25 gmol^‑1^, yellow plate, 0.09 × 0.04 × 0.02 mm, triclinic, space group *P-1*, a = 13.5603(4) Å, b = 14.9258(5)Å, c = 14.9437(6) Å, α = 64.175(4)°, β = 77.968(3)°, γ = 85.540(3)°, V = 2662.41(18) Å^3^, Z = 2, D_calc_ = 2.016 gcm^‑3^, F(000) = 1584, µ = 12.785 mm^‑1^, T = 120(1) K, θ_max_ = 66.748°, 28776 total reflections, 7479 with I_o_ > 2σ(I_o_), R_int_ = 0.0397, 9388 data, 765 parameters, 0 restraints, GooF = 0.993, R = 0.0322 and wR = 0.0743 [I_o_ > 2σ(I_o_)], R = 0.0441 and wR = 0.0785 (all reflections), 0.574 <d∆ρ < -0.689eÅ^‑3^.

**60. Complex (25Ag)EtOH**

**Figure S117**. The asymmetric unit **(25Ag)EtOH** with the thermal displacement parameter at 50% probability level. Colour Key: green = fluorine, ash white = silver, blue = nitrogen, dark grey = carbon, white = hydrogen.

**Crystal data for (25Ag)EtOH**: CCDC-2420975, C_11_H_15_AgF_3_N_3_O_6_, M = 450.13 gmol^‑1^, yellow plate, 0.16 × 0.1 × 0.04 mm, triclinic, space group *P-1*, a = 5.6353(2) Å, b = 12.4982(4) Å, c = 12.5192(3) Å, α = 110.743(3) °, β = 99.916(2) °, γ = 102.664(3) °, V = 773.77(4) Å^3^, Z = 2, D_calc_ = 1.932 gcm^‑3^, F(000) = 448, µ = 11.124 mm^‑1^, T = 120(1) K, θ_max_ = 66.749°, 8050 total reflections, 2533 with I_o_ > 2σ(I_o_), R_int_ = 0.0488, 2733 data, 221 parameters, 0 restraints, GooF = 1.075, R = 0.0505 and wR = 0.1352 [I_o_ > 2σ(I_o_)], R = 0.0526 and wR = 0.1372 (all reflections), 2.362 <d∆ρ < -1.631 eÅ^‑3^.

**61. Complex (25Ag)Tol_a**

**Figure S118**. The asymmetric unit **(25Ag)Tol_a** with the thermal displacement parameter at 50% probability level. Colour Key: green = fluorine, ash white = silver, blue = nitrogen, dark grey = carbon, white = hydrogen.

**Crystal data for (25Ag)Tol_a**: CCDC-2420976, C_9_H_9_AgF_3_N_3_O_5_, M = 404.06 gmol^‑1^, yellow plates (plates stacked to form block), 0.14 × 0.07 × 0.03 mm, triclinic, space group *P-1*, a = 5.8022(3) Å, b = 8.8667(4) Å, c = 12.5534(4) Å, α = 88.014(3)°, β = 78.932(3)°, γ = 81.664(4)°, V = 627.10(5) Å^3^, Z = 2, D_calc_ = 2.140 gcm^‑3^, F(000) = 396, µ = 13.570 mm^‑1^, T = 120(1) K, θ_max_ = 66.747°, 7997 total reflections, 2192 with I_o_ > 2σ(I_o_), R_int_ = 0.0305, 2220 data, 192 parameters, 0 restraints, GooF = 1.052, R = 0.0262 and wR = 0.0700 [I_o_ > 2σ(I_o_)], R = 0.0265 and wR = 0.0703 (all reflections), 0.922 <d∆ρ < -1.004 eÅ^‑3^.

**62. Ligand 25_H_2_O**

**Figure S119**. The asymmetric unit **25_H_2_O** with the thermal displacement parameter at 50% probability level. Colour Key: green = fluorine, ash white = silver, blue = nitrogen, dark grey = carbon, white = hydrogen.

**Crystal data for 25_H_2_O**: CCDC-2420996, C_7_H_11_N_3_O_4_, M = 201.19 gmol^‑1^, yellow needle, 0.26 × 0.06 × 0.03 mm, monoclinic, space group *P2_1_*, a = 8.7124(6) Å, b = 4.6053(2) Å, c = 11.8742(7) Å, α = 90°, β = 106.306(7) °, γ = 90°, V = 457.27(5) Å^3^, Z = 2, D_calc_ = 1.461 gcm^‑3^, F(000) = 212, µ = 1.037 mm^‑1^, T = 120(1) K, θ_max_ = 66.717°, 5143 total reflections, 1155 with I_o_ > 2σ(I_o_), R_int_ = 0.0857, 1265 data, 132 parameters, 1 restraints, GooF = 1.040, R = 0.0550 and wR = 0.1353 [I_o_ > 2σ(I_o_)], R = 0.0589 and wR = 0.1388 (all reflections), 0.330 <d∆ρ < -0.291 eÅ^‑3^.

**63. Complex (26Ag)Acet**

**Figure S120**. The asymmetric unit **(26Ag)Acet** with the thermal displacement parameter at 50% probability level. Colour Key: green = fluorine, ash white = silver, blue = nitrogen, dark grey = carbon, white = hydrogen. The asymmetric unit contains crystallographically two independent molecules (of which one is disordered, and one is not shown for viewing clarity) of the same shown above.

**Crystal data for (26Ag)Acet**: CCDC-2420991, C_45_H_29_Ag_4_F_12_N_4_O_12_, M = 1477.20 gmol^‑1^, colourless plates, 0.31 × 0.06 × 0.03 mm, monoclinic, space group *P2_1_/c*, a = 27.5789(4) Å, b = 7.53070(10) Å, c = 26.4137(4) Å, α = 90°, β = 117.690(2) °, γ = 90°, V = 4857.55(14) Å^3^, Z = 4, D_calc_ = 2.020 gcm^‑3^, F(000) = 2876, µ = 13.767 mm^‑1^, T = 120(1) K, θ_max_ = 66.748 °, 30422 total reflections, 7458 with I_o_ > 2σ(I_o_), R_int_ = 0.0364, 8535 data, 665 parameters, 30 restraints, GooF = 1.049, R = 0.0518 and wR = 0.1275 [I_o_ > 2σ(I_o_)], R = 0.0598 and wR = 0.1323 (all reflections), 1.739 <d∆ρ < -1.388 eÅ^‑3^.

**64. Complex (26Ag)Ben**

**Figure S121**. The asymmetric unit **(26Ag)Ben** with the thermal displacement parameter at 50% probability level. Colour Key: green = fluorine, ash white = silver, blue = nitrogen, dark grey = carbon, white = hydrogen. The asymmetric unit contains crystallographically two independent molecules (only one is shown for viewing clarity) of the same shown above.

**Crystal data for (26Ag)Ben**: CCDC-2420989, C_22_H_16_Ag_2_F_6_N_2_O_7_, M = 750.11 gmol^‑1^, colourless needles, 0.06× 0.06 × 0.04 mm, triclinic, space group *P-1,* a = 7.3131(2) Å, b = 13.09650(10) Å, c = 24.8335(2) Å, α = 90.3500(10)°, β = 92.515(2) °, γ = 97.755(2)°, V = 2354.26(7) Å^3^, Z = 4, D_calc_ = 2.116 gcm^‑3^, F(000) = 1464, µ = 14.247 mm^‑1^, T = 120(1) K, θ_max_ = 66.744°, 33925 total reflections, 7625 with I_o_ > 2σ(I_o_), R_int_ = 0.0412, 8344 data, 709 parameters, 2 restraints, GooF = 1.073, R = 0.0705 and wR = 0.2349 [I_o_ > 2σ(I_o_)], R = 0.0735 and wR = 0.2373 (all reflections), 5.256 <d∆ρ < -1.643 eÅ^‑3^.

**65. Complex (26Ag)EtOAc**

**Figure S122**. The asymmetric unit **(26Ag)EtOAc** with the thermal displacement parameter at 50% probability level. Colour Key: green = fluorine, ash white = silver, blue = nitrogen, dark grey = carbon, white = hydrogen.

**Crystal data for (26Ag)EtOAc**: CCDC-2420993, C_44_H_32_Ag_4_F_12_N_4_O_14_, M = 1500.21 gmol^‑1^, colourless plate, 0.13× 0.06 × 0.02 mm, triclinic, space group *P-1,* a = 7.2193(3) Å, b = 13.1832(4) Å, c = 13.6420(5) Å, α = 113.370(3)°, β = 97.620(4) °, γ = 92.990(3)°, V = 1173.46(8) Å^3^, Z = 1, D_calc_ = 2.123 gcm^‑3^, F(000) = 732, µ = 14.291 mm^‑1^, T = 120(1) K, θ_max_ = 66.741°, 13739 total reflections, 3592 with I_o_ > 2σ(I_o_), R_int_ = 0.0498, 4173 data, 355 parameters, 1 restraints, GooF = 1.006, R = 0.0343 and wR = 0.0865 [I_o_ > 2σ(I_o_)], R = 0.0403 and wR = 0.0895 (all reflections), 0.643 <d∆ρ < -1.331 eÅ^‑3^.

**66. Complex (26Ag)Tol**

**Figure S123**. The asymmetric unit **(26Ag)Tol** with the thermal displacement parameter at 50% probability level. Colour Key: green = fluorine, ash white = silver, blue = nitrogen, dark grey = carbon, white = hydrogen. The asymmetric unit contains crystallographically two independent molecules (only one is shown for viewing clarity) of the same shown above.

**Crystal data for (26Ag)Tol**: CCDC-2420982, C_88_H_64_Ag_8_F_24_N_8_O_28_, M = 3000.43 gmol^‑1^, colourless block, 0.1× 0.1 × 0.09 mm, triclinic, space group *P-1,* a = 7.31356(16) Å, b = 13.10310(18) Å, c = 24.8199(2) Å, α = 90.3518(9)°, β = 92.6220(12)°, γ = 97.7676(15)°, V = 2354.02(7) Å^3^, Z = 1, D_calc_ = 2.117 gcm^‑3^, F(000) = 1464, µ = 14.248 mm^‑1^, T = 120(1) K, θ_max_ = 66.742°, 33874 total reflections, 7512 with I_o_ > 2σ(I_o_), R_int_ = 0.0456, 8345 data, 713 parameters, 5 restraints, GooF = 1.077, R = 0.0692 and wR = 0.2358 [I_o_ > 2σ(I_o_)], R = 0.0729 and wR = 0.2392 (all reflections), 5.608 <d∆ρ < -1.592 eÅ^‑3^.

**Figure S124**. The partial pacing structure of **(20Ag)Tol** to demonstrate the -CN group participating in the silver(I) coordination. The trifluoroacetate groups are shown in capped stick models for viewing clarity.

**Figure S125**. The partial pacing structure of **(17Ag)H_2_O** to demonstrate the AgTFA forming both paddlewheel and non-paddlewheel structures. Selected 4-*tert*-butylpyridine N-oxide ligands are shown in capped stick models for viewing clarity.

**6. Hirshfeld surface analysis**

Hirshfeld surface analysis for **(9Ag)Tol**, **(14Ag)Ben**, **(20Ag)Tol**, **(21Ag)Tol_a**, **(21Ag)H_2_O** and **(26)Acet** were unable to be calculated due to the disorder and polymeric nature of the complexes.

**Table S14**. Hirshfeld surface analysis of silver(I) complexes **(1Ag)Ben – (3Ag)H_2_O_2**

| NCI type (%) | **(1Ag)Ben** | **(1Ag)Tol** | **(1Ag)H_2_O** | **(3Ag)EtOH** | **(3Ag)H_2_O** | **(3Ag)H_2_O_2** |
| --- | --- | --- | --- | --- | --- | --- |
| Ag∙∙∙Ag | 0 | 0 | 0 | 0.2 | 1.6 | 0.2 |
| Ag∙∙∙F | 1 | 0.9 | 0.9 | 0 | 0 | 0 |
| Ag∙∙∙O | 5 | 5 | 5 | 4.3 | 4.3 | 4.3 |
| Ag∙∙∙N | 0 | 0 | 0 | 0 | 0 | 0 |
| Ag∙∙∙C | 0 | 0 | 0 | 1.4 | 1.3 | 1.3 |
| Ag∙∙∙H | 0.9 | 0.9 | 0.9 | 3.6 | 3.2 | 3.6 |
| F∙∙∙F | 0.5 | 0.5 | 0.5 | 4.3 | 1.3 | 4.2 |
| F∙∙∙O | 3.9 | 3.8 | 3.8 | 1.6 | 1.5 | 1.7 |
| F∙∙∙N | 1.2 | 1.5 | 1.5 | 0.7 | 0.6 | 0.7 |
| F∙∙∙C | 6.3 | 6.3 | 6.3 | 6.8 | 6.5 | 7 |
| F∙∙∙H | 35.2 | 35.8 | 35.4 | 33 | 40.2 | 33 |
| O∙∙∙O | 1.2 | 1.2 | 1.2 | 1.3 | 0 | 1.3 |
| O∙∙∙N | 0.5 | 0.5 | 0.5 | 2.3 | 1.9 | 2.3 |
| O∙∙∙C | 0.9 | 0.9 | 0.9 | 4.2 | 3.7 | 4.2 |
| O∙∙∙H | 23.1 | 23.1 | 23.0 | 15.9 | 19.6 | 15.9 |
| N∙∙∙N | 0 | 0 | 0 | 0 | 0 | 0 |
| N∙∙∙C | 0.2 | 0.2 | 0.2 | 0.1 | 0.1 | 0.1 |
| N∙∙∙H | 0.3 | 0.3 | 0.3 | 1.1 | 0.8 | 1.1 |
| C∙∙∙C | 0.2 | 0.2 | 0.2 | 1.8 | 1.8 | 1.8 |
| C∙∙∙H | 3.9 | 3.8 | 3.8 | 3.7 | 2.6 | 3.5 |
| H∙∙∙H | 15.4 | 15.5 | 15.5 | 13.6 | 9 | 13.8 |

**Table S15.** Hirshfeld surface analysis of silver(I) complexes **(4Ag)Acet – (4Ag)EtOH_2**

| NCI type (%) | **(4Ag)Acet** | **(4Ag)Acet_2** | **(4Ag)Acet_3** | **(4Ag)Acet_4** | **(4Ag)EtOH_2** |
| --- | --- | --- | --- | --- | --- |
| Ag∙∙∙Ag | 0.9 | 0 | 0.9 | 0 | 0 |
| Ag∙∙∙F | 0 | 3.2 | 0 | 3.2 | 3.1 |
| Ag∙∙∙O | 3 | 5.8 | 3.1 | 5.4 | 5.6 |
| Ag∙∙∙N | 0.3 | 0.1 | 0.3 | 0.1 | 0.1 |
| Ag∙∙∙C | 0 | 0 | 0 | 0 | 0 |
| Ag∙∙∙H | 0.6 | 0.6 | 0.6 | 0.6 | 0.6 |
| F∙∙∙F | 5.3 | 7 | 5.2 | 6.8 | 6.8 |
| F∙∙∙O | 0.6 | 7 | 0.6 | 7.7 | 7.3 |
| F∙∙∙N | 0.1 | 1.4 | 0.1 | 1.4 | 1.4 |
| F∙∙∙C | 3.3 | 3.5 | 3.4 | 3.6 | 3.7 |
| F∙∙∙H | 37 | 40.4 | 37.2 | 40 | 40.1 |
| O∙∙∙O | 1.5 | 1.3 | 1.5 | 1.1 | 1.2 |
| O∙∙∙N | 1.1 | 0.4 | 1.1 | 0.4 | 0.4 |
| O∙∙∙C | 2.5 | 1.3 | 2.5 | 1.3 | 1.3 |
| O∙∙∙H | 17.0 | 16.1 | 17.0 | 16.7 | 16.6 |
| N∙∙∙N | 0 | 0 | 0 | 0 | 0 |
| N∙∙∙C | 0.6 | 0 | 0.7 | 0 | 0 |
| N∙∙∙H | 1.5 | 0 | 1.4 | 0 | 0 |
| C∙∙∙C | 2.2 | 0.3 | 2.2 | 0.3 | 0.3 |
| C∙∙∙H | 6.1 | 2.7 | 6.0 | 2.6 | 2.6 |
| H∙∙∙H | 16.4 | 8.9 | 16.3 | 8.8 | 8.8 |

**Table S16.** Hirshfeld surface analysis of silver(I) complexes **(4Ag)EtOH_3 – (4Ag)H_2_O_2**

| NCI type (%) | **(4Ag)EtOH_3** | **(4Ag)EtOH_4** | **(4Ag)EtOH_7** | **(4Ag)H2O** | **(4Ag)H2O_2** |
| --- | --- | --- | --- | --- | --- |
| Ag∙∙∙Ag | 0 | 0 | 0.9 | 0 | 0 |
| Ag∙∙∙F | 3.1 | 3.0 | 0 | 0.7 | 3.6 |
| Ag∙∙∙O | 5.8 | 5.8 | 3.1 | 0 | 6.4 |
| Ag∙∙∙N | 0.1 | 0.1 | 0.3 | 0 | 0.1 |
| Ag∙∙∙C | 0 | 0 | 0.4 | 2.2 | 0 |
| Ag∙∙∙H | 0.6 | 0.6 | 0.6 | 1.1 | 0.6 |
| F∙∙∙F | 6.8 | 6.9 | 5.1 | 3.1 | 6.9 |
| F∙∙∙O | 7.1 | 7.4 | 0.6 | 3.2 | 6.8 |
| F∙∙∙N | 1.4 | 1.3 | 0.1 | 0 | 1.4 |
| F∙∙∙C | 3.6 | 3.6 | 3.3 | 0.7 | 3.5 |
| F∙∙∙H | 40.5 | 40.3 | 37.4 | 43.3 | 40.2 |
| O∙∙∙O | 1.3 | 1.3 | 1.5 | 0.3 | 1.1 |
| O∙∙∙N | 0.4 | 0.4 | 1.1 | 0.8 | 0.4 |
| O-C | 1.3 | 1.3 | 2.3 | 2.5 | 1.4 |
| O∙∙∙H | 16.2 | 16.2 | 17.0 | 22.3 | 15.7 |
| N∙∙∙N | 0 | 0 | 0 | 0 | 0 |
| N∙∙∙C | 0 | 0 | 0.7 | 0.3 | 0 |
| N∙∙∙H | 0 | 0 | 1.4 | 1.1 | 0 |
| C∙∙∙C | 0.3 | 0.3 | 2.1 | 0.1 | 0.3 |
| C∙∙∙H | 2.6 | 2.4 | 6.0 | 4.4 | 2.6 |
| H∙∙∙H | 9.0 | 8.9 | 16.2 | 14.2 | 8.9 |

**Table S17.** Hirshfeld surface analysis of silver(I) complexes **(4Ag)H_2_O_3 – (8Ag)H_2_O**

| NCI type (%) | **(4Ag)H2O_3** | **(4Ag)H2O_4** | **(4Ag)H2O_5** | **(4Ag)H2O_a** | **(8Ag)H2O** |
| --- | --- | --- | --- | --- | --- |
| Ag∙∙∙Ag | 0 | 0 | 1.1 | 1.1 | 0.1 |
| Ag∙∙∙F | 3.6 | 3.5 | 0.5 | 0.5 | 1.4 |
| Ag∙∙∙O | 6.5 | 6.6 | 3.7 | 3.7 | 2.9 |
| Ag∙∙∙N | 0.1 | 0.1 | 0.1 | 0.1 | 0 |
| Ag∙∙∙C | 0 | 0 | 1.7 | 1.7 | 0.1 |
| Ag∙∙∙H | 0.6 | 0.6 | 1 | 1 | 4.1 |
| F∙∙∙F | 7 | 6.9 | 4.9 | 4.9 | 2.3 |
| F∙∙∙O | 6.7 | 6.5 | 2.9 | 2.8 | 3.4 |
| F∙∙∙N | 1.4 | 1.4 | 0 | 0 | 0.7 |
| F∙∙∙C | 3.6 | 3.6 | 0.6 | 0.5 | 2.2 |
| F∙∙∙H | 40.5 | 40.5 | 35 | 35.1 | 33.3 |
| O∙∙∙O | 1.2 | 1.4 | 1.4 | 1.4 | 0.8 |
| O∙∙∙N | 0.4 | 0.4 | 0.8 | 0.7 | 0 |
| O∙∙∙C | 1.3 | 1.3 | 2.6 | 2.7 | 0.9 |
| O∙∙∙H | 15.3 | 15.2 | 20.1 | 20.0 | 16.2 |
| N∙∙∙N | 0 | 0 | 0 | 0 | 0 |
| N∙∙∙C | 0 | 0 | 0.9 | 0.9 | 0.6 |
| N∙∙∙H | 0 | 0 | 1.4 | 1.4 | 1.4 |
| C∙∙∙C | 0.3 | 0.3 | 1.6 | 1.6 | 1.1 |
| C∙∙∙H | 2.6 | 2.7 | 5.7 | 5.8 | 6.9 |
| H∙∙∙H | 8.8 | 9 | 14.2 | 14.2 | 21.7 |

**Table S18.** Hirshfeld surface analysis of silver(I) complexes **(10Ag)EtOH – (11Ag)EtOAc**

| NCI type (%) | **(10Ag)EtOH** | **(10Ag)H2O** | **(10Ag)H2O_a** | **(11Ag)Tol** | **(11Ag)EtOAc** |
| --- | --- | --- | --- | --- | --- |
| Ag∙∙∙Ag | 0 | 0 | 0 | 0.1 | 0 |
| Ag∙∙∙F | 0.5 | 0.5 | 0.5 | 0 | 2 |
| Ag∙∙∙O | 3.5 | 3.6 | 3.5 | 4.5 | 4.2 |
| Ag∙∙∙N | 0.1 | 0.1 | 0.1 | 0.4 | 0 |
| Ag∙∙∙C | 1.5 | 1.4 | 1.4 | 0.7 | 0 |
| Ag∙∙∙H | 1.5 | 1.5 | 1.5 | 1.8 | 1.3 |
| F∙∙∙F | 2.8 | 2.8 | 2.8 | 2.7 | 2.3 |
| F∙∙∙O | 3.1 | 3.1 | 3.2 | 1.6 | 4.1 |
| F∙∙∙N | 0.9 | 0.6 | 0.9 | 0 | 0.9 |
| F∙∙∙C | 2.7 | 1.9 | 2.8 | 2.3 | 5 |
| F∙∙∙H | 34 | 34.2 | 34.4 | 32 | 41.2 |
| O∙∙∙O | 1 | 0.9 | 1 | 0.5 | 0.9 |
| O∙∙∙N | 0.8 | 0.9 | 0.8 | 1.1 | 0.4 |
| O∙∙∙C | 2.2 | 2.8 | 2.2 | 3 | 0.8 |
| O∙∙∙H | 14.7 | 14.3 | 14.3 | 16.5 | 17.5 |
| N∙∙∙N | 0 | 0 | 0 | 0 | 0 |
| N∙∙∙C | 0.1 | 0.3 | 0.1 | 0.3 | 0 |
| N∙∙∙H | 1.2 | 1.2 | 1.2 | 0.6 | 0.3 |
| C∙∙∙C | 0.5 | 0.5 | 0.5 | 0.1 | 0.3 |
| C∙∙∙H | 6.5 | 7 | 6.5 | 2.7 | 0.2 |
| H∙∙∙H | 22.4 | 22.5 | 22.2 | 29 | 18.3 |

**Table S19.** Hirshfeld surface analysis of silver(I) complexes **(11Ag)EtOH – (13Ag)Acet**

| NCI type (%) | **(11Ag)EtOH** | **(11Ag)H2O** | **(13Ag)Tol** | **(13Ag)EtOAc_a** | **(13Ag)Acet** |
| --- | --- | --- | --- | --- | --- |
| Ag∙∙∙Ag | 0 | 0.4 | 1.2 | 1.2 | 1.2 |
| Ag∙∙∙F | 2 | 0 | 0.1 | 0.1 | 0.1 |
| Ag∙∙∙O | 4.1 | 3.6 | 3.5 | 3.5 | 3.5 |
| Ag∙∙∙N | 0 | 0 | 0 | 0 | 0 |
| Ag∙∙∙C | 0 | 0 | 1.3 | 1.3 | 1.6 |
| Ag∙∙∙H | 1.3 | 2.1 | 1.1 | 1.2 | 1.2 |
| F∙∙∙F | 2.3 | 2.6 | 6.2 | 6.3 | 6.3 |
| F∙∙∙O | 4.2 | 0 | 3.0 | 3.0 | 3.0 |
| F∙∙∙N | 0.9 | 0 | 0.9 | 0.9 | 0.9 |
| F∙∙∙C | 5 | 1 | 3.0 | 3.0 | 3.1 |
| F∙∙∙H | 41.2 | 29.4 | 26.1 | 26.0 | 25.9 |
| O∙∙∙O | 0.8 | 0.9 | 2.5 | 2.5 | 2.5 |
| O∙∙∙N | 0.4 | 0.7 | 0.5 | 0.5 | 0.5 |
| O-C | 0.8 | 1 | 2.4 | 2.3 | 2.4 |
| O∙∙∙H | 17.7 | 12.7 | 25.7 | 25.5 | 25.7 |
| N∙∙∙N | 0 | 0 | 0 | 0 | 0 |
| N∙∙∙C | 0 | 0.1 | 0.5 | 0.5 | 0.6 |
| N∙∙∙H | 0.2 | 1.1 | 1.1 | 1.1 | 1.1 |
| C∙∙∙C | 0.3 | 0.6 | 2.1 | 2.1 | 2.1 |
| C∙∙∙H | 0.2 | 5 | 5.8 | 5.8 | 5.8 |
| H∙∙∙H | 18.3 | 38.8 | 12.8 | 13.0 | 12.9 |

**Table S20.** Hirshfeld surface analysis of silver(I) complexes **(14Ag)EtOAc – (15Ag)EtOAc_a**

| NCI type (%) | **(14Ag)EtOAc_a** | **(15Ag)Tol** | **(15Ag)EtOAc** | **(15Ag)EtOAc_a** |
| --- | --- | --- | --- | --- |
| Ag∙∙∙Ag | 0 | 1.2 | 1.2 | 1.2 |
| Ag∙∙∙F | 1.8 | 1 | 0.9 | 1 |
| Ag∙∙∙O | 3,5 | 6.2 | 6.2 | 6.2 |
| Ag∙∙∙N | 0 | 0 | 0 | 0 |
| Ag∙∙∙C | 0 | 1.7 | 1.7 | 1.7 |
| Ag∙∙∙H | 0.4 | 1.4 | 1.4 | 1.4 |
| F∙∙∙F | 6.7 | 0.1 | 0.1 | 0.1 |
| F∙∙∙O | 6.7 | 3.6 | 3.6 | 3.6 |
| F∙∙∙N | 1.5 | 0 | 0 | 0 |
| F∙∙∙C | 6.4 | 2.2 | 2.2 | 2.2 |
| F∙∙∙H | 37.7 | 28.5 | 28.5 | 28.6 |
| O∙∙∙O | 0.9 | 1.1 | 1.1 | 1.1 |
| O∙∙∙N | 0.2 | 1.4 | 1.4 | 1.4 |
| O∙∙∙C | 0.7 | 2 | 2 | 2 |
| O∙∙∙H | 20.8 | 11.3 | 11.3 | 11.4 |
| N∙∙∙N | 0 | 0 | 0 | 0 |
| N∙∙∙C | 0 | 0.2 | 0.2 | 0.2 |
| N∙∙∙H | 0.1 | 1 | 1 | 1 |
| C∙∙∙C | 0.2 | 0.4 | 0.4 | 0.4 |
| C∙∙∙H | 1.5 | 16.2 | 16.3 | 16.2 |
| H∙∙∙H | 10.9 | 20.5 | 20.4 | 20.4 |

**Table S21.** Hirshfeld surface analysis of silver(I) complexes **(15Ag)Acet – (16Ag)EtOAc**

| NCI type (%) | **(15Ag)Acet** | **(15Ag)EtOH** | **(15Ag)H2O** | **(16Ag)Tol** | **(16Ag)EtOAc** |
| --- | --- | --- | --- | --- | --- |
| Ag∙∙∙Ag | 1.2 | 1.2 | 1.2 | 1 | 1 |
| Ag∙∙∙F | 1 | 0.9 | 0.1 | 1.2 | 1.2 |
| Ag∙∙∙O | 6.2 | 6.2 | 3.5 | 3.4 | 3.5 |
| Ag∙∙∙N | 0 | 0 | 0 | 0 | 0 |
| Ag∙∙∙C | 1.7 | 1.7 | 1.3 | 0 | 0 |
| Ag∙∙∙H | 1.4 | 1.4 | 1.1 | 1 | 1 |
| F∙∙∙F | 0.1 | 0.1 | 6.2 | 2.1 | 2.1 |
| F∙∙∙O | 3.6 | 3.6 | 3.0 | 2.6 | 2.6 |
| F∙∙∙N | 0 | 0 | 0.9 | 0.1 | 0.1 |
| F∙∙∙C | 2.2 | 2.2 | 3.0 | 2.1 | 2.1 |
| F∙∙∙H | 28.5 | 28.5 | 26.1 | 26.1 | 26.2 |
| O∙∙∙O | 1.1 | 1.1 | 2.5 | 1 | 1 |
| O∙∙∙N | 1.4 | 1.4 | 0.5 | 0.2 | 0.2 |
| O∙∙∙C | 2 | 2 | 2.4 | 1.7 | 1.6 |
| O∙∙∙H | 11.3 | 11.3 | 25.7 | 15.8 | 15.8 |
| N∙∙∙N | 0 | 0 | 0 | 0.4 | 0.4 |
| N∙∙∙C | 0.2 | 0.2 | 0.5 | 1.0 | 1.0 |
| N∙∙∙H | 1 | 1 | 1.1 | 0.6 | 0.6 |
| C∙∙∙C | 0.4 | 0.4 | 2.1 | 6.1 | 6.1 |
| C∙∙∙H | 16.2 | 16.2 | 5.8 | 11 | 11.1 |
| H∙∙∙H | 20.4 | 20.5 | 12.8 | 22.5 | 22.6 |

**Table S22.** Hirshfeld surface analysis of silver(I) complexes **(17Ag)Ben – (17Ag)EtOAc**

| NCI type (%) | **(17Ag)Ben** | **(17Ag)Ben_a** | **(17Ag)Tol** | **(17Ag)Tol_a** | **(17Ag)EtOAc** |
| --- | --- | --- | --- | --- | --- |
| Ag∙∙∙Ag | 0.6 | 0 | 0 | 0 | 0.2 |
| Ag∙∙∙F | 0.2 | 0.1 | 0.6 | 0.1 | 0 |
| Ag∙∙∙O | 6.4 | 3.1 | 1.8 | 3.1 | 3.6 |
| Ag∙∙∙N | 0 | 0 | 0 | 0 | 0 |
| Ag∙∙∙C | 0.1 | 0.4 | 0 | 0.4 | 0.9 |
| Ag∙∙∙H | 1.6 | 0.3 | 0 | 0.3 | 3.2 |
| F∙∙∙F | 1.9 | 2.9 | 0 | 2.9 | 3.8 |
| F∙∙∙O | 1.3 | 2 | 2.5 | 2 | 0.1 |
| F∙∙∙N | 0 | 0 | 1.4 | 0 | 0.4 |
| F∙∙∙C | 2.3 | 2.2 | 5.3 | 2.2 | 6.3 |
| F∙∙∙H | 24 | 27.1 | 25.3 | 27.1 | 26.1 |
| O∙∙∙O | 1.2 | 0.7 | 0.5 | 0.7 | 1 |
| O∙∙∙N | 1 | 0.1 | 0 | 0.1 | 0.6 |
| O∙∙∙C | 1.9 | 0.6 | 0 | 0.6 | 2.2 |
| O∙∙∙H | 10.4 | 17.1 | 15.7 | 17.1 | 16.8 |
| N∙∙∙N | 0 | 0 | 0 | 0 | 0 |
| N∙∙∙C | 0.7 | 0.4 | 0 | 0.4 | 1.1 |
| N∙∙∙H | 0.7 | 2 | 0.1 | 2 | 0.4 |
| C∙∙∙C | 1.6 | 7.7 | 5.8 | 7.7 | 3.5 |
| C∙∙∙H | 22.3 | 11.7 | 8.2 | 11.7 | 11 |
| H∙∙∙H | 21.7 | 21.6 | 32.6 | 21.6 | 18.9 |

**Table S23.** Hirshfeld surface analysis of silver(I) complexes **(17Ag)EtOAc_a – (21Ag)Tol**

| NCI type (%) | **(17Ag)EtOAc_a** | **(20Ag)Ben*** | **(21Ag)Tol** |
| --- | --- | --- | --- |
| Ag∙∙∙Ag | 0 | 0.2 | 0 |
| Ag∙∙∙F | 0.6 | 0 | 2 |
| Ag∙∙∙O | 1.8 | 4.2 | 3.6 |
| Ag∙∙∙N | 0 | 5.3 | 0 |
| Ag∙∙∙C | 0 | 0 | 0 |
| Ag∙∙∙H | 0 | 0.2 | 0.1 |
| F∙∙∙F | 0 | 5.9 | 6.6 |
| F∙∙∙O | 2.4 | 0.7 | 20.1 |
| F∙∙∙N | 1.3 | 4.9 | 2.3 |
| F∙∙∙C | 5.3 | 9.2 | 5.3 |
| F∙∙∙H | 25.7 | 11.8 | 23 |
| O∙∙∙O | 0.8 | 1.6 | 2.3 |
| O∙∙∙N | 0 | 2 | 1.5 |
| O∙∙∙C | 0 | 3.3 | 2.4 |
| O∙∙∙H | 15.5 | 18.5 | 25.5 |
| N∙∙∙N | 0 | 1.2 | 0 |
| N∙∙∙C | 0 | 1.4 | 0.1 |
| N∙∙∙H | 0.3 | 9.1 | 0.1 |
| C∙∙∙C | 5.8 | 3.4 | 0 |
| C∙∙∙H | 8.1 | 6.6 | 0.5 |
| H∙∙∙H | 32.6 | 7.8 | 4.9 |

*benzene solvent not included in the HSA analysis. Including benzene in HSA, the program crashes

**Table S24.** Hirshfeld surface analysis of silver(I) complexes **(24Ag)Ben – (25Ag)Tol_a**

| NCI type (%) | **(24Ag)Ben** | **(24Ag)Tol_a** | **(24Ag)EtOAc** | **(25Ag)Ben** | **(25Ag)Tol_a** |
| --- | --- | --- | --- | --- | --- |
| Ag∙∙∙Ag | 0 | 0 | 0 | 0 | 0.1 |
| Ag∙∙∙F | 0 | 0 | 0 | 0 | 0 |
| Ag∙∙∙O | 5.3 | 5.3 | 5.3 | 2.1 | 4 |
| Ag∙∙∙N | 0.6 | 0.5 | 0.5 | 0.8 | 1.1 |
| Ag∙∙∙C | 0.2 | 0.2 | 0.2 | 0.1 | 0.2 |
| Ag∙∙∙H | 1.9 | 1.9 | 1.9 | 4.1 | 2.8 |
| F∙∙∙F | 3.9 | 3.8 | 3.8 | 2.5 | 3.5 |
| F∙∙∙O | 6.5 | 6.4 | 6.5 | 3.3 | 6.1 |
| F∙∙∙N | 0.9 | 1 | 0.9 | 0.9 | 0.4 |
| F∙∙∙C | 1.2 | 1.2 | 1.2 | 3.6 | 3.2 |
| F∙∙∙H | 21.8 | 21.9 | 22 | 19.3 | 21.7 |
| O∙∙∙O | 3.7 | 3.7 | 3.6 | 1.2 | 1.6 |
| O∙∙∙N | 2.5 | 2.5 | 2.4 | 2.4 | 1.8 |
| O∙∙∙C | 7.6 | 7.6 | 7.5 | 5.3 | 5.9 |
| O∙∙∙H | 24.8 | 24.9 | 25.1 | 23.6 | 24.9 |
| N∙∙∙N | 0 | 0 | 0 | 0 | 0 |
| N∙∙∙C | 0.2 | 0.2 | 0.2 | 1.9 | 1.7 |
| N∙∙∙H | 0.3 | 0.3 | 0.3 | 0.5 | 3 |
| C∙∙∙C | 0.9 | 0.9 | 0.9 | 1.3 | 1.3 |
| C∙∙∙H | 3.5 | 3.3 | 3.3 | 8.1 | 3.6 |
| H∙∙∙H | 14.4 | 14.5 | 14.3 | 18.8 | 13 |

**Table S25.** Hirshfeld surface analysis of silver(I) complexes **(25Ag)EtOAc – (26Ag)EtOAc**

| NCI type (%) | **(25Ag)EtOAc** | **(25Ag)Acet** | **(25Ag)EtOH** | **(26Ag)Ben** | **(26Ag)Tol** | **(26Ag)EtOAc** |
| --- | --- | --- | --- | --- | --- | --- |
| Ag∙∙∙Ag | 1.7 | 1.1 | 0.1 | 0.2 | 0.2 | 0.2 |
| Ag∙∙∙F | 0 | 0 | 0 | 0.1 | 0.1 | 0.1 |
| Ag∙∙∙O | 4 | 4.2 | 3.2 | 4.8 | 4.8 | 3.7 |
| Ag∙∙∙N | 0.4 | 0.4 | 1.1 | 0 | 0 | 0 |
| Ag∙∙∙C | 0.2 | 0.2 | 0.4 | 0.6 | 0.6 | 0.7 |
| Ag∙∙∙H | 0.6 | 0.6 | 2.6 | 0.3 | 0.4 | 0.8 |
| F∙∙∙F | 3.2 | 3.1 | 5.1 | 4.9 | 4.9 | 4.2 |
| F∙∙∙O | 6.5 | 7.8 | 0.8 | 2 | 1.9 | 2.1 |
| F∙∙∙N | 0.1 | 0.1 | 0 | 0 | 0 | 0 |
| F∙∙∙C | 0 | 0.5 | 1.8 | 2.2 | 2.3 | 2.1 |
| F∙∙∙H | 27.9 | 24.1 | 16.9 | 30.2 | 30.2 | 28.4 |
| O∙∙∙O | 2.3 | 4.7 | 0.9 | 1.5 | 1.4 | 1.2 |
| O∙∙∙N | 1 | 0.9 | 1.8 | 0 | 0 | 0 |
| O∙∙∙C | 3.2 | 3.2 | 6.2 | 0.8 | 0.8 | 0.9 |
| O∙∙∙H | 26.1 | 28.8 | 26.8 | 16.7 | 16.7 | 16.9 |
| N∙∙∙N | 0.2 | 0.2 | 0 | 0 | 0 | 0 |
| N∙∙∙C | 1.0 | 1.1 | 1.3 | 1.1 | 1.1 | 1.5 |
| N∙∙∙H | 2.3 | 2.3 | 3.1 | 1.7 | 1.6 | 1.5 |
| C∙∙∙C | 1.0 | 1.1 | 1.2 | 9.2 | 9.2 | 9.6 |
| C∙∙∙H | 3.6 | 3.2 | 4 | 5.7 | 5.7 | 6.7 |
| H∙∙∙H | 14.7 | 12.3 | 22.7 | 17.9 | 17.9 | 19.6 |

**7. Powder X-ray diffraction**

Profile and weighted profile R-factors (R_p_ and R_wp_) with Goodness of fit (GOF = χ) are the agreement factors for the Pawley fits.

$\chi=\frac{\sum w_{i\left( y_{io}-y_{ic} \right)^{½}}}{N-P}= \left[ \frac{R_{wp}}{R_{exp}} \right]$

**Figure S126.** Pawley refinement plot of **(4Ag)EtOAc** obtained using 1:1 PyNO:AgTFA equivalent ratio. Experimental PXRD pattern is shown in black, while the refined profile is shown in red. The green- and fuchsia-coloured bars on top correspond to characteristic Bragg peak positions of the assigned phases, which correspond to single crystal structures of **(4Ag)H_2_O_5** (CCDC number: 2420941) and **(4Ag)Acet** (CCDC number: 2420936), respectively. The primary phase of these two is **(4Ag)Acet**. The difference plot of the experimental vs. refined profiles is shown below in blue. Unit cell parameters are given in Table S25.

**Table S26.** Pawley fit results of **(4Ag)EtOAc.**

| Parameters | SCXRD | PXRD | SCXRD | PXRD |
| --- | --- | --- | --- | --- |
|  | **(4Ag)H2O_5** | | **(4Ag)Acet** | |
| Temp (K) | 120 | 293 | 160 | 293 |
| Crystal system | triclinic | triclinic | triclinic | triclinic |
| Space group | *P-1* | *P-1* | *P-1* | *P-1* |
| *a* /Å | 11.1686(8) | 11.323(8) | 11.1408(4) | 10.786(3) |
| *b* /Å | 11.2239(6) | 11.10(1) | 14.2693(10) | 14.267(6) |
| *c* /Å | 14.3218(6) | 14.143(9) | 15.8845(9) | 15.88(1) |
| *α* /° | 108.773(4) | 108.11(1) | 115.148(7) | 113.77(1) |
| *β* /° | 97.281(5) | 97.45(2) | 91.377(4) | 92.14(2) |
| *γ* /° | 112.532(7) | 112.92(2) | 95.088(4) | 94.49(2) |
| *V*/Å^3^ | 1504.50 | 1491.28 | 2271.48 | 2223.39 |
| *R*_p_ | 0.0445 | | | |
| *R*_wp_ | 0.0568 | | | |
| *GOF* | 1.638 | | | |

**Figure S127.** Pawley refinement plot of **(4Ag)Acet** (CCDC number: 2420936) obtained using 1:1 PyNO:AgTFA equivalent ratio. Experimental PXRD pattern is shown in black, while the refined profile is shown in red. The green- and fuchsia-coloured bars on top correspond to characteristic Bragg peak positions of the assigned phases, which correspond to single crystal structures of **(4Ag)H_2_O_5** (CCDC number: 2420941) and **(4Ag)Acet** (CCDC number: 2420936), respectively. The **(4Ag)Acet** sample contains roughly 1:1 ratio of **(4Ag)H_2_O_5** and **(4Ag)Acet**. No other phases are observed. The difference plot of the experimental vs. refined profiles is shown below in blue. Unit cell parameters are given in Table S26.

**Table S27**. Pawley fit results of **(4Ag)Acet**

| Parameters | SCXRD | PXRD | SCXRD | PXRD |
| --- | --- | --- | --- | --- |
|  | **(4Ag)H2O_5** | | **(4Ag)Acet** | |
| Temp (K) | 120 | 293 | 160 | 293 |
| Crystal system | triclinic | triclinic | triclinic | triclinic |
| Space group | *P-1* | *P-1* | *P-1* | *P-1* |
| *a* /Å | 11.1686(8) | 11.45(1) | 11.1408(4) | 11.099(2) |
| *b* /Å | 11.2239(6) | 11.13(1) | 14.2693(10) | 14.224(2) |
| *c* /Å | 14.3218(6) | 14.34(1) | 15.8845(9) | 15.870(2) |
| *α* /° | 108.773(4) | 108.65(1) | 115.148(7) | 113.677(2) |
| *β* /° | 97.281(5) | 97.28(1) | 91.377(4) | 91.917(1) |
| *γ* /° | 112.532(7) | 112.43(2) | 95.088(4) | 94.894(1) |
| *V*/Å^3^ | 1504.5 | 1533.87 | 2271.48 | 2279.77 |
| *R*_p_ | 0.0541 | | | |
| *R*_wp_ | 0.0636 | | | |
| *GOF* | 1.941 | | | |

**Figure 128.** Pawley refinement plot of **(4Ag)Acet_2** obtained using 1:2 PyNO:AgTFA equivalent ratio. Experimental PXRD pattern is shown in black and refined profile in red, whereas green, fuchsia and blue colored bars on top correspond to characteristic Bragg peak positions of the assigned phases corresponding to single crystal structures of **(4Ag)H_2_O_5** (CCDC number: 2420941), **(4Ag)Acet** (CCDC number: 2420936), and **(4Ag)Acet_2** (CCDC number: 2420940), respectively. The sample contains a mixture of **(4Ag)H2O_5**, **(4Ag)Acet** and **(4Ag)Acet_2** phases, of which the **(4Ag)Acet_2** seems to be the main phase with strongest peak intensities. **(4Ag)H_2_O_5** and **(4Ag)Acet** are less dominant but roughly equal portions relative to each other. No other phases observed. The difference plot of experimental vs. refined profile is shown below in blue. Unit cell parameters are given in Table S27.

**Table S28**. Pawley fit results of **(4Ag)Acet_2.**

| Parameters | SCXRD | PXRD | SCXRD | PXRD | SCXRD | PXRD |
| --- | --- | --- | --- | --- | --- | --- |
|  | **(4Ag)H_2_O_5** | | **(4Ag)Acet_2** | | **(4Ag)Acet** | |
| Temp (K) | 120 | 293 | 170 | 293 | 160 | 293 |
| Crystal system | triclinic | triclinic | triclinic | triclinic | triclinic | triclinic |
| Space group | *P-1* | *P-1* | *P-1* | *P-1* | *P-1* | *P-1* |
| *a* /Å | 11.1686(8) | 11.461(4) | 7.1272(3) | 7.183(2) | 11.1408(4) | 11.099(1) |
| *b* /Å | 11.2239(6) | 11.103(5) | 7.9649(4) | 8.025(2) | 14.2693(10) | 14.101(2) |
| *c* /Å | 14.3218(6) | 14.333(4) | 13.8340(8) | 13.776(1) | 15.8845(9) | 15.866(2) |
| *α* /° | 108.773(4) | 108.492(5) | 91.870(4) | 92.287(7) | 115.148(7) | 113.726(4) |
| *β* /° | 97.281(5) | 97.365(4) | 94.100(4) | 90.83(1) | 91.377(4) | 91.917(3) |
| *γ* /° | 112.532(7) | 112.278(5) | 104.262(4) | 105.986(6) | 95.088(4) | 94.877(3) |
| *V*/Å^3^ | 1504.5 | 1535.19 | 758.14 | 762.58 | 2271.48 | 2258.84 |
| *R*_p_ | 0.0708 | | | | | |
| *R*_wp_ | 0.0879 | | | | | |
| *GOF* | 3.056 | | | | | |

**Figure S129.** Pawley refinement plot of **(4Ag)Acet_3** (CCDC number: 2420935) obtained using 1:3 PyNO:AgTFA equivalent ratio. Experimental PXRD pattern is shown in black and refined profile in red, whereas fuchsia- and blue-coloured bars on top correspond to characteristic Bragg peak positions of the assigned phases corresponding to single crystal structures of **(4Ag)Acet** and **(4Ag)Acet_2**, accordingly. The sample contains a mixture of **(4Ag)Acet** (CCDC number: 2420936) and **(4Ag)Acet_2** (CCDC number: 2420940) of which the latter is the main phase. The difference plot of experimental vs. refined profile is shown below in blue. Unit cell parameters are given in Table S28.

**Table 29**. Pawley fit results of **(4Ag)Acet_3**.

| Parameters | SCXRD | PXRD | SCXRD | PXRD |
| --- | --- | --- | --- | --- |
|  | **(4Ag)Acet_2** | | **(4Ag)Acet** | |
| Temp (K) | 170 | 293 | 160 | 293 |
| Crystal system | triclinic | triclinic | triclinic | triclinic |
| Space group | *P-1* | *P-1* | *P-1* | *P-1* |
| *a* /Å | 7.1272(3) | 7.288(2) | 11.1408(4) | 11.131(2) |
| *b* /Å | 7.9649(4) | 7.996(3) | 14.2693(10) | 14.265(3) |
| *c* /Å | 13.8340(8) | 13.865(1) | 15.8845(9) | 15.705(4) |
| *α* /° | 91.870(4) | 91.149(6) | 115.148(7) | 115.271(3) |
| *β* /° | 94.100(4) | 91.997(4) | 91.377(4) | 92.352(3) |
| *γ* /° | 104.262(4) | 106.302(5) | 95.088(4) | 95.562(2) |
| *V*/Å^3^ | 758.14 | 774.74 | 2271.48 | 2235.05 |
| *R*_p_ | 0.0680 | | | |
| *R*_wp_ | 0.0857 | | | |
| *GOF* | 2.245 | | | |

**Figure 130.** Pawley refinement plot of **(4Ag)Acet_4** (CCDC number: 2420944) obtained using 1:4 PyNO:AgTFA equivalent ratio. Experimental PXRD pattern is shown in black and refined profile in red, whereas fuchsia- and blue-coloured bars on top correspond to characteristic Bragg peak positions of the assigned phases corresponding to single crystal structures of **(4Ag)Acet** (CCDC number: 2420936)and **(4Ag)Acet_2** (CCDC number: 2420940), respectively. This sample contains a mixture of **(4Ag)Acet** and **(4Ag)Acet_2** of which the latter is the main phase. The difference plot of experimental vs. refined profile is shown below in blue. Unit cell parameters are given in Table S29.

**Table 30**. Pawley fit results **of (4Ag)Acet_4**

| Parameters | SCXRD | PXRD | SCXRD | PXRD |
| --- | --- | --- | --- | --- |
|  | **4Ag_Acet_2** | | **4Ag_Acet** | |
| Temp (K) | 170 | 293 | 160 | 293 |
| Crystal system | triclinic | triclinic | triclinic | triclinic |
| Space group | *P-1* | *P-1* | *P-1* | *P-1* |
| *a* /Å | 7.1272(3) | 7.288(1) | 11.1408(4) | 11.132(2) |
| *b* /Å | 7.9649(4) | 7.995(2) | 14.2693(10) | 14.266(3) |
| *c* /Å | 13.8340(8) | 13.8536(8) | 15.8845(9) | 15.705(3) |
| *α* /° | 91.870(4) | 91.149(4) | 115.148(7) | 115.270(3) |
| *β* /° | 94.100(4) | 91.992(2) | 91.377(4) | 92.345(3) |
| *γ* /° | 104.262(4) | 106.298(4) | 95.088(4) | 95.586(2) |
| *V*/Å^3^ | 758.14 | 773.90 | 2271.48 | 2235.29 |
| *R*_p_ | 0.0583 | | | |
| *R*_wp_ | 0.0761 | | | |
| *GOF* | 2.061 | | | |

**8. DFT xyz coordinates files**

**8.1 xyz coordinates for monodentate silver(I) mode**

E=-1669.14440376 a.u.

Ag 0.956452000 11.976173000 4.254315000

F 5.203159000 15.068049000 3.950112000

F 3.628647000 15.731357000 5.254920000

F 5.016290000 14.222766000 5.927988000

O 3.963845000 12.748537000 3.544904000

O 2.256263000 13.539529000 4.791234000

C 4.328731000 14.666137000 4.873492000

C 3.421122000 13.529886000 4.333503000

Ag 2.890444000 11.171468000 2.325420000

F -1.647648000 8.734709000 1.818295000

F -0.999293000 7.730991000 3.618889000

F 0.017504000 7.369820000 1.756320000

O 2.496177000 12.765592000 0.599992000

O -0.163979000 10.348334000 3.549944000

O 1.486023000 9.465652000 2.285527000

N 2.061950000 13.870037000 1.094651000

C 0.738063000 14.035710000 1.324020000

H 0.130622000 13.188761000 1.038757000

C 2.923995000 14.864854000 1.402655000

H 3.959073000 14.634018000 1.200099000

C 0.255039000 15.198680000 1.877209000

H -0.810171000 15.285380000 2.048402000

C 2.475942000 16.041644000 1.957723000

H 3.203626000 16.803698000 2.204169000

C -0.576623000 8.300090000 2.489527000

C 0.364094000 9.492313000 2.802438000

C 1.125796000 16.224878000 2.207736000

H 0.762890000 17.140478000 2.655026000

**8.2 xyz coordinates for bidentate silver(I) mode**

E=-3015.09166520 a.u.

Ag 0.447267000 11.691623000 8.830201000

Ag -1.332745000 9.787606000 10.115057000

Ag -3.147450000 7.890011000 8.145836000

Ag -5.676536000 9.141354000 7.884953000

F 2.232216000 9.557136000 13.342954000

F 3.371921000 11.184718000 12.520017000

F 3.500340000 9.219747000 11.629824000

F -2.890210000 10.731659000 5.320848000

F -4.308722000 11.713937000 6.621679000

F -2.796928000 12.866607000 5.617053000

F -5.453077000 8.526035000 3.218309000

F -4.739229000 6.528950000 3.601244000

F -3.337829000 8.156006000 3.364742000

F -4.399184000 8.764183000 12.909868000

F -5.565366000 10.499904000 12.395736000

F -3.417791000 10.567215000 12.232699000

O -0.783939000 7.830392000 8.443822000

O 1.836321000 11.227257000 10.332563000

O 0.464964000 9.793926000 11.406254000

O -2.478756000 10.649355000 8.260231000

O -0.978732000 12.046103000 7.328209000

O -5.566689000 8.649300000 5.829567000

O -3.578403000 7.599419000 5.995086000

O -5.577300000 9.540035000 9.966915000

O -3.525656000 8.654032000 10.249201000

N 0.018040000 8.310702000 7.547320000

C -0.390363000 8.512614000 6.277003000

H -1.408845000 8.216753000 6.058438000

C 0.460194000 9.070066000 5.348753000

H 0.085951000 9.227894000 4.345761000

C 1.751578000 9.419808000 5.706031000

C 2.157539000 9.184211000 7.010823000

H 3.154836000 9.432245000 7.350202000

C 1.279871000 8.629145000 7.913164000

H 1.511959000 8.412430000 8.945840000

C -4.564785000 8.056246000 5.392171000

C -4.533083000 7.822824000 3.860476000

C -4.547441000 9.278688000 10.604905000

C -4.499947000 9.789850000 12.067320000

C 1.519705000 10.412942000 11.228736000

C 2.671200000 10.097693000 12.216896000

C -2.072065000 11.449183000 7.406555000

C -3.037493000 11.715405000 6.223066000

H 2.425783000 9.865533000 4.986739000

**8.3 xyz coordinates for bidentate (one silver(I) and one water)**

E=-1745.54366820 a.u.

O 4.834241000 12.190611000 2.170365000

H 5.599540000 12.327399000 1.567873000

H 4.371080000 11.408406000 1.860072000

Ag 6.158266000 11.487259000 4.205348000

F 10.003516000 15.309130000 4.324028000

F 8.147800000 15.716092000 5.331190000

F 9.609437000 14.418204000 6.248007000

O 9.093502000 12.978533000 3.539691000

O 7.294036000 13.223121000 4.883525000

C 9.054637000 14.769523000 5.085162000

C 8.408392000 13.523967000 4.423290000

Ag 8.388489000 11.198217000 2.506429000

F 4.917830000 7.673425000 1.401918000

F 5.577388000 6.758476000 3.244611000

F 6.882992000 6.795174000 1.535990000

O 7.139196000 12.833272000 0.905049000

O 5.789879000 9.432355000 3.516928000

O 7.535917000 9.267967000 2.092717000

N 7.450449000 14.067461000 1.132865000

C 6.655383000 14.853327000 1.891317000

H 5.759237000 14.378053000 2.264208000

C 8.598089000 14.563630000 0.623904000

H 9.165185000 13.855722000 0.037999000

C 7.007421000 16.156212000 2.161711000

H 6.351068000 16.745049000 2.788592000

C 8.980718000 15.859911000 0.871807000

H 9.914422000 16.212091000 0.453612000

C 5.974751000 7.490991000 2.207408000

C 6.501165000 8.876670000 2.663074000

C 8.183609000 16.680418000 1.653615000

H 8.477916000 17.698426000 1.871077000

**8.4 xyz coordinates for silver(I) and N-O∙∙∙π interaction**

E=-1901.22153109 a.u.

Ag 0.977064000 11.548934000 6.128381000

F 4.997244000 14.132200000 7.052624000

F 4.975554000 15.253146000 5.218029000

F 3.587374000 15.746891000 6.796973000

O 0.316245000 10.249166000 7.979499000

O 2.724834000 14.082773000 4.461420000

O 2.769679000 12.871474000 6.364232000

N -0.889494000 9.842176000 8.151874000

C -1.470733000 9.998214000 9.364689000

H -0.834986000 10.469693000 10.099734000

C -1.582881000 9.258117000 7.147247000

H -1.059258000 9.200934000 6.203022000

C -2.758943000 9.576079000 9.594875000

H -3.181747000 9.722906000 10.580395000

C -2.875255000 8.826422000 7.343606000

H -3.391490000 8.373762000 6.506679000

C 4.208795000 14.733562000 6.170149000

C 3.129943000 13.776256000 5.603181000

C -3.486916000 8.978775000 8.577201000

H -4.502635000 8.644190000 8.741795000

Ag 1.166940000 12.952035000 3.595625000

F -2.234435000 8.916695000 3.613179000

F -1.571967000 9.503342000 1.649828000

F -2.973409000 10.738398000 2.735025000

O -0.322431000 10.400897000 4.587078000

O -0.352635000 11.728515000 2.769569000

C -1.890745000 9.961032000 2.857169000

C -0.725647000 10.772690000 3.479282000

C -2.034304000 12.833346000 6.433437000

C -0.975703000 13.339180000 7.182376000

C -2.327681000 13.375521000 5.191349000

H -0.752856000 12.927196000 8.160301000

H -3.143820000 12.974580000 4.602141000

C -0.215294000 14.393440000 6.686537000

C -1.569413000 14.429060000 4.698157000

H 0.610002000 14.786299000 7.268148000

H -1.801454000 14.851561000 3.727380000

C -0.514294000 14.938895000 5.444079000

H 0.078597000 15.761177000 5.060899000

H -2.631606000 12.016345000 6.821529000

**8.5 xyz coordinates for π-π between PyNO and benzene**

E=-555.272758909 a.u.

O 0.957626000 9.791514000 7.091636000

N 1.581155000 10.667503000 6.430648000

C 2.695122000 10.342469000 5.721088000

H 2.965348000 9.299995000 5.788052000

C 3.398958000 11.288946000 5.016810000

H 4.286859000 10.973756000 4.483952000

C 2.983555000 12.611304000 5.005179000

C 1.844639000 12.932482000 5.726338000

H 1.467254000 13.947036000 5.758638000

C 1.164882000 11.962320000 6.424559000

H 0.275288000 12.135196000 7.012024000

C 5.945547000 11.416808000 7.682000000

H 6.757480000 11.977123000 7.232097000

C 5.983152000 10.029928000 7.696379000

H 6.825825000 9.505818000 7.259310000

C 4.942564000 9.313481000 8.271628000

H 4.969600000 8.229606000 8.279453000

C 3.864822000 9.982157000 8.834148000

H 3.039065000 9.425109000 9.259428000

C 3.830321000 11.369309000 8.821647000

H 2.984288000 11.890800000 9.254260000

C 4.868474000 12.086292000 8.245596000

H 4.835834000 13.169783000 8.230570000

H 3.531099000 13.365907000 4.456677000

**8.6 xyz coordinates for π-π between PyNOs**

E=-646.431705147 a.u.

O 3.528728000 5.121112000 6.498417000

N 3.999511000 4.427282000 5.553530000

C 4.175324000 3.086046000 5.696602000

C 4.653127000 2.313053000 4.665195000

H 4.769550000 1.249528000 4.833133000

C 4.963700000 2.885680000 3.441485000

C 4.790840000 4.254420000 3.307196000

H 4.995435000 4.760402000 2.373407000

C 4.320563000 4.999880000 4.362052000

H 3.899866000 2.712987000 6.671662000

H 4.154554000 6.066095000 4.331611000

H 5.327440000 2.285751000 2.618189000

O 1.980869000 4.768887000 1.778727000

N 1.494277000 4.287946000 2.841019000

C 1.298099000 2.948647000 2.971365000

C 0.808485000 2.407595000 4.136572000

H 0.678368000 1.333681000 4.187614000

C 0.504383000 3.220436000 5.217331000

C 0.697846000 4.585281000 5.072313000

H 0.499182000 5.272167000 5.883759000

C 1.179996000 5.094710000 3.890191000

H 1.570615000 2.381526000 2.093979000

H 1.363107000 6.142325000 3.704539000

H 0.132860000 2.804935000 6.144287000
